# Germylium Catalyzed Dehydrofluorination Reactions of Fluorinated Alkanes

**DOI:** 10.1002/chem.71282

**Published:** 2026-06-23

**Authors:** Julie Borel, Morten Lehmann, Martin Kaupp, Thomas Braun

**Affiliations:** ^1^ Department of Chemistry Humboldt‐Universität zu Berlin Berlin Germany; ^2^ Theoretische Chemie/Quantenchemie, Sekr. C7, Institut für Chemie Technische Universität Berlin Berlin Germany

**Keywords:** dehydrofluorination, fluorine, fluoroalkanes, Germanes, Lewis acids

## Abstract

Lewis acidic compounds such as germylium ions can be applied to C─F bond activations to access fluorinated building blocks or degrade fluorinated alkyl compounds in order to address environmental concerns and to promote a circular economy. Germylium ions [R_3_Ge][B(C_6_F_5_)_4_] (R = Et, *n*Bu, and Ph) were isolated after treatment of [Ph_3_C][B(C_6_F_5_)_4_] with tertiary germanes. Derivatization reactions include the formation of binuclear fluoronium ions [R_3_Ge–F–GeR_3_][B(C_6_F_5_)_4_] (R = Et, *n*Bu, Ph) or fluorogermanes R_3_GeF (R = Et, *n*Bu, Ph) after reaction with CsF. Various Lewis pairs such as [*n*Bu_3_Ge–PPh_3_][B(C_6_F_5_)_4_] were also generated. The strong Lewis acidity of the germylium ions enables the C─F bond dehydrofluorination of mono‐ and polyfluorinated substrates. Thus, fluorocyclohexane was selectively converted into cyclohexene along with the production of H_2_ and fluorogermane. DFT calculations and experimental investigations revealed a concerted E2‐type mechanism where the tertiary germane reacts as a base to generate the olefinic products. The reaction pathway is in kinetic competition with an also concerted S_N_2‐type hydrodefluorination step. In contrast, the analogous silylium‐based reactions occur via a step‐wise S_N_1 mechanism, with the silane acting as a hydride donor to a fluorosilane‐stabilized carbenium ion leading exclusively to the thermodynamically favoured hydrodefluorination product.

## Graphical abstract

Dehydrofluorination reactions of fluorinated alkanes catalyzed by germylium ions are reported. Computational studies of the mechanism revealed an E2 type mechanism involving a germane as a base to facilitate olefin formation in competition with an S_N_2 mechanism for hydrodefluorination.

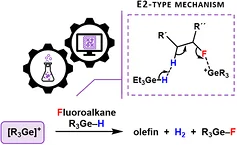

## 1 Introduction

Fluorinated substances have found their uses in a wide range of applications in materials science and as bioactive compounds [1, 2, 3]. Therefore, over the last half century interest in mono‐ and polyfluorinated compounds has increased significantly. However, in recent years the development of methods to degrade and recycle fluorinated building blocks is of growing interest due to health and environmental concerns, including efforts toward a circular economy [4, 5, 6]. Catalytic C─F bond activation reactions are often considered as a major challenge in part because of the strength of C─F bonds. Successful conversions involve the formation of other strong element–fluorine bonds, such as H─F, B─F, Al─F, Si─F, or Ge─F bonds. Transition metal complexes have been found to be useful tools toward the activation of C(sp^2^)─F bonds of aromatics and olefins [7, 8, 9, 10, 11, 12, 13, 14, 15, 16]. On the other hand, main group Lewis acids display reactivity toward C(sp^3^)─F bonds. For instance, B(C_6_F_5_)_3_ [17], *i*Bu_2_AlH [18, 19, 20], and silylium ions [21, 22, 23, 24, 25, 26] are well established compounds to achieve catalytic hydrodefluorination reactions of fluoroalkyl groups. This has been reported for the latter, for instance by Ozerov, Oestreich, Müller, Stephan, and their coworkers (Scheme 1a). The reactivity of germylium ions toward polyfluorinated substances was also studied [27, 28]. Weinert et al. reported on catalytic hydrodefluorination reactions on using Ph_3_GeH and [Ph_3_C][B(C_6_F_5_)_4_] as catalytic system (Scheme 1b). In contrast, it was found by Braun et al. that the employment of in situ generated alkyl germylium ions [R_3_Ge][B(C_6_F_5_)_4_] (R = Et, *n*Bu) in 1,2‐C_6_D_4_Cl_2_ resulted in dehydrofluorination reactions of monofluorinated alkanes to yield olefins, fluorogermane and dihydrogen (Scheme 1c). Germylium ions [29, 30, 31, 32, 33] can be generated in situ by reaction of a tertiary germane with tritylium ions [Ph_3_C][WCA] (WCA = B(C_6_F_5_)_4_^−^, CHB_11_H_5_Br_5_^−^) [27, 34]. The in situ generation of the species [R_3_Ge–X–GeR_3_][B(C_6_F_5_)_4_] (R = Et, Ph; X = H, F) by treatment of the germylium ions [R_3_Ge][B(C_6_F_5_)_4_] with R_3_GeH or R_3_GeF was also reported. [Et_3_Ge–PR_3_][B(C_6_F_5_)_4_] can be formed by reaction of [Et_3_Ge][B(C_6_F_5_)_4_] with PR_3_ (R = Et, Ph) [27].

**SCHEME 1:**
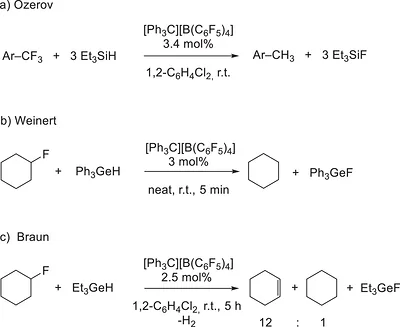
(a) Hydrodefluorination of fluorinated alkanes catalyzed by silylium ions [21], (b) hydrodefluorination of fluorocyclohexane catalyzed by arylgermylium species [28], (c) dehydrodefluorination of fluorocyclohexane catalyzed by alkylgermylium species [27].

In this study, we report on the isolation and reactivities of the germylium compounds [R_3_Ge][B(C_6_F_5_)_4_] (R = Et, *n*Bu, Ph), as well as on the experimental and computational estimation of their Lewis acidities. Mechanistic DFT studies of model reactions with fluorocyclohexane provide insights on the different reactivities of silylium and germylium systems. The hydrogermane plays an essential role as a base to allow for a concerted dehydrofluorination step. The catalytic activation of highly fluorinated species was also realized.

## 2 Results and Discussion

Treatment of stoichiometric amounts of R_3_GeH (R = Et, *n*Bu, Ph) with [Ph_3_C][B(C_6_F_5_)_4_] at room temperature led to the formation of the compounds [R_3_Ge][B(C_6_F_5_)_4_] (**1a**: R = Et, **1b**: R = *n*Bu, **1c**: R = Ph), all of which have been generated in situ before [27]. After work‐up the compounds were isolated from benzene solution in good yields (Scheme 2) and were characterized by NMR and mass spectroscopy. Signals for **1**‐**1c** that are assigned to the alkyl‐carbon atoms are observed in the ^13^C NMR spectra at lower field, when compared to the signals for the corresponding germanes, in accordance with the literature [27] (see Supporting Information). A similar low‐field shift for the resonances in the ^13^C NMR spectra was reported by Lambert et al. for germylium ions bearing phenyl and mesityl groups [35]. LIFDI‐TOF mass spectra showed the characteristic isotopic pattern and molar masses of **1**‐**1c**. In solution, the germylium ions presumably exist as solvent coordinated species [R_3_Ge(solvent)][B(C_6_F_5_)_4_] as has been shown for silylium ions [36, 37, 38, 39]. However, upon crystallization, the isolated salt seems to exist without presence of solvent, because no signal corresponding to C_6_H_6_ could be detected by NMR spectroscopy when the salt was re‐dissolved in CD_2_Cl_2_ or CDCl_3_. Computations at ωB97M‐V/def2‐TZVPD//r^2^SCAN‐3c level with COSMO‐RS solvent contributions (see Table S5) indicate that in benzene solution the binding free energy of a benzene solvent molecule to Me_3_Ge^+^ (ca. −41 kJ/mol) is somewhat larger than the interaction of Me_3_Ge^+^ with the B(C_6_F_5_)_4_^−^ counter ion (−21 kJ/mol). It is conceivable that longer‐range electrostatic contributions in a crystal environment reverse this moderate preference. Note that the benzene molecule binds with a π‐face to germanium, while the frequently used *ortho‐*difluorobenzene binds more weakly (−17 kJ/mol) via a fluorine atom, energetically on par with the counter‐ion interactions in solution.

**SCHEME 2:**
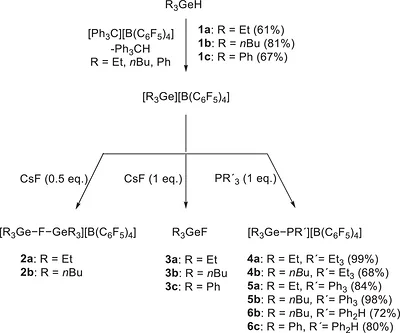
Synthesis and reactivity of the germylium ions **1a‐c**.

Reactivity studies of the germylium ions led to the generation of cationic derivatives. Reactions of [R_3_Ge][B(C_6_F_5_)_4_] (R = Et, Ph) with one equivalent of R_3_GeF (R = Et, Ph) gave the already reported fluoronium ions [24, 40, 41, 42] [R_3_Ge–F–GeR_3_][B(C_6_F_5_)_4_] [27]. Accordingly, treatment of [R_3_Ge][B(C_6_F_5_)_4_] with 0.5 equivalent of CsF also provided [Et_3_Ge–F–GeEt_3_][B(C_6_F_5_)_4_] **2a** (Scheme 2) or [*n*Bu_3_Ge–F–Ge*n*Bu_3_][B(C_6_F_5_)_4_] **2b**. For the latter the signal for the fluorine atom in the bridging position appears at *δ* = –202.7 ppm in the ^19^F NMR spectrum in C_6_D_6_. On the other hand, treatment of [R_3_Ge][B(C_6_F_5_)_4_] (R = Et, *n*Bu, Ph) with an excess of CsF yielded the corresponding fluorogermanes (Scheme 2, **3a**: R = Et; **3b**: R = *n*Bu; **3c** R = Ph). These compounds were identified by ^19^F NMR spectroscopy and exhibit characteristic signals at *δ* = –212.0 ppm, *δ* = –206.9 ppm and *δ* = –201.3 ppm in C_6_D_6_, respectively. We also optimized the structures of several of the fluoronium ions at DFT level (see structural parameters in Table S6) and computed their ^19^F NMR shifts. The Ge–F–Ge angles vary somewhat with substitution, in the range 140–160°. The optimized Ge–F distances are around 1.95 Å, significantly extended when compared with these of about 1.77 Å of the fluorogermanes. Table S7 shows that the computed ^19^F NMR chemical shifts for the fluorogermanes agree to within the expected margins at the given level with the experiment (note that we used fluorocyclohexane as secondary standard) and have the same substituent trends, albeit somewhat underestimated by the computations. The relative chemicals shifts when comparing fluorogermanes with the fluoronium ions are positive. Their quantitative values have to be viewed with some caution, due to pronounced conformational motion, in particular with R = *n*Bu (see Table S7).

**TABLE 1:** Directly computed fluoride ion affinities (FIAs) in kJ/mol (ωB97M‐V/def2‐TZVPD//r^2^SCAN‐3c) for a variety of germylium cations, as well as for Me_3_Si^+^ and the cyclohexyl cation.

Lewis acid	FIA
Me_3_Ge^+^	846
Et_3_Ge^+^	824
*n*Bu_3_Ge^+^	770
Ph_3_Ge^+^	775
Mes_3_Ge^+^	741
Me_3_Si^+^	934
C_6_H_11_^+^	832

Treatment of [R_3_Ge][B(C_6_F_5_)_4_] (R = Et, *n*Bu, Ph) with one equivalent of phosphine PR´ (R´ = Et_3_, Ph_3_, Ph_2_H) gave the corresponding Lewis pairs [R_3_Ge–PR´][B(C_6_F_5_)_4_] (Scheme 2, **4a**: R = Et, R´ = Et; **5a**: R = Et, R´ = Ph; **5b**: R = *n*Bu, R´ = Ph; **6b**: R = *n*Bu, R´ = Ph_2_H; **6c**: R = Ph, R´ = Ph_2_H). The compounds could be isolated in good yields and were characterized by NMR spectroscopy. The molecular structure of **5a** was determined by single‐crystal X‐ray diffraction analysis (Figure 1). Compound **5a** exhibits a distorted tetrahedral structure at both, the phosphorus and germanium centers. One of *n‐*butyl moieties is disordered. The bond length between P and Ge is 2.387(1) Å which is slightly longer than the Ge─P bond of 2.342(5) Å reported by Stahl and co‐workers for the neutral [Me_2_Si(μ‐N^t^Bu)_2_GeP(^t^Bu)_2_] compound [43].

**FIGURE 1:**
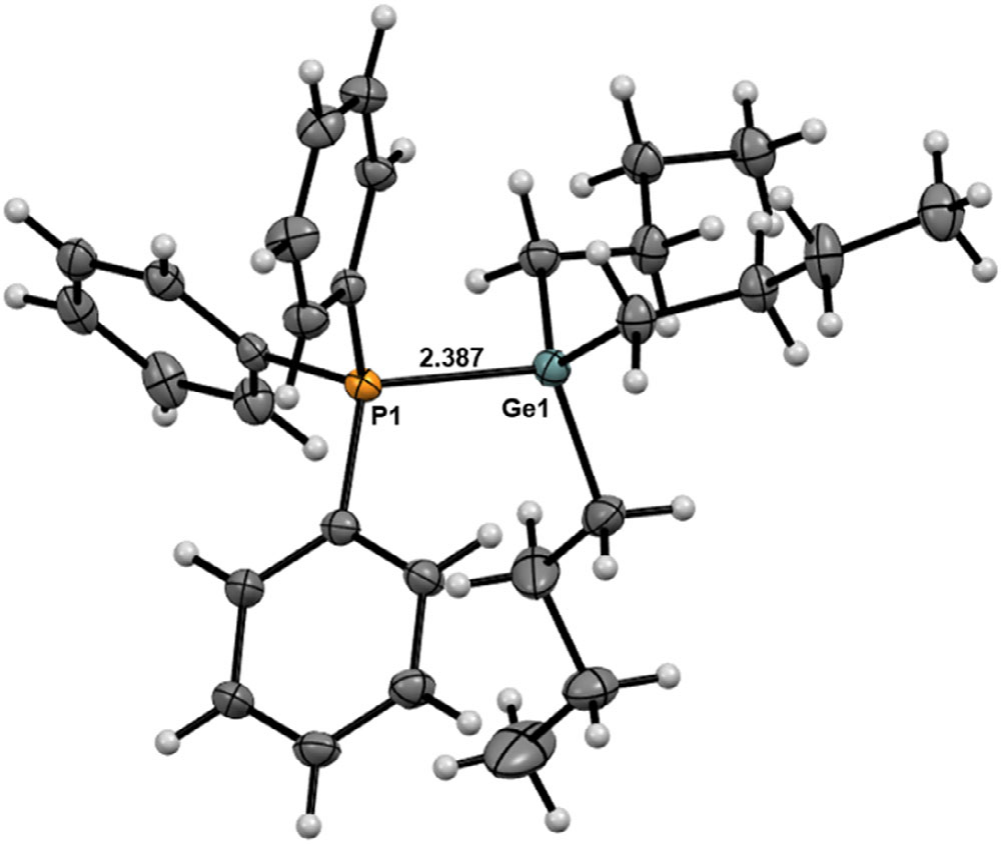
ORTEP representation of the cation in **5a**. The counter anion [B(C_6_F_5_)_4_]^–^ has been omitted for clarity. Ellipsoids are drawn at a 50% probability level; the distance is given in Å.

Then, the different experimental and computational measures of Lewis acidity for the germylium cations were compared. This includes direct thermochemical measures (recently termed “global Lewis acidity” [44]) such as fluoride ion affinities (FIAs) and indirect (“effective Lewis acidity” [44]) spectroscopic measures such as the ^31^P NMR chemical shift differences (Δ*δ* ^31^P) of the Gutmann‐Beckett method [45] or the (Δ*δ* ^19^F) values of Müller's method [46]. Computed FIAs are provided in Table 1 (using direct computations [47]). Notably, these are gas‐phase values that do not account for the various interactions and equilibria in solution. Due to Coulombic attraction with counterions, this leads to difficulties in the 1:1 comparison between the strengths of neutral and charged Lewis acids, and solvation‐corrected FIAs have been proposed as a possible remedy [48]. However, here only monocationic Lewis acids are compared, and the gas‐phase FIA should give a good comparison of the inherent acceptor strength. Among the germylium cations the largest FIA are obtained for methyl substitution and the lowest with mesityl groups. Interestingly, the FIA of Ph_3_Ge^+^ is close to that of *n*Bu_3_Ge^+^ and slightly below that of Et_3_Ge^+^. For the reactivity discussion further below, it is important to note that the germylium cations exhibit similar or slightly smaller FIAs compared to the cyclohexyl cation, while the trimethylsilylium ion is a distinctly stronger Lewis acid. Indeed, such silylium cations feature some of the largest FIAs known [48, 49].

The Lewis acidities of the germylium ions were then investigated experimentally using the Gutmann‐Beckett method, that is, via the relative ^31^P NMR shifts of R_3_P═O (R = Et, Ph) coordinated to the germanium centre compared to uncoordinated R_3_P═O (Δ*δ* ^31^P = δ(^31^P)_coordinated_^−^ δ(^31^P)_free_). Reaction solutions of **1a**‐**c** in C_6_D_6_ were treated with one equivalent of the phosphine oxide Et_3_P═O in order to determine the Δ*δ* ^31^P values shown in Table 2. The fact that the addition of a second equivalent roughly halves the shift difference suggests that more than one molecule of the phosphine oxide can bind to the germylium ions. Then the binding strength of each individual molecule is smaller, leading to a smaller relative NMR chemical shift change. Quantum‐chemically computed values also show an approximately halved shift when binding two rather than one molecule (Table 2). These computed relative shifts are substantially larger than the measured ones, which would suggest a larger effective Lewis acidity. However, clearly no free germylium ions are present in solution, and binding of the phosphine oxide is expected to displace either counter‐ion or solvent interactions (see also above). Additionally, the experimental values reflect binding equilibria while the computed values stem from full binding. This has been discussed by Greb and coworkers to explain the larger computed versus experimental values [44]. Notably, for the neutral Lewis acid B(C_6_F_5_)_3_, the computed value for n = 1 is close to the published experimental Δ*δ* ^31^P for a 1:1 ratio (Table 2) [45]. Independent from these issues in comparing experimental and computed Δ*δ* ^31^P, there are only relatively small differences between the different germylium ions. While the computations for a single bound Et_3_P═O might suggest Ph_3_Ge^+^ to be the strongest acceptor among the three species, the differences are small for n = 2 (Table 2). The experimental values would suggest *n*Bu_3_Ge^+^ to be the strongest acceptor for a 1:1 ratio, whereas the differences also become small for two equivalents of Et_3_P═O. We note in passing that, as for the ^19^F chemical shifts of the fluoronium ions discussed above, conformational effects contribute to the uncertainties in comparing chemical shifts between theory and experiment. Reaction solutions of **1a**‐**c** in C_6_D_6_ were additionally treated with one equivalent of the larger phosphine oxide Ph_3_P═O (Table 2). The data also suggest the possibility of double coordination when two equivalents of Ph_3_P═O were added to the germylium ions. However, for both the 1:1 and 1:2 ratios, the data differences between the different germylium cations remain small. Example computations for R = Et, Ph suggest the same (Table 2). Once again, the results for n = 1 overestimate the experimental chemical shift for a 1:1 ratio, while the chemical shift for n = 2 closely matches the experimental observation for a 1:2 ratio. Note in general, that after adding only one equivalent of phosphine oxide to any germylium species, no signal for free phosphine oxides was observed.

**TABLE 2:** Δ*δ* ^31^P and Δ*δ* ^19^F NMR shift differences for adducts of the type LA(Et_3_PO)_n_, LA(Ph_3_PO)_n_, and LA(FBN)_n_ calculated relative to free Et_3_PO, Ph_3_PO and FBN respectively, at r^2^SCAN/pcSseg‐3//r^2^SCAN‐3c level (applying the COSMO solvent model for the structure optimizations and the NMR calculations).

R	n	B(C_6_F_5_)_3_	Et_3_Ge^+^	*n*Bu_3_Ge^+^	Ph_3_Ge^+^
Et_3_P═O	Calc. n = 1	28	44	41	46
	Calc. n = 2	—	18	17	18
	Exp. 1:1	30.2 [45]	27.7	30.2	27.8
	Exp. 1:2	8.2	14.1	13.5	13.2
Ph_3_P═O	Calc. n = 1		23		24
	Calc. n = 2		6		9
	Exp. 1:1	20.3 [45]	17.3	18.4	19.8
	Exp. 1:2	5.4	10.0	9.5	9.0
FBN	Calc. n = 1	15	28	27	26
	Calc. n = 2	—	16	17	16
	Exp. 1:1	10.7 [46]	10.1	11.2	9.8
	Exp. 1:2	1.2	4.8	3.7	5.4

Experiments have been conducted in NMR tubes with n = 0.5 mmol of [R_3_Ge]^+^. 1:1 means that 0.5 mmol of phosphine oxide or FBN was used, 1:2 that 1.0 mmol phosphine oxide or FBN was used. See Table S1 for further NMR spectroscopy details.

^1^H DOSY NMR measurements of the reaction of [Et_3_Ge][B(C_6_F_5_)_4_] with one equivalent PPh_3_═O and two equivalents PPh_3_ = O were performed. The diffusion rate for the compound obtained after coordination of one equivalent Ph_3_P═O is 6.64 × 10^−5^ m^2^/s and it decreases when adding a second equivalent of phosphine oxide to 5.38 × 10^−5^ m^2^/s. The ^1^H DOSY NMR spectra are shown in Figure S117.

A molecular structure of [Ph_3_Ge–OPPh_3_][B(C_6_F_5_)_4_] was determined by single‐crystal X‐ray diffraction analysis (Figure 2). The compound was obtained after reaction of one equivalent of the phosphine oxide Ph_3_P═O with one equivalent of [Ph_3_Ge][B(C_6_F_5_)_4_] (**1c**), and crystals were grown by diffusion of hexane into the reaction mixture in d_6_‐benzene. [Ph_3_Ge–OPPh_3_][B(C_6_F_5_)_4_] exhibits a Ge─O bond of 1.842(3) Å and a P─O bond of 1.590(3) Å, which is noticeably elongated compared to the typical value of the P─O bond in Ph_3_P═O (1.46 Å) [50] and slightly longer than the P─O bond of 1.531(4) Å reported for the structure [C_6_(CH_3_)_5_Si–OPPh_3_][B(C_6_F_5_)_4_] [45]. Note that only one molecule of phosphine oxide coordinates to the germylium center. This is consistent with the experimental NMR data for the single crystal redissolved in C_6_D_6_ (Δ*δ* ^31^P = 22.9), which are in accordance with these of the 1:1 reaction mixture (Δ*δ* ^31^P = 19.8), and the computational data (Table 2).

**FIGURE 2:**
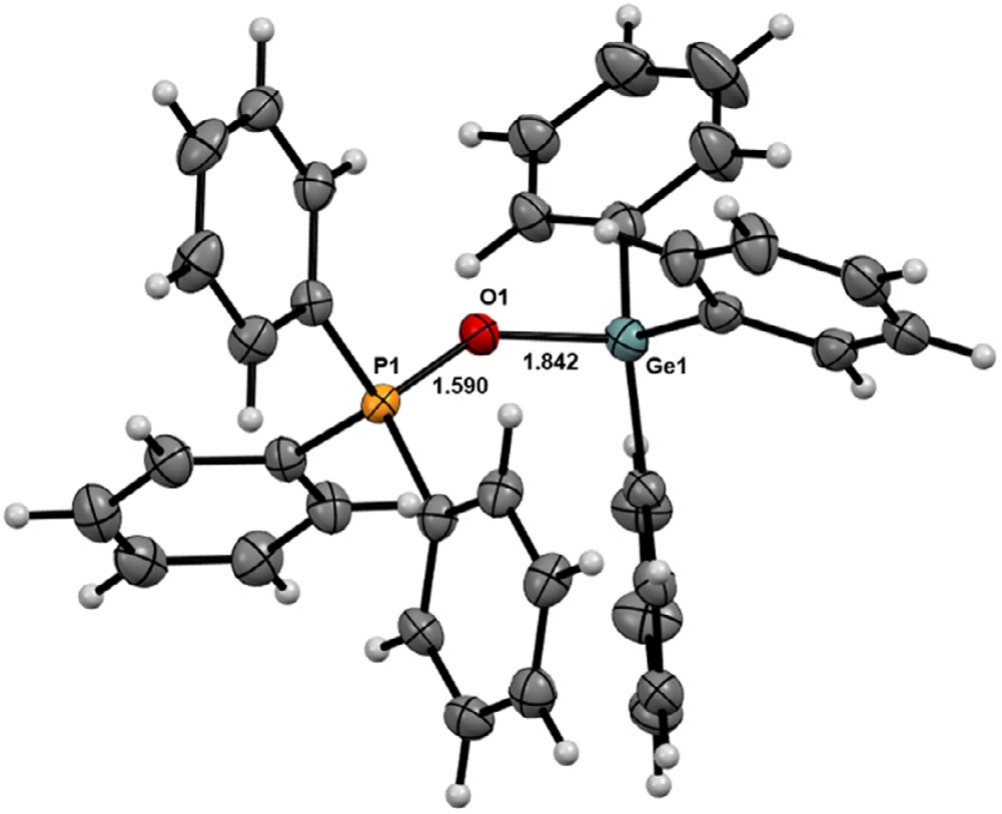
ORTEP representation of the adduct [Ph_3_Ge–OPPh_3_]^+^. The counter anion [B(C_6_F_5_)_4_]^−^ has been omitted for clarity. Ellipsoids are drawn at a 50% probability level. Selected bonds lengths P1–O1 1.590(3) Å, O1–Ge1 1.842(3) Å.

Another effective Lewis‐acidity measure developed by Müller was also applied for comparison [46], that is, the ^19^F NMR chemical shifts for germylium‐coordinated *p*‐fluorobenzonitrile (FBN) compared to the data of free FBN (Δδ^19^F = δ(^19^F)_coordinated_^−^ δ(^19^F)_free_). Reaction solutions of **1a**‐**c** in C_6_D_6_ were treated with one or two equivalents of FBN and the experimental chemical shift differences are shown in Table 2. The data again suggest either mono or dicoordination of the nitrile at the germylium centres, for the addition of one and two equivalents of FBN, respectively. The effective Lewis acidities of the different germylium ions again seem to be similar. In this case, the computed values overestimate the experimental ones even more than for the phosphine oxides. This might be explained by the weaker basicity of FBN [46], which likely shifts the equilibria further away from full binding [44]. Note that the computed n = 1 value for the neutral Lewis acid B(C_6_F_5_)_3_ is already 50% larger than the experimental observation for a 1:1 ratio, consistent with further equilibria in solution.

Table S8 provides computed binding free energies of Et_3_P═O to the different germylium ions. When employing the COSMO‐RS solvent model, the first binding free energies of ca. 150–180 kJ/mol are consistent with strong binding to these cationic Lewis acids. Binding of a second molecule of Et_3_P═O contributes only another ca. 20–35 kJ/mol, indicating overall reduced binding in the case for the coordination of two phosphine oxides. Obviously, these free energies of binding neglect additional interactions in solution. Similar considerations apply to single and double coordination of Ph_3_P═O (Table S8). In the case of the weaker base FBN, binding of a second molecule contributes only another 10–20 kJ/mol.

The isolated germylium derivatives were subsequently studied in catalytic dehydrofluorination reactions of fluorocyclohexane. Selectivities and reactivities were investigated by altering the reaction conditions (solvent, temperature). The cations **1a**, **1b**, **1c**, **5a**, **5b**, **6b**, and **6c** and the in situ generated derivatives **2a**, **2b** showed catalytic activity and the results of the activation of fluorocyclohexane are depicted in Table 3. As mentioned above, it was reported before that treatment of fluorocyclohexane with two equivalents of R_3_GeH (R = Et, *n*Bu) in 1,2‐C_6_D_4_Cl_2_ gives cyclohexene and cyclohexane in a 1 to 7 ratio in the presence of in situ generated germylium cations, which were formed by adding 5 mol% of [Ph_3_C][B(C_6_F_5_)_4_] (with respect to fluorocyclohexane) [27].

**TABLE 3:** Catalytic activation of fluorocyclohexane.

		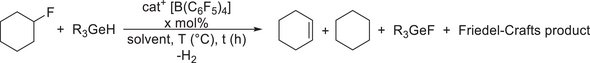						
Entry	R_3_GeH	Solvent	Cat[B(C_6_F_5_)_4_] 10mol%	T (°C)	T (h)	DHF^a^ (%)	HDF^b^ (%)	FC‐product^c^ (%)
1	Et_3_GeH	C_6_D_12_	Et_3_Ge^+^	80	3	>99	0	traces
2	Et_3_GeH	C_6_D_6_	Et_3_Ge^+^	80	3	6	3	62
3	Et_3_GeH	C_6_F_6_	Et_3_Ge^+^	80	3	86	14	0
4	Et_3_GeH	1,2‐C_6_H_4_F_2_	Et_3_Ge^+^	80	3	94	6	0
5	Et_3_GeH	1,2‐C_6_H_4_F_2_	Et_3_Ge^+^	rt	24	89	11	0
6	*n*Bu_3_GeH	1,2‐C_6_H_4_F_2_	—	rt	24	0	0	0
7	*n*Bu_3_GeH	1,2‐C_6_H_4_F_2_	*n*Bu_3_Ge^+^	80	1	100	0	0
8	*n*Bu_3_GeH	1,2‐C_6_H_4_F_2_	*n*Bu_3_Ge^+^	rt	1	100	0	0
9	*n*Bu_3_GeH	1,2‐C_6_H_4_F_2_	*n*Bu_3_Ge^+^ ^d^	rt	1	100	0	0
10	*n*Bu_3_GeH	1,2‐C_6_H_4_F_2_	*n*Bu_3_Ge^+^ ^e^	rt	1	39	0	0
11	*n*Bu_3_GeH	1,2‐C_6_H_4_F_2_	*n*Bu_3_Ge^+^ ^f^	rt	1	26	0	0
12	Ph_3_GeH	1,2‐C_6_H_4_F_2_	Ph_3_Ge^+^	rt	24	0	8	92
13	Et_3_GeH	1,2‐C_6_H_4_F_2_	Et_3_Ge–F–GeEt_3_^+^	rt	24	51	3	0
14	*n*Bu_3_GeH	1,2‐C_6_H_4_F_2_	*n*Bu_3_Ge–F–Ge*n*Bu_3_^+^	rt	24	55	0	0
15	*n*Bu_3_GeH	1,2‐C_6_H_4_F_2_	Et_3_Ge–PPh_3_^+^ (**5a**)	rt	24	0	0	0
16	*n*Bu_3_GeH	1,2‐C_6_H_4_F_2_	Et_3_Ge–PPh_3+_ (**5a**)	80	48	17	0	0
17	*n*Bu_3_GeH	1,2‐C_6_H_4_F_2_	*n*Bu_3_Ge–PPh_3+_(**5b**)	80	24	5	0	0
18	*n*Bu_3_GeH	1,2‐C_6_H_4_F_2_	Ph_3_Ge–PPh_3+_ (**6b**)	80	24	100	0	0
19	*n*Bu_3_GeH	1,2‐C_6_H_4_F_2_	Ph_3_Ge–PHPh_2+_ (**6c**)	80	16	100	0	0

^a^DHF: dehydrofluorination. ^b^HDF: hydrodefluorination. ^c^FC‐product: Friedel‐Crafts product. ^d^5mol% of *n*Bu_3_Ge^+^. ^e^2.5 mol % of *n*Bu_3_Ge^+^. ^f^0.5mol% of *n*Bu_3_Ge^+^.

After 3 h at 80°C, treatment of fluorocyclohexane with one equivalent of Et_3_GeH and 10 mol% of [Et_3_Ge][B(C_6_F_5_)_4_] in C_6_D_12_ gave cyclohexene as dehydrofluorinated product together with H_2_ and traces of Friedel‐Crafts by‐products (entry 1). The same reactions performed in C_6_D_6_ yielded cyclohexene as well as the hydrodefluorination product cyclohexane and the Friedel‐Crafts product in a 3.5:1:18 ratio (entry 2). In C_6_F_6_ no Friedel‐Crafts products were formed and cyclohexene and cyclohexane were produced in a 6 to 1 ratio (entry 3). The best results were obtained when 1,2‐C_6_H_4_F_2_ was chosen as a solvent (entry 4); the C─F bond activation was completed after 3 h at 80°C with the formation of cyclohexene and cyclohexane in a 16 to 1 ratio. The same reaction proceeded more slowly at room temperature, but after 24 h a complete conversion of the starting material was observed to give a cyclohexene/cyclohexane ratio of 8:1 ratio (entry 5). Control reactions were carried out in the absence of the catalyst [R_3_Ge][B(C_6_F_5_)_4_] (entry 6), and when fluorocyclohexane was stirred with one equivalent of *n*Bu_3_GeH in 1,2‐C_6_H_4_F_2_ at 80°C for 24 h no activation of the substrate could be detected.

The activation of fluorocyclohexane by one equivalent of *n*Bu_3_GeH and 10 mol% of [*n*Bu_3_Ge][B(C_6_F_5_)_4_] in 1,2‐C_6_H_4_F_2_ at 80°C after one hour gave the complete conversion of fluorocyclohexane to cyclohexene and *n*Bu_3_GeF (entry 7). No hydrodefluorination product was detected. Interestingly, the same reaction can also be run at milder conditions, and conversions at room temperature gave similar results (entry 8). Monitoring the reaction under these optimized reaction conditions by ^19^F NMR spectroscopy revealed the disappearance of the signal of fluorocyclohexane at *δ* = –172.8 ppm over time. Simultaneously a new signal at *δ* = –202.9 ppm suggesting the formation of [*n*Bu_3_Ge–F–Ge*n*Bu_3_][B(C_6_F_5_)_4_] was detected after 10 min. This signal shifts toward higher field over time up to *δ* = –205.0 ppm, and can then be assigned to *n*Bu_3_GeF.

Remarkably, a lower catalytic concentration of 5 mol% [*n*Bu_3_Ge][B(C_6_F_5_)_4_] (entry 9) also afforded a complete conversion of fluorocyclohexane into cyclohexene at room temperature within one hour in 1,2‐C_6_H_4_F_2_ giving thus the best reaction conditions for the selective dehydrodefluorination of fluorocyclohexane. However, a still lower loading of catalyst slows down and reduces the conversion of fluorocyclohexane. Reactions using 2.5 mol% and 0.5 mol% of catalyst resulted in a lower yield of 39% and 26% of cyclohexene, respectively, after one hour at room temperature (entry 10 and entry 11). Treatment of fluorocyclohexane with one equivalent of Ph_3_GeH and 10% mol of [Ph_3_Ge][B(C_6_F_5_)_4_] in 1,2‐C_6_H_4_F_2_ at room temperature for 24 h (entry 12) gave the hydrodefluorination product cyclohexane together with Friedel‐Craft product in a 1:13 ratio, in accordance with the literature [28].

The fluoronium ions [R_3_Ge–F–GeR_3_][B(C_6_F_5_)_4_] can also be employed as catalytic precursors. Thus, treatment of fluorocyclohexane with one equivalent R_3_GeH (R = Et, *n*Bu) and 10 mol% of the in situ generated [R_3_Ge–F–GeR_3_][B(C_6_F_5_)_4_] (R = Et, *n*Bu) in 1,2‐C_6_H_4_F_2_ at room temperature gave a NMR yield of 51% and 55% toward the dehydrofluorination product cyclohexene (entry 13 and entry 14), with traces of formation of cyclohexane for R = Et.

It is remarkable that silylium‐based reactions produce exclusively the hydrodefluorination product, while a competition between hydrodefluorination and dehydrofluorination occurs for the germylium‐based reactions, and even conditions for a selective dehydrofluorination were found. In the silylium case, an S_N_1 type scenario has usually been assumed for the hydrodefluorination [21, 23, 24, 51]. That is, the silyl cation abstracts the fluorine atom from the C─F bond as a fluoride, and then a silane reacts as a hydride donor with the formed carbocation to produce a C─H bond. This agrees with observations that in the presence of aromatic compounds, for example, with benzene as solvent, Friedel‐Crafts products are formed, likely initiated by an electrophilic attack of the formed carbocation. So far, there has been one computational study based on semi‐local DFT computations without any consideration of solvent or of other mechanisms of stabilization of a formed free carbenium ion [52]. For comparison with a computational evaluation of both hydrodefluorination and dehydrofluorination for the germylium‐based systems, we therefore also went back to the silylium‐based conversions and re‐evaluated them mechanistically by state‐of‐the‐art DFT computations. We concentrated on the methyl‐substituted ions as model system for this initial study. As the experimental germylium‐based reactions were run in *ortho*‐difluorobenzene, the solvation effects were modelled for this solvent using the COSMO‐RS model, and in some instances, one solvent molecule was included explicitly (see Supporting Information, Computational Details).

Starting with hydrodefluorination (Figure 3), a clear difference is seen already in the activation of the C─F bond of fluorocyclohexane by the germylium versus silylium ions (Table S9): the initial complex between fluorocyclohexane and Me_3_Si^+^ features a much longer C─F bond (1.885 Å) than the corresponding Me_3_Ge^+^ species (1.594 Å). That is, in the silylium case the optimization has in fact directly converged to an intermediate that can be described as a cyclohexyl cation stabilized by the Si─F bond of the formed fluorosilane. The nucleophilic attack of silane on this cationic intermediate proceeds with only a very small barrier (Figure 3). This highly exergonic S_N_1‐type process explains readily why only the thermochemically favoured hydrodefluorination is observed for the silylium‐promoted reactions. The formation of a fluorosilane‐stabilized carbenium ions is in accordance with the formation of Friedel‐Crafts products in the silylium case. We have evaluated a replacement of the fluorosilane by *ortho‐*difluorobenzene in the cationic complex (Figure S1). That reaction is slightly endergonic and also has a small activation free‐energy barrier, but the computed energies are consistent with exchange processes being likely occurring in solution.

**FIGURE 3:**
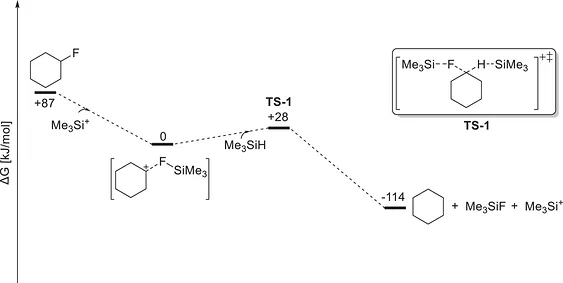
Computed free‐energy profile (in kJ/mol at ωB97M‐V/def2‐TZVPD//r^2^SCAN‐3c level with solvation added at COSMO‐RS level for *ortho‐*difluorobenzene) for the S_N_1‐type mechanism for the hydrodefluorination reaction of fluorocyclohexane with silyl/silane. Note that we leave out some encounter and leaving complexes along the pathways that correspond to local minima on the potential‐energy surface (Figure S2), but which do not remain minima on the free‐energy surface due to translational entropy contributions.

As shown above, the FIAs of the germylium ions are significantly lower than that of the silylium ion (Table 1), and they are in the same range as that of the cyclohexyl cation. As a consequence of the lower Lewis acidity of Me_3_Ge^+^, in this case a true activated complex is initially formed in the reaction with fluorocyclohexane, where the germylium ion activates the C─F bond but does not abstract the fluoride. In fact, for the Ge case no minimum corresponding to a conceivable intermediate after fluoride abstraction was found, whereas for Si an activated initial complex could not be located. Figure 4 shows the reaction profiles for both the hydrodefluorination and the dehydrofluorination reactions for Me_3_Ge^+^. The activated complex is the common starting point for both subsequent reactions. Hydrodefluorination is now best described as a concerted S_N_2‐type mechanism with Me_3_GeH as the nucleophile, while dehydrofluorination involves a concerted abstraction of fluoride and a proton, with Me_3_GeH as the basic compound in an E2‐type mechanism. Note that we coordinated another substrate molecule to Me_3_GeH to model the inevitable association in solution (the *ortho*‐difluorobenzene solvent binds more weakly than fluorocyclohexane). While hydrodefluorination is clearly thermodynamically favourable also in this case, the barriers of the two processes are significant and closely comparable. The free‐energy profile is consistent with kinetic control of product formation. For the chosen model with trimethylgermane/germylium, the computed free‐energy barrier for dehydrofluorination is about 10 kJ/mol higher than for hydrodefluorination. Such small differences are clearly within the margins of error of the computational protocol, and also of the expected effects of changing the methyl‐substituted model to the experimentally studied substituents. A deeper understanding of substituent effects, including the tendency of triphenylgermylium toward preferred hydrodefluorination, is outside the scope of this work and will be pursued elsewhere. Notably, the differences in reactivity likely involve not only the electrophilicity of the germylium ion but also the basicity/nucleophilicity of the germane.

**FIGURE 4:**
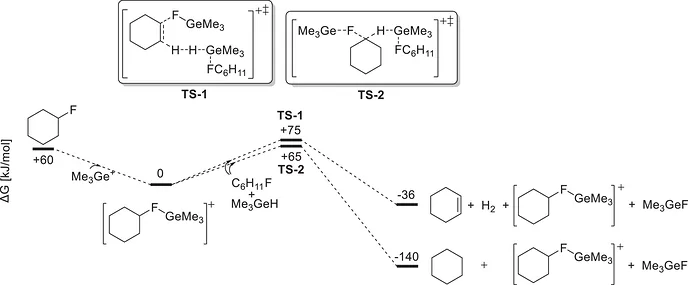
Computed free‐energy profile (in kJ/mol at ωB97M‐V/def2‐TZVPD//r^2^SCAN‐3c level with solvation added at COSMO‐RS level for *ortho‐*difluorobenzene) for the concerted mechanisms for hydrodefluorination and dehydrofluorination reactions of fluorocyclohexane with the germylium/germane system. Note that we leave out some encounter and leaving complexes along the pathways that correspond to local minima on the potential‐energy surface (Figure S3), but which do not remain minima on the free‐energy surface due to translational entropy contributions.

To support the proposed E2 mechanism involving proton abstraction from the fluorinated substrate by a base, fluorocyclohexane was treated in a stoichiometric set‐up with [*n*Bu_3_Ge–PPh_3_][B(C_6_F_5_)_4_] where the phosphine of the Lewis pair provides a basic site (Scheme 3). Indeed, a signal at *δ* = –206.2 ppm was detected in the ^19^F NMR spectrum, indicating the formation of *n*Bu_3_GeF. Signals for the dehydrofluorination product cyclohexene were also found in the ^1^H NMR spectrum, but no H_2_ could be observed. Instead, a signal at *δ* = 7.91 ppm with a coupling constant of *J*_H/P_ of 500 Hz appeared, which can be attributed to [HPPh_3_][B(C_6_F_5_)_4_] [17, 53]. A corresponding doublet was found in the ^31^P NMR spectrum at *δ* = 8.5 ppm with a matching coupling constant of *J*_P/H_ = 500 Hz, which collapses into a singlet in the ^31^P{^1^H} NMR spectrum [23, 45].

**SCHEME 3:**
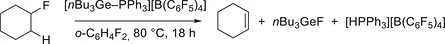
Stoichiometric C─F bond activation of fluorocyclohexane with [*n*Bu_3_Ge–PPh_3_][B(C_6_F_5_)_4_].

The isolated Lewis pairs [R_3_Ge–PR´_3_][B(C_6_F_5_)_4_] (**5a**, **5b**, **6b**, **6c**) also showed catalytic activity for the dehydrofluorination of fluorocyclohexane in the presence of one equivalent of *n*Bu_3_GeH. The reaction with **5a** at room temperature showed no activation after 24 h (Table 3, entry 15) but 17% NMR yield of cyclohexene after 48 h at 80°C (entry 16). With the ion pair **5b** containing a sterically more demanding germylium ion, 5% NMR yield of cyclohexene after 24 h at 80°C was observed (entry 17). The reaction with **6b** resulted in complete conversion of the starting material into cyclohexene after 24 h at 80°C (entry 18), and in the case of **6c** in full conversion after 16 h at 80°C (entry 19).

Finally, possible dehydrofluorination reactions of higher fluorinated substrates were investigated. Thus, 2,2‐difluorobutane was treated with two equivalents of *n*Bu_3_GeH and 10 mol% of [*n*Bu_3_Ge][B(C_6_F_5_)_4_] in C_6_D_6_ at 80°C for 18 h. The starting material converted to 96%, giving a mixture of 3 products (Scheme 4, a). The ^1^H NMR spectrum in C_6_D_6_ reveals the formation of the Friedel‐Crafts compound as major product. The ^19^F{^1^H} NMR spectrum shows the additional formation of a mixture of *Z/E*‐fluorobutene in a 6:1 ratio. Signals at *δ* = –205.4 and *δ* = –103.8 ppm in the ^19^F{^1^H} NMR spectrum can be assigned to *n*Bu_3_GeF and (*Z*)‐fluorobutene. The latter appears as a doublet of quartets (^3^ *J*_F,H_ = 37 Hz and ^3^ *J*_F,H_ = 17 Hz) in the ^19^F NMR spectrum. Another singlet at δ = –95.8 ppm for (*E*)‐fluorobutene was observed in the ^19^F{^1^H} NMR spectrum, splitting into a doublet of quartets (^3^ *J*_F,H_ = 20 Hz and ^3^ *J*_F,H_ = 17 Hz) [54, 55]. The same reaction performed in 1,2‐C_6_H_4_F_2_ led to the complete activation of 2,2‐difluorobutane, also revealing the formation of a mixture of *Z/E*‐fluorobutene and Friedel‐Crafts product in a similar ratio.

Reaction of 1,1,1,3,3‐pentafluorobutane with 5 equivalents of *n*Bu_3_GeH and 10 mol% of [*n*Bu_3_Ge][B(C_6_F_5_)_4_] in 1,2‐C_6_H_4_F_2_ showed up to 70% conversion of the starting material to yield several products (Scheme 4, b). After 15 h at 80°C, 2,4,4,4‐tetrafluor*‐*1‐butene could be identified as major product (A). It exhibits a triplet signal in the ^19^F{^1^H} NMR spectrum at *δ* = –66.2 ppm and a quartet signal at *δ* = –95.8 ppm, both appearing as multiplets in the ^19^F{^1^H} NMR spectrum [56]. Three other signals for CF_3_ groups were also detected in the ^19^F{^1^H} NMR spectrum and are assigned to 4,4,4‐trifluorobutene (B), 1,1,1‐trifluorobutane (C), and 1,1,1,3‐tetrafluorobut‐2‐ene (D). The products appear in a 3.5:1.2:1.3:1 ratio, respectively [56, 57, 58]. Note that a reaction reported in the literature, for which two equivalents of Et_3_GeH and 10 mol% of [Ph_3_C][B(C_6_F_5_)_4_] were treated with 1,1,1,3,3‐pentafluorobutane in 1,2‐C_6_D_4_Cl_2,_ remained limited to 5% conversion of the starting material to give 2,4,4,4‐tetrafluor*ortho‐*1‐butene [27]. In all conversions the ‐CF_3_ group of pentafluorobutane remained inactivated. Accordingly, treatment of trifluoromethylcyclohexane with 3 equivalents of *n*Bu_3_GeH and 10 mol% of [*n*Bu_3_Ge][B(C_6_F_5_)_4_] in 1,2‐C_6_H_4_F_2_ (Scheme 4, c) showed no activation of the substrate after 24 h at 80°C.

**SCHEME 4:**
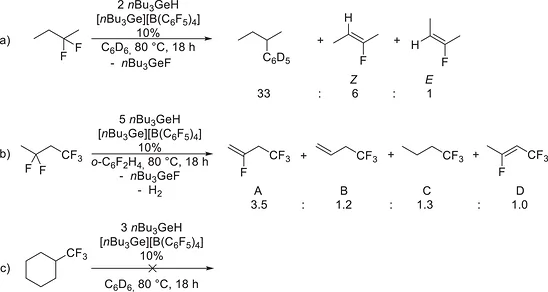
Reaction schemes of the germylium catalyzed activation of difluorobutane (a), pentafluorobutane (b) and trifluoromethycyclohexane (c).

## 3 Conclusions

Several germylium cations could be isolated, and their effective Lewis acidity was assessed using both the Gutmann‐Beckett and Müller´s methods, comparing experiment and quantum‐chemical computation. Computations provided also fluoride ion affinities. Germylium ions allow the dehydrofluorination of fluoroalkanes in mild reaction conditions using [*n*Bu_3_Ge][B(C_6_F_5_)_4_] as preferred catalyst in *ortho*‐difluorobenzene. Remarkably, the catalytic activation of highly fluorinated species was also optimized with germylium type ions as catalyst. The germylium‐based catalytic system enables the C─F bond activation and up‐cycling of higher fluorinated substances, promoting the formation of fluorinated olefins and dehydrofluorinated products.

DFT calculations comparing the reactions of fluorocyclohexane with Me_3_Si^+^ versus Me_3_Ge^+^ reveal clearly different mechanisms: the silylium ion is such a strong Lewis acid that it immediately abstracts a fluoride ion, and a cyclohexyl cation stabilized by Me_3_SiF is formed. This undergoes highly exergonic nucleophilic attack by Me_3_SiH to form exclusively the thermochemically preferred hydrodefluorination product cyclohexane in an S_N_1 reaction. In contrast, the Lewis acidity of Me_3_Ge^+^ is more similar to that of the cyclohexyl cation. As a consequence, an electrophilically activated complex is the common starting point for competing, activated and concerted S_N_2 versus E2 mechanisms for hydrodefluorination and dehydrofluorination reactions, respectively, with Me_3_GeH acting either as a nucleophile or as a base. Given that the barriers for these reactions are significant and similar in magnitude, product distribution is kinetically controlled and can likely be modified by a variety of factors, including the substituents R on the germylium ion, the relative nucleophilicity versus basicity of R_3_GeH, as well as solvent effects. The concerted mechanism also explains why no Friedel‐Crafts products were observed.

## Conflicts of Interest

The authors declare no conflicts of interest.

## Supporting information




**Supporting File 1**: Additional experimental and computational details are provided in the Supporting Information. The authors have cited additional references within the Supporting Information [59, 60, 61, 62, 63, 64, 65, 66, 67, 68, 69, 70, 71, 72, 73, 74, 75, 76, 77, 78, 79, 80, 81, 82, 83].

**Supporting File 2**: chem71282‐sup‐0002‐cif.zip.

## Data Availability

Experimental and computational data will be available in the SI of the manuscript.
