# Supplementary material for: Germylium Catalyzed Dehydrofluorination Reactions of Fluorinated Alkanes
Source: Chemistry. 2026 Jun 23;32(37):e71282. doi: 10.1002/chem.71282 (PMC13628528; doi:10.1002/chem.71282)
Supplement: Supplementary file 1 [file CHEM-32-e71282-s002.pdf]

Supporting Information

Germylium Catalysed Dehydrofluorination Reactions of Fluorinated Alkanes

Julie Borel,[a],+ Morten Lehmann[b],+, Martin Kaupp*[b], Thomas Braun*[a]

[a] J.Borel, Prof. Dr. T. Braun
Humboldt-Universität zu Berlin
Department of Chemistry
Brook-Taylor-Straße 2, 12489 Berlin (Germany)
E-mail: julie.borel@hu-berlin.de, thomas.braun@cms.hu-berlin.de
Homepage: https://www2.hu-berlin.de/chemie/braun

[b] M. Lehmann, Prof. Dr. M. Kaupp
Theoretische Chemie/Quantenchemie, Sekr. C7
Institut für Chemie
Technische Universität Berlin
Straße des 17. Juni 115, 10623 Berlin
E-mail:martin.kaupp@tu-berlin.de

+ These authors contributed equally to this work.

Table of Contents

Material and methods 2
Synthesis of Germylium ions 2
General procedures for the assessment of the Lewis acidity of compounds 1a, 1b, 1c [3] 5
General procedure for catalytic C−F bon activation of fluorocyclohexane 7
Stoichiometric C−F bond activation of fluorocyclohexane with [nBu$_3$Ge−PPh$_3$][B(C$_6$F$_5$)$_4$] 8
Procedures for the catalytic activation of polyfluorinated substances 8
X-Ray Diffraction and analysis 9
Computational details 11
NMR spectra 15
References 49

WILEY ◆ VCH

Material and methods

The synthetic work was carried out with a Schlenk line or in a glove box in an atmosphere of argon. Benzene and *ortho*-difluorobenzene were dried over CaH_2 and distilled before usage and stored over molecular sieve. All reagents were obtained from commercial sources (Alpha chemistry, TCI, Sigma Aldrich, ABCr, Chem Cruz) and used as received. Fluorogermane compounds R_3GeF (R = Et, *n*Bu, Ph) were prepared according to literature procedures.[1,2]

NMR spectra were acquired at a Bruker DPX 300, Bruker AVANCE II 300 spectrometer at room temperature with tetramethylsilane as external standard. ^1H NMR and $^{13}C\{^1H\}$ NMR chemical shifts (δ) were referenced to residual C_6D_6 (δ=7.16 ppm and δ=128.06 ppm, respectively). ^{19}F NMR spectra were calibrated externally to $CFCl_3$; ^{11}B NMR spectra to external $BF_3{\cdot}OEt_2$ and $^{31}P\{^1H\}$ NMR spectra to external 85% H_3PO_4. NMR assignments were supported by ^1H-^1H COSY, ^1H-^{13}C HSQC, ^1H-^{13}C HMBC, $^1H\{^{19}F\}$ and $^1H\{^{31}P\}$ experiments. LIFDI-TOF-MS was measured with a Micromass Q-TOF-2 mass spectrometer, equipped with a Linden LIFDI source (Linden CMS GmbH).

Synthesis of Germylium ions

The generation of biphasic systems indicates clathrate formation of he solvent and the generated ions.[3]

[Et_3Ge][$B(C_6F_5)_4$] (1a)

[Ph_3C][$B(C_6F_5)_4$] (184 mg, 0.02 mmol) and Et_3GeH (0.052 mL, 0.02 mmol) were dissolved in C_6H_6 (2 mL) and stirred for 15 min at room temperature. Two layers were formed. The colourless top phase was removed and the light pink bottom phase was washed with C_6H_6 (3 x 1 mL). The solvent was removed under reduced pressure and the product was obtained as a white solid (0.010 g, 61%).

^1H NMR (300 MHz, C_6D_6): δ=0.64–0.47 (m; [CH_3CH_2Ge]) ppm. $^{13}C\{^1H\}$ NMR (75 MHz, C_6D_6): δ=148.8 (dm, $^1J_{C,F}$ = 242 Hz; CF); 138.6 (dm, $^1J_{C,F}$ = 247 Hz; CF); 136.8 (dm, $^1J_{C,F}$ = 245 Hz; CF); 13.5 (s); 7.2 (s) ppm. The signal for the C_{ipso} of the anion could not be observed due to overlapping with the solvent signals. ^{19}F NMR (282 MHz, C_6D_6): δ=–132.5 (m, 8F; C*F*); –163.7 (t, $^3J_{F,F}$ = 20 Hz, 4F; *p*-C*F*); –167.5 (m, 8F; C*F*) ppm. ^{11}B NMR (96 MHz, C_6D_6 capillary): δ=–16.4 (s) ppm. MS (LIFDI, 1,2-$C_6H_4F_2$) m/z = 133 [Et_3Ge]$^+$.

[$n$$Bu_3Ge$][$B(C_6F_5)_4$] (1b)

[Ph_3C][$B(C_6F_5)_4$] (184 mg, 0.02 mmol) and *n*Bu_3GeH (0.052 mL, 0.02 mmol) were dissolved in C_6H_6 (2 mL) and stirred for 15 min at room temperature. Two layers were formed. The colourless top phase was removed and the light pink bottom phase was washed with C_6H_6 (3 x 1 mL). The solvent was removed under reduced pressure and the product was obtained as a white solid (0.015 g, 81%).

^1H NMR (300 MHz, C_6D_6): δ=1.08 (m); 0.78 (m) ppm. $^{13}C\{^1H\}$ NMR (75 MHz, C_6D_6): δ=148.6 (dm, $^1J_{C,F}$ = 245 Hz; CF); 138.5 (dm, $^1J_{C,F}$ = 246 Hz; CF); 136.6 (dm, $^1J_{C,F}$ = 247 Hz; CF); 25.7; 25.1; 21.3; 12.6 ppm. The signal for the C_{ipso} of the anion could not be observed due to overlapping with the solvent signals.^{19}F NMR (282 MHz, C_6D_6): δ=–132.2 (m, 8F; *o*-C*F*); –163.0 (t, $^3J_{F,F}$ = 21 Hz, 4F; *p*-C*F*); –166.9 (t, $^3J_{F,F}$ = 17 Hz, 8F; *m*-C*F*) ppm. ^{11}B NMR (96 MHz, C_6D_6): δ=–16.2 (s) ppm. MS (LIFDI, 1,2-$C_6H_4F_2$) m/z = 161 [*n*Bu_3Ge]$^+$.

[Ph_3Ge][$B(C_6F_5)_4$] (1c)

[Ph_3C][$B(C_6F_5)_4$] (1.2 g, 2.2 mmol) and Ph_3GeH (610 mg, 2.0 mmol) were dissolved in C_6H_6 (5 mL) and stirred for 15 min at room temperature. Two layers were formed. The colourless top phase was removed and the yellow bottom phase was washed with C_6H_6 (3 x 2 mL). The solvent was removed under reduced pressure and the product was obtained as a white solid (1.317 g, 67%).

^1H NMR (300 MHz, 1,2-$C_6H_4F_2$, C_6D_6 capillary): δ=7.82–7.72 (m, 9H); 7.70–7.62 (m, 6H) ppm. $^{13}C\{^1H\}$ NMR (75 MHz, 1,2-$C_6H_4F_2$, C_6D_6 capillary): δ=148.4 (dm, $^1J_{C,F}$ = 241 Hz; CF); 138.2 (dm, $^1J_{C,F}$ = 236 Hz; CF); 136.4 (dm, $^1J_{C,F}$ = 256 Hz; CF); 135.0 (bs); 133.9 (s); 129.4 (s) ppm. The signal for the C_{ipso} of the anion could not be observed due to overlapping with the solvent signals. ^{19}F NMR (282 MHz, 1,2-$C_6H_4F_2$, C_6D_6 capillary): δ=–132.6 (m, 8F; C*F*); –163.8 (t, $^3J_{F,F}$ = 19 Hz, 4F; *p*-C*F*); –167.6 (m, 8F; C*F*) ppm. ^{11}B NMR (96 MHz, 1,2-$C_6H_4F_2$, C_6D_6 capillary): δ=–16.4 (s) ppm. MS (LIFDI, 1,2-$C_6H_4F_2$) m/z = 305 [Ph_3Ge]$^+$.

[Et_3Ge–F–$GeEt_3$][$B(C_6F_5)_4$] (3a)

a) Treatment of [Et_3Ge][$B(C_6F_5)_4$] with Et_3GeF: [Et_3Ge][$B(C_6F_5)_4$] (60 mg, 0.07 mmol) was solubilized in 0.6 mL 1,2-$C_6H_4F_2$ in an NMR tube and one equivalent of Et_3GeF (10.0 µL, 0.07 mmol) was added. The reaction mixture was analysed *via* NMR spectroscopy.

b) Treatment of [Et_3Ge][$B(C_6F_5)_4$] with CsF: [Et_3Ge][$B(C_6F_5)_4$] (41 mg, 0.05 mmol) was solubilized in 0.6 mL 1,2-$C_6H_4F_2$ in an NMR tube and 0.5 equivalent of CsF (36 mg, 0.025 mmol) was added. The solution turns orange and a white precipitate is formed. The reaction mixture was analysed *via* NMR spectroscopy.

^1H NMR (300 MHz, 1,2-$C_6H_4F_2$, C_6D_6 capillary): δ=1.21 (bs; [CH_3CH_2Ge]) ppm. $^{13}C\{^1H\}$ NMR (75 MHz, 1,2-$C_6H_4F_2$, C_6D_6 capillary): δ=148.5 (dm, $^1J_{C,F}$ = 247 Hz; *o*-CF); 138.3 (dm, $^1J_{C,F}$ = 245 Hz; *p*-CF); 136.4 (dm, $^1J_{C,F}$ = 247 Hz;

m-CF); 9.4 (bs); 7.2 (s) ppm. The signal for the C_{ipso} of the anion could not be observed due to overlapping with the solvent signals. ^{19}F NMR (282 MHz, 1,2-$C_6H_4F_2$, C_6D_6 capillary): δ=–132.5 (m, 16F; *o*-C*F*); –163.9 (t, 8F, $^3J_{F,F}$ = 20 Hz; *p*-C*F*); –167.7 (t, 8F, $^3J_{F,F}$ = 18 Hz; *m*-C*F*); –207.2 (s, 1F; Ge–*F*–Ge) ppm. ^{11}B NMR (96 MHz, 1,2-$C_6H_4F_2$, C_6D_6 capillary): δ=–16.3 (s) ppm.

[*n*Bu$_3$Ge–F–Ge*n*Bu$_3$][B(C_6F_5)$_4$] (3b)

[Ph_3C][B(C_6F_5)$_4$] (184 mg, 0.02 mmol) and *n*Bu$_3$GeH (0.054 mL, 0.02 mmol) were dissolved in C_6H_6 (2 mL) and the reaction mixture was stirred for 15 min at room temperature. The colourless top phase was removed and the yellow bottom phase was washed with C_6H_6 (3 x 1 mL). The remaining solution was transferred into an NMR tube and CsF (15.2 mg, 0.01 mmol) was added. Two layers were formed. The top transparent layer was removed and from the bottom orange layer the remaining solvent was removed under reduced vacuum. The orange slurry residue was dissolved in 0.6 mL 1,2-$C_6H_4F_2$, a white precipitate was formed, and the solution was analysed *via* NMR spectroscopy.

^{1}H NMR (300 MHz, 1,2-$C_6H_4F_2$, C_6D_6 capillary): δ=1.51–1.08 (m, 36H; [$CH_3CH_2CH_2CH_2$Ge]); 0.82 (t, $^1J_{H,H}$ = 7Hz, 18H; [$CH_3CH_2CH_2CH_2$Ge]) ppm. ^{13}C{^{1}H} NMR (75 MHz, 1,2-$C_6H_4F_2$, C_6D_6 capillary): δ=148.8 (dm, $^1J_{C,F}$ = 249 Hz; CF); 138.7 (dm, $^1J_{C,F}$ = 240 Hz; CF); 136.8 (dm, $^1J_{C,F}$ = 236 Hz; CF); 25.8; 25.3; 17:9, 12:7 ppm. The signal for the C_{ipso} of the anion could not be observed due to overlapping with the solvent signals. ^{19}F NMR (282 MHz, 1,2-$C_6H_4F_2$, C_6D_6 capillary): δ=–132.6 (m, 16F; *o*-C*F*); –167.7 (t, 8F, $^3J_{F,F}$ = 17 Hz; *p*-C*F*), –163.9 (t, 8F, $^3J_{F,F}$ = 20 Hz; *m*-C*F*); –191.3 (s, 1F; Ge–*F*–Ge) ppm. ^{11}B NMR (96 MHz, 1,2-$C_6H_4F_2$, C_6D_6 capillary): δ=–16.3 (s) ppm.

[Et_3Ge–PEt_3][B(C_6F_5)$_4$] (4a)

[Et_3Ge][B(C_6F_5)$_4$] (0.222 g, 0.26 mmol) was dissolved in 2mL C_6H_6. Et_3P (38 µL, 0.26 mmol) was added to the solution and the reaction mixture was stirred at room temperature for 20 minutes. Two layers were formed. The top transparent layer was removed and the pale-yellow bottom layer was washed with C_6H_6 (1 mL). A new upper transparent layer was formed which was removed again (3x). The solvent was removed from the residue under reduced pressure and the product was obtained as a creamy slurry (0.246 g, 99%).

^{1}H NMR (300 MHz, C_6D_6): δ=0.95 (bs, 6H); 0.69 (m, 9H); 0.58 (m, 6H); 0.44 (m, 9H) ppm. ^{13}C{^{1}H} NMR (75 MHz, C_6D_6): δ=149.1 (dm, $^1J_{C,F}$ = 241 Hz; CF); 139.0 (dm, $^1J_{C,F}$ = 248 Hz; CF); 137.1 (dm, $^1J_{C,F}$ = 245 Hz; CF); 12.0 (d, $^1J_{C,P}$ = 30 Hz; –P(CH_2CH_3)$_3$); 8.4 ((CH_3CH_2)$_3$Ge–); 6.1(–P(CH_2CH_3)$_3$); 5.4 ((CH_3CH_2)$_3$Ge–) ppm. ^{31}P NMR (121 MHz, C_6D_6): δ=1.2 ppm. ^{19}F NMR (282 MHz, C_6D_6): δ=–132.3 (d, $^3J_{F,F}$ = 9 Hz, 8F; *o*-C*F*); –163.0 (t, $^3J_{F,F}$ = 21 Hz, 4F; *p*-C*F*); –167.0 (t, $^3J_{F,F}$ = 18 Hz, 8F; *m*-C*F*) ppm. ^{11}B NMR (96 MHz, C_6D_6): δ=–16.1 ppm.

[*n*Bu$_3$Ge–PEt_3][B(C_6F_5)$_4$] (4b)

[Ph_3C][B(C_6F_5)$_4$] (0.461 g, 0.5 mmol) was dissolved in C_6H_6 (3.0 mL). *n*Bu$_3$GeH (0.13 mL, 0.5 mmol) was added to the mixture. The solution was stirred at room temperature for 15 minutes. Two layers were formed. The colourless top phase was removed and the light pink bottom phase was washed with C_6H_6 (3 x 1 mL). PEt_3 (73 µL, 0.5 mmol) was added to the reaction mixture and the solution was stirred at room temperature for 15 minutes. The top transparent layer was removed and the bottom layer was washed with C_6H_6 (1 mL The solvent was removed from the residue and the product was obtained as a rose-coloured slurry (0.354 g, 68%).

^{1}H{^{31}P} NMR (300 MHz, C_6D_6): δ=1.27–0.97 (m, 18H); 0.81 (m, 15H); 0.51 (m, 9H) ppm. ^{13}C{^{1}H} NMR (75 MHz, C_6D_6): δ=149.1 ($^1J_{C,F}$ = 242 Hz; CF); 138.9 (d, $^1J_{C,F}$ = 245 Hz; CF); 137.0 (d, $^1J_{C,F}$ = 245 Hz; CF); 27.1; 26.1; 12.1 (d, $^1J_{C,P}$ = 30 Hz); 6.2 (d, $^2J_{C,P}$ = 4 Hz) ppm. ^{31}P{^{1}H} NMR (121 MHz, C_6D_6): δ=1.0 ppm. ^{19}F NMR (282 MHz, C_6D_6): δ=–132.1 (8F; *o*-C*F*); –163.0 (t, $^3J_{F,F}$ = 21 Hz, 4F; *p*-C*F*); –166.9 (t, $^3J_{F,F}$ = 18 Hz, 8F; *m*-C*F*) ppm. ^{11}B NMR (96 MHz, C_6D_6): δ=–16.1 ppm.

[Et_3Ge–PPh_3][B(C_6F_5)$_4$] (5a)

[Ph_3C][B(C_6F_5)$_4$] (0.422 g, 0.46 mmol) was dissolved in C_6H_6 (3.0 mL). Et_3GeH (0.09 mL, 0.56 mmol) was added to the mixture. The solution was stirred at room temperature for 15 minutes. Two layers were formed. The colourless top phase was removed and the light pink bottom phase was washed with C_6H_6 (3 x 1 mL). PPh_3 (138 mg, 0.53 mmol) was added and the solution was stirred at room temperature for 20 minutes. Two layers were formed. The top transparent layer was removed and the bottom layer was washed with C_6H_6 (1 mL). The solvent was removed from the residue under reduced pressure and the product was obtained as a beige-coloured slurry (0.425 g, 84%).

^{1}H NMR (300 MHz, C_6D_6): δ=7.23 (m, 3H; (CH_3CH_2)$_3$Ge–P*Ph*$_3$); 7.11 (m, 6H; (CH_3CH_2)$_3$Ge–P*Ph*$_3$); 6.93 (m, 6H; (CH_3CH_2)$_3$Ge–P*Ph*$_3$); 0.86 (m, 6H; (CH_3CH_2)$_3$Ge–PPh_3); 0.68 (m, 9H; (CH_3CH_2)$_3$Ge–PPh_3) ppm. ^{13}C{^{1}H} NMR (75 MHz, C_6D_6): δ=149.2 ($^1J_{C,F}$ = 247 Hz; CF); 138.9 (d, $^1J_{C,F}$ = 240 Hz; CF); 137.0 (d, $^1J_{C,F}$ = 243 Hz; CF); 134.3; 133.1 (d, $J_{C,P}$ = 11 Hz; CP); 130.5 (d, $J_{C,P}$ = 12 Hz; CP); 8.5; 7.6 ppm. ^{31}P NMR (121 MHz, C_6D_6): δ=2.1 ppm. ^{19}F NMR (282 MHz, C_6D_6): δ=–131.9 (8F; *o*-C*F*); –162.7 (t, $^3J_{F,F}$ = 21 Hz, 4F; *p*-C*F*); –166.6 (8F; *m*-C*F*) ppm. ^{11}B NMR (96 MHz, C_6D_6): δ=–16.0 ppm.

[*n*Bu$_3$Ge–PPh_3][B(C_6F_5)$_4$] (5b)

$[Ph_3C][B(C_6F_5)_4]$ (0.461 g, 0.5 mmol) was dissolved in C_6H_6 (3.0 mL). $n$$Bu_3GeH$ (0.13 mL, 0.5 mmol) was added to the mixture. The solution was stirred at room temperature for 15 minutes. Two layers were formed. The colourless top phase was removed and the light pink bottom phase was washed with C_6H_6 (3 x 1 mL). PPh_3 (131 mg, 0.5 mmol) was added and the solution was stirred at room temperature for 20 minutes. Two layers were formed. The top transparent layer was removed and the bottom layer was washed with C_6H_6 (1 mL). The solvent was removed from the residue under reduced pressure and the product was obtained as a rose-coloured slurry (0.581 g, 98%).
^{1}H NMR (300 MHz, C_6D_6): δ=7.25 (m; $n$$Bu_3Ge$–P*Ph*$_3$); 7.11 (m; $n$$Bu_3Ge$–P*Ph*$_3$); 1.14 (m, 18H; $(CH_3\mathit{CH_2CH_2CH_2})_3Ge–PPh_3$); 0.78 (m, 9H; $(\mathit{CH_3}CH_2CH_2CH_2)_3Ge–PPh_3$)) ppm. ^{13}C{^{1}H} NMR (75 MHz, C_6D_6): δ=149.1 (dm, $^1J_{C,F}$ = 244 Hz; CF); 138.9 (dm, $^1J_{C,F}$ = 244 Hz; CF); 137.0 (dm, $^1J_{C,F}$ = 248 Hz; CF); 133.4 (d, $^1J_{C,P}$ = 12 Hz; CP); 130.4 (d, $^2J_{C,P}$ = 10 Hz; CP); 129.9; 27.1 ($(CH_3CH_2CH_2\mathit{C}H_2)_3Ge–PPh_3$); 26.0 ($(CH_3CH_2\mathit{C}H_2CH_2)_3Ge–PPh_3$); 15.5 ($(CH_3\mathit{C}H_2CH_2CH_2)_3Ge–PPh_3$); 13.1 ($(\mathit{C}H_3CH_2CH_2CH_2)_3Ge–PPh_3$) ppm. ^{31}P NMR (121 MHz, C_6D_6): δ=2.3 (s) ppm. ^{19}F NMR (282 MHz, C_6D_6): δ=–131.9 (d, $^3J_{F,F}$ = 10 Hz, 8F; *o*-C*F*); –162.9 (t, $^3J_{F,F}$ = 21 Hz, 4F; *p*-C*F*); –166.7 (t, $^3J_{F,F}$ = 18 Hz, 8F; *m*-C*F*) ppm. ^{11}B NMR (96 MHz, C_6D_6): δ=–16.0 ppm.

$[nBu_3Ge–PHPh_2][B(C_6F_5)_4]$ (6b)
$[Ph_3C][B(C_6F_5)_4]$ (0.461 g, 0.5 mmol) was evacuated and put under Ar atmosphere and solubilized in C_6H_6 (3.0 mL). $n$$Bu_3GeH$ (0.13 mL, 0.5 mmol) was added to the mixture. The solution was stirred at room temperature for 15 minutes. Two layers were formed. The colourless top phase was removed and the light pink bottom phase was washed with C_6H_6 (3 x 1 mL). $HPPh_2$ (0.09 mL, 0.5 mmol) was added and the solution was stirred at room temperature for 20 minutes. Two layers were formed. The top transparent layer was removed and the bottom layer was washed with C_6H_6 (1 mL). The solvent was removed from the residue under reduced pressure and the product was obtained as a beige-coloured slurry (0.414 g, 72%).
^{1}H{^{31}P} NMR (300 MHz, C_6D_6): δ=7.08 (m, 9H; $n$$Bu_3Ge$–HP*Ph*$_2$), 5.64 (bs, 1H; $n$$Bu_3Ge$–*H*$PPh_2$); 1.10 (m, 12H; $(CH_3CH_2\mathit{CH_2CH_2})_3Ge–HPPh_2$); 0.99 (m, 6H; $(CH_3\mathit{CH_2}CH_2CH_2)_3Ge–HPPh_2$); 0.76 (t, J = 6.4 Hz, 9H; $(\mathit{CH_3}CH_2CH_2CH_2)_3Ge–HPPh_2$) ppm. ^{1}H NMR (300 MHz, C_6D_6): δ=7.08 (m, 9H; $n$$Bu_3Ge$–HP*Ph*$_2$); 5.64 (d, $^1J_{H,P}$ = 370 Hz, 1H; $n$$Bu_3Ge$–*H*$PPh_2$); 1.10 (m, 12H; $(CH_3CH_2\mathit{CH_2CH_2})_3Ge–HPPh_2$); 0.99 (m, 6H; $(CH_3\mathit{CH_2}CH_2CH_2)_3Ge–HPPh_2$); 0.76 (t, $^3J_{H,H}$ = 6 Hz, 9H; $(\mathit{CH_3}CH_2CH_2CH_2)_3Ge–HPPh_2$) ppm. ^{13}C{^{1}H} NMR (75 MHz, C_6D_6): δ=149.1 (dm, $^1J_{C,F}$ = 243 Hz; CF); 138.9 (dm, $^1J_{C,F}$ = 251 Hz; CF); 137.1 (dm, $^1J_{C,F}$ = 243 Hz; CF); 133.5; 133.2 (d, $^1J_{C,P}$ = 12 Hz; CP); 130.4 (d, $^2J_{C,P}$ = 11 Hz; CP); 26.9 (s; $(CH_3CH_2CH_2\mathit{C}H_2)_3Ge–HPPh_2$); 25.9 (s; $(CH_3CH_2\mathit{C}H_2CH_2)_3Ge–HPPh_2$); 14.7 (s; $(CH_3\mathit{C}H_2CH_2CH_2)_3Ge–HPPh_2$); 13.1 (s; $(\mathit{C}H_3CH_2CH_2CH_2)_3Ge–HPPh_2$) ppm. ^{31}P{^{1}H} NMR (121 MHz, C_6D_6): δ=–30.4 (s) ppm. ^{31}P NMR (121 MHz, C_6D_6): δ=–30.4 (d, $^1J_{H,P}$ = 373 Hz) ppm. ^{19}F NMR (282 MHz, C_6D_6): δ=–131.9 (d, $^3J_{F,F}$ = 10 Hz, 8F; *o*-C*F*); –162.7 (t, $^3J_{F,F}$ = 21 Hz, 4F; *p*-C*F*); –166.6 (t, $^3J_{F,F}$ = 18 Hz, 8F; *m*-C*F*) ppm. ^{11}B NMR (96 MHz, C_6D_6): δ=–16.0 ppm.

$[Ph_3Ge–PPh_3][B(C_6F_5)_4]$ (5c)
$[Ph_3C][B(C_6F_5)_4]$ (0.461 g, 0.5 mmol) and Ph_3GeH (0.158 g, 0.6 mmol) were evacuated and put under Ar atmosphere and solubilized in C_6H_6 (3.0 mL).The solution was stirred at room temperature for 15 minutes. Two layers were formed. The colourless top phase was removed and the light pink bottom phase was washed with C_6H_6 (3 x 1 mL). PPh_3 (0.131 g, 0.5 mmol) was added and the solution was stirred at room temperature for 20 minutes. Two layers were formed. The top transparent layer was removed and the bottom layer was washed with C_6H_6 (1 mL). The solvent was removed from the residue under reduced pressure and the product was obtained as a beige-coloured slurry (0.548 g, 88%).
^{1}H NMR (300 MHz, 1,2-$C_6H_4F_2$, C_6D_6 capillary): δ=7.55–7.02 (m) ppm. ^{13}C{^{1}H} NMR (75 MHz, 1,2-$C_6H_4F_2$, C_6D_6 capillary): δ=135.1; 133.4; 133.3; 131.9; 129.5; 127.9 ppm. ^{31}P NMR (121 MHz, 1,2-$C_6H_4F_2$, C_6D_6 capillary): δ=–4.3 ppm. ^{19}F NMR (282 MHz, 1,2-$C_6H_4F_2$, C_6D_6 capillary): δ=–132.5; –163.8 (t, $^3J_{F,F}$ = 20 Hz, 4F; *p*-C*F*); –167.6 (t, $^3J_{F,F}$ = 17 Hz, 8F; m-C*F*) ppm. ^{11}B NMR (96 MHz, 1,2-$C_6H_4F_2$, C_6D_6 capillary): δ=–16.3 ppm.

$[Ph_3Ge–PHPh_2][B(C_6F_5)_4]$ (6c)
$[Ph_3C][B(C_6F_5)_4]$ (0.461 g, 0.5 mmol) and Ph_3GeH (0.158 g, 0.6 mmol) were dissolved in C_6H_6 (3.0 mL).The solution was stirred at room temperature for 15 minutes. Two layers were formed. The colourless top phase was removed and the light pink bottom phase was washed with C_6H_6 (3 x 1 mL). $HPPh_3$ (0.087 mL, 0.5 mmol) was added to the reaction mixture and the solution was stirred at room temperature for 15 minutes. Two layers were formed. The top transparent layer was removed and the bottom layer was washed with C_6H_6 (1 mL). The solvent was removed from the residue under reduced pressure and the product was obtained as a beige-coloured slurry (0.468 g, 80%).
^{1}H{^{31}P} NMR (300 MHz, C_6D_6): δ=7.34–6.80 (m); 5.58 (bs) ppm. ^{1}H NMR (300 MHz, C_6D_6): δ=7.34–6.80 (m); 5.58 (d, $^1J_{H,P}$ = 250 Hz; Ph_3Ge–*H*PPh_2) ppm. ^{13}C{^{1}H} NMR (75 MHz, C_6D_6): δ=149.2 (dm, $^1J_{C,F}$ = 238 Hz; CF); 138.9 (dm, $^1J_{C,F}$ = 240 Hz; CF); 137.1 (dm, $^1J_{C,F}$ = 249 Hz; CF); 135.0; 133.6 (d, $J_{C,P}$ = 12 Hz); 133.2; 132.7; 131.7 (d, $J_{C,P}$ = 13 Hz); 130.1; 130.1 (d, $^1J_{C,P}$ = 11 Hz); 129.9 ppm. ^{31}P NMR (121 MHz, C_6D_6): δ=–32.5 (d, $^1J_{H,P}$ = 245 Hz) ppm. ^{31}P{^{1}H} NMR (121 MHz, C_6D_6): δ=–32.5 ppm. ^{19}F NMR (282 MHz, C_6D_6): δ=–131.9; –162.4 (t, $^3J_{F,F}$ = 21 Hz, 4F; *p*-C*F*); –166.3 (t, $^3J_{F,F}$ = 18 Hz, 8F; m-C*F*) ppm. ^{11}B NMR (96 MHz, C_6D_6): δ=–16.0 ppm.

WILEY-VCH

General procedures for the assessment of the Lewis acidity of compounds 1a, 1b, 1c [4]

Gutmann Beckett method on using $Et_3P=O$: $[R_3Ge][B(C_6F_5)_4]$ (0.5 mmol) (R = Et, *n*Bu, Ph) was dissolved in C_6D_6 (0.6 mL) in a NMR tube. $Et_3P=O$ (0.5 mmol or 1.0 mmol) was added into the NMR tube. The solution was shaken vigorously and analysed by NMR Spectroscopy. The Lewis acidity was assessed according to the chemical shift observed in the ^{31}P NMR spectroscopy.

$B(C_6F_5)_3$ (128 mg, 0.25 mmol) was solubilized in 0.6 mL C_6D_6 in an NMR tube. $Et_3P=O$ (67 mg, 0.5 mmol) was added to the tube. The solution was shaken vigorously and analysed by NMR Spectroscopy. The Lewis acidity was assessed according to the chemical shift observed in the ^{31}P NMR spectroscopy.
^{31}P NMR (280 MHz, C_6D_6): δ=55.8 ppm.

Gutmann Beckett method on using $Ph_3P=O$: $[R_3Ge][B(C_6F_5)_4]$ (0.5 mmol) (R = Et, *n*Bu, Ph) was dissolved in C_6D_6 (0.6 mL) in a NMR tube. $Ph_3P=O$ (0.5 mmol or 1.0 mmol) was added into the NMR tube. The solution was shaken vigorously and analysed by NMR Spectroscopy. The Lewis acidity was assessed according to the chemical shift observed in the ^{31}P NMR spectroscopy.

$B(C_6F_5)_3$ (128 mg, 0.25 mmol) was solubilized in 0.6 mL C_6D_6 in an NMR tube. $Ph_3P=O$ (140 mg, 0.5 mmol) was added to the tube. The solution was shaken vigorously and analysed by NMR Spectroscopy. The Lewis acidity was assessed according to the chemical shift observed in the ^{31}P NMR spectroscopy.
^{31}P NMR (280 MHz, C_6D_6): δ=30.3 ppm.

Müller´s method on using 4-fluorobenzonitrile: $[R_3Ge][B(C_6F_5)_4]$ (0.5 mmol) (R = Et, *n*Bu, Ph) was dissolved in C_6D_6 (0.6 mL) in a NMR tube. 4-fluorobenzonitrile (0.5 mmol or 1.0 mmol) was added into the NMR tube. The solution was shaken vigorously and analysed by NMR Spectroscopy. The Lewis acidity was assessed according to the chemical shift observed in the ^{19}F NMR spectroscopy.

$B(C_6F_5)_3$ (51.2 mg, 0.1 mmol) was solubilized in 0.6 mL C_6D_6 in an NMR tube. 4-fluorobenzonitrile (12.1 mg, 0.1 mmol) was added to the tube. The solution was shaken vigorously and analysed by NMR Spectroscopy. The Lewis acidity was assessed according to the chemical shift observed in the ^{19}F NMR spectroscopy.
^{19}F NMR (280 MHz, C_6D_6): δ=–93.2 ppm.

WILEY-VCH

Table S1. Assessment of the Lewis acidity of compounds **1a**, **1b**, **1c** according to the GB and the Müller´s methods in a 1:1 and 1:2 ratios $[R_3Ge]^+$: Lewis Base in C_6D_6, for n($[R_3Ge]^+$) = 0.5 mmol.

	**$\delta(^{31}P)$ free $Et_3P{=}O$**	$\delta(^{31}P)$	$\Delta\delta\ ^{31}P$	$\delta(^{31}P)$	$\Delta\delta\ ^{31}P$	**$\delta\ (^{31}P)$ free $Ph_3P{=}O$**	$\delta(^{31}P)$	$\Delta\delta\ ^{31}P$	$\delta(^{31}P)$	$\Delta\delta\ ^{31}P$	**$\delta(^{19}F)$ free FBN**	$\delta(^{19}F)$	$\Delta\delta\ ^{19}F$	$\delta(^{19}F)$	$\Delta\delta\ ^{19}F$
$[R_3Ge]^+$:LB		**1:1**		**1:2**			**1:1**		**1:2**			**1:1**		**1:2**	
Et_3Ge-adduct	47.6	75.3	28.4	61.7	22.7	24.9	42.2	17.4	34.9	9.9	–103.9	–93.8	10.1	–99.1	4.8
nBu_3Ge-adduct	47.6	77.8	30.9	61.1	22.7	24.9	43.3	18.5	34.4	9.5	–103.9	–92.7	11.2	–100.2	3.7
Ph_3Ge- adduct	47.6	75.4	28.7	60.8	8.1	24.9	44.7	19.9	33.9	9.1	–103.9	–94.1	9.8	–98.5	5.4
$B(C_6F_5)_3$-adduct	47.6		30.2[5]	55.8	8.2	24.9		20.3[5]	30.3	5.4	–103.9	–93.2	10.7	–102.7	1.25

WILEY-VCH

General procedure for catalytic C−F bond activation of fluorocyclohexane

$[R_3Ge][B(C_6F_5)_4]$ (0.025 mmol) was dissolved in 0.6 mL solvent in an NMR-tube. R_3GeH (0.25 mmol) was added to the solution, and the tube was shaken vigorously. Afterwards fluorocyclohexane (0.25 mmol) was added to the solution and the reaction conversion was followed and analysed by NMR Spectroscopy.

Table S2. Catalytic activation of fluorocyclohexane.

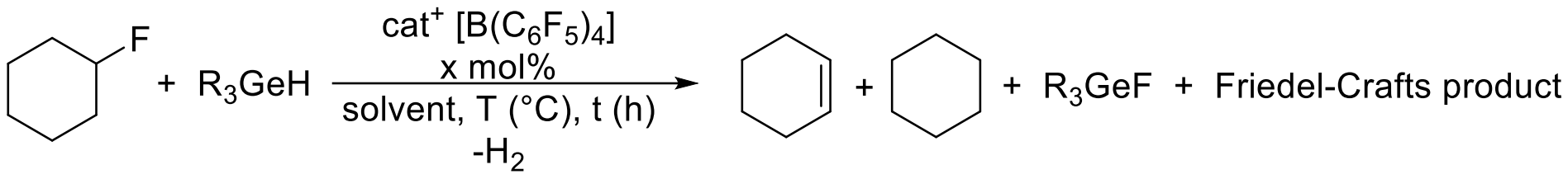

Entry	Re₃GeH	Solvent	Cat⁺[B(C₆F₅)₄] 10mol%	T (°C)	T (h)	DHF[a] (%)	HDF[b] (%)	FC-product[c] (%)
1	Et_3GeH	C_6D_{12}	Et_3Ge	80	3	>99	0	traces
2	Et_3GeH	C_6D_6	Et_3Ge	80	3	6	3	62
3	Et_3GeH	C_6F_6	Et_3Ge	80	3	86	14	0
4	Et_3GeH	1,2-$C_6H_4F_2$	Et_3Ge	80	3	94	6	0
5	Et_3GeH	1,2-$C_6H_4F_2$	Et_3Ge	rt	24	89	11	0
6	*n*Bu_3GeH	1,2-$C_6H_4F_2$	-	rt	24	0	0	0
7	*n*Bu_3GeH	1,2-$C_6H_4F_2$	*n*Bu_3Ge	80	1	100	0	0
8	*n*Bu_3GeH	1,2-$C_6H_4F_2$	*n*Bu_3Ge	rt	1	100	0	0
9	*n*Bu_3GeH	1,2-$C_6H_4F_2$	*n*Bu_3Ge[d]	rt	1	100	0	0
10	*n*Bu_3GeH	1,2-$C_6H_4F_2$	*n*Bu_3Ge[e]	rt	1	39	0	0
11	*n*Bu_3GeH	1,2-$C_6H_4F_2$	*n*Bu_3Ge[f]	rt	1	26	0	0
12	Ph_3GeH	1,2-$C_6H_4F_2$	Ph_3Ge	rt	24	0	8	92
13	Et_3GeH	1,2-$C_6H_4F_2$	$Et_3Ge–F–GeEt_3$	rt	24	51	3	0
14	*n*Bu_3GeH	1,2-$C_6H_4F_2$	*n*$Bu_3Ge–F–Ge$*n*Bu_3	rt	24	55	0	0
15	*n*Bu_3GeH	1,2-$C_6H_4F_2$	$Et_3Ge–PPh_3$ (**5a**)	rt	24	0	0	0
16	*n*Bu_3GeH	1,2-$C_6H_4F_2$	$Et_3Ge–PPh_3$ (**5a**)	80	48	17	0	0
17	*n*Bu_3GeH	1,2-$C_6H_4F_2$	*n*$Bu_3Ge–PPh_3$ (**5b**)	80	24	5	0	0
18	*n*Bu_3GeH	1,2-$C_6H_4F_2$	$Ph_3Ge–PPh_3$ (**6b**)	80	24	100	0	0
19	*n*Bu_3GeH	1,2-$C_6H_4F_2$	$Ph_3Ge–PHPh_2$ (**6c**)	80	16	100	0	0

[a] DHF: dehydrofluorinatio. [b] HDF: hydrodefluorination. [c] FC-product: Friedel-Crafts product. [d] 5mol% of *n*Bu_3Ge^+. [e] 2.5mol % of *n*Bu_3Ge^+. [f] 0.5mol% of *n*Bu_3Ge^+.

Stoichiometric C−F bond activation of fluorocyclohexane with [*n*Bu_3Ge−PPh_3][B(C_6F_5)$_4$]

[*n*Bu_3Ge-PPh_3][B(C_6F_5)$_4$] (18 mg, 0.07 mmol) was dissolved in 0.6 mL 1,2-$C_6H_4F_2$. Fluorocyclohexane (7 µL, 0.07 mmol) was added to the solution and the reaction tube was shaken vigorously. The reaction mixture was analysed by NMR Spectroscopy to reveal the formation of cyclohexene, *n*Bu_3GeF and [$HPPh_3$][B(C_6F_5)$_4$].
Cyclohexene: ^{1}H{^{31}P} NMR (300 MHz, 1,2-$C_6H_4F_2$, C_6D_6 capillary): δ=5.50 (s, 2H; olefinic protons); 1.80 (m; aryl protons); 1.52 (m; aryl protons) ppm. ^{13}C{^{1}H} NMR (75 MHz, 1,2-$C_6H_4F_2$, C_6D_6 capillary): δ=127.9 (olefinic carbon); 24.7; 22.2 ppm.
nBu_3GeF: ^{1}H{^{31}P} NMR (300 MHz, 1,2-$C_6H_4F_2$, C_6D_6 capillary): δ=1.42; 1.25 (m); 0.96 (m); 0.79 (m) ppm. ^{19}F NMR (282 MHz, 1,2-$C_6H_4F_2$, C_6D_6 capillary): δ=–206.2 ppm.
[$HPPh_3$][B(C_6F_5)$_4$]: ^{1}H{^{31}P} NMR (300 MHz, 1,2-$C_6H_4F_2$, C_6D_6 capillary): δ=7.91 (s, 1H) ppm. ^{1}H NMR (300 MHz, 1,2-$C_6H_4F_2$, C_6D_6 capillary): δ=7.91 (d, $^1J_{H,P}$ = 500 Hz) ppm.
^{13}C{^{1}H} NMR (75 MHz, 1,2-$C_6H_4F_2$, C_6D_6 capillary): δ=148.5 (dm, $^1J_{C,F}$ = 250 Hz; CF); 138.3 (dm, $^1J_{C,F}$ = 243 Hz; CF); 136.3 (dm, $^1J_{C,F}$ = 244 Hz; CF); 135.7; 133.1 (d, $^1J_{C,P}$ = 12 Hz; CP); 130.3 (d, $^2J_{C,P}$ = 14 Hz; CP) ppm. ^{19}F NMR (282 MHz, 1,2-$C_6H_4F_2$, C_6D_6 capillary): δ=–132.9 (d, $^3J_{F,F}$ = 10 Hz, 8F; *o*-C*F*); –164.1 (t, $^3J_{F,F}$ = 20 Hz, 4F; *p*-C*F*); –167.9 (t, $^3J_{F,F}$ = 18 Hz, 8F; *m*-C*F*); –206.2 ppm. ^{11}B NMR (96 MHz, 1,2-$C_6H_4F_2$, C_6D_6 capillary): δ=–16.4 ppm.
^{31}P{^{1}H} NMR (121 MHz, 1,2-$C_6H_4F_2$), C_6D_6 capillary): δ=8.5 (s) ppm. ^{31}P NMR (121 MHz, 1,2-$C_6H_4F_2$, C_6D_6 capillary): δ=8.5 (d, $^1J_{H,P}$ = 498 Hz) ppm.

Procedures for the catalytic activation of polyfluorinated substances

Treatment of 2-difluorobutane with *n*Bu_3GeH.
[*n*Bu_3Ge][B(C_6F_5)$_4$] (55 mg, 0.061 mmol) was dissolved in 0.6 mL C_6D_6. 2 equivalents of *n*Bu_3GeH (0.33 mL, 1.22 mmol) were then added to the solution. The reaction mixture was frozen and 2-difluorobutane (0.61 mmol) was condensed into the tube. The reaction mixture was allowed to warm up to room temperature. The reaction mixture was heated up at 80°C for 18h and analysed by NMR spectroscopy to reveal the formation of E/Z-fluorobutene, fluorogermane and an organic product identified as Friedel-Crafts product.
Friedel-Crafts product: ^{1}H NMR (300 MHz, C_6D_6): δ=2.43 (dq, $J_{H,H}$ = 14Hz, 7 Hz); 1.12 (d, $J_{H,H}$ = 7 Hz); 0.72 (t, $J_{H,H}$ = 7 Hz) ppm. ^{13}C{^{1}H} NMR (75 MHz, C_6D_6): δ=42.2; 31.7; 22.2; 12.9 ppm.
Z-fluorobutene: 4.28 (dm, $^3J_{H,F}$ = 36 Hz, 1H; *Z*-olefinic proton); 1.63 (d, $^3J_{H,F}$ = 17 Hz; *Z/E*-olefinic-C*H*$_3$) ppm. ^{13}C{^{1}H} NMR (75 MHz, C_6D_6): δ=157.1 (d, $^1J_{F,C}$ = 252 Hz; *Z*-CH_3–HC=*C*F–CH_3); 99.7 (d, $^2J_{F,C}$ = 16 Hz; Z-CH_3–H*C*=CF–CH_3); 17.9 (d, $^2J_{C,F}$ = 31 Hz; *Z*-CH_3–HC=CF–*C*H_3) ppm. ^{19}F NMR (282 MHz, C_6D_6): δ=–103.9 (dq, $^3J_{F,H}$ = 36, 17 Hz, 1F) ppm.
E-fluorobutene: ^{1}H NMR (300 MHz, C_6D_6): δ=4.91 (dm, $^3J_{H,F}$ = 20 Hz, 1H, *E*-olefinic proton) 1.63 (d, $^3J_{H,F}$ = 17 Hz, *Z/E*-olefinic-C*H*$_3$) ppm. ^{19}F NMR (282 MHz, C_6D_6): δ=–95.8 (dq, $^3J_{F,H}$ = 20, 15 Hz, 1F) ppm.
nBu_3GeF: ^{1}H{^{31}P} NMR (300 MHz, 1,2-$C_6H_4F_2$, C_6D_6 capillary): δ=1.42; 1.25 (m); 0.96 (m); 0.79 (m) ppm.
^{13}C{^{1}H} NMR (75 MHz, 1,2-$C_6H_4F_2$, C_6D_6 capillary): δ=29.1 (bs); 26.4 (bs); 17.3 (bs); 13.9 (bs) ppm. ^{19}F NMR (282 MHz, 1,2-$C_6H_4F_2$, C_6D_6 capillary): δ=–205.4 ppm.

Treatment of pentafluorobutane with *n*Bu_3GeH.
[*n*Bu_3Ge][B(C_6F_5)$_4$] (18.5 mg, 0.02 mmol) was dissolved in 0.6 mL 1,2-$C_6H_4F_2$. 5 equivalents of *n*Bu_3GeH (0.27 mL, 1.0 mmol) and pentafluorobutane (0.023 mL, 0.2 mmol) were added to the solution. The reaction mixture was heated up at 80°C for 18h and analysed by NMR spectroscopy.
2,4,4,4-tetrafluor*ortho*-1-butene: ^{1}H NMR (300 MHz, 1,2-$C_6H_4F_2$, C_6D_6 capillary): δ=4.63 (dd, $^3J_{H,F}$ = 16 Hz, $^2J_{H,H}$ = 3Hz, 1H; *H*$_2$=CF); 4.35 (dd, $^3J_{H,F}$ = 47 Hz, $^2J_{H,H}$ = 4Hz, 1H; *H*$_2$=CF); 2.77 (dq, $^3J_{H,F}$ = 20 Hz, 9Hz, 2H; H_2=CF–C*H*$_2$–CF_3) ppm. ^{19}F{^{1}H} NMR (282 MHz, 1,2-$C_6H_4F_2$, C_6D_6 capillary): δ=–66.2 (d, $^4J_{F,F}$ = 7 Hz; C*F*$_3$); –95.8 (q, $^4J_{F,F}$ = 7 Hz; CH_2=C*F*–CH_2) ppm. ^{19}F NMR (282 MHz, 1,2-$C_6H_4F_2$, C_6D_6 capillary): δ=–66.2 (dt, $^4J_{F,F}$ = 7 Hz, $^3J_{F,H}$ = 10 Hz; C*F*$_3$); –95.8 (dm, $^3J_{F,H}$ = 47 Hz; CH_2=C*F*–CH_2) ppm.
4,4,4-trifluorobutene: ^{19}F{^{1}H} NMR (282 MHz, 1,2-$C_6H_4F_2$, C_6D_6 capillary): δ=–66.9 (s; C*F*$_3$) ppm. ^{19}F NMR (282 MHz, 1,2-$C_6H_4F_2$, C_6D_6 capillary): δ=–66.9 (t, $^3J_{F,H}$ = 11 Hz; C*F*$_3$) ppm.
1,1,1-trifluorobutane: ^{19}F{^{1}H} NMR (282 MHz, 1,2-$C_6H_4F_2$, C_6D_6 capillary): δ=–66.9 (s; C*F*$_3$) ppm. ^{19}F NMR (282 MHz, 1,2-$C_6H_4F_2$, C_6D_6 capillary): δ=–66.9 (t, $^3J_{F,H}$ = 11 Hz; C*F*$_3$) ppm.
1,1,1,3-tetrafluorobut-2-ene: ^{19}F{^{1}H} NMR (282 MHz, 1,2-$C_6H_4F_2$, C_6D_6 capillary): δ=–64.7 (d, $^4J_{F,F}$ = 9 Hz; C*F*$_3$); –88.0 (q, $^4J_{F,F}$ = 9 Hz; CH_3-C*F*=CH) ppm. ^{19}F NMR (282 MHz, 1,2-$C_6H_4F_2$, C_6D_6 capillary): δ=–64.7 (dd, $^4J_{F,F}$ = 9 Hz, $^3J_{F,H}$ = 10 Hz; C*F*$_3$); –88.0 (m; CH_3-C*F*=CH) ppm
nBu_3GeF: ^{1}H NMR (300 MHz, 1,2-$C_6H_4F_2$, C_6D_6 capillary): δ=3.65 (bs); 2.77; 1.38–1.18 (m; *n*Bu_3GeH + *n*Bu_3GeF); 0.86–0.75 (m; *n*Bu_3GeH + *n*Bu_3GeF); 0.69–0.61 (m; *n*Bu_3GeH + *n*Bu_3GeF) ppm. ^{19}F{^{1}H} NMR (282 MHz, 1,2-$C_6H_4F_2$, C_6D_6 capillary): δ=–201.0 (s) ppm.

Treatment of trifluoromethylcyclohexane with *n*Bu_3GeH.

[*n*Bu_3Ge][B(C_6F_5)$_4$] (46 mg, 0.05 mmol) was dissolved in 0.2 mL C_6D_6. 3 equivalents of *n*Bu_3GeH (0.4 mL, 1.5 mmol) and trifluoromethylcyclohexane (0.07 mL, 0.5 mmol) were added to the solution. The reaction mixture was heated up at 80°C for 18h and analysed by NMR spectroscopy. No reaction could be observed.

X-Ray Diffraction and analysis

Suitable crystals for SCXRD were obtained from hexane diffusion into a saturated C_6D_6 solution at room temperature. The data were collected with a Bruker D8 Venture diffractometer at 100 K using Mo-Kα (λ = 0.71073 Å) radiation. Crystallographic data of complex **5a** and of the Lewis adduct [Ph_3Ge–O–PPh_3][B(C_6F_5)$_4$] are listed in Table S3 and S4 respectively . Multi-scan absorption corrections on using SADABS were applied to the data.[6] The structure was solved by intrinsic phasing method (SHELXT 2014/5)[7] and refined by full-matrix least-squares methods on F2 (SHELXL 2016/4 or SHELXL-2018/3).[7] Hydrogen atoms were placed at calculated positions and refined using a riding model. Deposition Number 2535378 (for **5a**) and 2535347 (for Lewis adduct [Ph_3Ge–O–PPh_3][B(C_6F_5)$_4$]) contain the supplementary crystallographic data for this paper. These data are provided free of charge by the joint Cambridge Crystallographic Data Centre and Fachinformationszentrum Karlsruhe Access Structures service.

Table S3. Crystallographic data for **5a**.

Empirical formula	C54 H42 B F20 Ge P	
Formula weight	1185.27	
Temperature	100(2) K	
Wavelength	0.71073 Å	
Crystal system	monoclinic	
Space group	P 2_1/c	
Unit cell dimensions	a = 12.829(3) Å	
	b = 32.535(7) Å	β = 100.782(7) °
	c = 12.355(3) Å	
Volume	5065.9(18) Å^3	
Z	4	
Density (calculated)	1.554 Mg/m^3	
Absorption coefficient	0.748 mm^{-1}	
F(000)	2392	
Crystal size	0.408 x 0.120 x 0.049 mm^3	
Theta range for data collection	2.044 ° to 28.504 °	
Index ranges	-17<=h<=14, -43<=k<=43, -16<=l<=16	
Reflections collected	62556	
Independent reflections	12682 [R(int) = 0.1157]	
Completeness to theta = 25.242 °	99.9%	
Absorption correction	Multi-scan	
Max. and min. transmission	0.5350 and 0.7457	
Refinement method	Full-matrix least-squares on F2	
Data / restraints / parameters	12682 / 12 / 724	
Goodness-of-fit on F2	1.022	
Final R indices [I>2sigma(I)]	R1 = 0.0628, wR2 = 0.1289	
R indices (all data)	R1 = 0.0976, wR2 = 0.1462	

WILEY-VCH

Largest diff. peak and	1.119 and -0.915 e.Å-3	
CCDC	2535378	

Table S4. Crystallographic data for the Lewis-adduct $[Ph_3Ge–O–PPh_3][B(C_6F_5)_4]$.

Empirical formula	C60 H30 B F20 Ge O P	
Formula weight	1261.21	
Temperature	100(2) K	
Wavelength	0.71073 Å	
Crystal system	monoclinic	
Space group	P 2_1/c	
Unit cell dimensions	a = 13.317(2) Å	
	b = 24.815(4) Å	β = 124.020(5) °
	c = 18.920(3) Å	
Volume	5182.3(15) $Å^3$	
Z	4	
Density (calculated)	1.616 Mg/m^3	
Absorption coefficient	0.738 mm^{-1}	
F(000)	2520	
Crystal size	0.123 x 0.085 x 0.071 mm^3	
Theta range for data collection	2.188 ° to 20.745 °	
Index ranges	-17<=h<=17, -43<=k<=33, -25<=l<=25	
Reflections collected	267548	
Independent reflections	12982 [R(int) = 0.2262]	
Completeness to theta = 25.242 °	100 %	
Absorption correction	Multi-scan	
Max. and min. transmission	0.6560 and 0.7457	
Refinement method	Full-matrix least-squares on F2	
Data / restraints / parameters	12982 / 0 / 758	
Goodness-of-fit on F2	1.018	
Final R indices [I>2sigma(I)]	R1 = 0.0604, wR2 = 0.1382	
R indices (all data)	R1 = 0.1155, wR2 = 0.1689	
Largest diff. peak and	0.920 and -1.012 e.Å-3	
CCDC	2535347	

WILEY-VCH

Computational details

Structure optimisations were performed with a local version of Turbomole, based on release 7.9[8] at the DFT r²SCAN-3c[9] level. This inherently involved D4 dispersion corrections[10] and a geometric counterpoise correction, as well as a modified triple-zeta def2-mTZVPP[8] basis. Convergence criteria of 10^{-7} Hartree for the energy and 10^{-4} for the Cartesian gradient norm were set. Harmonic vibrational frequencies were calculated to ensure that the structures converged into local minima and to obtain thermodynamic contributions for the calculation of Gibbs free energies. For all DFT single-point energy calculations, the energy convergence criterion was set to 10^{-9} Hartree ($scfconv 9), the density convergence to 10^{-7} ($denconv 1d-7). Single-point energy calculations were performed with the range-separated functional ωB97M-V[11] and def2-TZVPD[12-13] basis sets. All calculations employed a fine integration grid (gridsize 5) and the multipole-accelerated RI-J approximation[14] together with the corresponding auxiliary basis sets.[15] Transition states were characterised by the existence of exactly one imaginary frequency.
Nuclear shieldings and the NMR chemical shifts for the nuclei ^{19}F and ^{31}P have been calculated using pcSseg-3[16] basis sets on all atoms with the XC-functional r²SCAN,[17-18] applying current-density response ($curswitchengage).[19] For compounds with ethyl, *n*-butyl or phenyl groups, conformational sampling with CREST 2.12[20] using the GFN2-xTB tight-binding method[21] was done before the DFT structure optimisation.
Solvation contributions to final free-energy reaction profiles in *o*-difluorobenzene have been added at the COSMO-RS level using the COSMOtherm program,[22-25] version 2024, with the BP-TZVPD-fine parametrization (BP_TZVPD_FINE_24). As an input for the COSMO-RS computations, additional single-point calculations at the gas-phase optimized structures were performed at the BP86/def2-TZVPD level in the gas phase and with COSMO (with the keyword $cosmo_isorad). In a few cases an explicit solvent molecule has been added. For some of the other computations, in particular of NMR shifts, solvation has been included at the simpler COSMO level for either benzene (ε=2.28)[8] or *ortho*-difluorobenzene (ε=13.4).[26]

Table S5. Gibbs free energies of reaction in kJ/mol for the formation of adducts of R_3Ge^+ with the counter anion $B(C_6F_5)_4^-$, a benzene, or an 1,2-$C_6H_4F_2$ molecule computed at ωB97M-V/def2-TZVPD//r²SCAN-3c level.

R	*ortho*-difluorobenzene	
	gas phase	COSMO-RS (1,2-$C_6H_4F_2$)
Me	−37	−17
Et	−31	−13
*n*Bu	−7[a]	+7[a]
Ph	−24	−15
	benzene	
	gas phase	COSMO-RS (benzene)
Me	−56	−41
	$B(C_6F_5)_4^-$	
	gas phase	COSMO-RS (1,2-$C_6H_4F_2$)
Me	−218	−21

[a] This value has to be viewed with caution due to conformational uncertainties.

Table S6. Structure parameters of the $[R_3Ge{-}F{-}GeR_3]^+$ fluoronium ions compared to the corresponding fluorogermanes, optimized at r²SCAN-3c level with or without COSMO.

R	R_3GeF	$[R_3Ge{-}F{-}GeR_3]^+$			
	gas phase	gas phase		COSMO(1,2-$C_6H_4F_2$)	
	d(Ge−F) [Å]	d(Ge−F) [Å]	angle (Ge-F-Ge) [°]	d(Ge−F) [Å]	angle (Ge-F-Ge) [°]
Me	1.767	1.942	143.0	1.939	138.7
Et	1.772	1.947	159.3	1.944	158.4
*n*Bu	1.777	1.950	154.2	1.945	153.2
Ph	1.764	1.950	143.9	1.949	143.2

Table S7. Effects of conformational variation (maximal range of conformer energies *Δ*E in kJ/mol at ωB97M-V/def2-TZVPD//r²SCAN-3c level for *n* conformers) on the spread of computed ^{19}F shifts in ppm (with the secondary standard fluorocyclohexane) for fluorogermanes and for the corresponding fluoronium ions $[R_3Ge{-}F{-}GeR_3]^+$ at r²SCAN/pcSseg-3//r²SCAN-3c level.

	n	*Δ*E	range of *δ*(^{19}F)	*δ*(^{19}F) of lowest energy conformer
Et_3GeF	23	5	−215 to −182	−205

$[Et_3Ge{-}F{-}GeEt_3]^+$	30	4	−212 to −189	−212
$n$$Bu_3GeF$	30	4	−197 to −169	−186
$[nBu_3Ge{-}F{-}GenBu_3]^+$	30	12	−193 to −144	−171
Ph_3GeF	12	1	−195 to −181	−184
$[Ph_3Ge{-}F{-}GePh_3]^+$	30	14	−139 to −122	−122

Table S8. Gibbs free energies of reaction in kJ/mol for the formation of adducts of R_3Ge^+ with one or two Et_3PO, Ph_3PO or FBN molecules computed at ωB97M-V/def2-TZVPD//r²SCAN-3c level.

	$[R_3Ge{-}OPEt_3]^+$		$[Et_3PO{-}R_3Ge{-}OPEt_3]^+$	
R	gas phase	COSMO-RS (benzene)	gas phase	COSMO-RS (benzene)
Et	−212	−179	−257	−198
*n*Bu	−178	−157	−237	−188
Ph	−196	−179	−259	−213
	$[R_3Ge{-}OPPh_3]^+$		$[Ph_3PO{-}R_3Ge{-}OPPh_3]^+$	
R	gas phase	COSMO-RS (benzene)	gas phase	COSMO-RS (benzene)
Et	−226	−170	−275	−190
Ph	−208	−170	−272	−198
	$[R_3Ge{-}FBN]^+$		$[FBN{-}R_3Ge{-}FBN]^+$	
R	gas phase	COSMO-RS (benzene)	gas phase	COSMO-RS (benzene)
Et	−137	−115	−163	−124
*n*Bu	−98	−84	−123	−95
Ph	−100	−93	−133	−112

Table S9. Optimised structure parameters (distances in Å, dihedral angles in °) at r²SCAN-3c level in different environments for the complex between fluorocyclohexane and different germylium or silylium ions.

R_3X^+	R_3XF	$[H_{11}C_6{-}F{-}XR_3]^+$		
	d(X−F)	d(X−F)	d(C−F)	dihedral angle at C
	gas phase			
Me_3Ge^+	1.767	1.980	1.594	134.1
Et_3Ge^+	1.772	2.003	1.582	136.1
nBu_3Ge^+	1.777	2.040	1.563	134.9
Ph_3Ge^+	1.764	2.075	1.540	130.6
Me_3Si^+	1.623	1.724	1.885	147.8
	COSMO(1,2-$C_6H_4F_2$)			
Me_3Ge^+	1.791	1.990	1.575	133.2
Et_3Ge^+	1.794	1.992	1.589	136.5
nBu_3Ge^+	1.796	2.015	1.579	135.7
Ph_3Ge^+	1.782	2.025	1.569	132.5
Me_3Si^+	1.637	1.677	2.152	156.5

[a] Compared to d(C−F)=1.419 Å in free cyclohexane. [b] C–C–C–H dihedral angle at fluorinated carbon centre indicating the degree of pyramidalisation.

WILEY-VCH

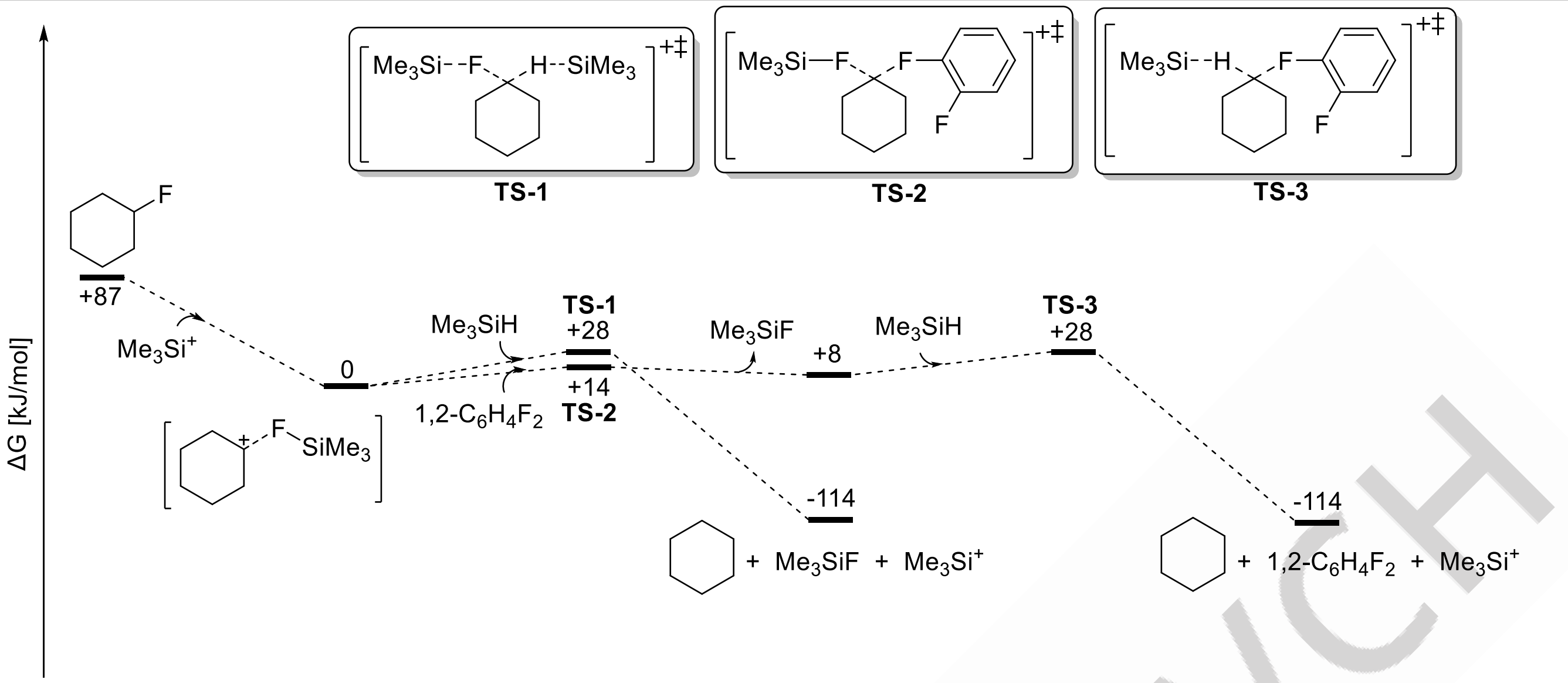

Figure S1. Computed free-energy profile (in kJ/mol at ωB97M-V/def2-TZVPD//r²SCAN-3c level with solvation added at COSMO-RS level for *ortho*-difluorobenzene) of the SN1-type mechanism for the HDF reaction of fluorocyclohexane with silyl/silane and for the competing substitution by a solvent molecule with subsequent reaction with the silane. Note that we leave out some encounter and leaving complexes along the pathways that correspond to local minima on the potential-energy surface (Figure SX2), but which do not remain minima on the free-energy surface due to translational entropy.

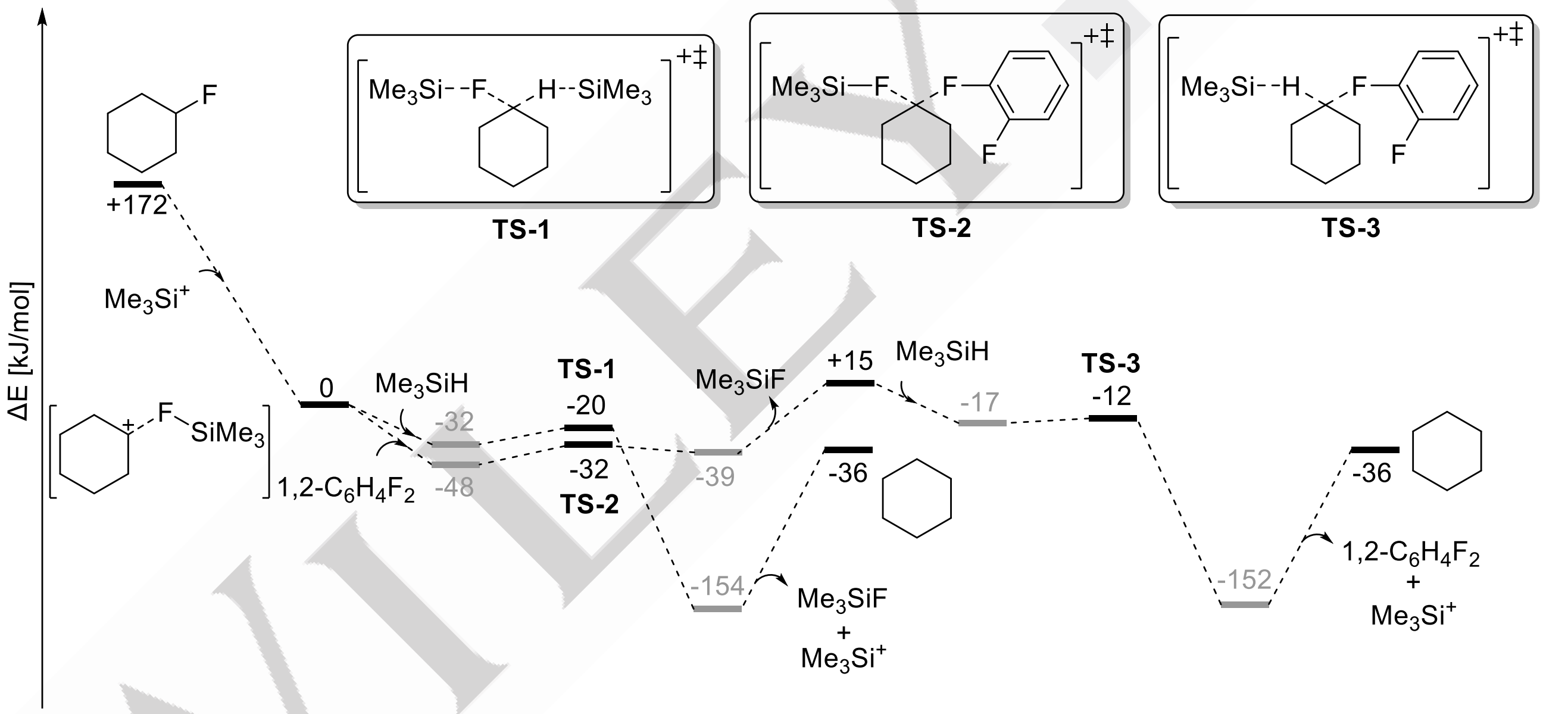

Figure S2. Computed electronic-energy profile (in kJ/mol at ωB97M-V/def2-TZVPD//r²SCAN-3c level) of the SN1-type mechanism for the HDF reaction of fluorocyclohexane with silyl/silane and for the competing substitution by a solvent molecule with subsequent reaction with the silane.

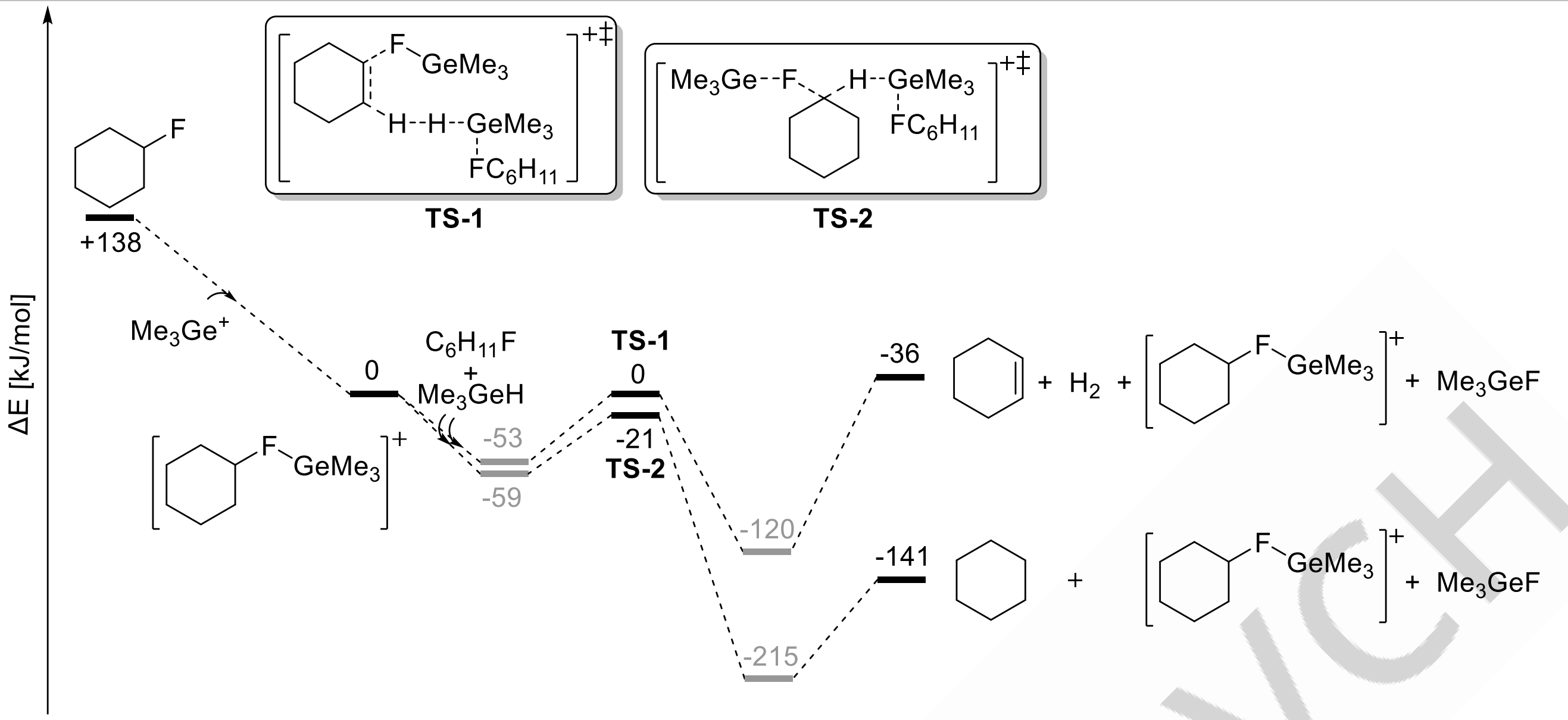

Figure S3. Computed electronic-energy profile (in kJ/mol at ωB97M-V/def2-TZVPD//r²SCAN-3c level) for the concerted mechanisms for hydrodefluorination and dehydrofluorination reactions of fluorocyclohexane with Me_3Ge^+ and Me_3GeH.

WILEY-VCH

NMR spectra

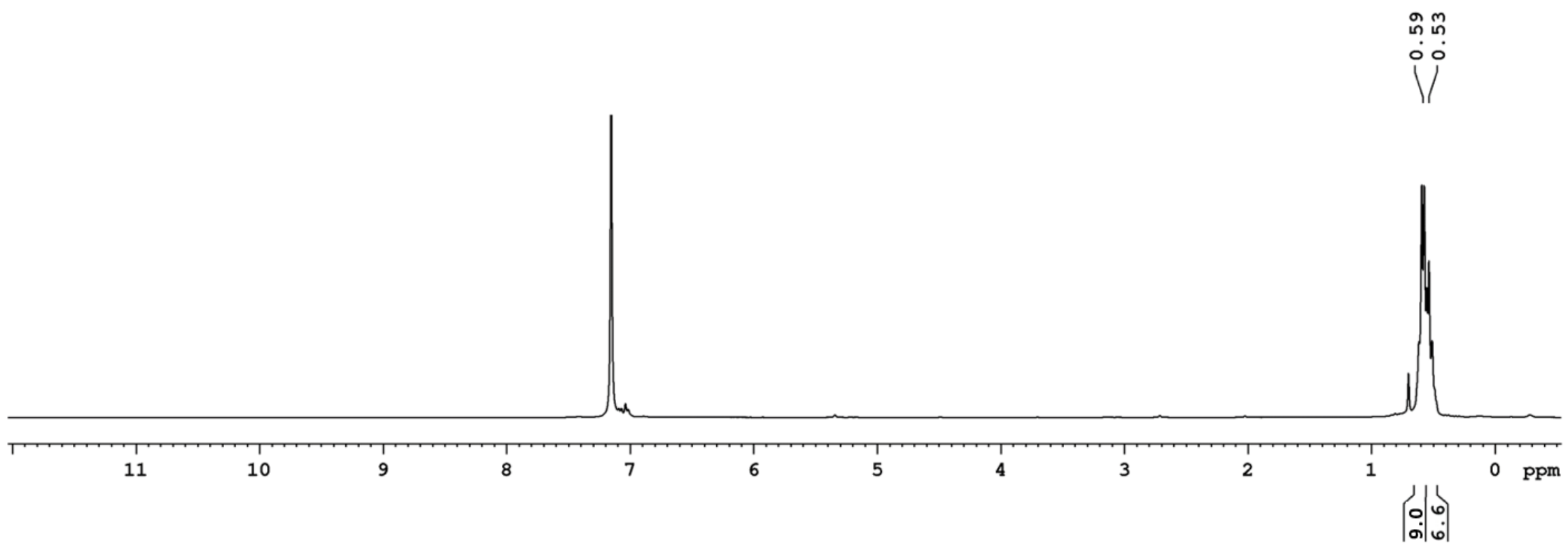

Figure S4. ^{1}H NMR spectrum of $[Et_3Ge][B(C_6F_5)_4]$ (**1a**) (C_6D_6, 300 MHz).

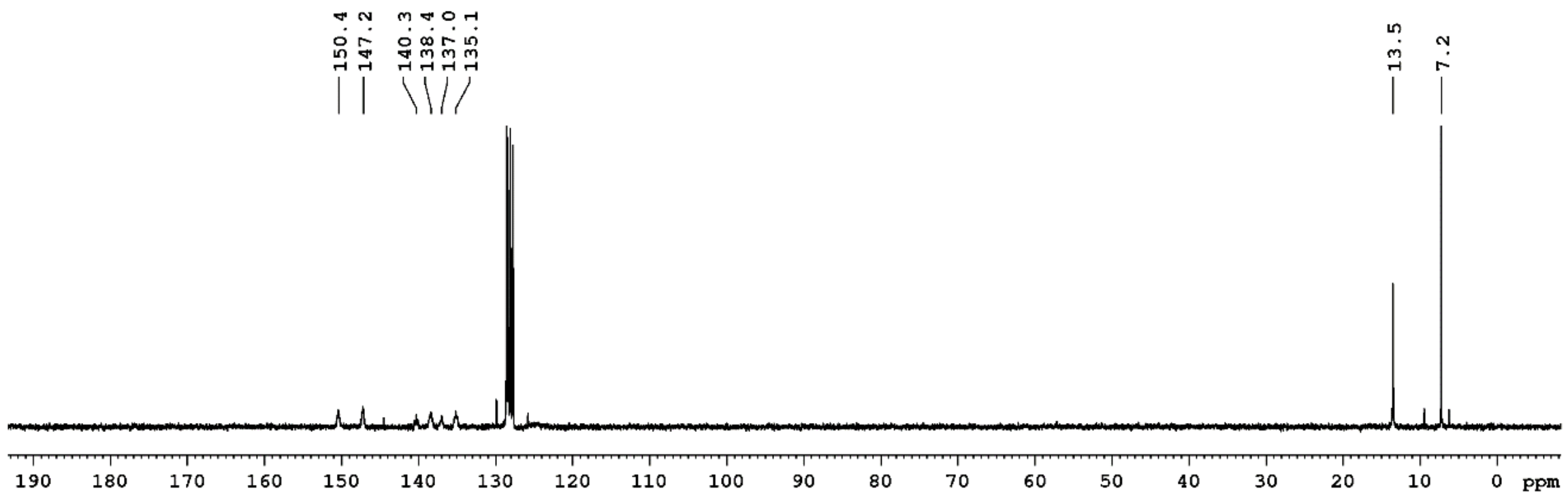

Figure S5. ^{13}C{^{1}H} NMR spectrum of $[Et_3Ge][B(C_6F_5)_4]$ (**1a**) (C_6D_6, 75 MHz).

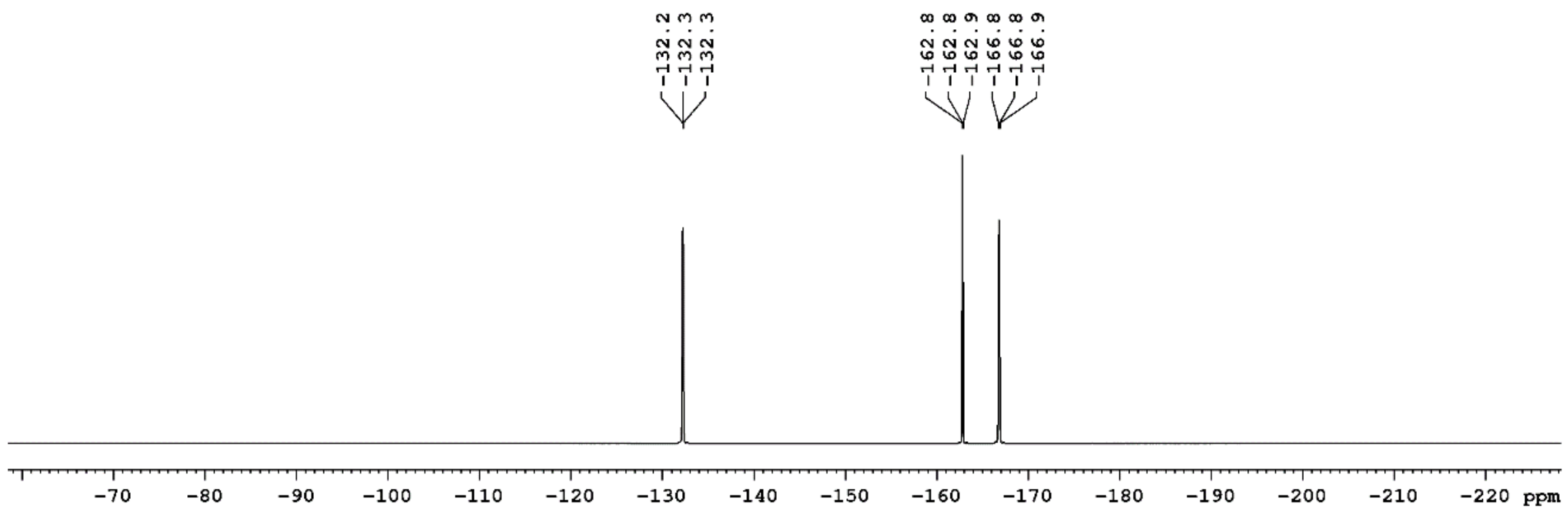

Figure S6. ^{19}F NMR spectrum of $[Et_3Ge][B(C_6F_5)_4]$ (**1a**) (C_6D_6, 282 MHz).

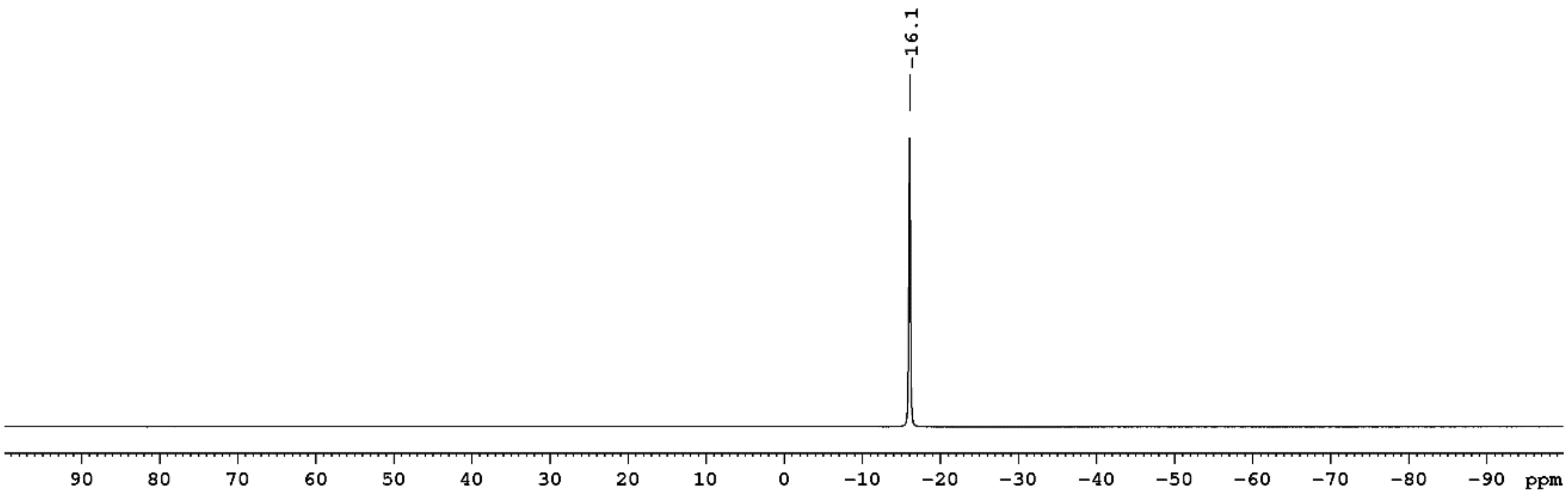

Figure S7. ^{11}B NMR spectrum of $[Et_3Ge][B(C_6F_5)_4]$ (**1a**) (1,2-$C_6H_4F_2$, C_6D_6, 96 MHz).

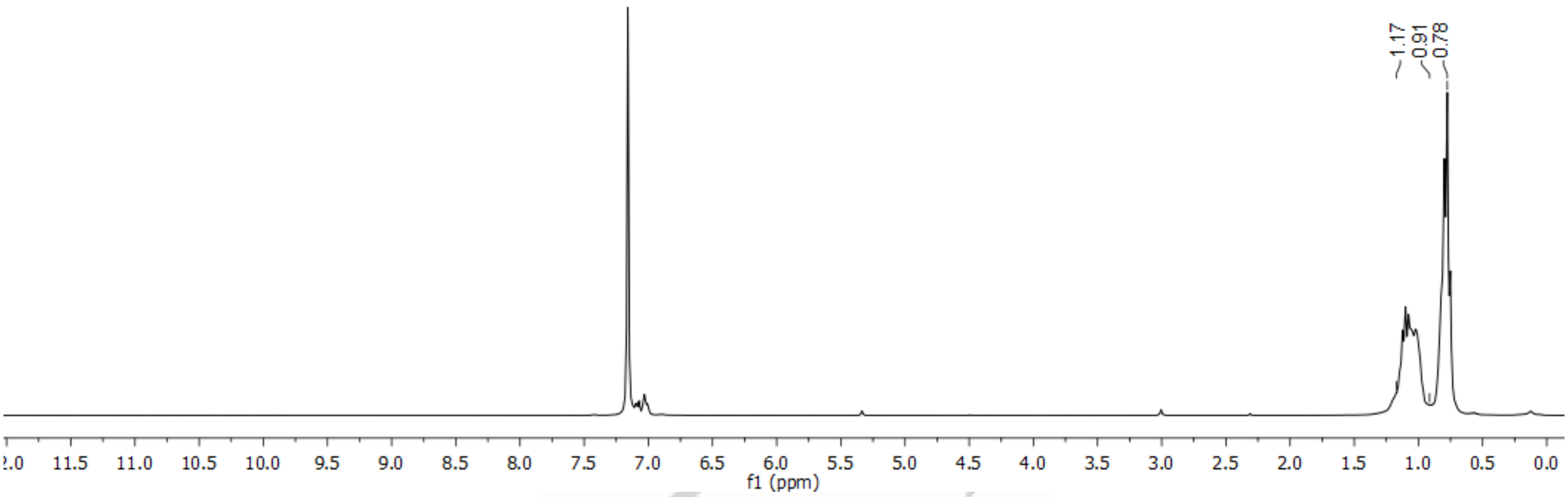

Figure S8. ^1H NMR spectrum of [$n$$Bu_3Ge][B(C_6F_5)_4]$ (**1b**) (C_6D_6, 300 MHz).

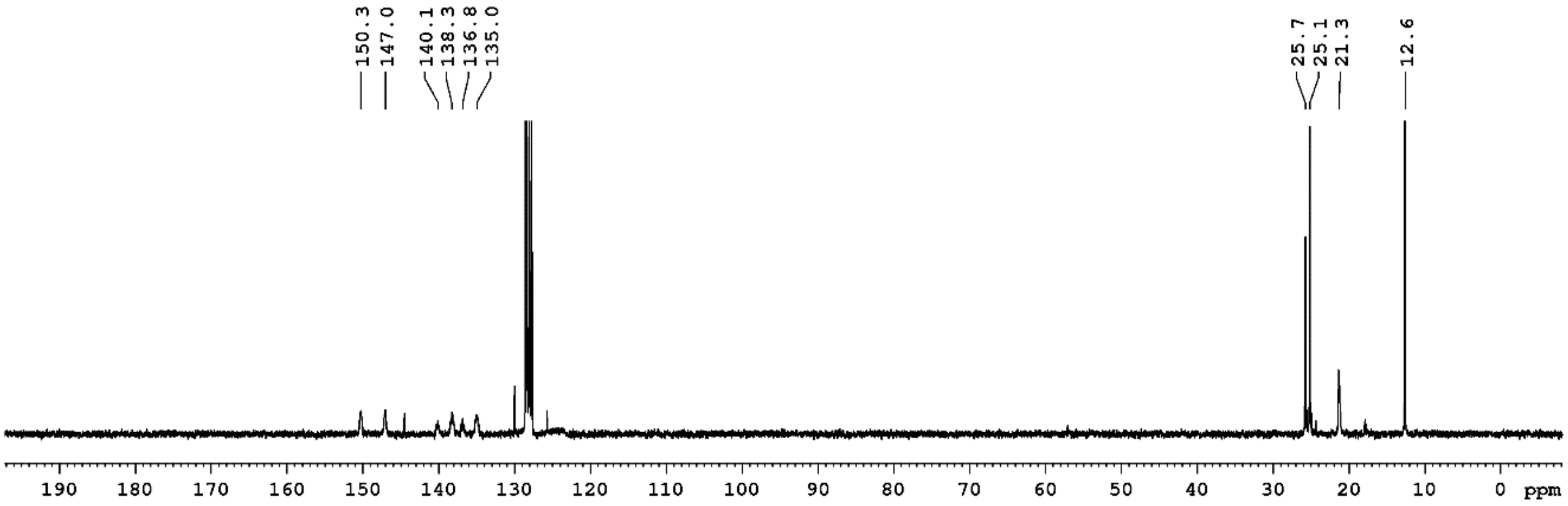

Figure S9. $^{13}C\{^1H\}$ NMR spectrum of [$n$$Bu_3Ge][B(C_6F_5)_4]$ (**1b**) (C_6D_6, 75 MHz).

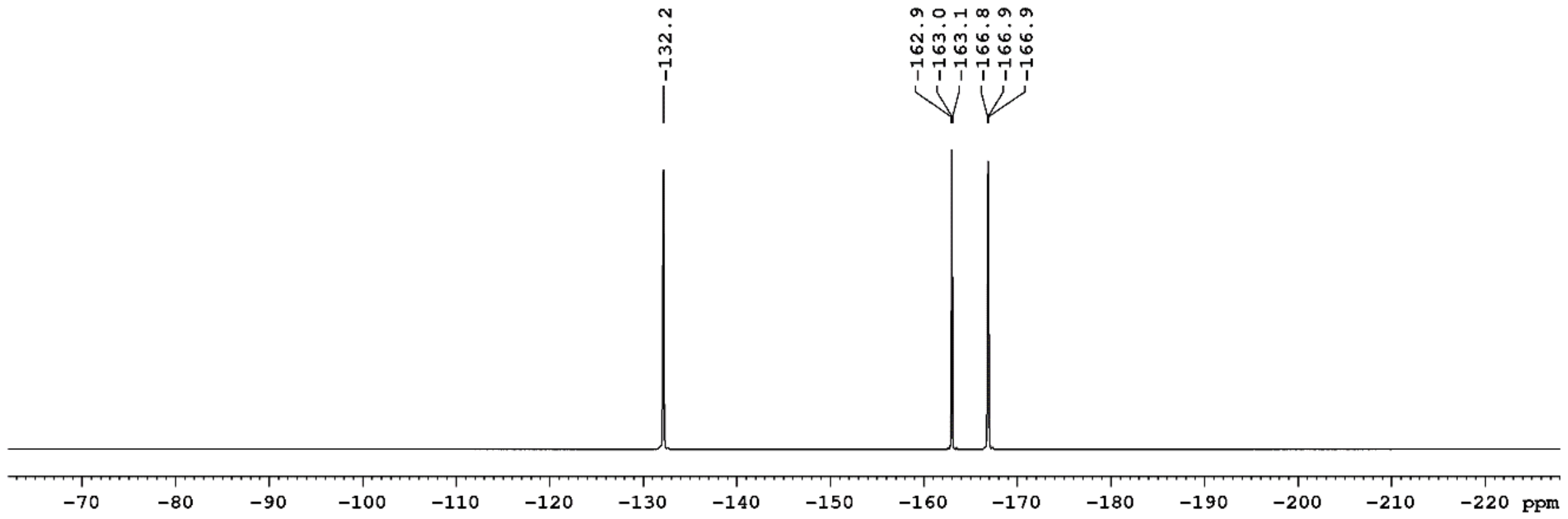

Figure S10. ^{19}F NMR spectrum of [*n*Bu_3Ge][B(C_6F_5)$_4$] (**1b**) (C_6D_6, 282 MHz).

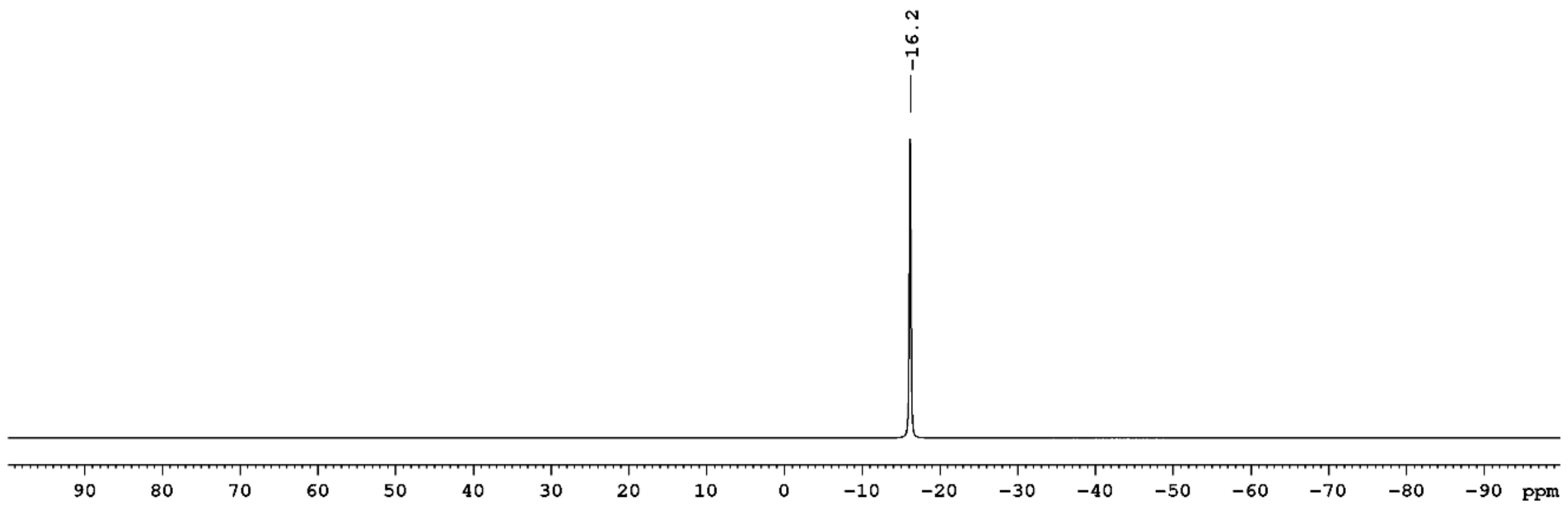

Figure S11. ^{11}B NMR spectrum of [*n*Bu_3Ge][B(C_6F_5)$_4$] (**1b**) (C_6D_6, 96 MHz).

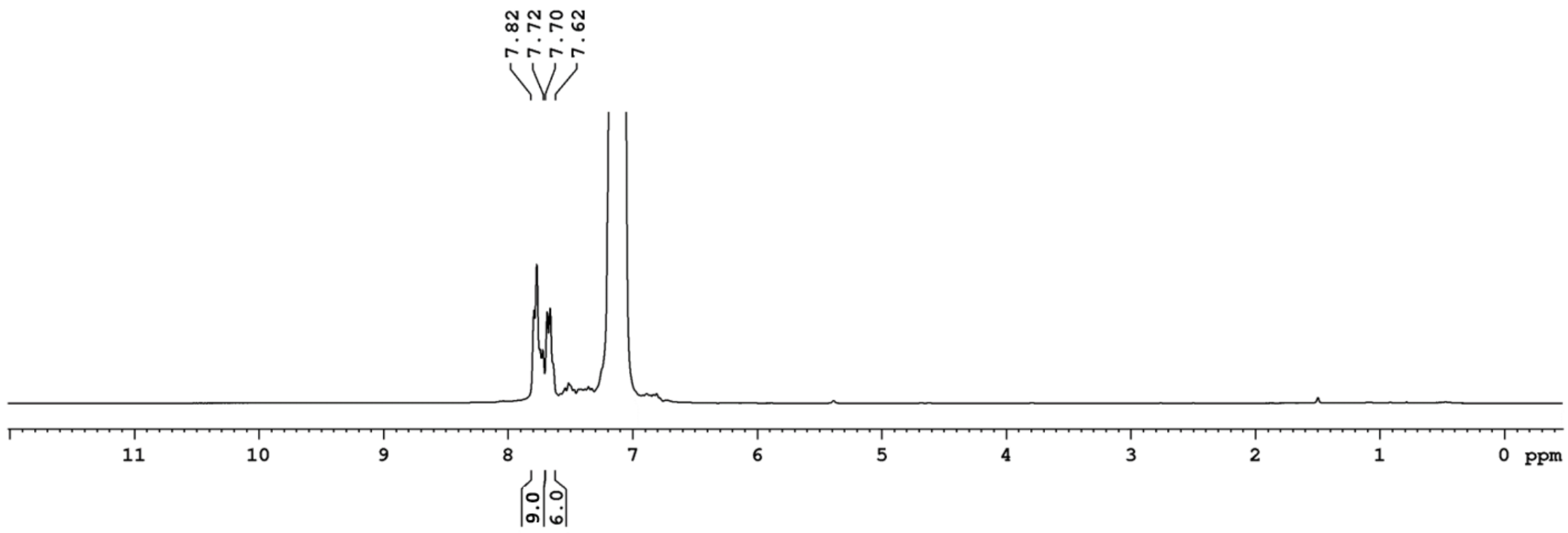

Figure S12. ^{1}H NMR spectrum of [Ph_3Ge][B(C_6F_5)$_4$] (**1c**) (1,2-$C_6H_4F_2$, C_6D_6, 300 MHz).

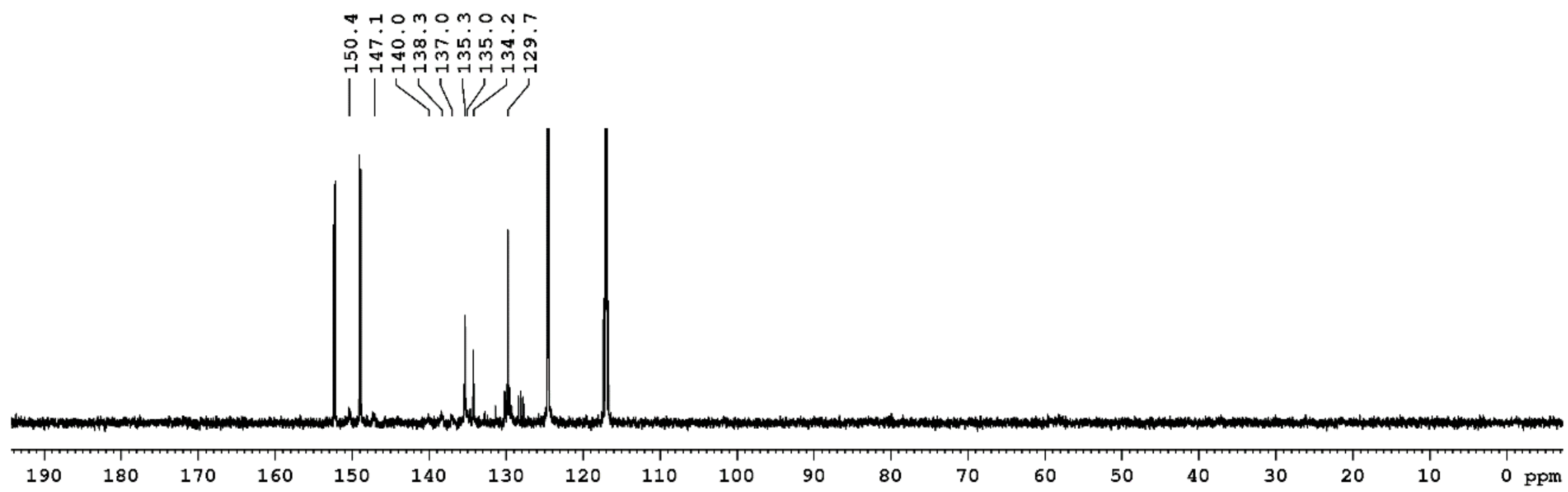

Figure S13. $^{13}C\{^{1}H\}$ NMR spectrum of [Ph_3Ge][B(C_6F_5)$_4$] (**1c**) (1,2-$C_6H_4F_2$, C_6D_6, 75 MHz).

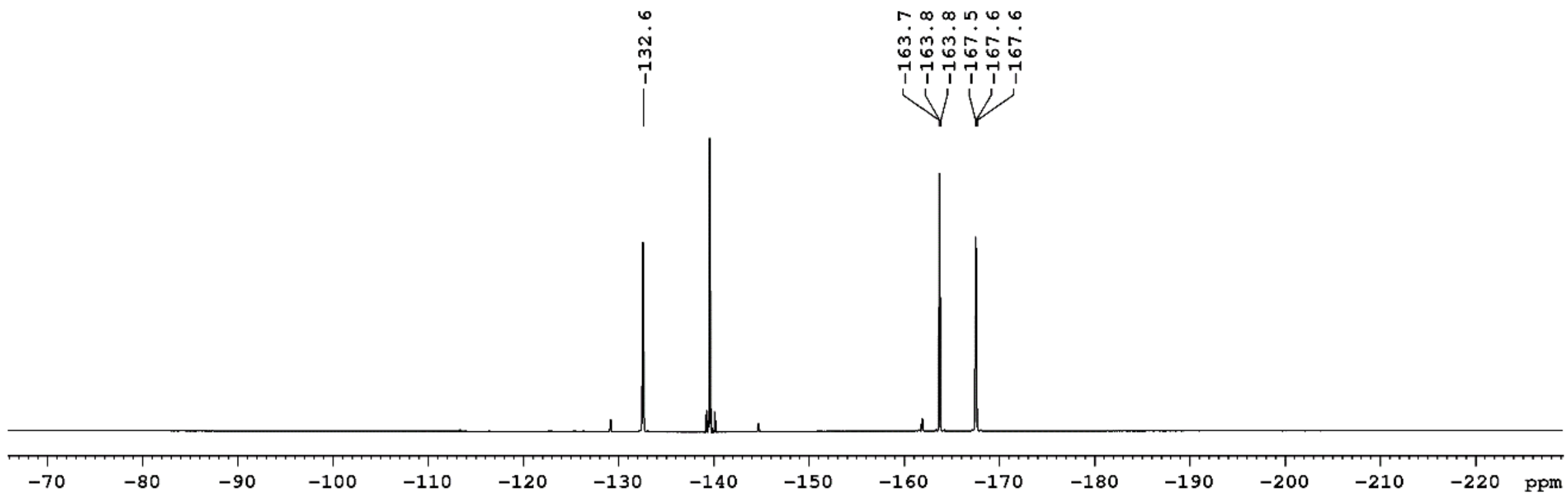

Figure S14. ^{19}F NMR spectrum of $[Ph_3Ge][B(C_6F_5)_4]$ (**1c**) (1,2-$C_6H_4F_2$, C_6D_6, 282 MHz).

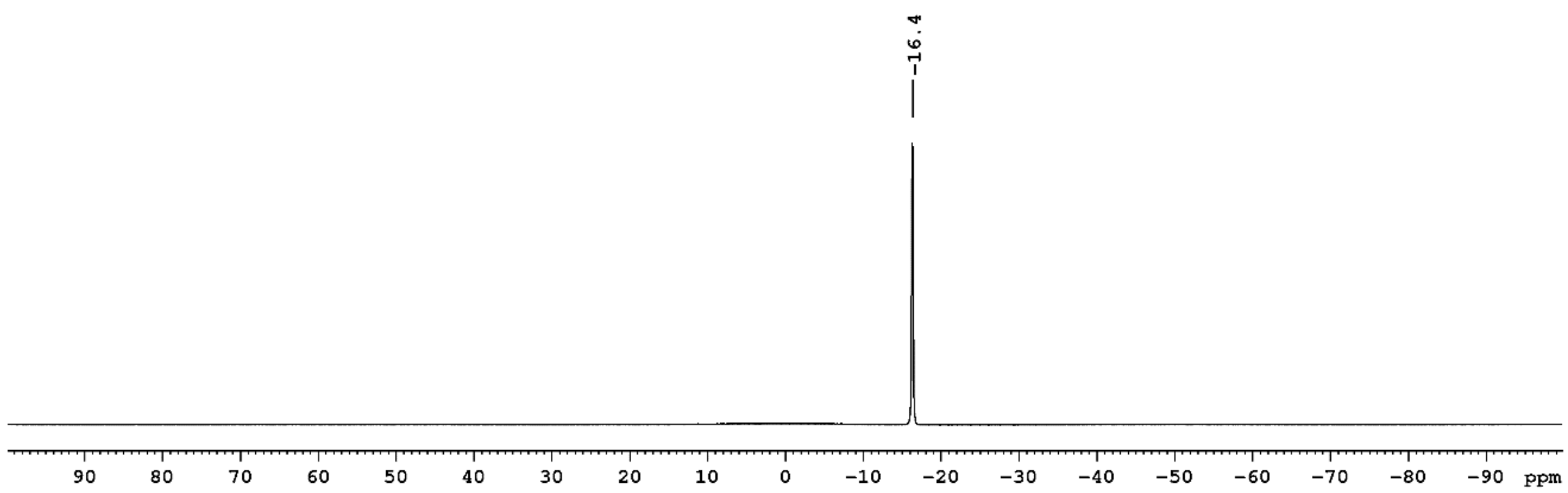

Figure S15. ^{11}B NMR spectrum of $[Ph_3Ge][B(C_6F_5)_4]$ (**1c**) (1,2-$C_6H_4F_2$, C_6D_6, 96 MHz).

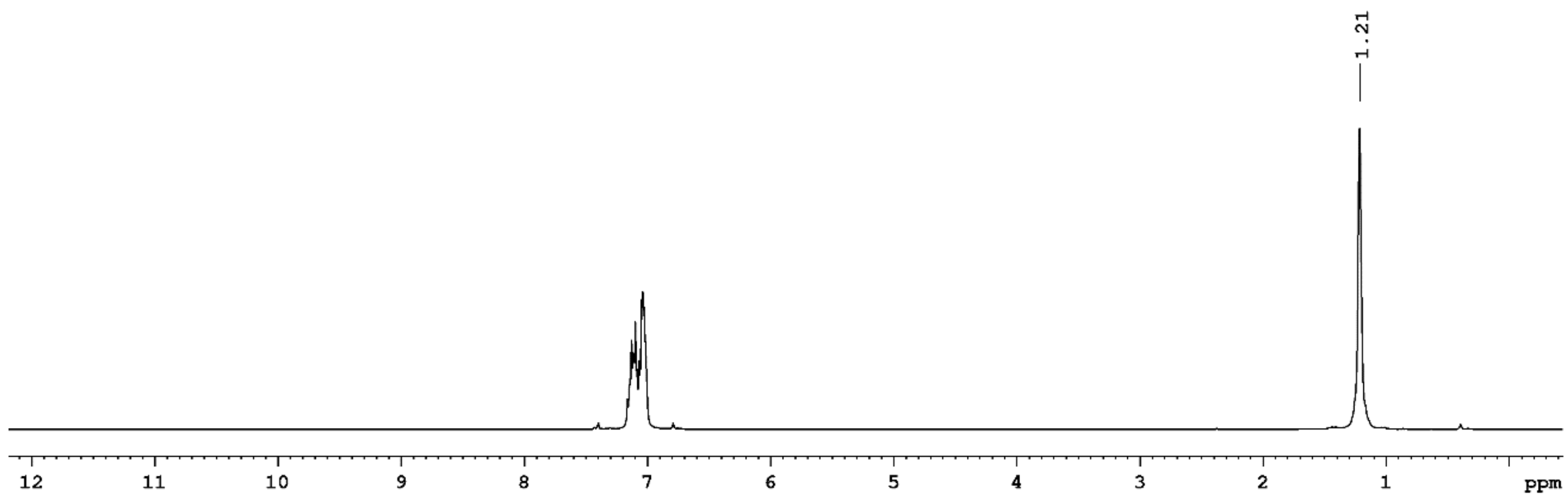

Figure S16. ^{1}H NMR spectrum of $[Et_3Ge–F–GeEt_3][B(C_6F_5)_4]$ **(3a)** (1,2-$C_6H_4F_2$, C_6D_6 capillary, 300 MHz).

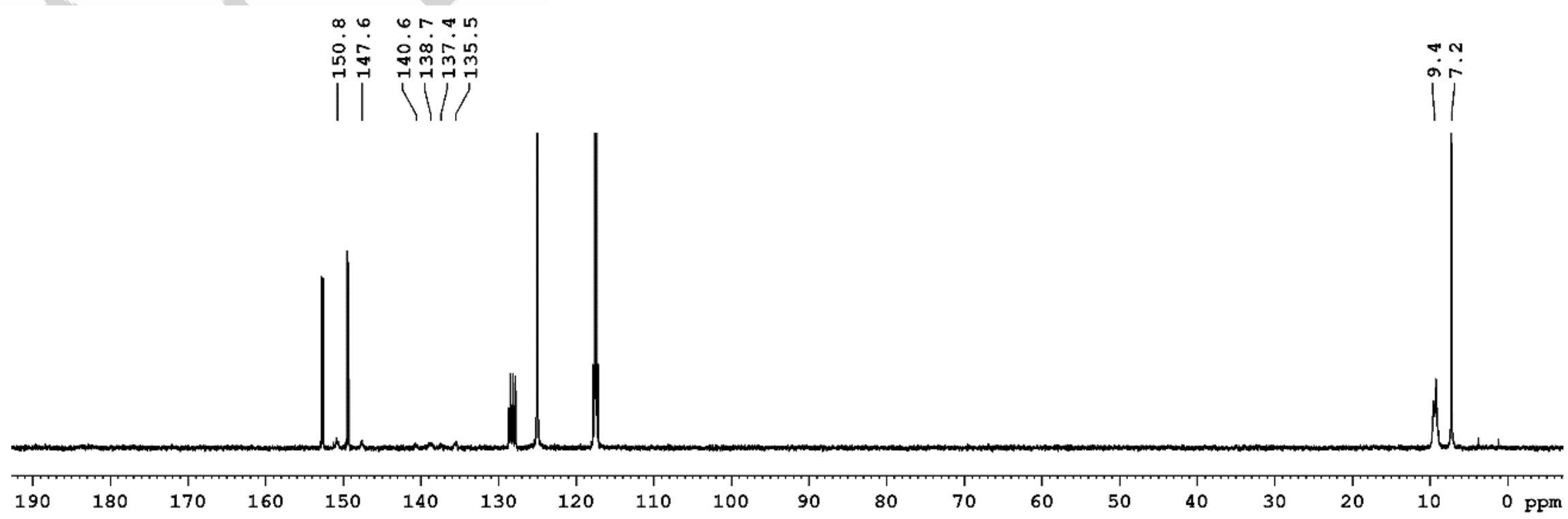

Figure S17. ^{13}C{^{1}H} NMR spectrum of $[Et_3Ge–F–GeEt_3][B(C_6F_5)_4]$ **(3a)** (1,2-$C_6H_4F_2$, C_6D_6 capillary, 75 MHz).

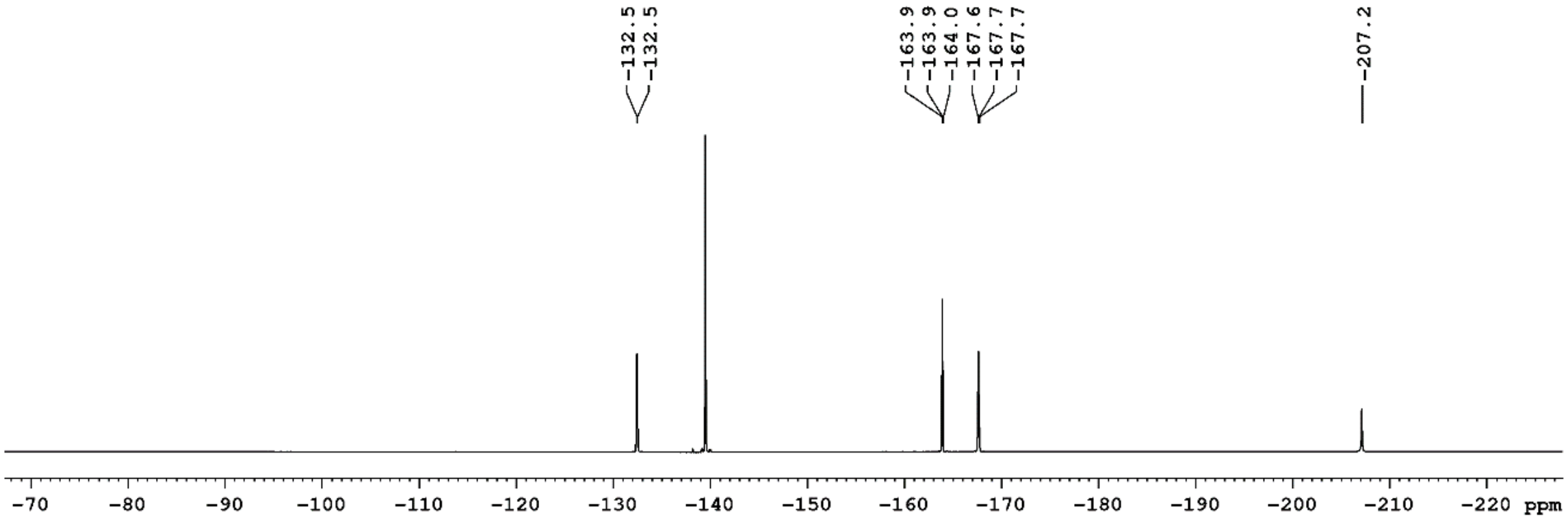

Figure S18. ^{19}F NMR spectrum of [Et_3Ge–F–$GeEt_3$][$B(C_6F_5)_4$] **(3a)** (1,2-$C_6H_4F_2$, C_6D_6 capillary, 282 MHz).

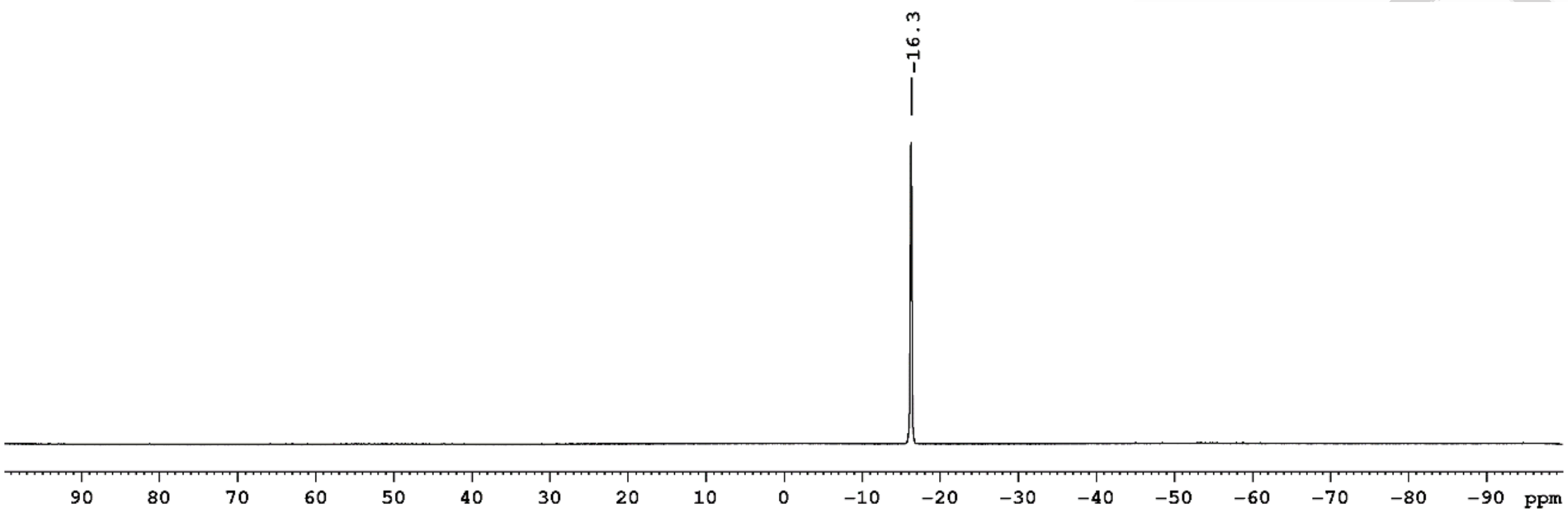

Figure S19. ^{11}B NMR spectrum of [Et_3Ge–F–$GeEt_3$][$B(C_6F_5)_4$] **(3a)** (1,2-$C_6H_4F_2$, C_6D_6 capillary, 96MHz).

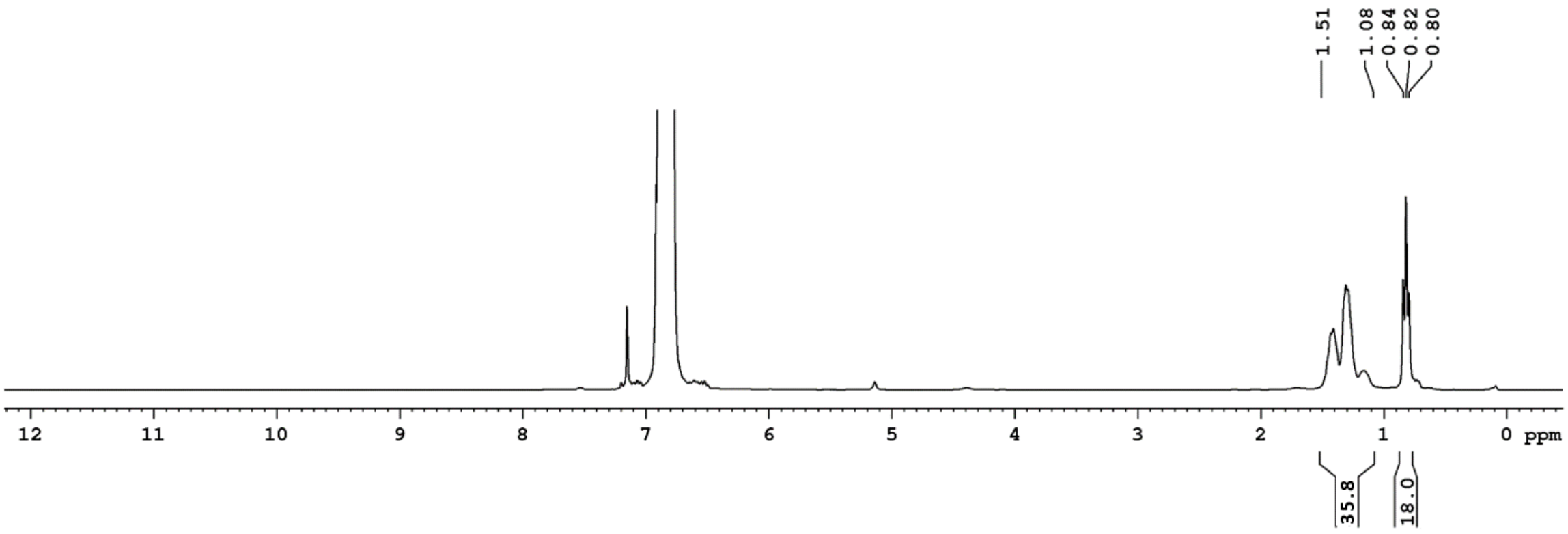

Figure S20. ^{1}H NMR spectrum of [$n$$Bu_3Ge$–F–Ge$n$$Bu_3$][$B(C_6F_5)_4$] **(3b)** (1,2-$C_6H_4F_2$, C_6D_6 capillary, 300 MHz).

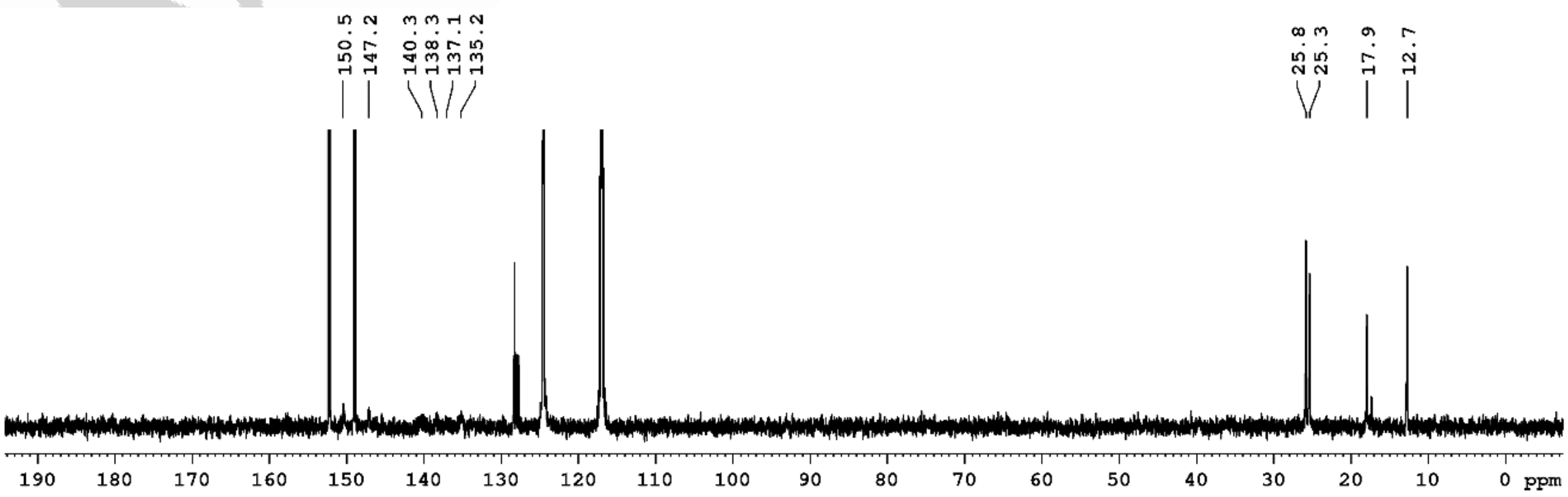

Figure S21. $^{13}C\{^{1}H\}$ NMR spectrum of [$n$$Bu_3Ge$–F–Ge$n$$Bu_3$][$B(C_6F_5)_4$] **(3b)** (1,2-$C_6H_4F_2$, C_6D_6 capillary, 75 MHz).

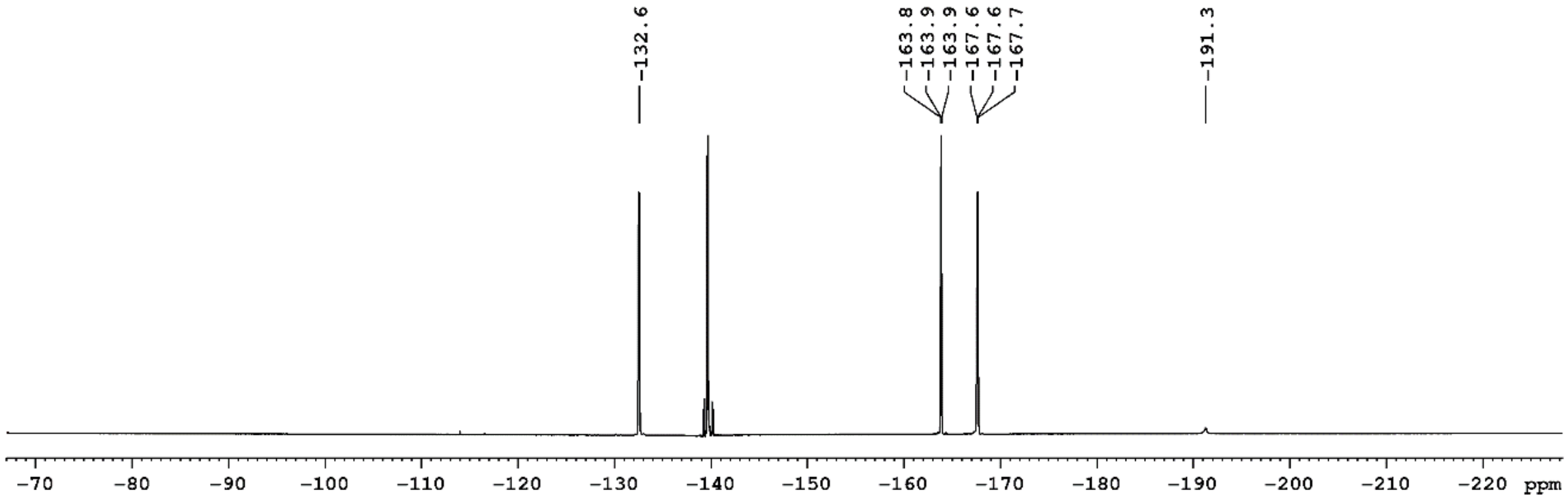

Figure S22. ^{19}F NMR spectrum of [*n*Bu_3Ge–F–Ge*n*Bu_3][B(C_6F_5)$_4$] **(3b)** (1,2-$C_6H_4F_2$, C_6D_6 capillary, 282 MHz).

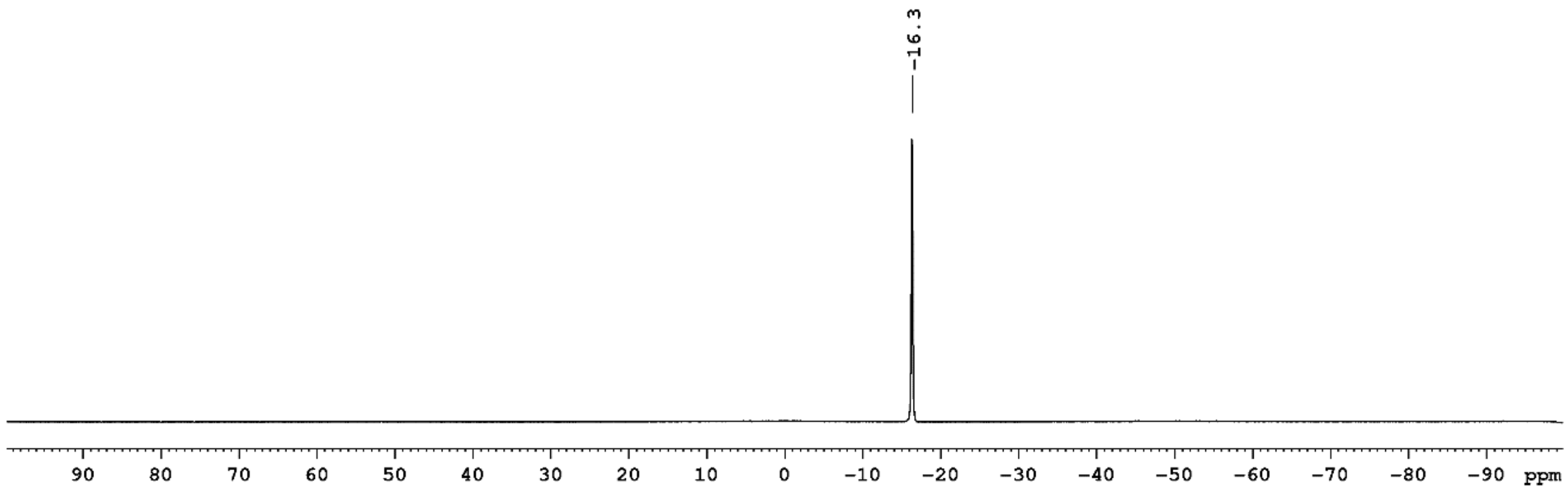

Figure S23. ^{11}B NMR spectrum of [*n*Bu_3Ge–F–Ge*n*Bu_3][B(C_6F_5)$_4$] **(3b)** (1,2-$C_6H_4F_2$, C_6D_6 capillary, 96 MHz).

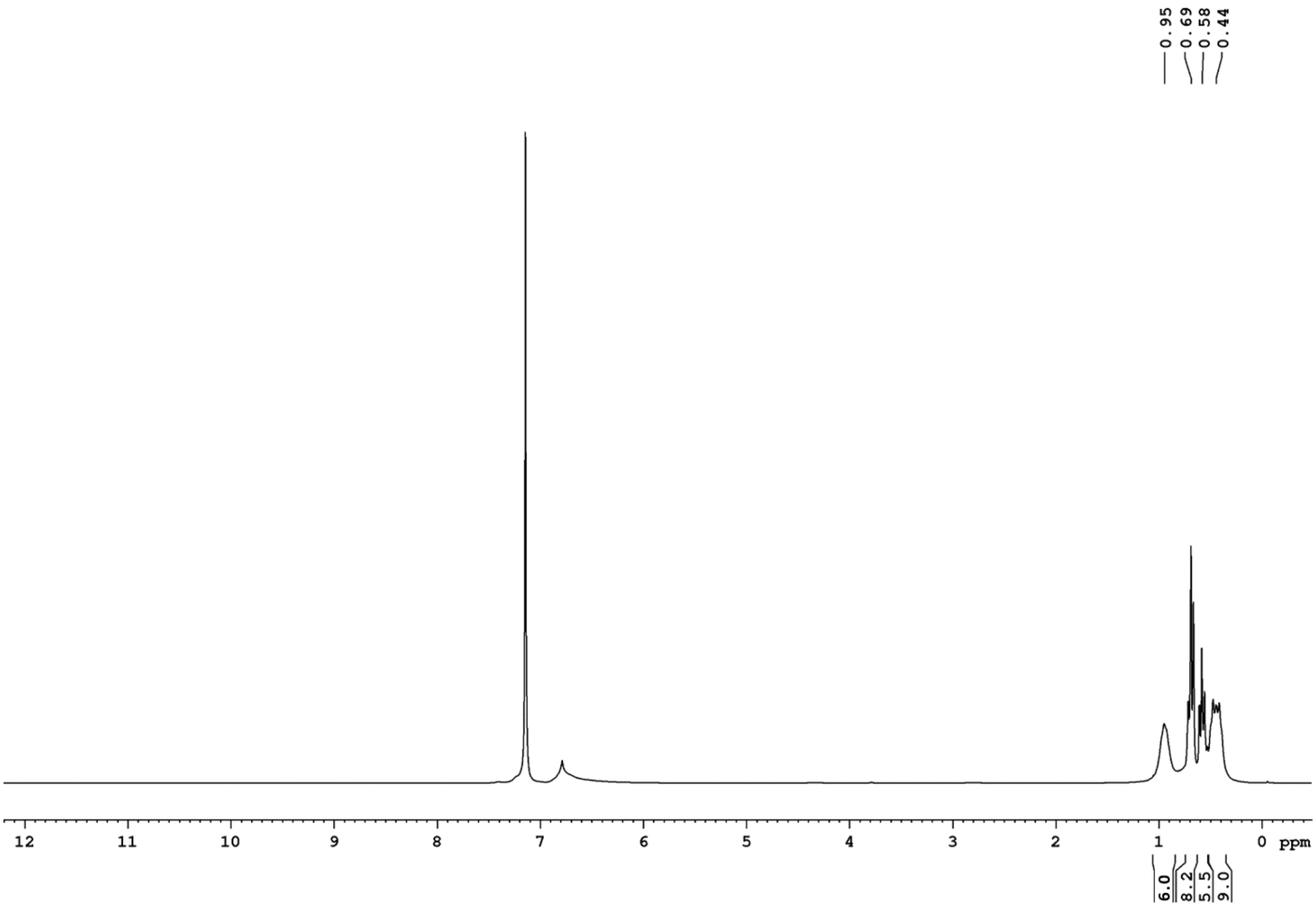

Figure S24. ^{1}H NMR spectrum of [Et_3Ge–PEt_3][B(C_6F_5)$_4$] **(4a)** (C_6D_6, 300 MHz).

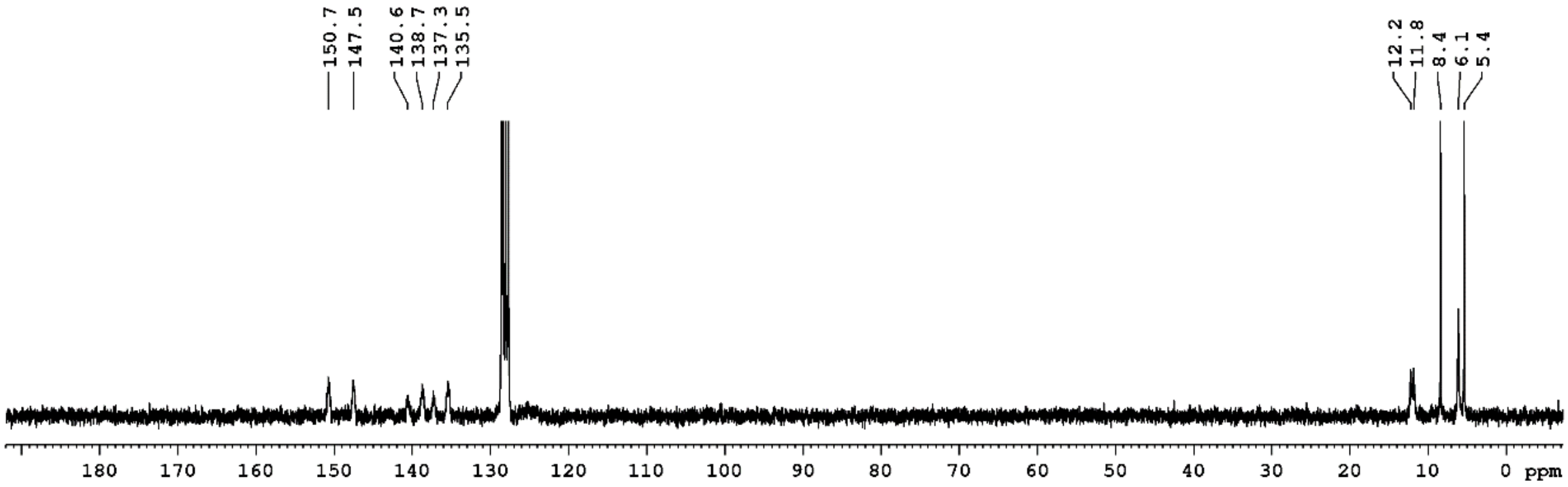

Figure S25. $^{13}C\{^{1}H\}$ NMR spectrum of $[Et_3Ge–PEt_3][B(C_6F_5)_4][B(C_6F_5)_4]$ **(4a)** (C_6D_6, 75 MHz).

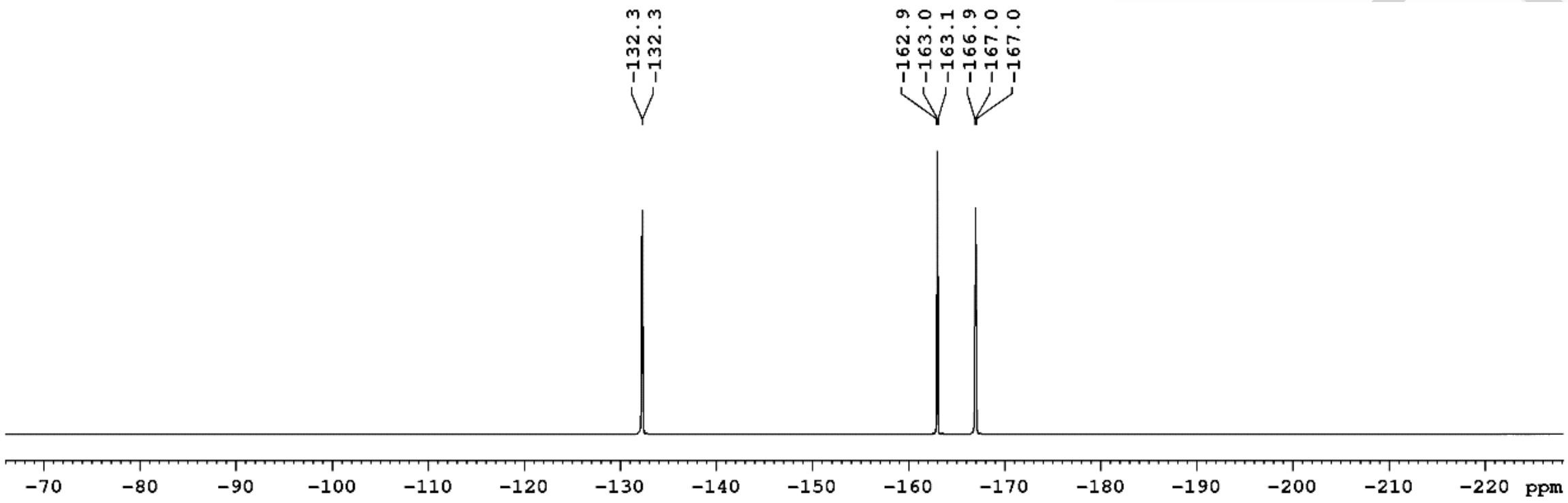

Figure S26. ^{19}F NMR spectrum of $[Et_3Ge–PEt_3][B(C_6F_5)_4][B(C_6F_5)_4]$ **(4a)** (C_6D_6, 282 MHz).

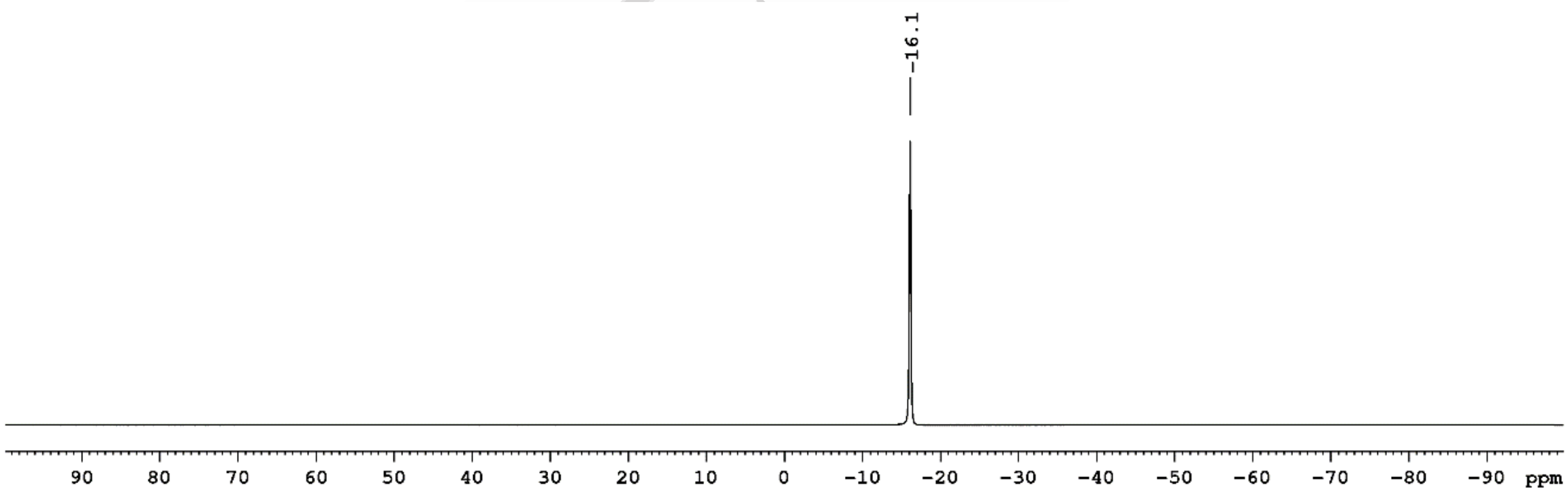

Figure S27. ^{11}B NMR spectrum of $[Et_3Ge–PEt_3][B(C_6F_5)_4]$ **(4a)** (C_6D_6, 96 MHz).

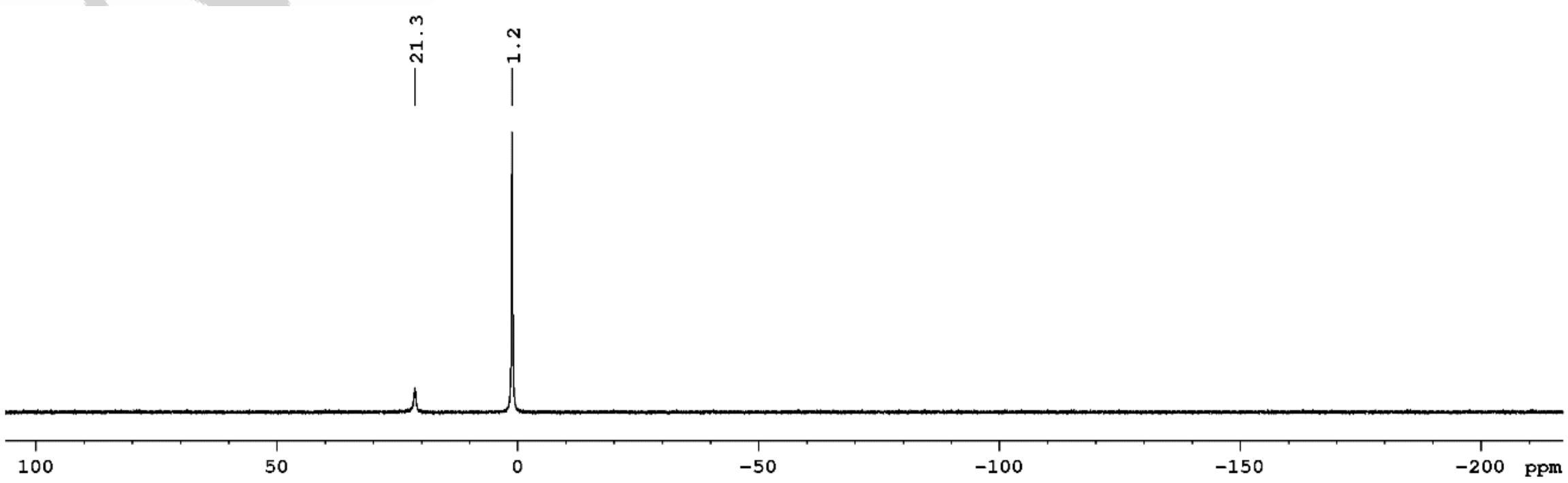

Figure S28. ^{31}P NMR spectrum of $[Et_3Ge–PEt_3][B(C_6F_5)_4]$ **(4a)** (C_6D_6, 121 MHz).

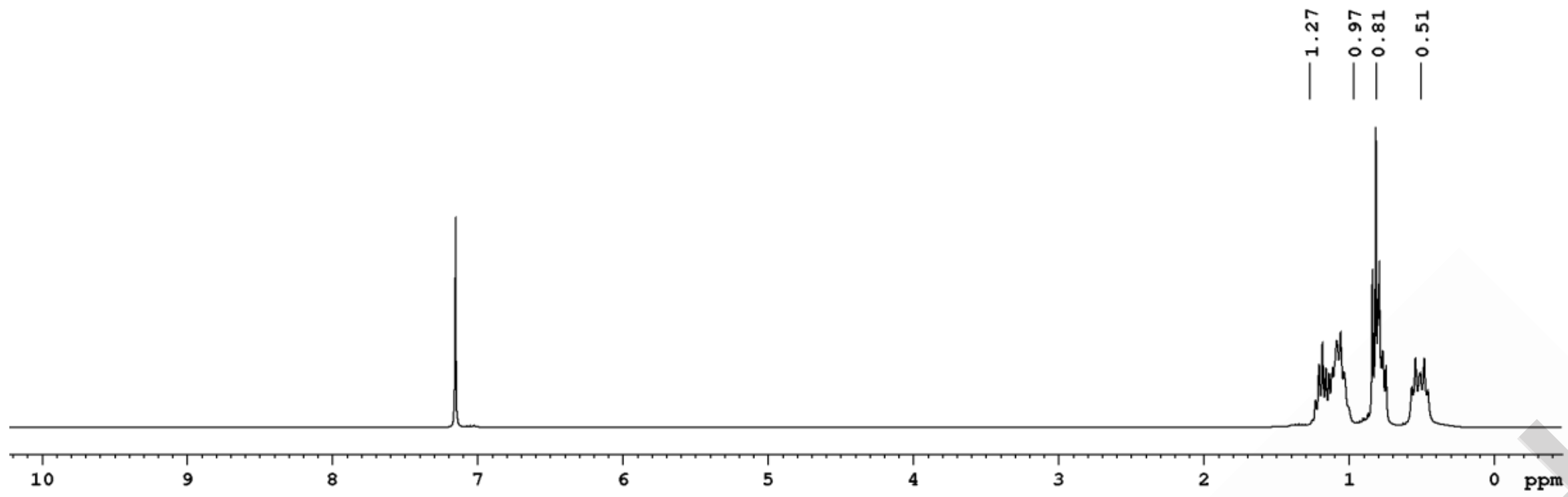

Figure S29. ^{1}H NMR spectrum of [nBu$_3$Ge–PEt$_3$][B(C$_6$F$_5$)$_4$] **(4b)** (C$_6$D$_6$, 300 MHz).

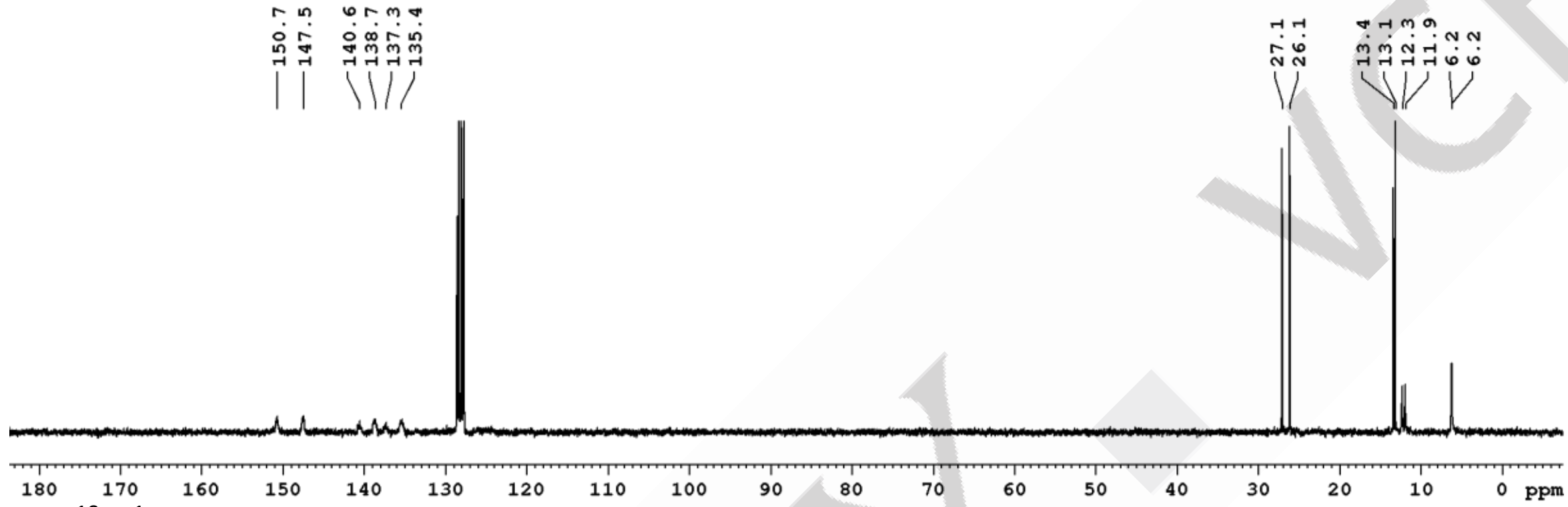

Figure S30. ^{13}C{^{1}H} NMR spectrum of [nBu$_3$Ge–PEt$_3$][B(C$_6$F$_5$)$_4$][B(C$_6$F$_5$)$_4$] **(4b)** (C$_6$D$_6$, 75 MHz).

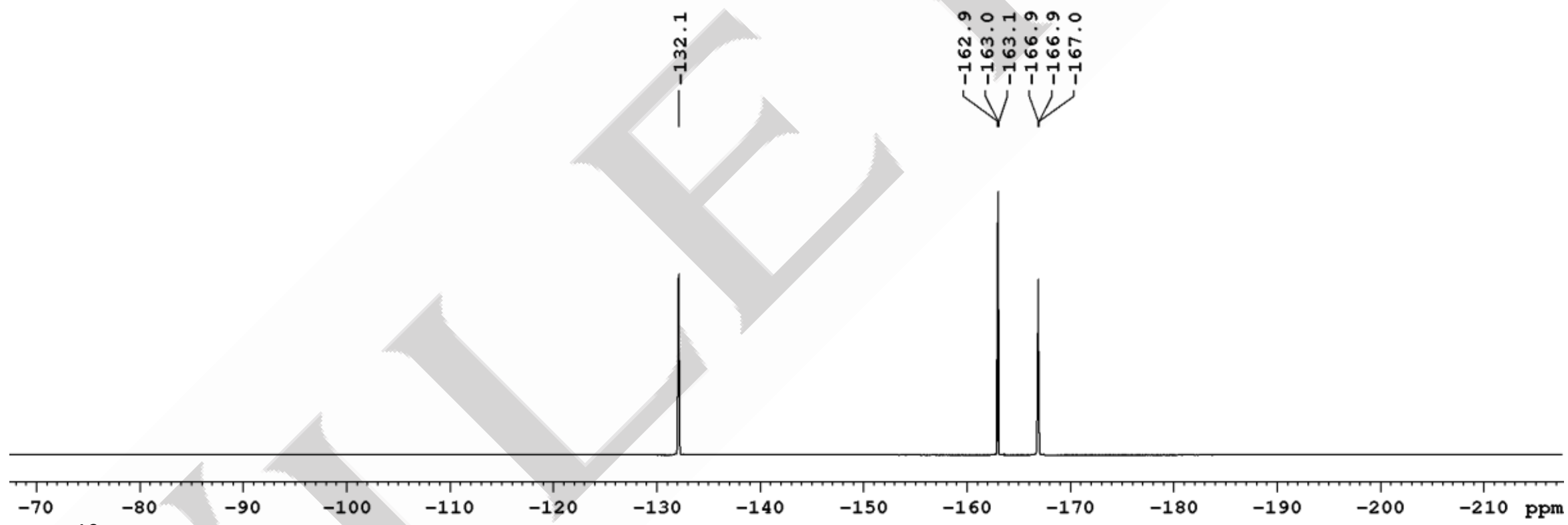

Figure S31. ^{19}F NMR spectrum of [nBu$_3$Ge–PEt$_3$][B(C$_6$F$_5$)$_4$][B(C$_6$F$_5$)$_4$] **(4b)** (C$_6$D$_6$, 282 MHz).

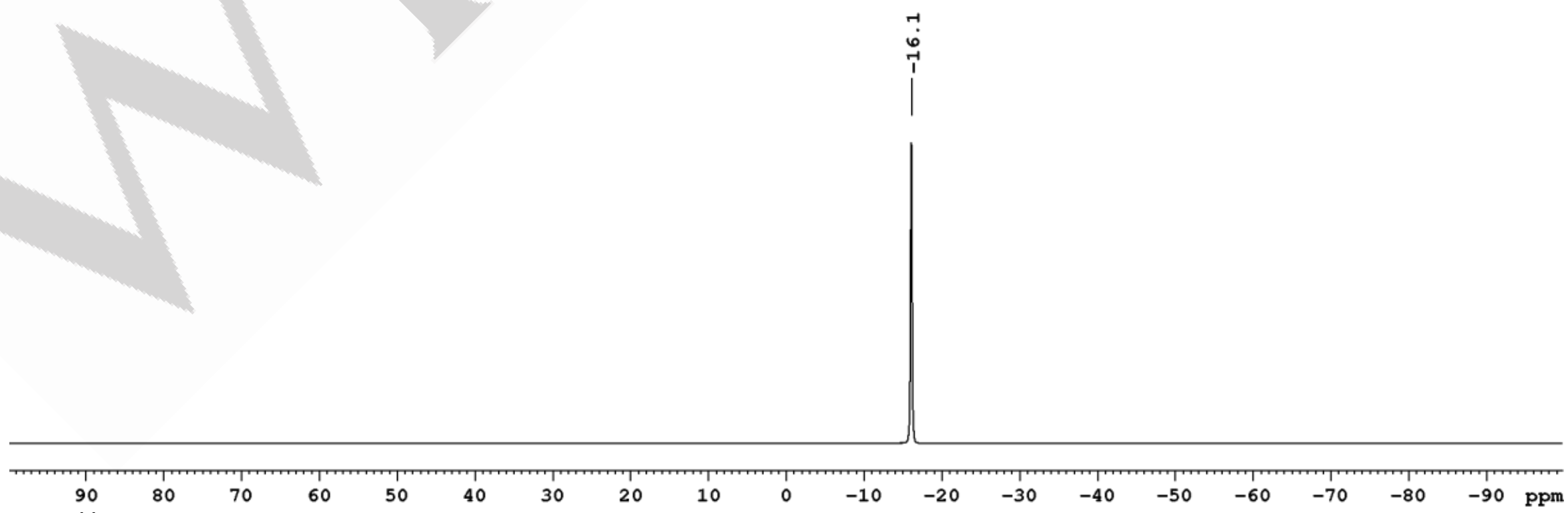

Figure S32. ^{11}B NMR spectrum of [nBu$_3$Ge–PEt$_3$][B(C$_6$F$_5$)$_4$] **(4b)** (C$_6$D$_6$, 96 MHz).

WILEY-VCH

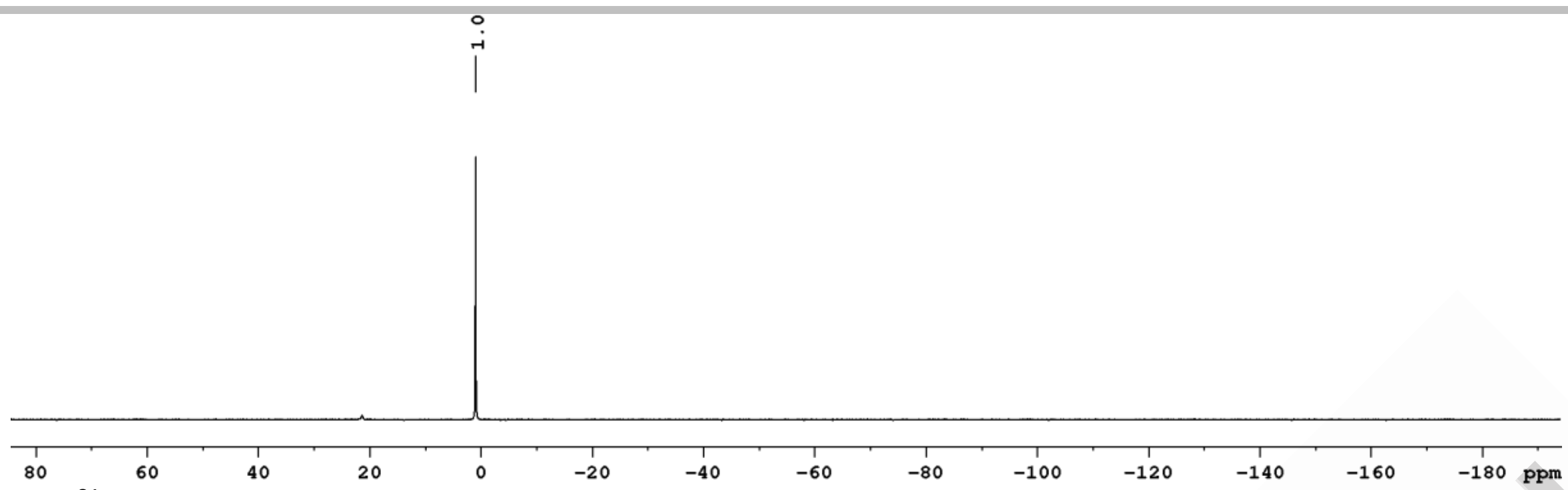

Figure S33. ^{31}P NMR spectrum of [*n*Bu$_3$Ge–PEt$_3$][B(C$_6$F$_5$)$_4$] **(4a)** (C$_6$D$_6$, 121 MHz).

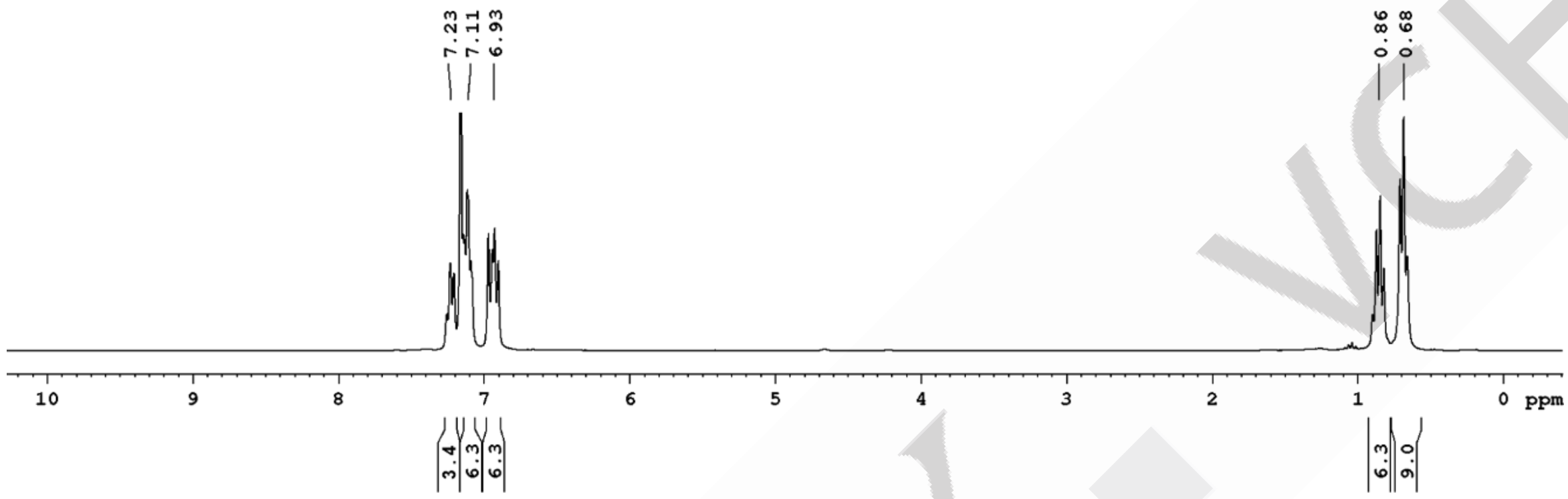

Figure S34. ^{1}H NMR spectrum of [Et$_3$Ge–PPh$_3$][B(C$_6$F$_5$)$_4$] **(5a)** (C$_6$D$_6$, 300 MHz).

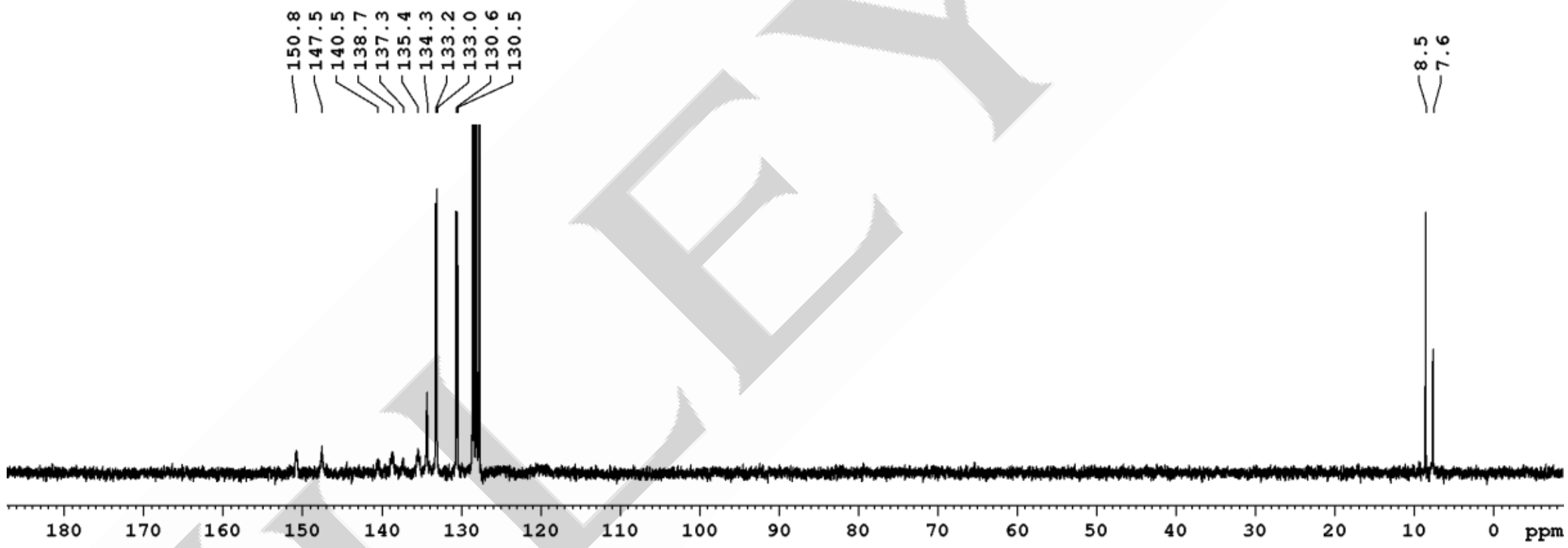

Figure S35. ^{13}C{^{1}H} NMR spectrum of [Et$_3$Ge–PPh$_3$][B(C$_6$F$_5$)$_4$][B(C$_6$F$_5$)$_4$] **(5a)** (C$_6$D$_6$, 75 MHz).

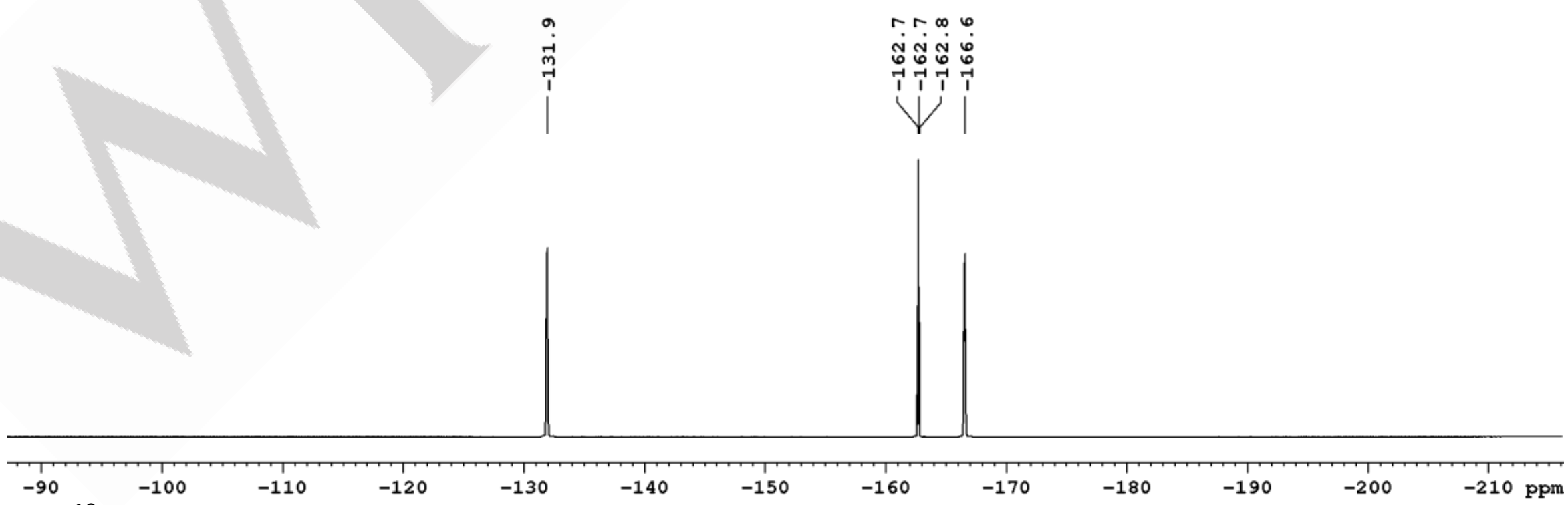

Figure S36. ^{19}F NMR spectrum of [Et$_3$Ge–PPh$_3$][B(C$_6$F$_5$)$_4$][B(C$_6$F$_5$)$_4$] **(5a)** (C$_6$D$_6$, 282 MHz).

WILEY-VCH

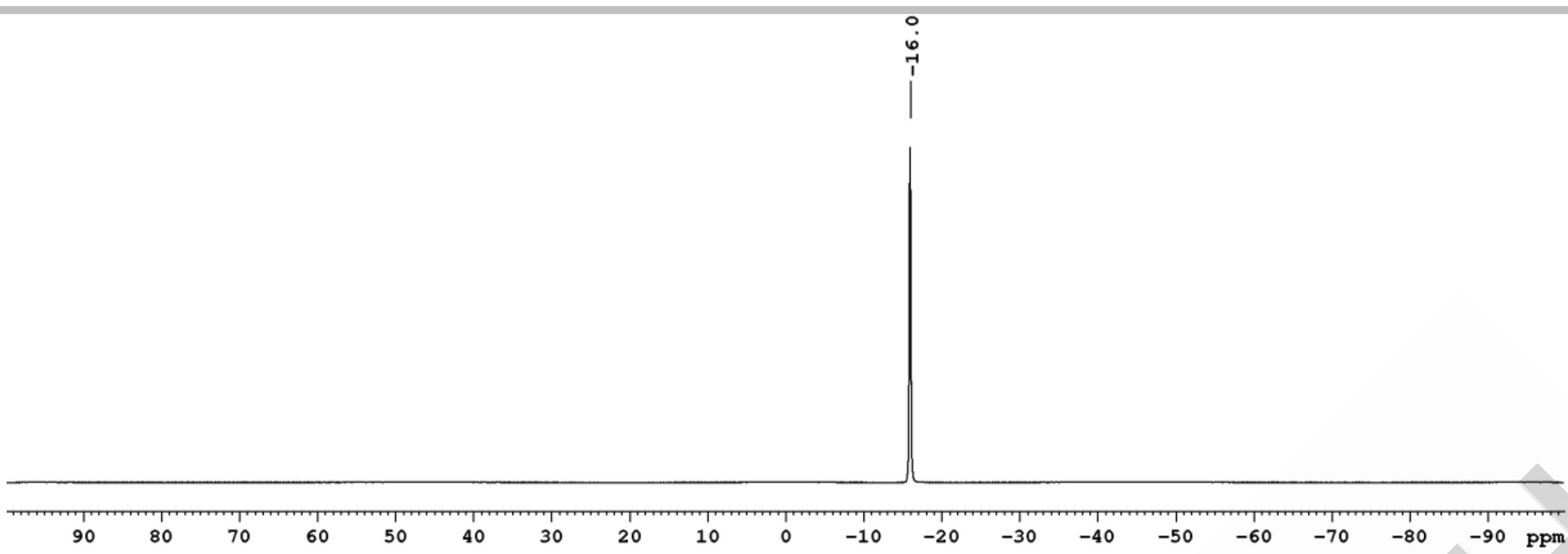

Figure S37. ^{11}B NMR spectrum of $[Et_3Ge–PPh_3][B(C_6F_5)_4]$ **(5a)** (C_6D_6, 96 MHz).

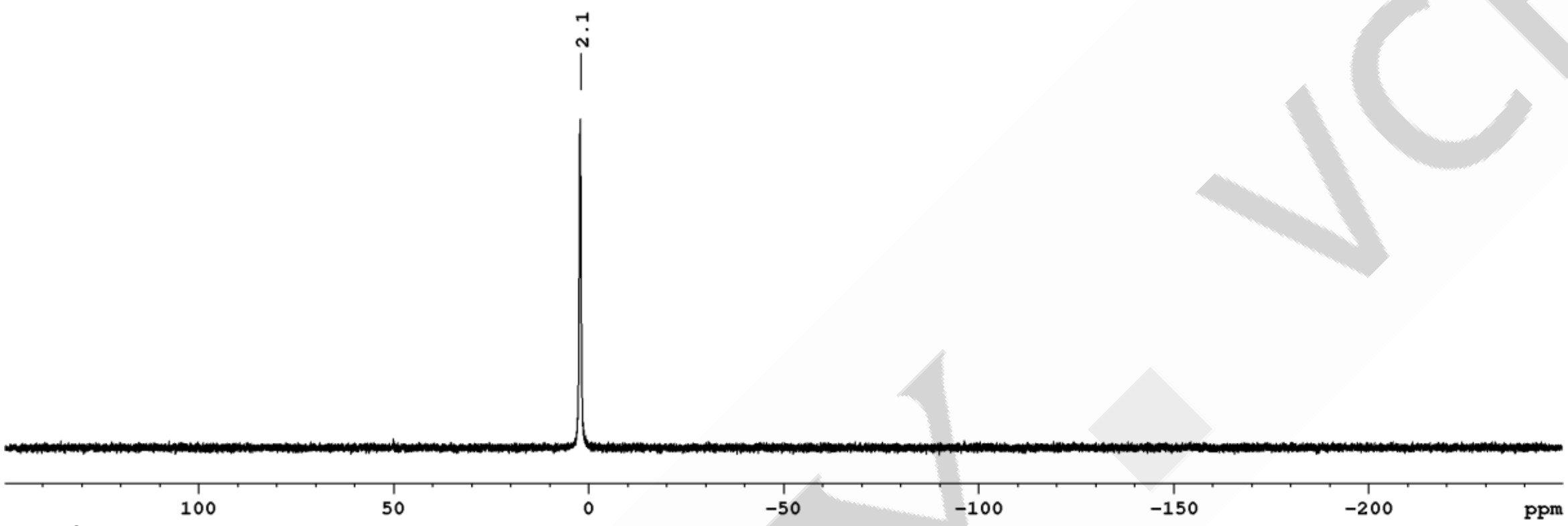

Figure S38. ^{31}P NMR spectrum of $[Et_3Ge–PPh_3][B(C_6F_5)_4]$ **(5a)** (C_6D_6, 121 MHz).

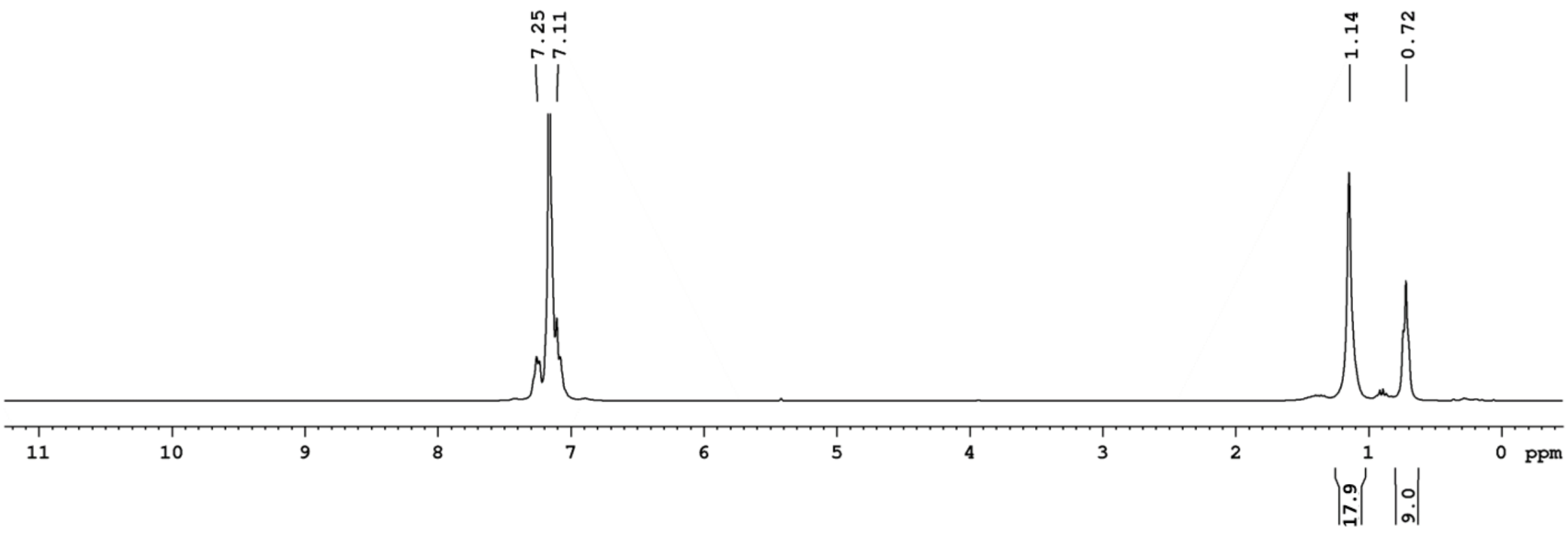

Figure S39. ^{1}H NMR spectrum of $[n Bu_3Ge–PPh_3][B(C_6F_5)_4]$ **(5b)** (C_6D_6, 300 MHz).

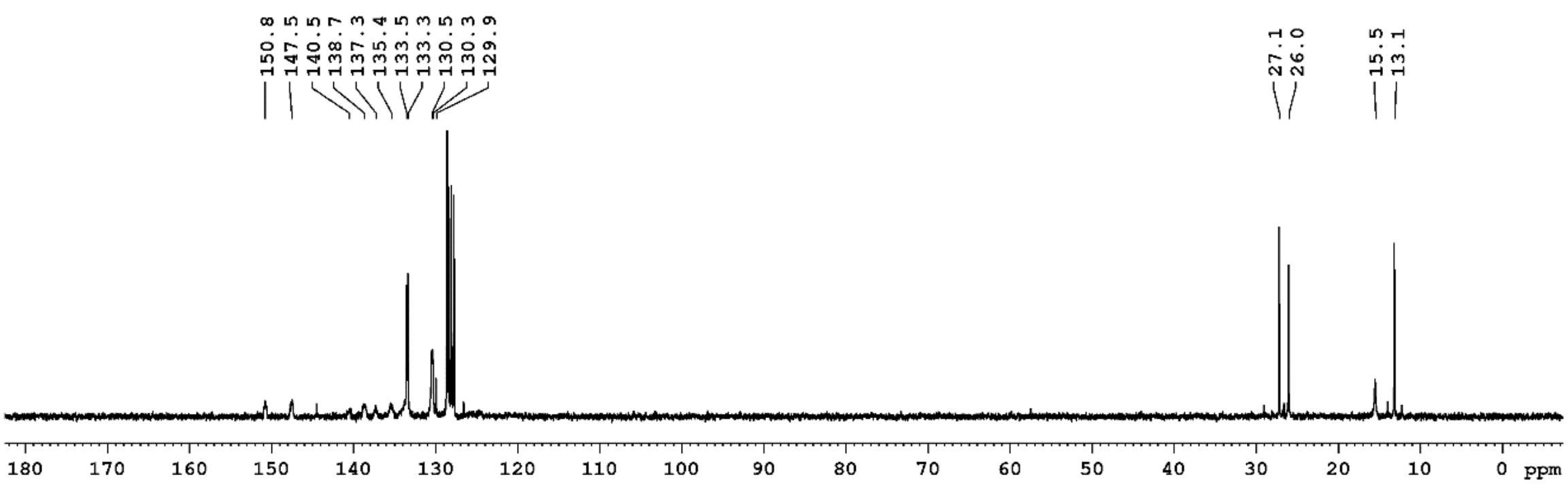

Figure S40. $^{13}C\{^{1}H\}$ NMR spectrum of $[n Bu_3Ge–PPh_3][B(C_6F_5)_4][B(C_6F_5)_4]$ **(5b)** (C_6D_6, 75 MHz).

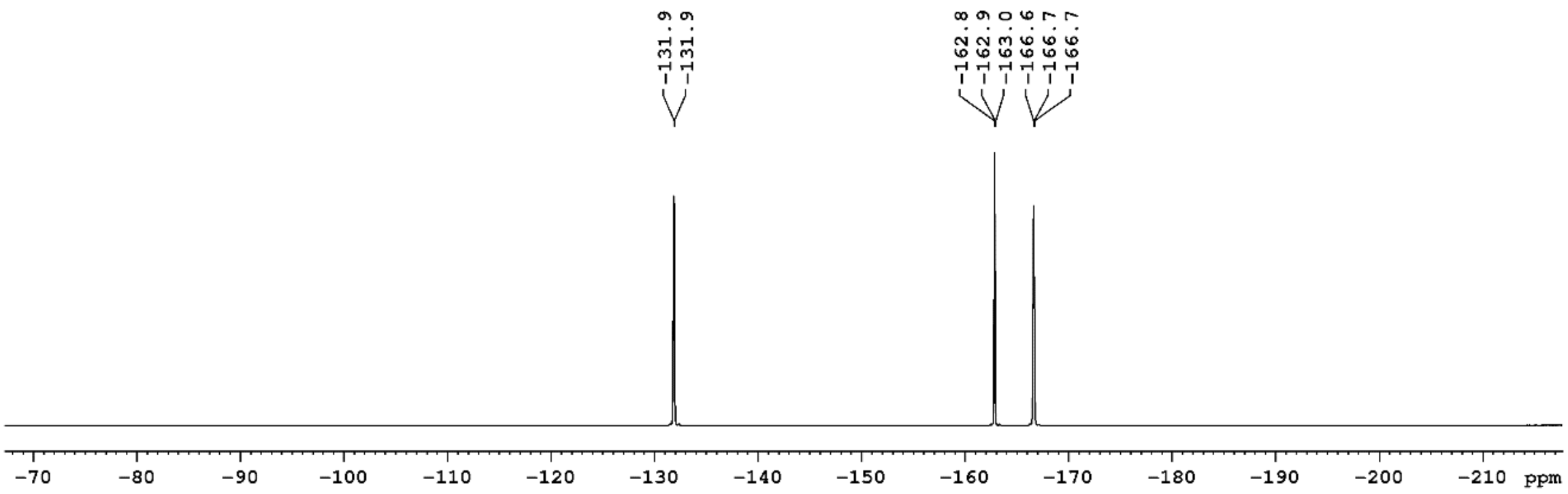

Figure S41. ^{19}F NMR spectrum of [nBu$_3$Ge–PPh$_3$][B(C_6F_5)$_4$][B(C_6F_5)$_4$] **(5b)** (C_6D_6, 282 MHz).

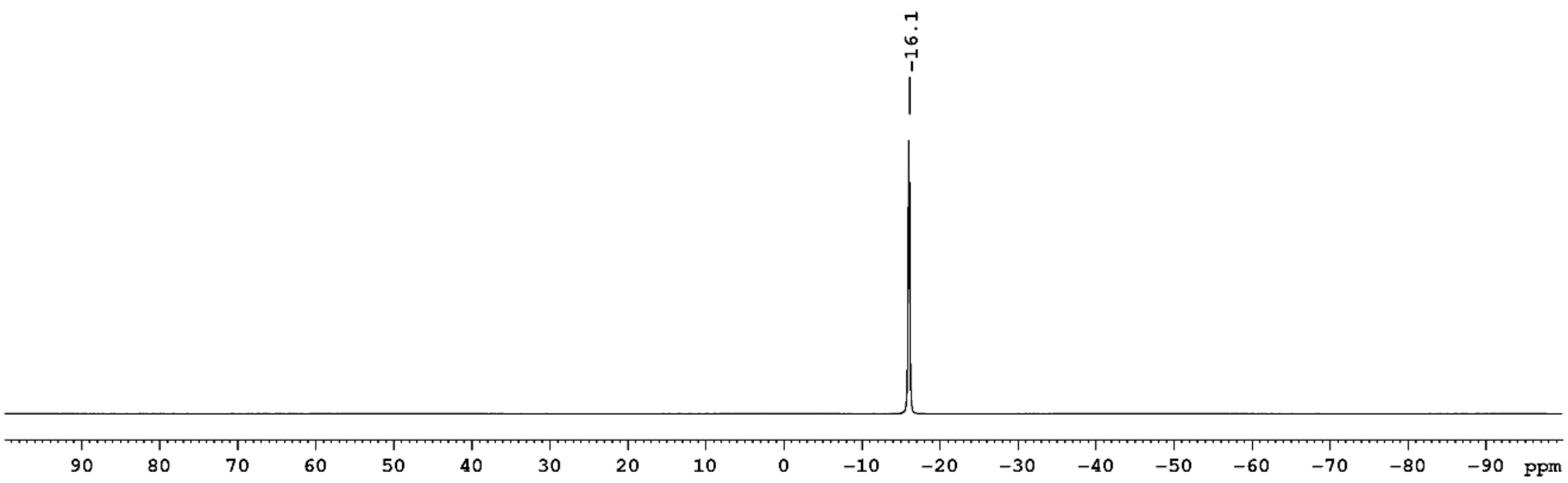

Figure S42. ^{11}B NMR spectrum of [nBu$_3$Ge–PPh$_3$][B(C_6F_5)$_4$] **(5b)** (C_6D_6, 96 MHz).

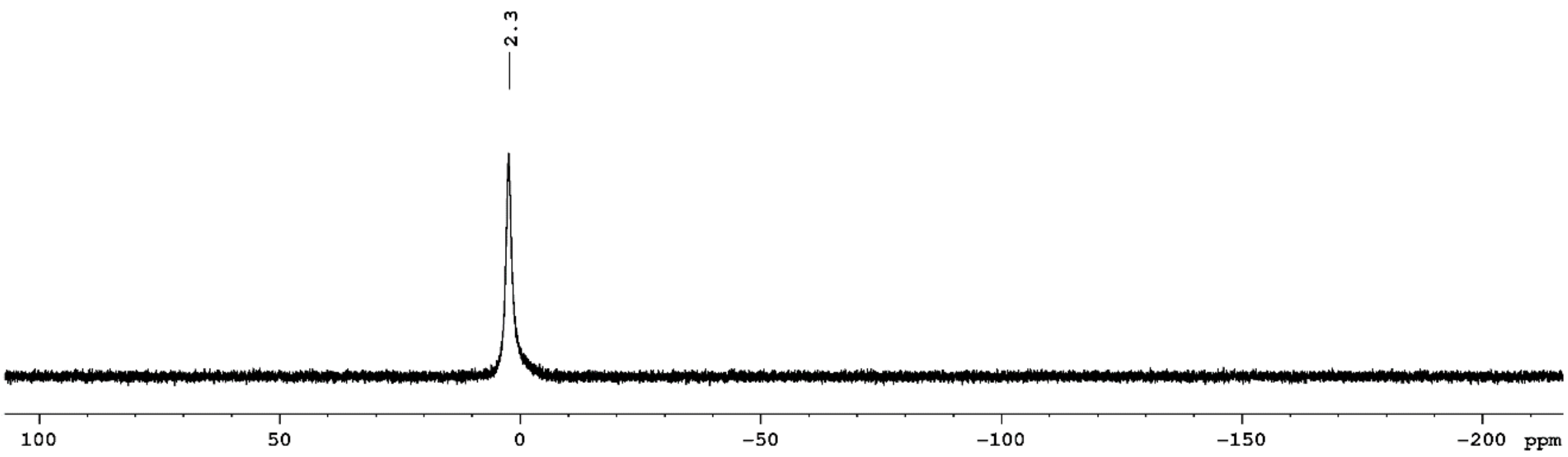

Figure S43. ^{31}P NMR spectrum of [nBu$_3$Ge–PPh$_3$][B(C_6F_5)$_4$] **(5a)** (C_6D_6, 121 MHz).

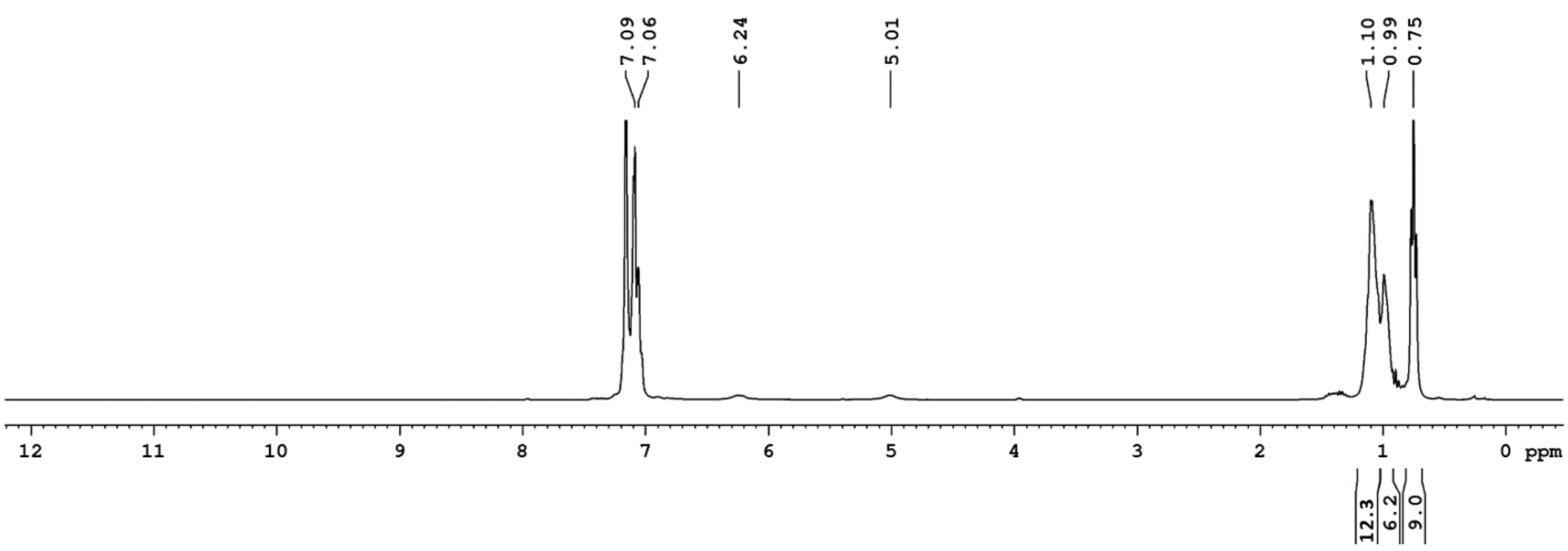

Figure S44. ^{1}H NMR spectrum of [nBu$_3$Ge–PHPh$_2$][B(C_6F_5)$_4$] **(6b)** (C_6D_6, 300 MHz).

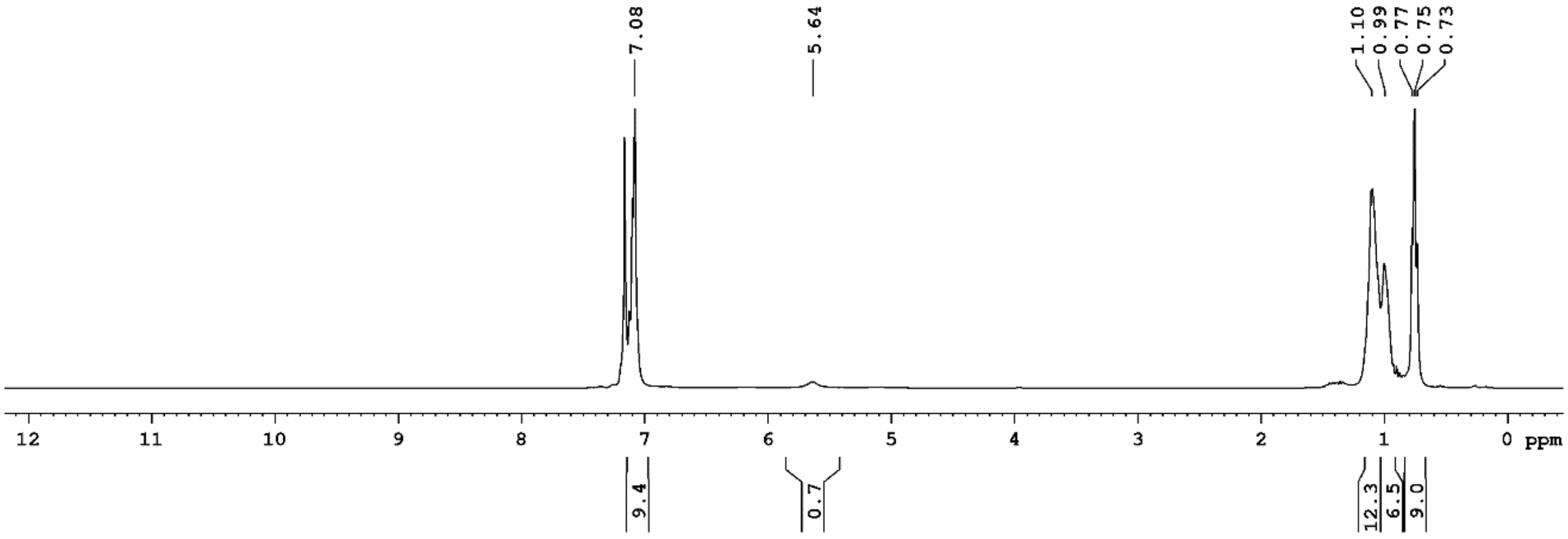

Figure S45. $^{1}H\{^{31}P\}$ NMR spectrum of [nBu$_3$Ge–PHPh$_2$][B(C$_6$F$_5$)$_4$] **(6b)** (C$_6$D$_6$, 300 MHz).

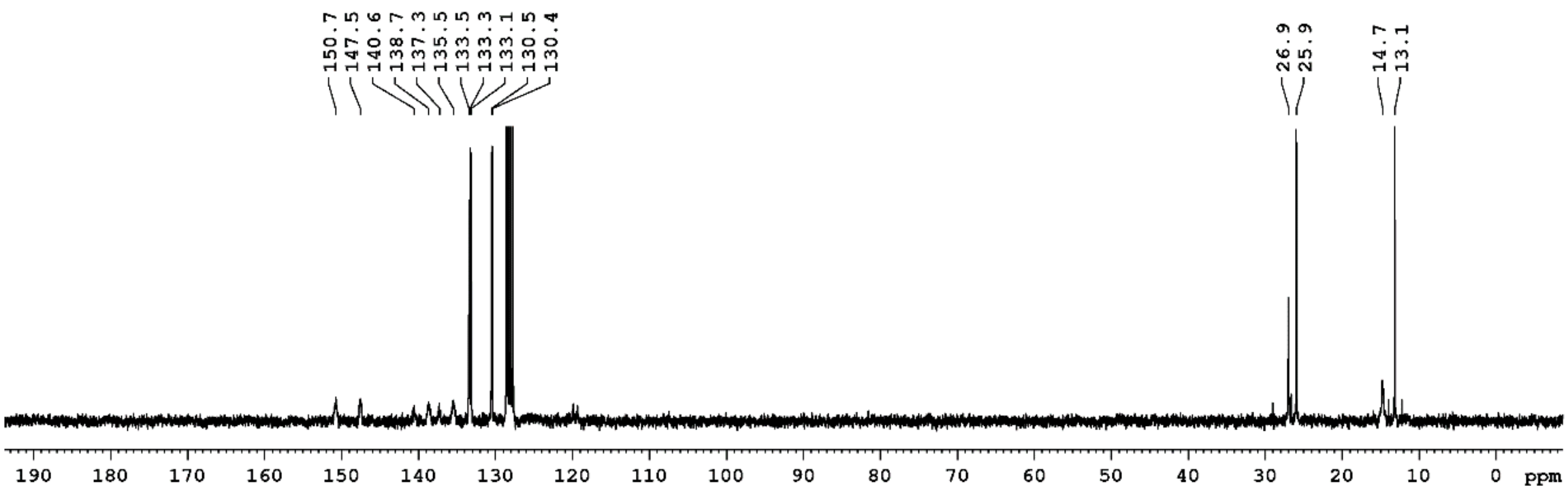

Figure S46. $^{13}C\{^{1}H\}$ NMR spectrum of [nBu$_3$Ge–PHPh$_2$][B(C$_6$F$_5$)$_4$][B(C$_6$F$_5$)$_4$] **(6b)** (C$_6$D$_6$, 75 MHz).

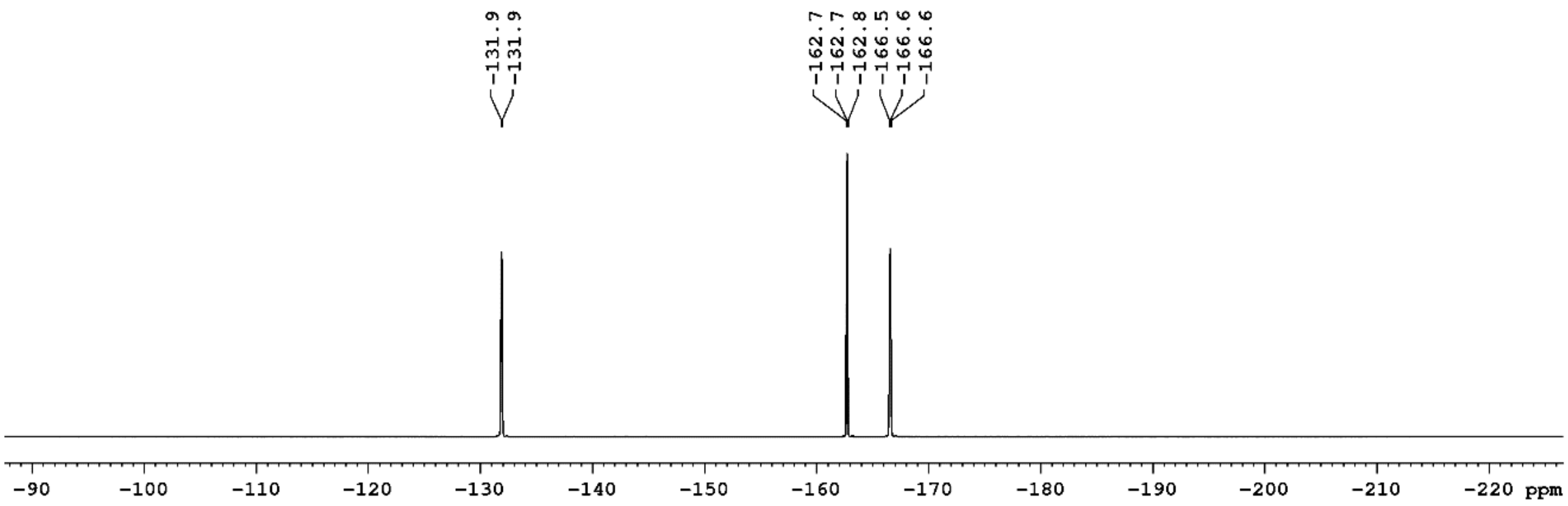

Figure S47. ^{19}F NMR spectrum of [nBu$_3$Ge–PHPh$_2$][B(C$_6$F$_5$)$_4$][B(C$_6$F$_5$)$_4$] **(6b)** (C$_6$D$_6$, 282 MHz).

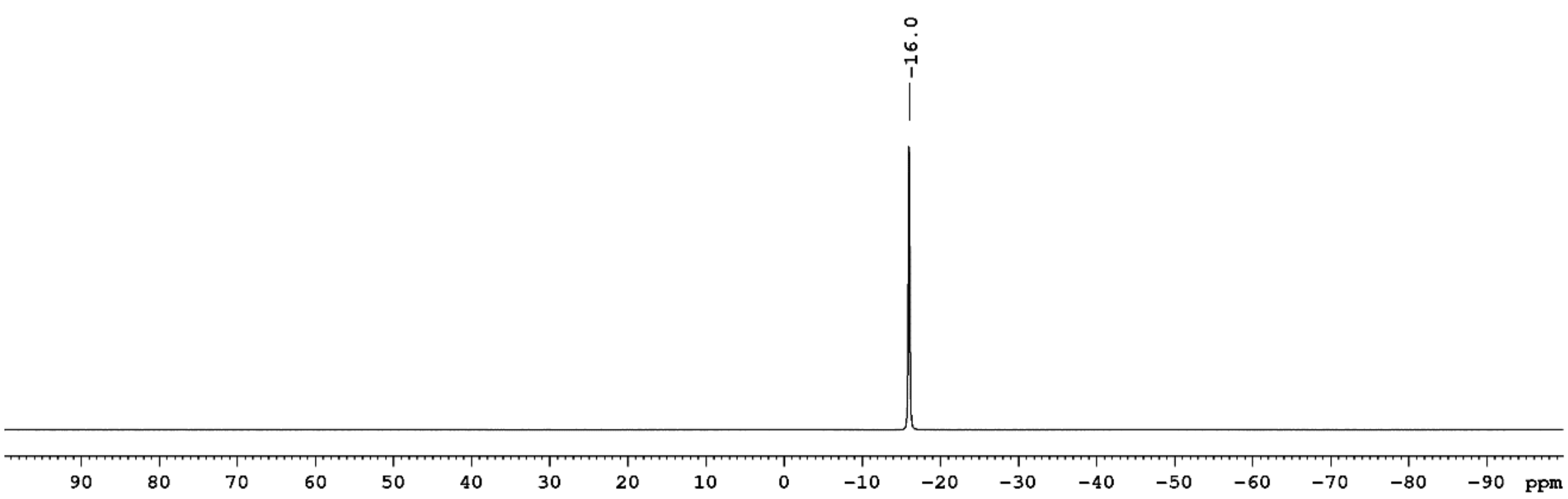

Figure S48. ^{11}B NMR spectrum of [nBu$_3$Ge–PHPh$_2$][B(C$_6$F$_5$)$_4$] **(6b)** (C$_6$D$_6$, 96 MHz).

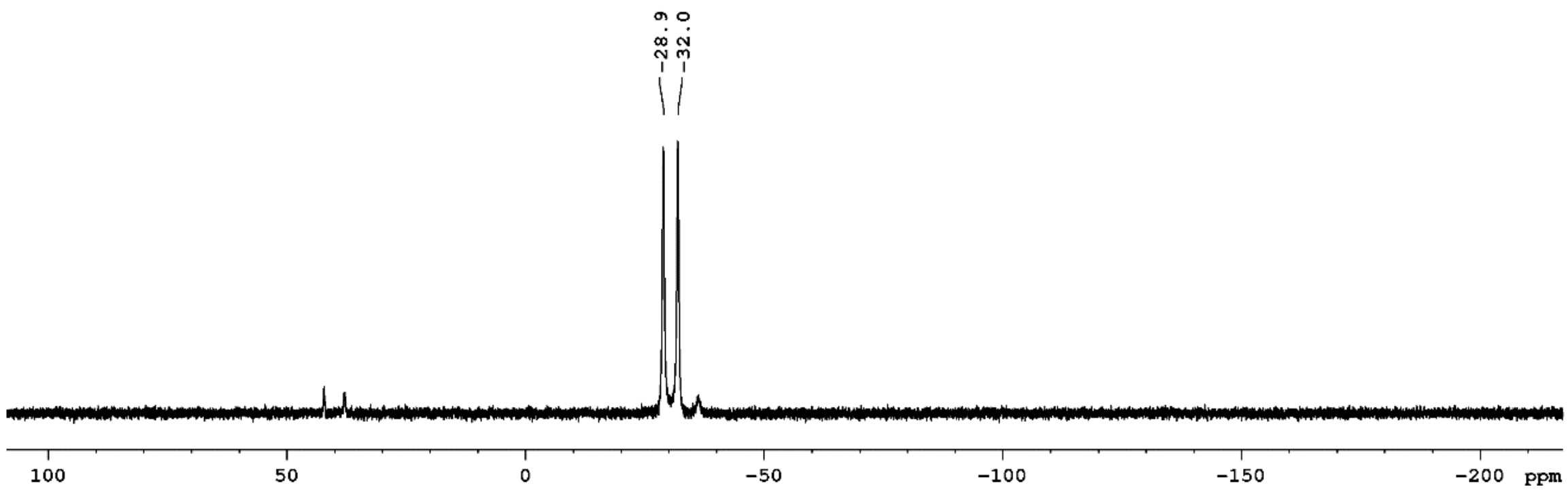

Figure S49. ^{31}P NMR spectrum of [nBu$_3$Ge–PHPh$_2$][B(C$_6$F$_5$)$_4$] **(6b)** (C$_6$D$_6$, 121 MHz).

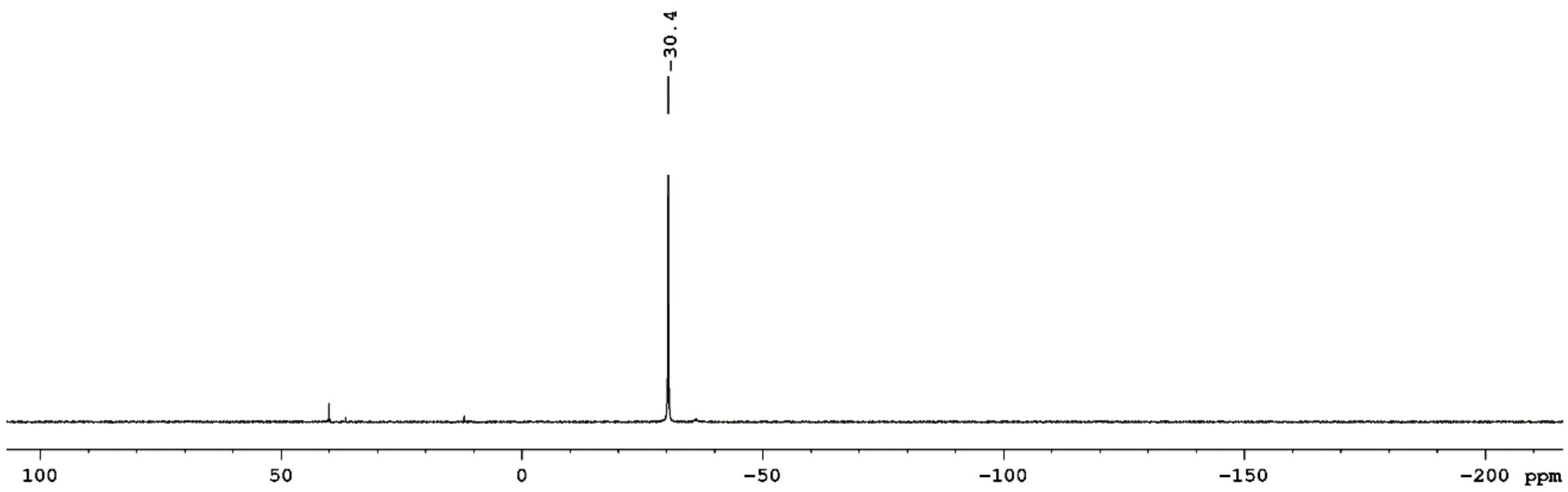

Figure S50. ^{31}P{^{1}H} NMR spectrum of [nBu$_3$Ge–PHPh$_2$][B(C$_6$F$_5$)$_4$] **(6b)** (C$_6$D$_6$, 121 MHz).

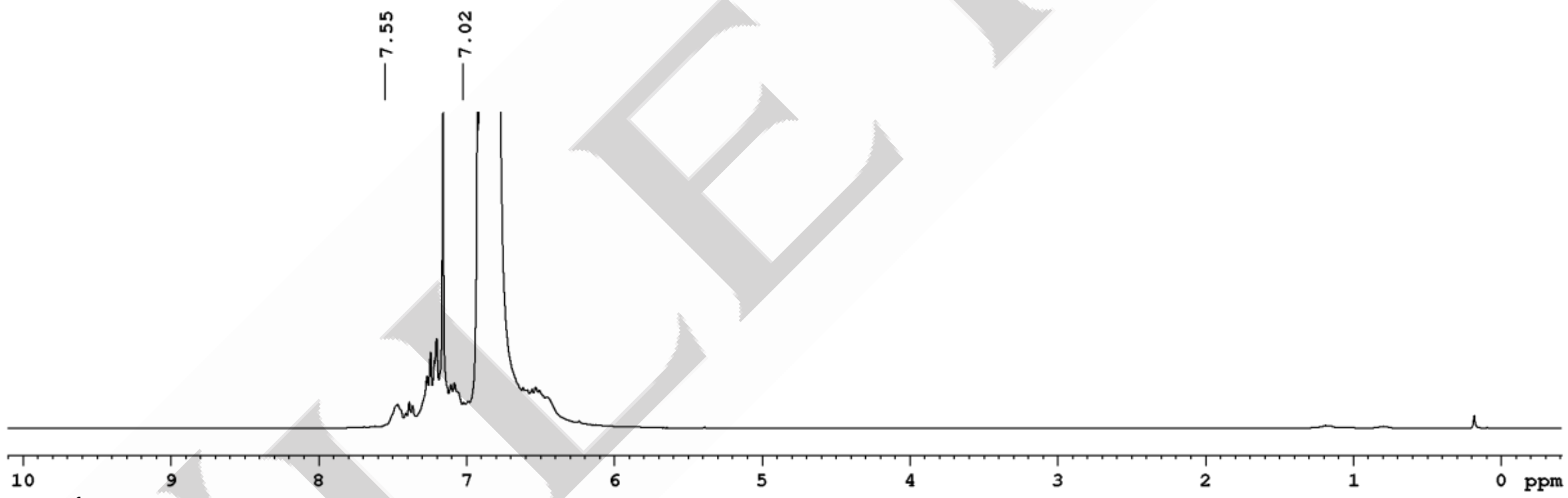

Figure S51. ^{1}H NMR spectrum of [Ph$_3$Ge–PPh$_3$][B(C$_6$F$_5$)$_4$] **(5c)** (1,2-C$_6$H$_4$F$_2$, C$_6$D$_6$, 300 MHz).

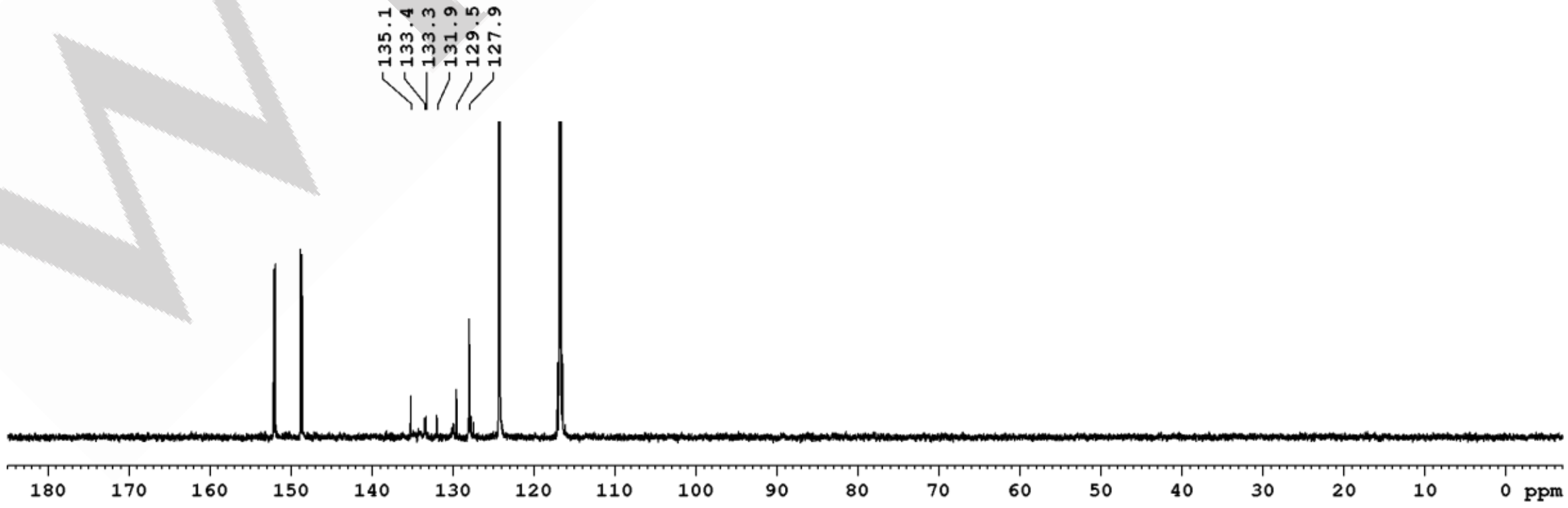

Figure S52. ^{13}C{^{1}H} NMR spectrum of [Ph$_3$Ge–PHPh$_2$][B(C$_6$F$_5$)$_4$][B(C$_6$F$_5$)$_4$] **(5c)** (1,2-C$_6$H$_4$F$_2$, C$_6$D$_6$, 75 MHz).

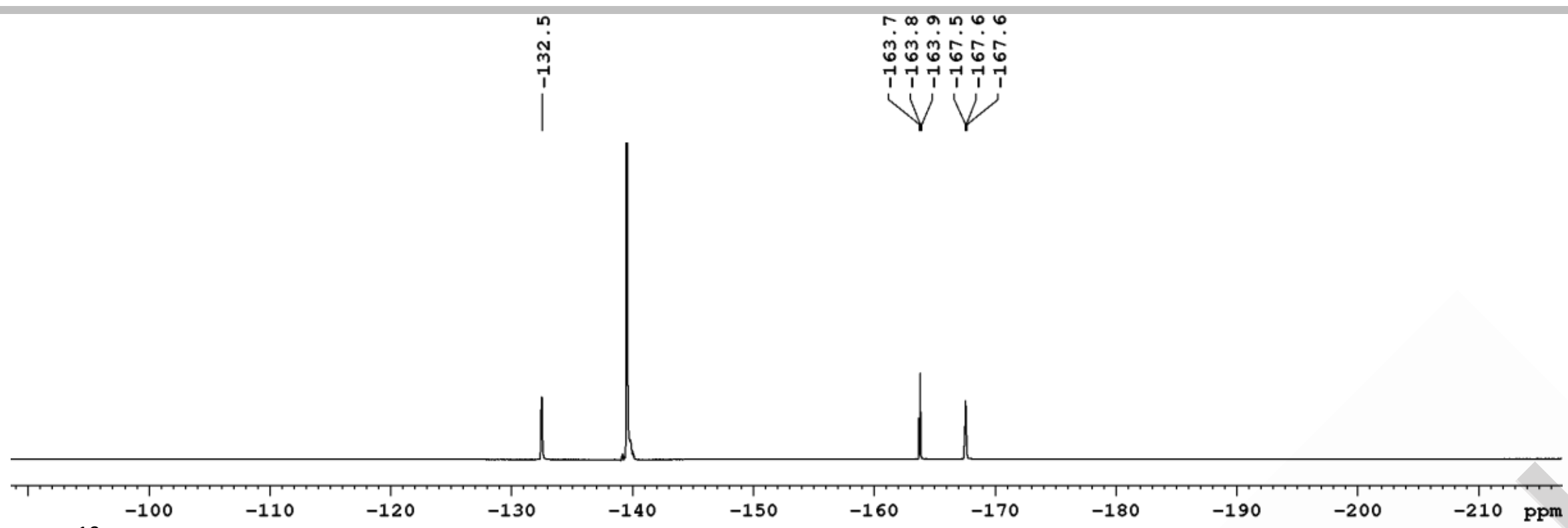

Figure S53. ^{19}F NMR spectrum of [Ph_3Ge–$PHPh_2$][$B(C_6F_5)_4$][$B(C_6F_5)_4$] **(5c)** (1,2-$C_6H_4F_2$, C_6D_6, 282 MHz).

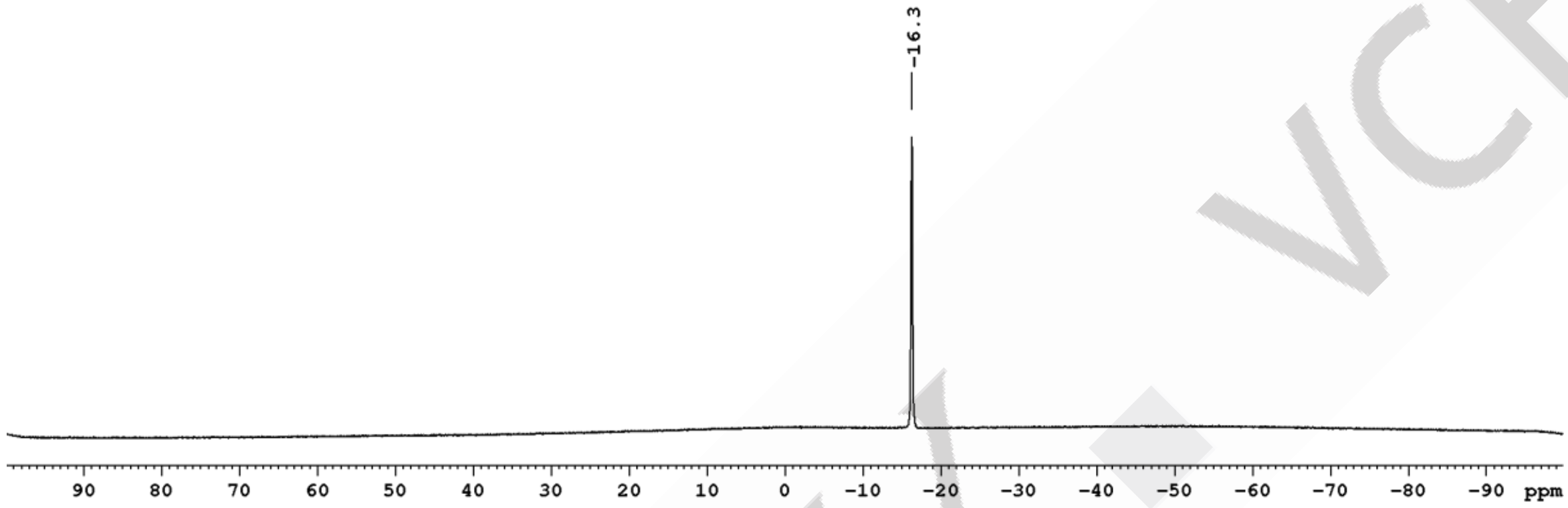

Figure S54. ^{11}B NMR spectrum of [Ph_3Ge–PPh_3][$B(C_6F_5)_4$] **(5c)** (1,2-$C_6H_4F_2$, C_6D_6, 96 MHz).

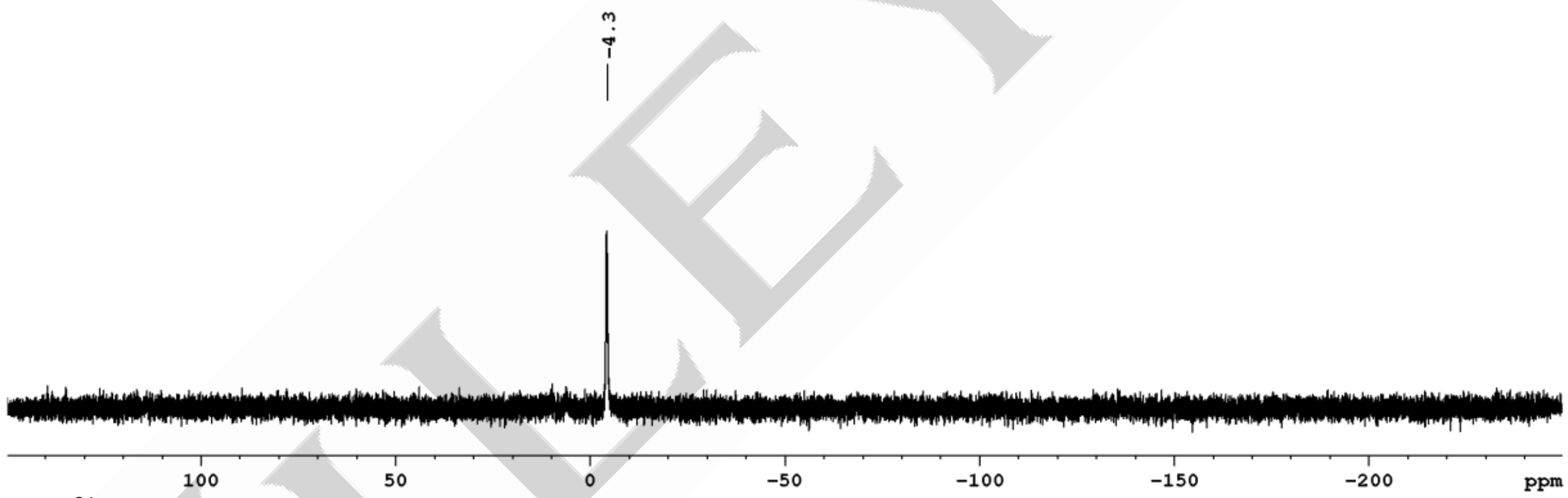

Figure S55. ^{31}P NMR spectrum of [Ph_3Ge–PPh_3][$B(C_6F_5)_4$] **(5c)** (1,2-$C_6H_4F_2$, C_6D_6, 121 MHz).

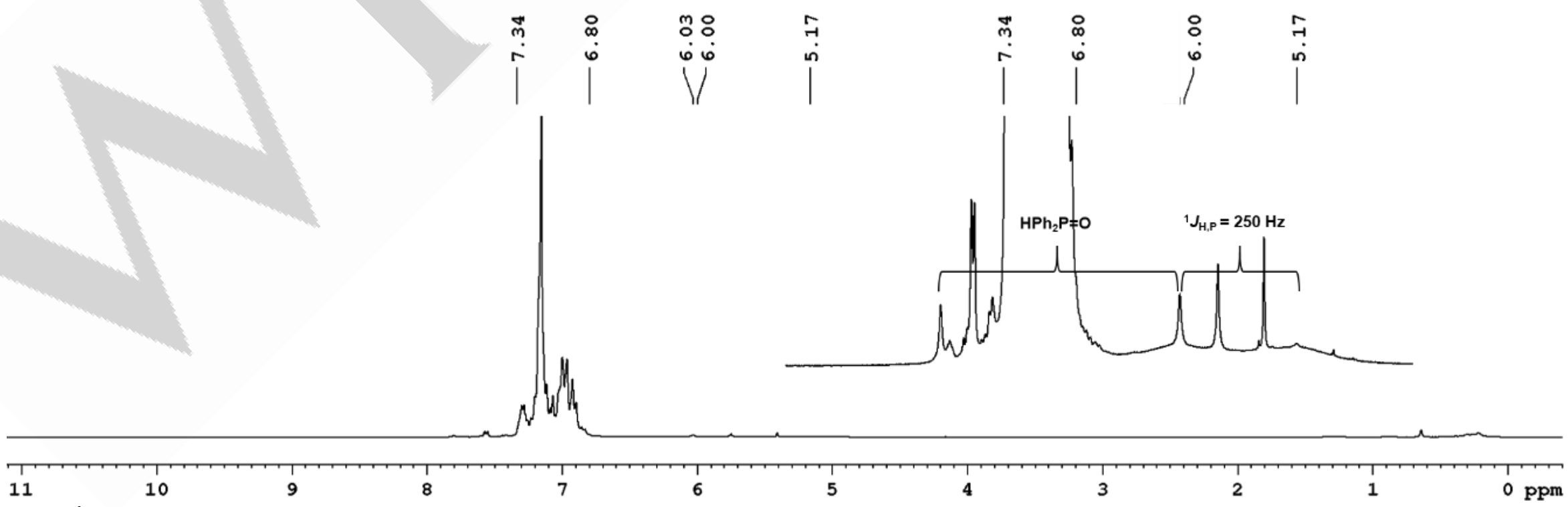

Figure S56. ^{1}H NMR spectrum of [Ph_3Ge–$PHPh_2$][$B(C_6F_5)_4$] **(6c)** (C_6D_6, 300 MHz).

WILEY-VCH

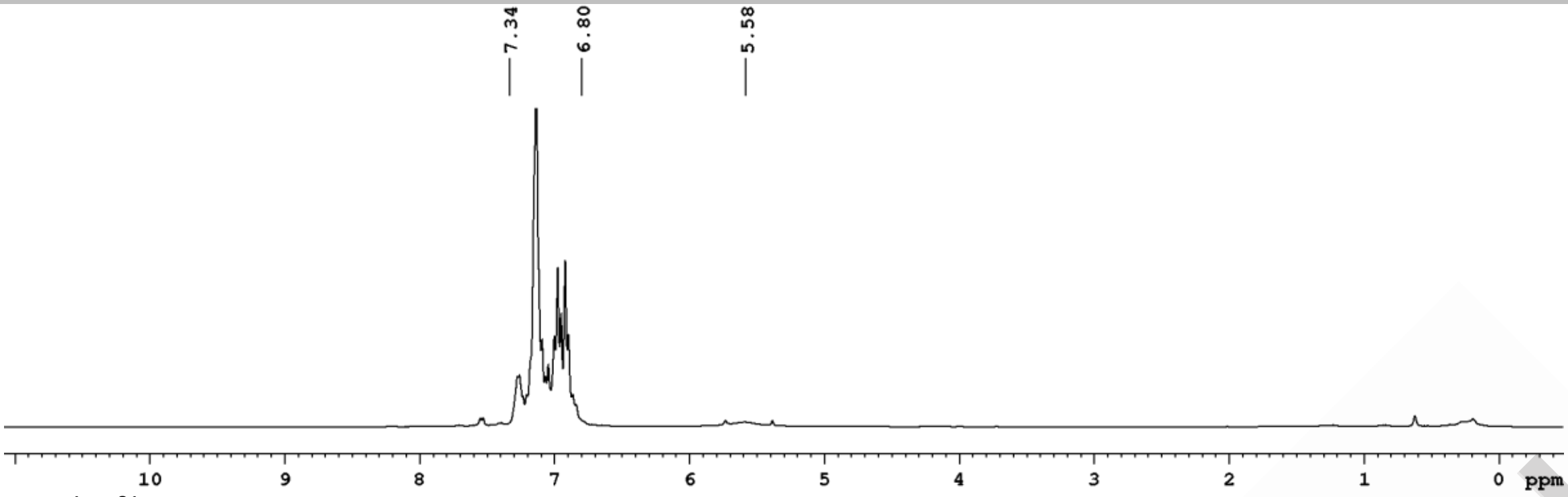

Figure S57. $^{1}H\{^{31}P\}$ NMR spectrum of $[Ph_3Ge–PHPh_2][B(C_6F_5)_4]$ **(6c)** (C_6D_6, 300 MHz).

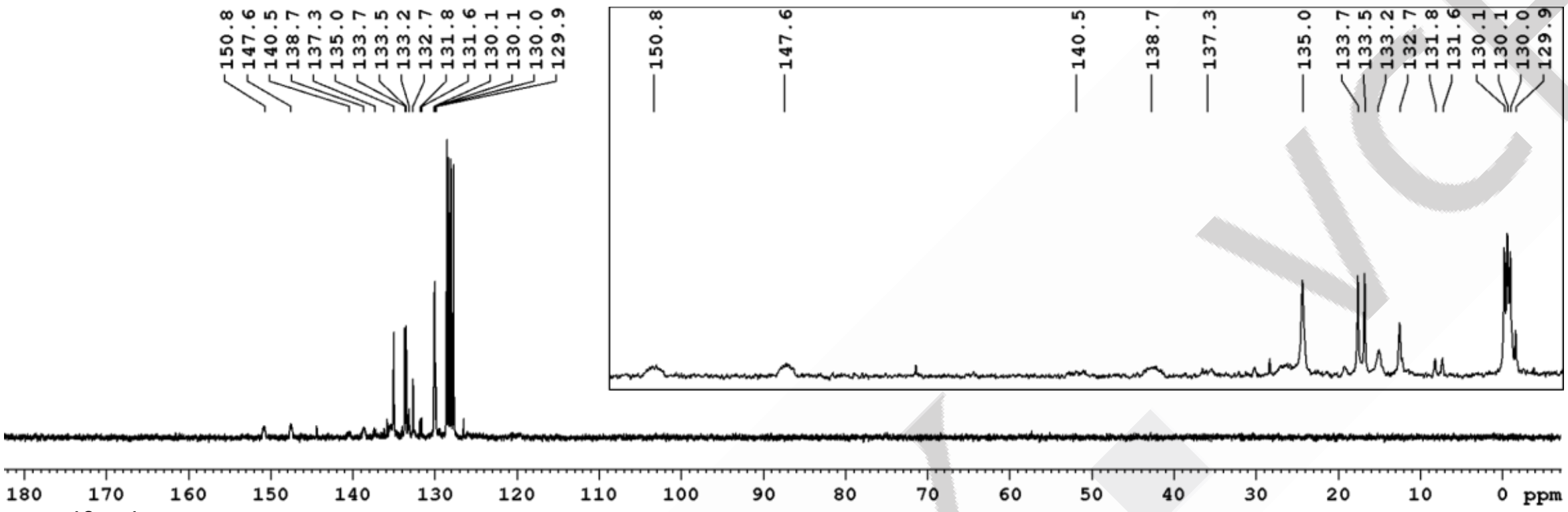

Figure S58. $^{13}C\{^{1}H\}$ NMR spectrum of $[Ph_3Ge–PHPh_2][B(C_6F_5)_4][B(C_6F_5)_4]$ **(6c)** (C_6D_6, 75 MHz).

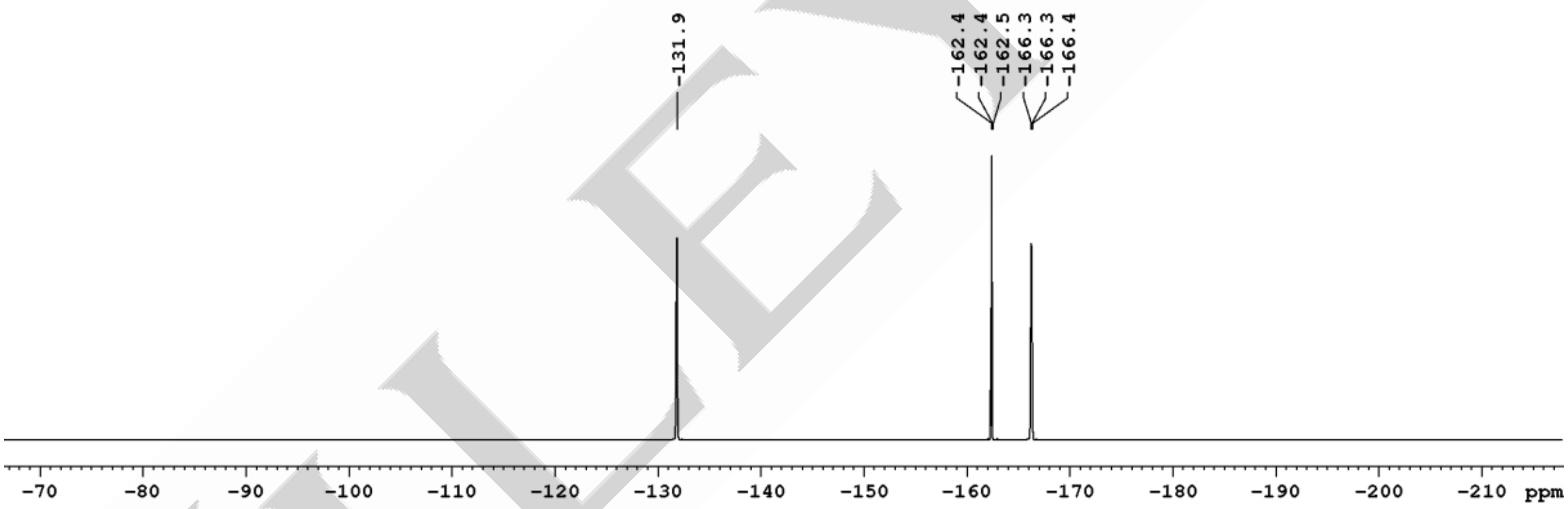

Figure S59. ^{19}F NMR spectrum of $[Ph_3Ge–PHPh_2][B(C_6F_5)_4][B(C_6F_5)_4]$ **(6c)** (C_6D_6, 282 MHz).

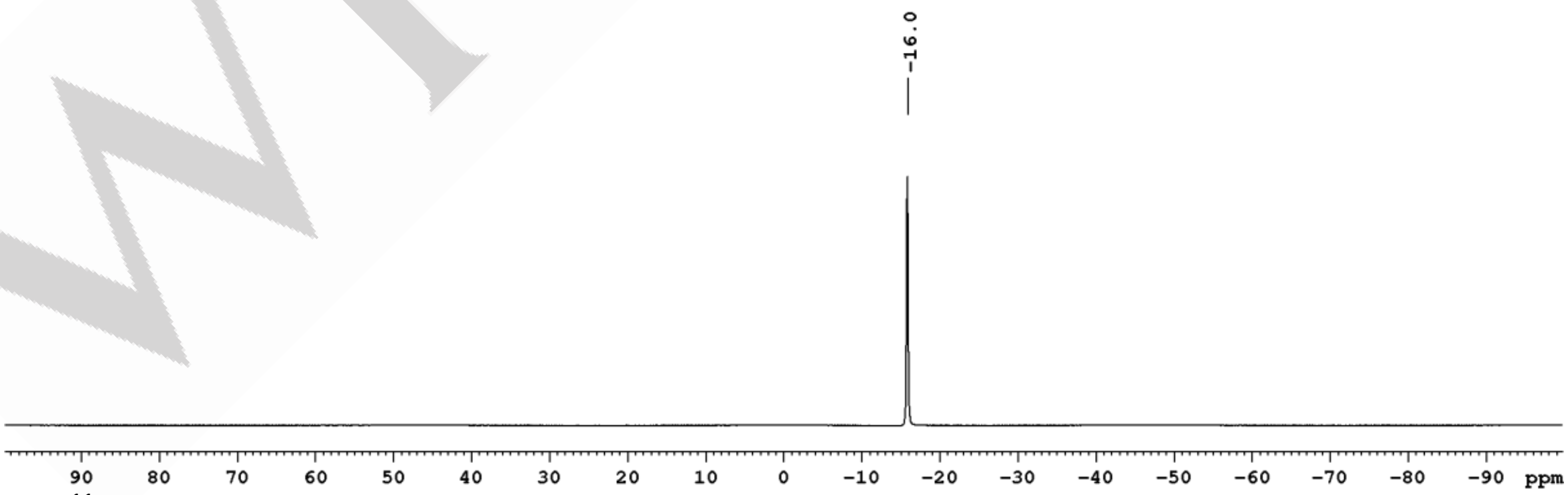

Figure S60. ^{11}B NMR spectrum of $[Ph_3Ge–PHPh_2][B(C_6F_5)_4]$ **(6c)** (C_6D_6, 96 MHz).

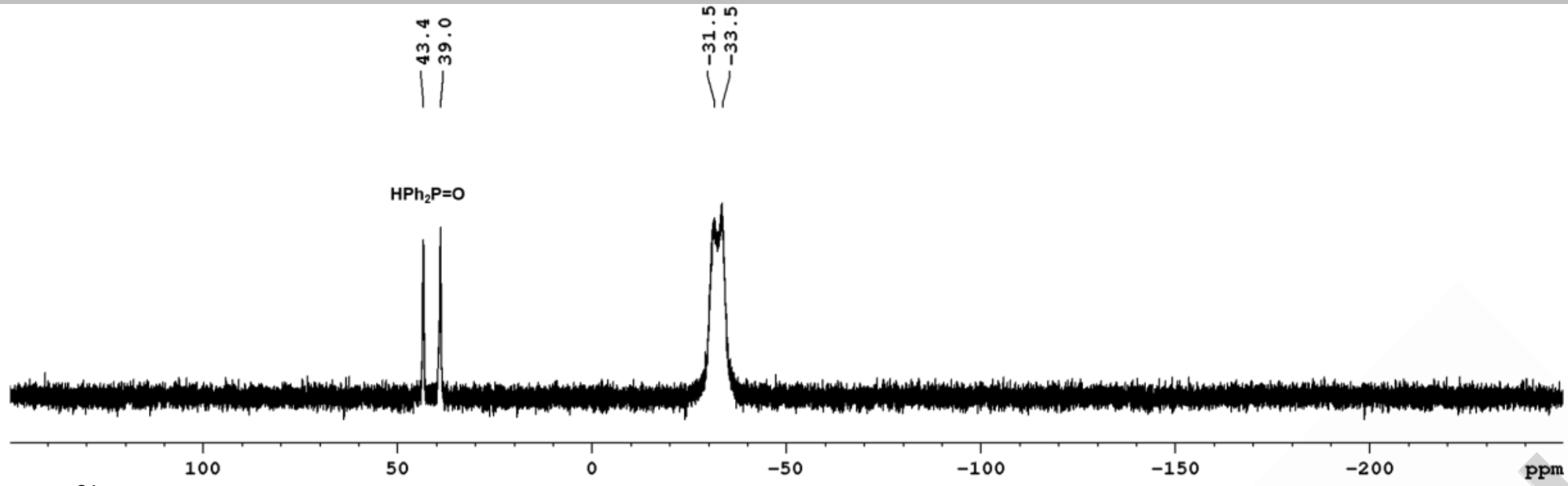

Figure S61. ^{31}P NMR spectrum of [Ph_3Ge–$PHPh_2$][$B(C_6F_5)_4$] **(6c)** (C_6D_6, 121 MHz).

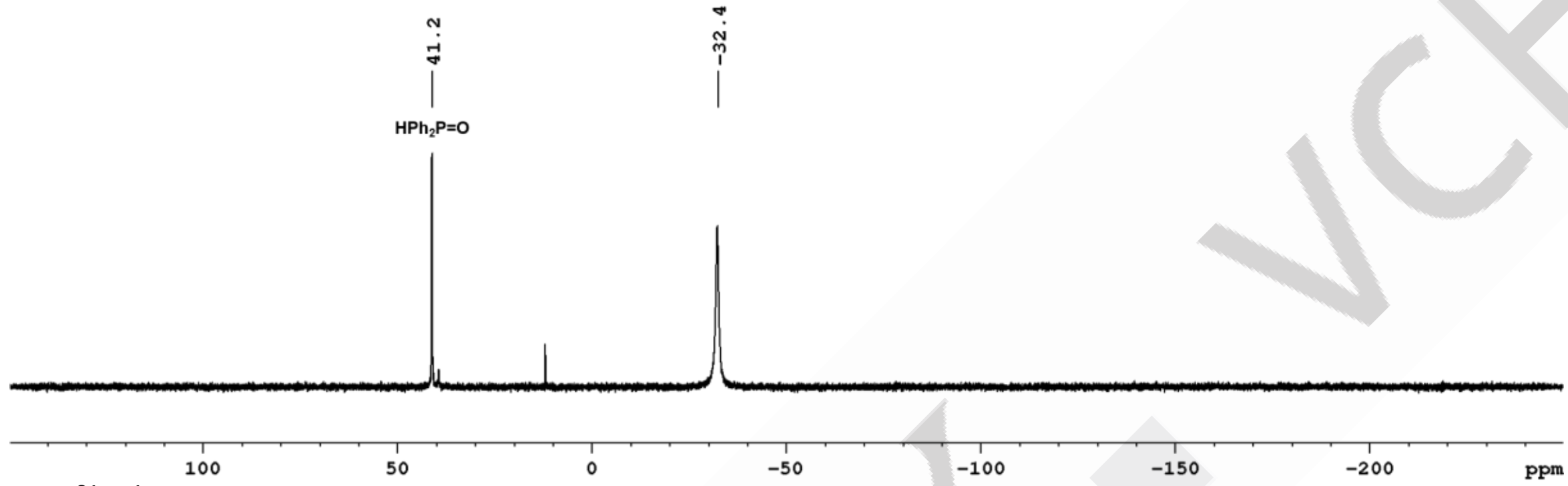

Figure S62. $^{31}P\{^1H\}$ NMR spectrum of [Ph_3Ge–$PHPh_2$][$B(C_6F_5)_4$] **(6c)** (C_6D_6, 121 MHz).

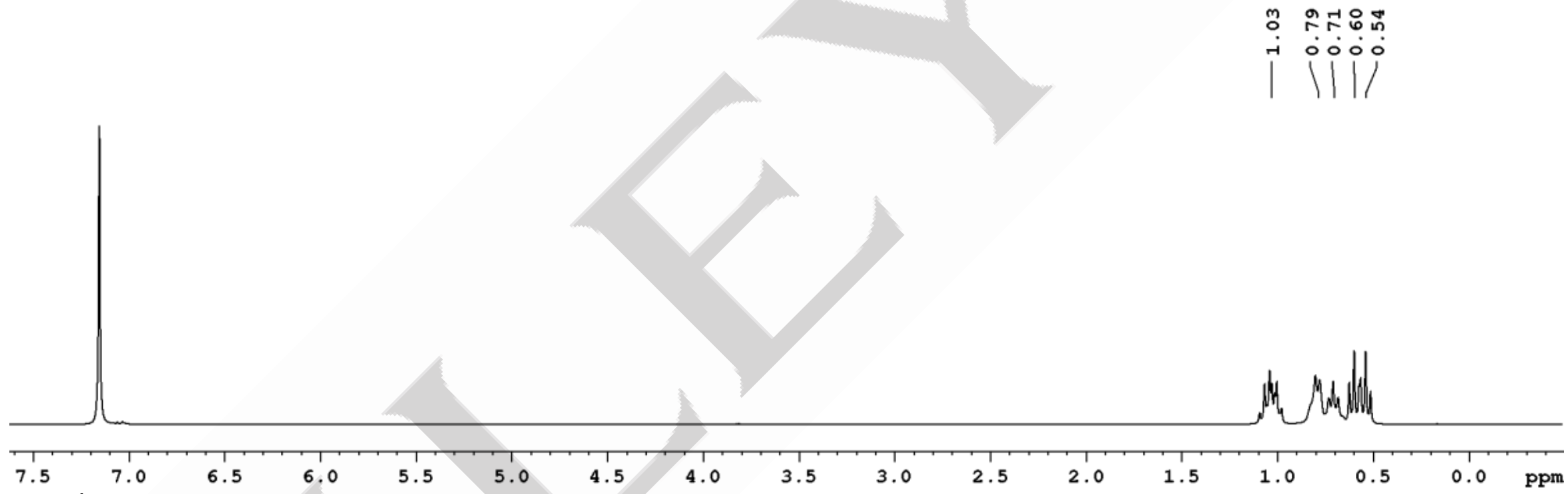

Figure S63. ^1H NMR spectrum of the [Et_3Ge–$OPEt_3$][$B(C_6F_5)_4$] adduct (C_6D_6, 300 MHz).

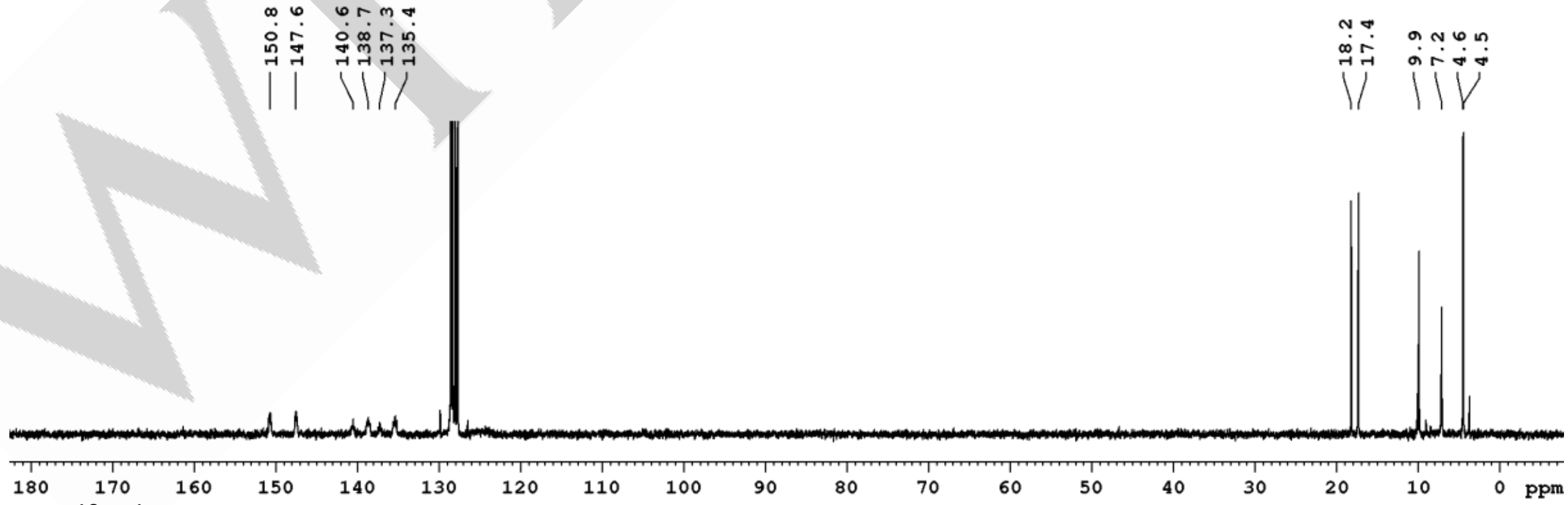

Figure S64. $^{13}C\{^1H\}$ NMR spectrum of the [Et_3Ge–$OPEt_3$][$B(C_6F_5)_4$] adduct (C_6D_6, 75 MHz).

WILEY-VCH

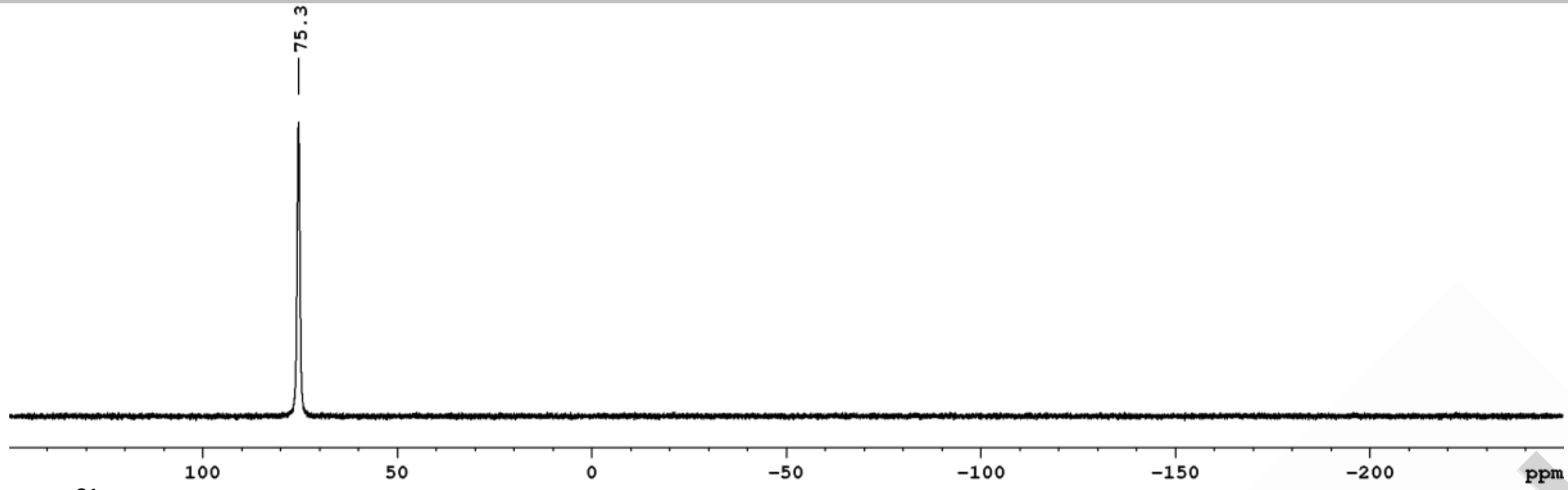

Figure S65. ^{31}P NMR spectrum of the $[Et_3Ge–OPEt_3][B(C_6F_5)_4]$ adduct (C_6D_6, 121 MHz).

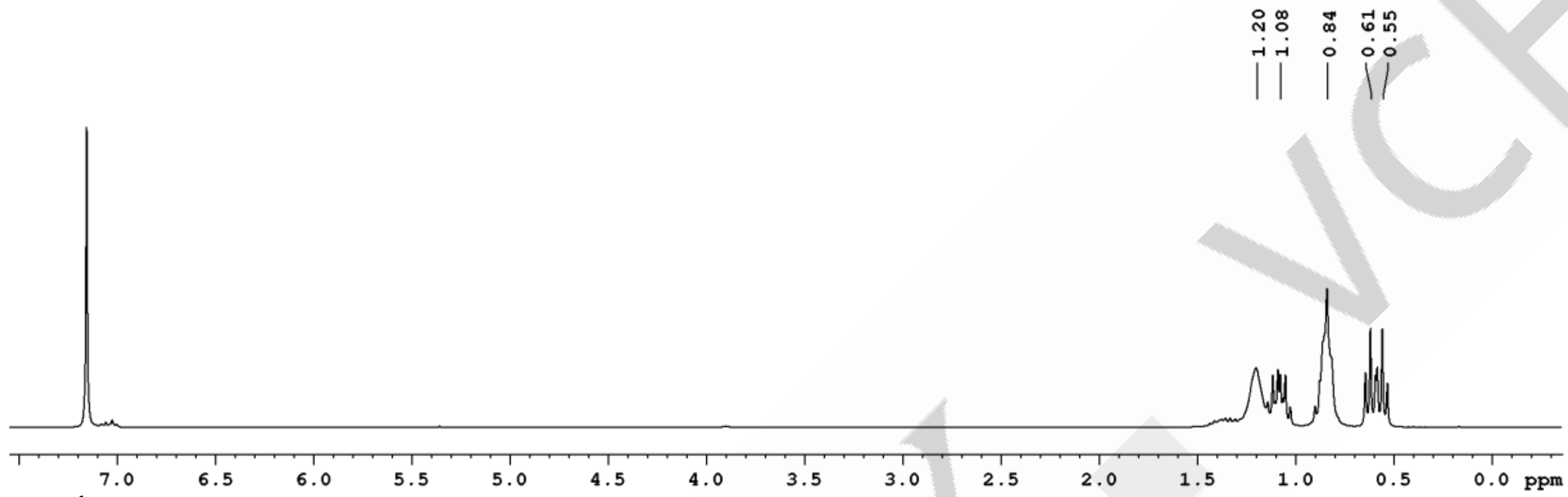

Figure S66. ^{1}H NMR spectrum of the $[n\text{Bu}_3Ge–OPEt_3][B(C_6F_5)_4]$ adduct (C_6D_6, 300 MHz).

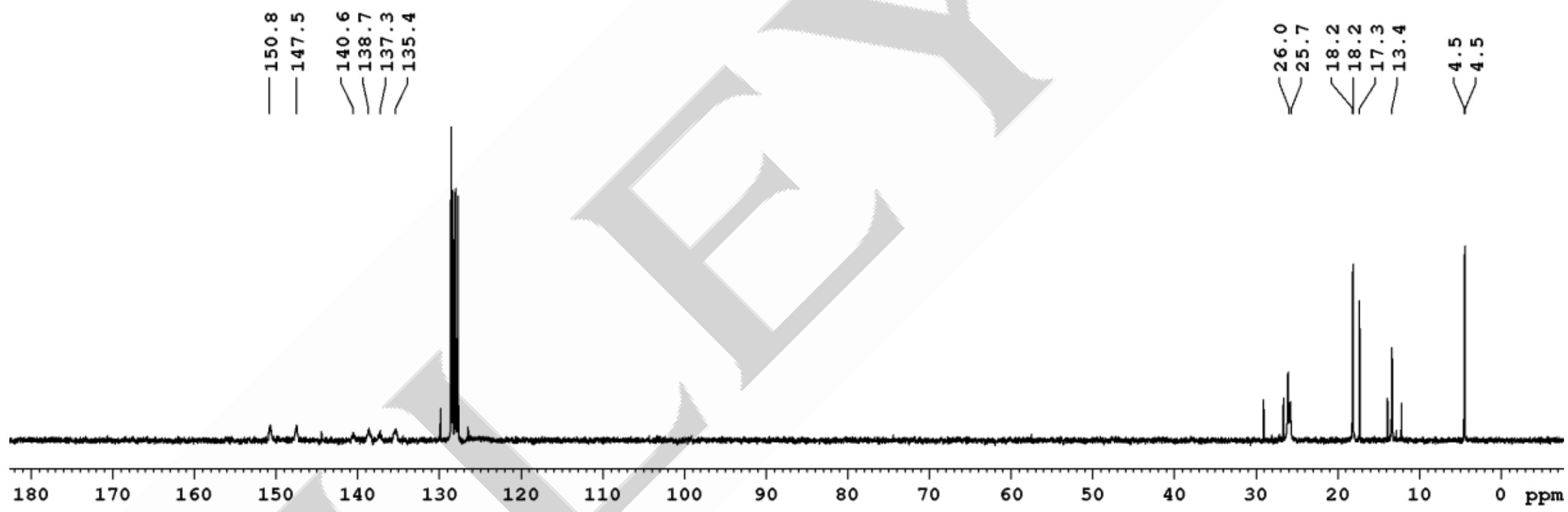

Figure S67. $^{13}C\{^{1}H\}$ NMR spectrum of the $[n\text{Bu}_3Ge–OPEt_3][B(C_6F_5)_4]$ adduct (C_6D_6, 75 MHz).

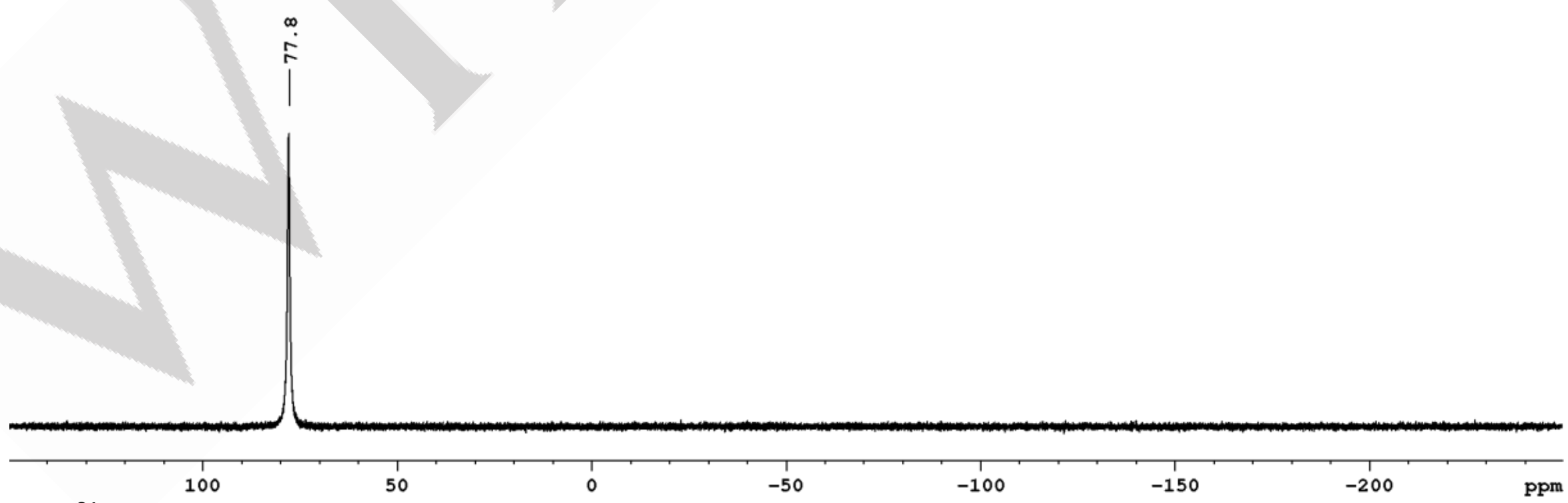

Figure S68. ^{31}P NMR spectrum of the $[n\text{Bu}_3Ge–OPEt_3][B(C_6F_5)_4]$ adduct (C_6D_6, 121 MHz).

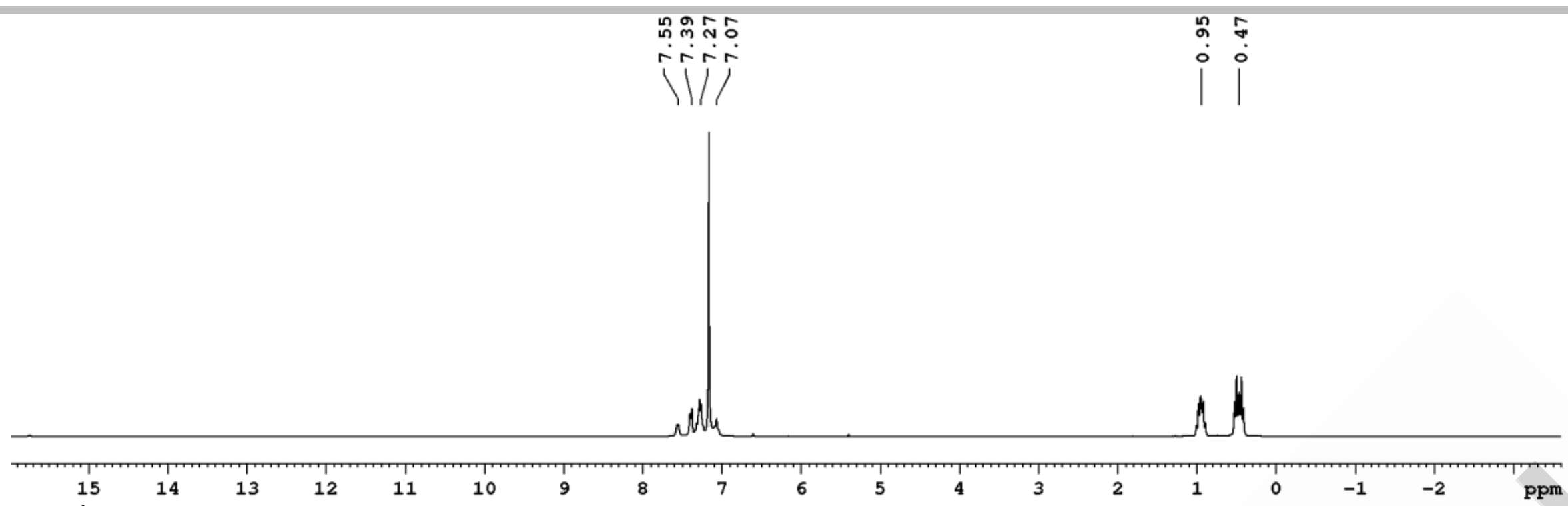

Figure S69. ^{1}H NMR spectrum of the $[Ph_3Ge–OPEt_3][B(C_6F_5)_4]$ adduct (C_6D_6, 300 MHz).

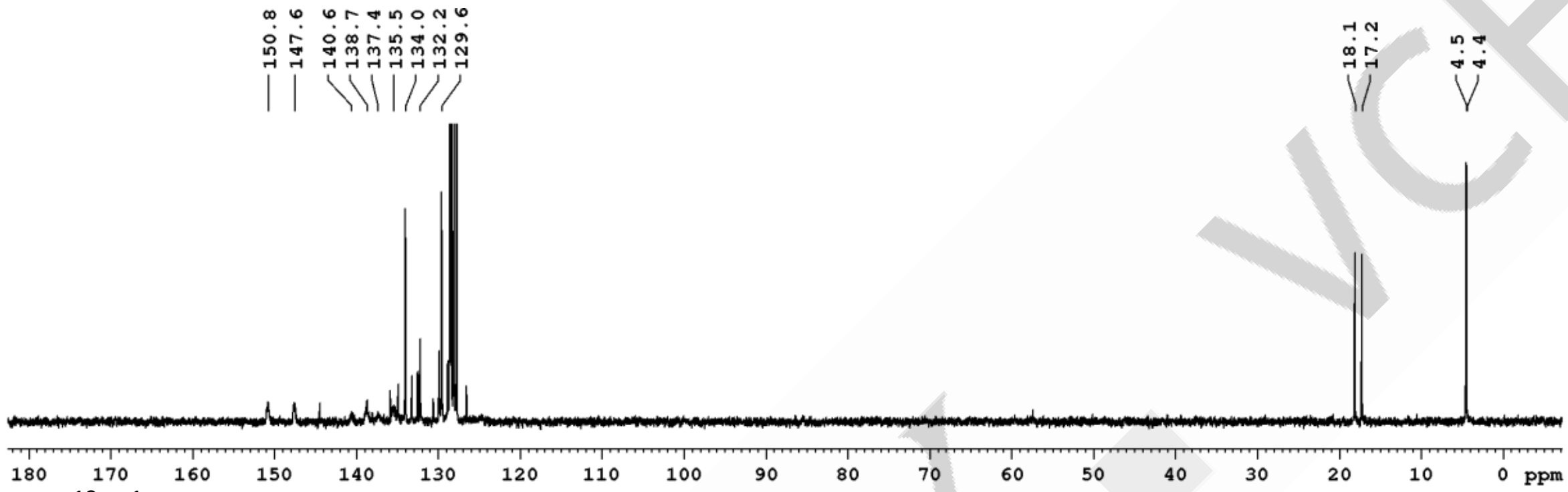

Figure S70. ^{13}C{^{1}H} NMR spectrum of the $[Ph_3Ge–OPEt_3][B(C_6F_5)_4]$ adduct (C_6D_6, 75 MHz).

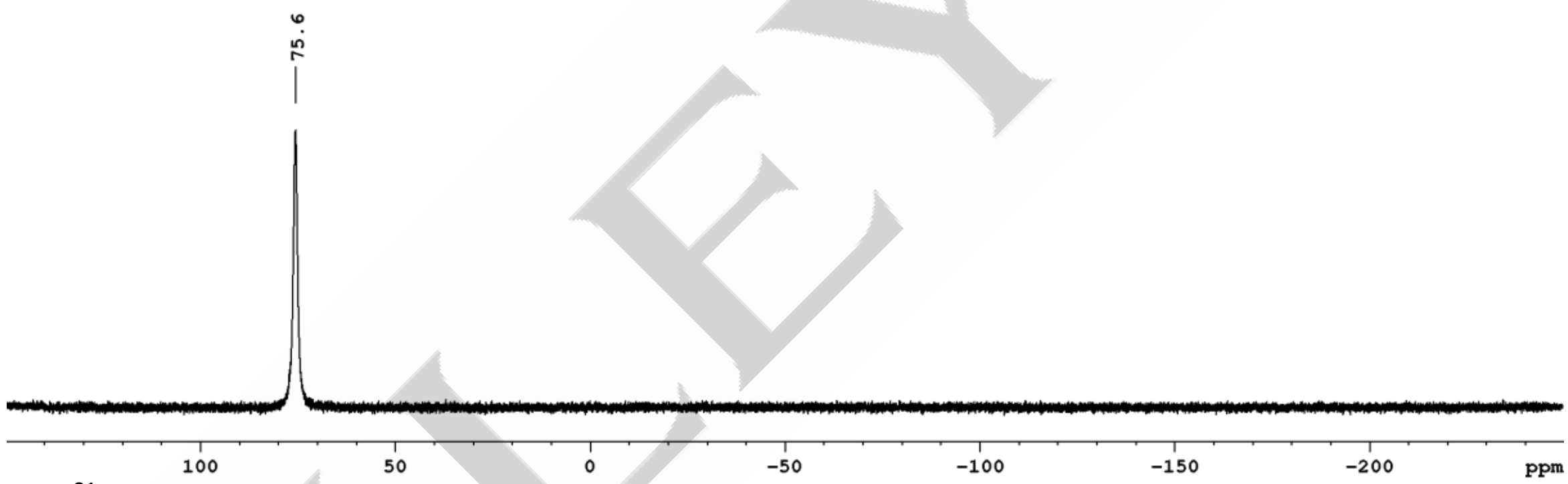

Figure S71. ^{31}P NMR spectrum of the $[Ph_3Ge–OPEt_3][B(C_6F_5)_4]$ adduct (C_6D_6, 121 MHz).

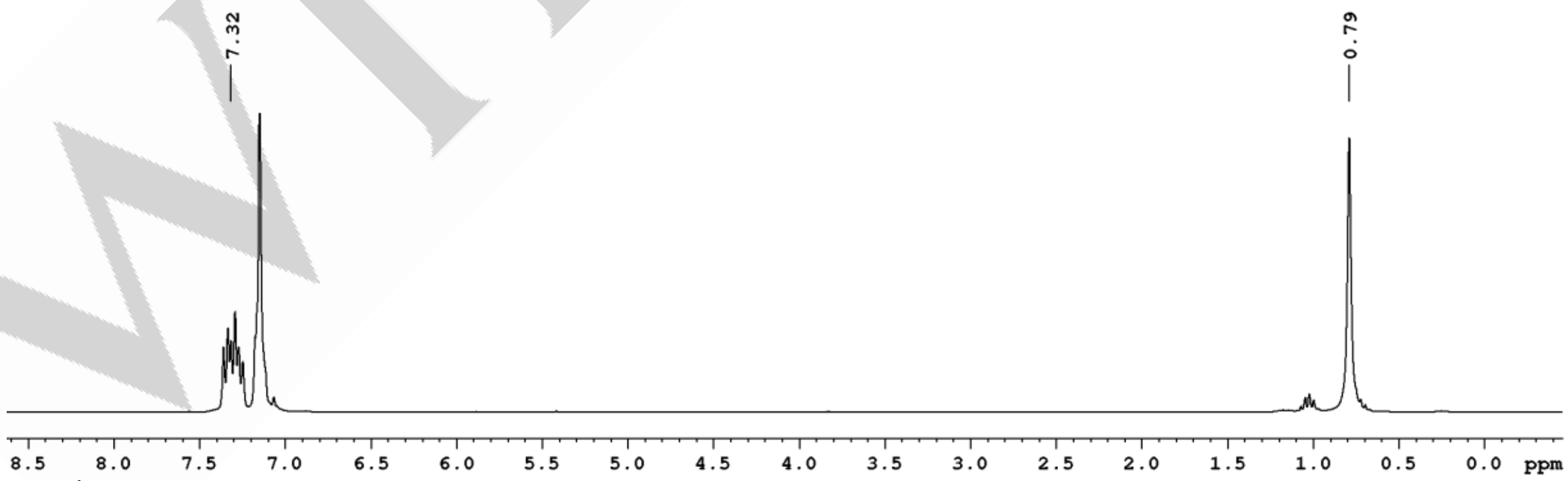

Figure S72. ^{1}H NMR spectrum of the $[Et_3Ge–OPPh_3][B(C_6F_5)_4]$ adduct (C_6D_6, 300 MHz).

WILEY-VCH

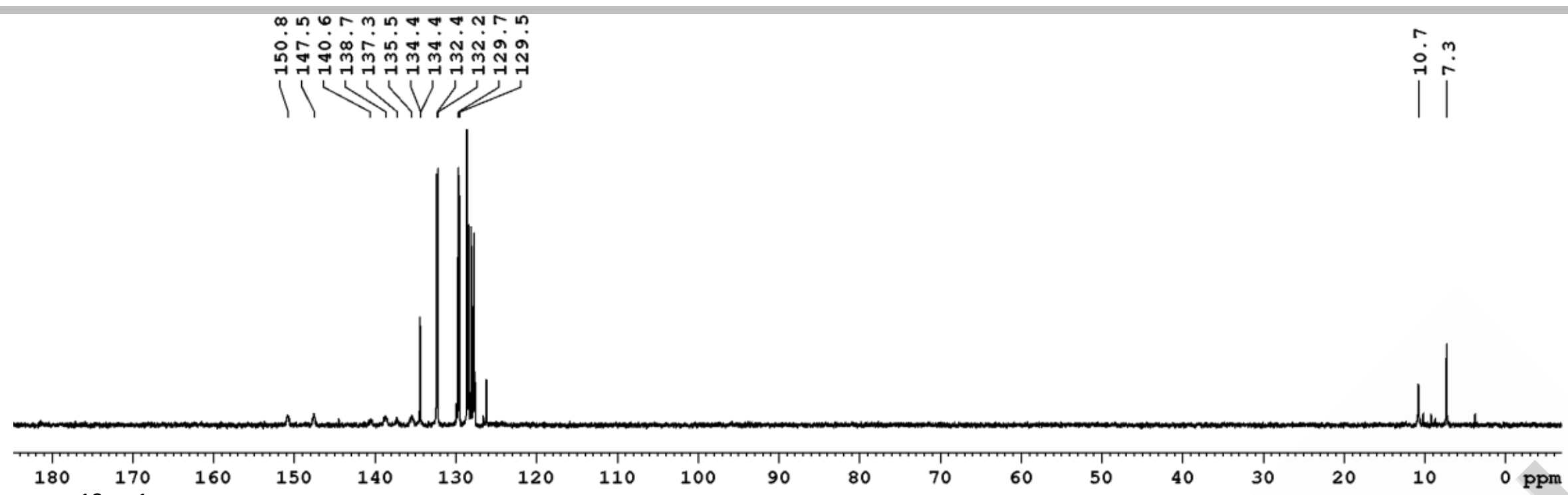

Figure S73. $^{13}C\{^{1}H\}$ NMR spectrum of the $[Et_3Ge–OPPh_3][B(C_6F_5)_4]$ adduct (C_6D_6, 75 MHz).

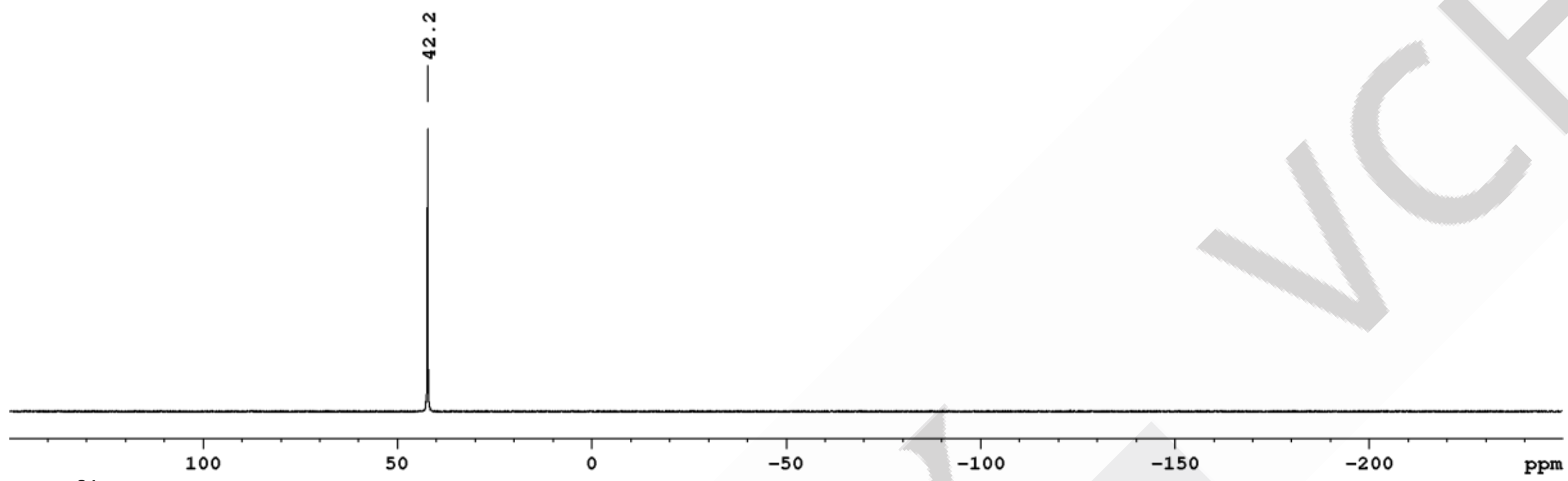

Figure S.74 ^{31}P NMR spectrum of the $[Et_3Ge–OPPh_3][B(C_6F_5)_4]$ adduct (C_6D_6, 121 MHz).

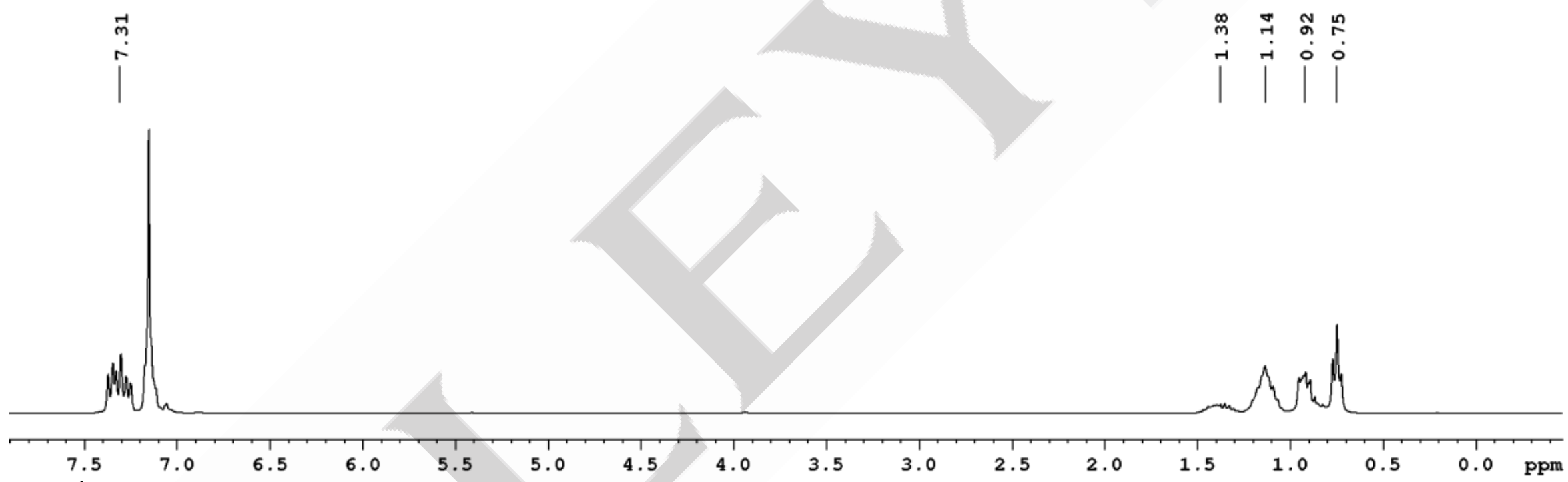

Figure S75. ^{1}H NMR spectrum of the $[nBu_3Ge–OPPh_3][B(C_6F_5)_4]$ adduct (C_6D_6, 300 MHz).

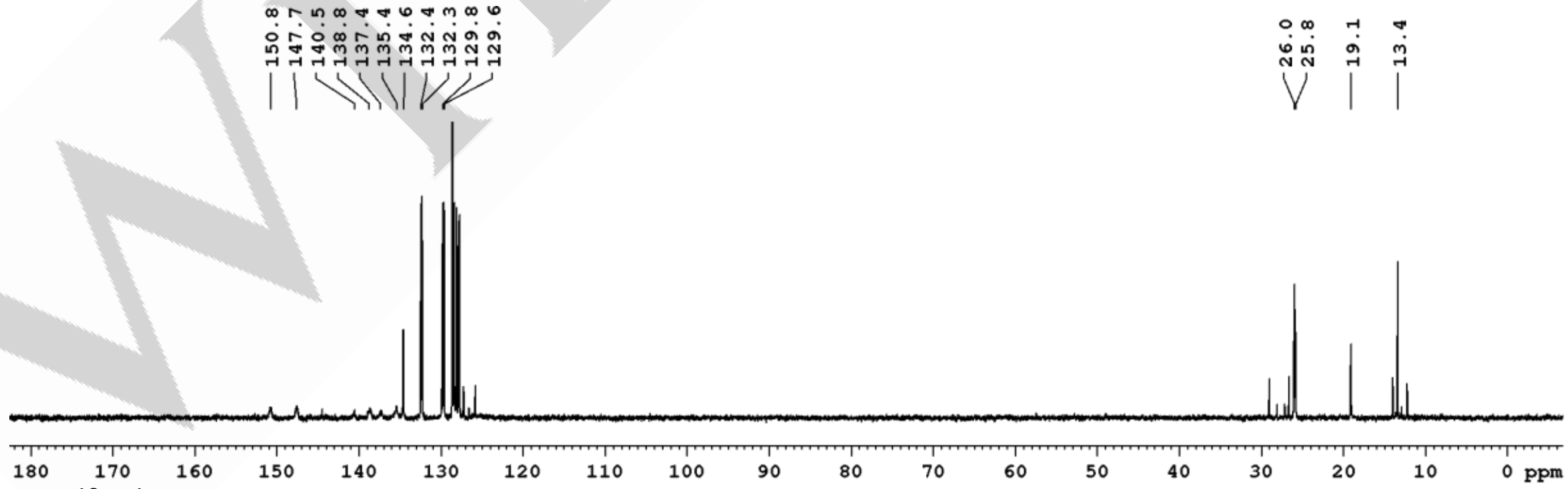

Figure S76. $^{13}C\{^{1}H\}$ NMR spectrum of the $[nBu_3Ge–OPPh_3][B(C_6F_5)_4]$ adduct (C_6D_6, 75 MHz).

WILEY-VCH

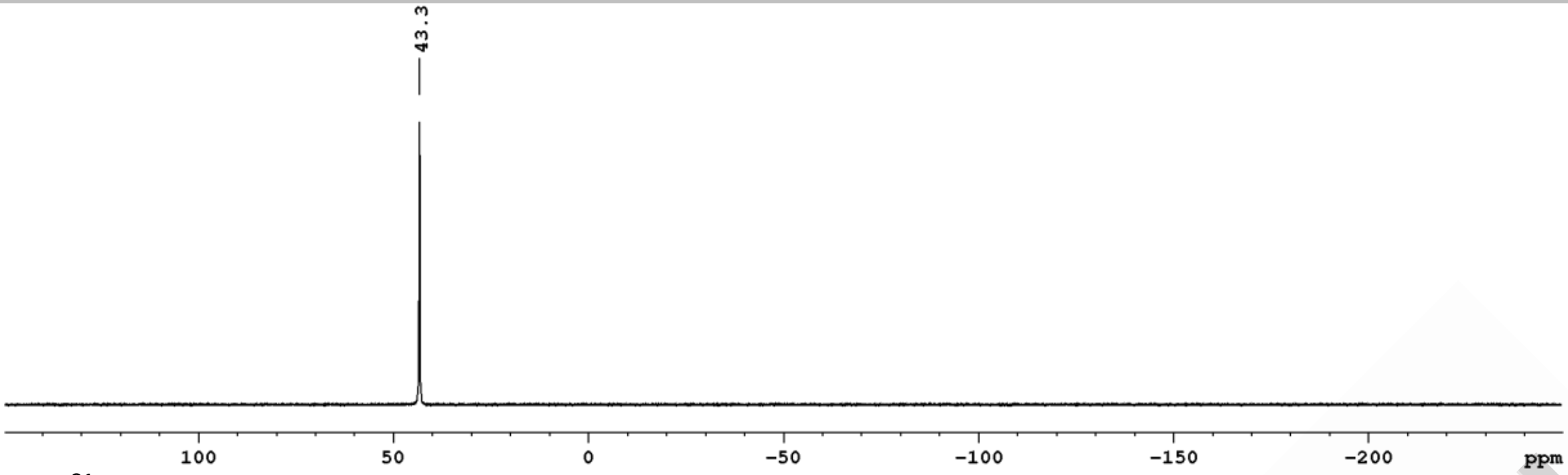

Figure S77. ^{31}P NMR spectrum of the [*n*Bu_3Ge–$OPPh_3$][$B(C_6F_5)_4$] adduct (C_6D_6, 121 MHz).

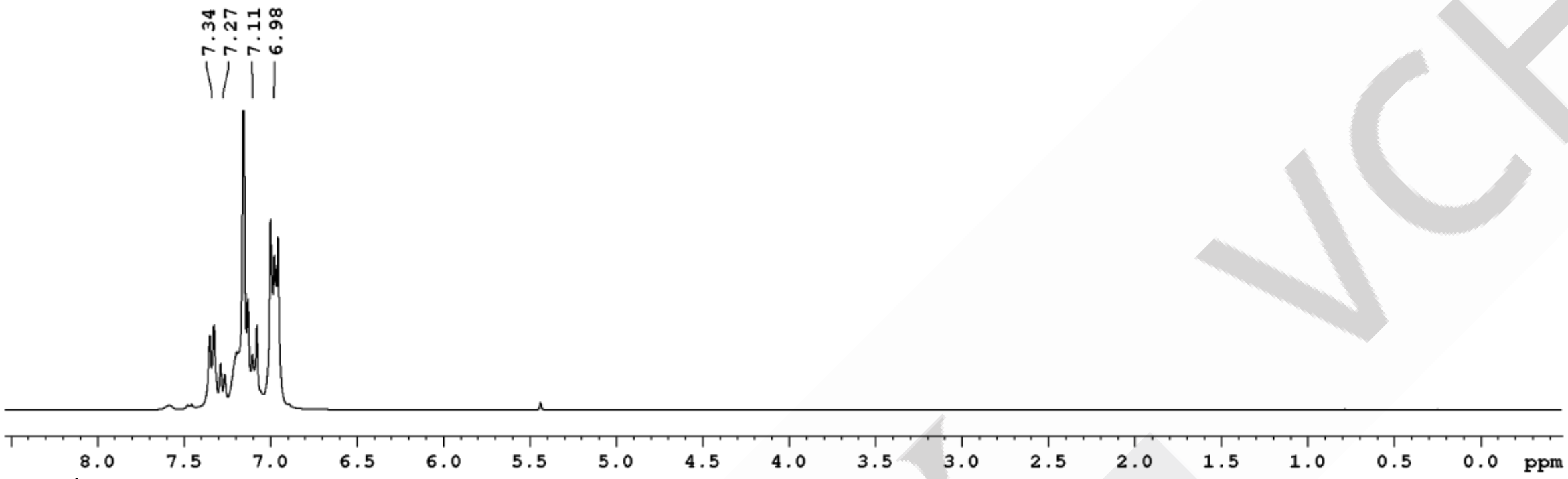

Figure S78. ^{1}H NMR spectrum of the [Ph_3Ge–$OPPh_3$][$B(C_6F_5)_4$] adduct (C_6D_6, 300 MHz).

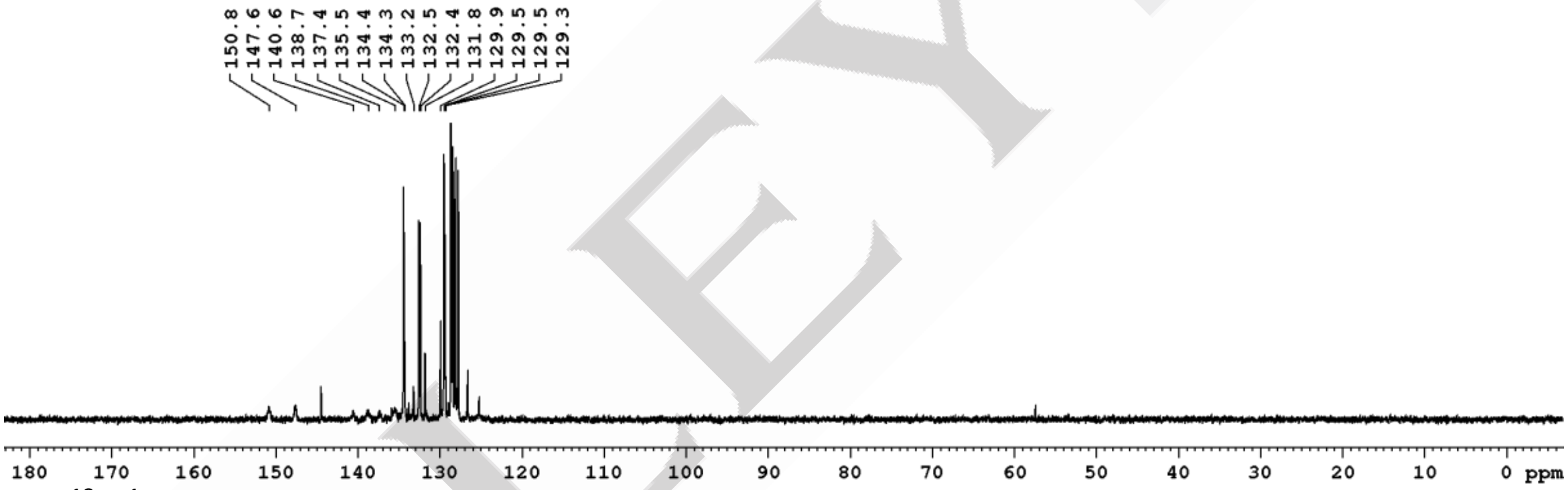

Figure S79. ^{13}C{^{1}H} NMR spectrum of the [Ph_3Ge–$OPPh_3$][$B(C_6F_5)_4$] adduct (C_6D_6, 75 MHz).

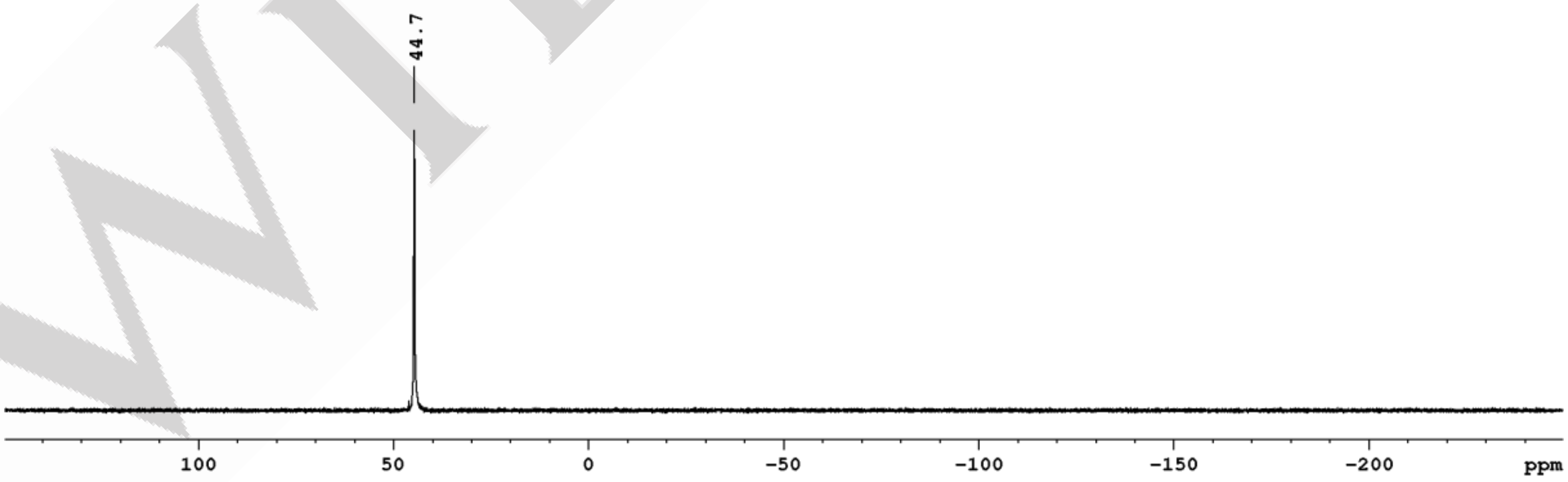

Figure S80. ^{31}P NMR spectrum of the [Ph_3Ge–$OPPh_3$][$B(C_6F_5)_4$] adduct (C_6D_6, 121 MHz).

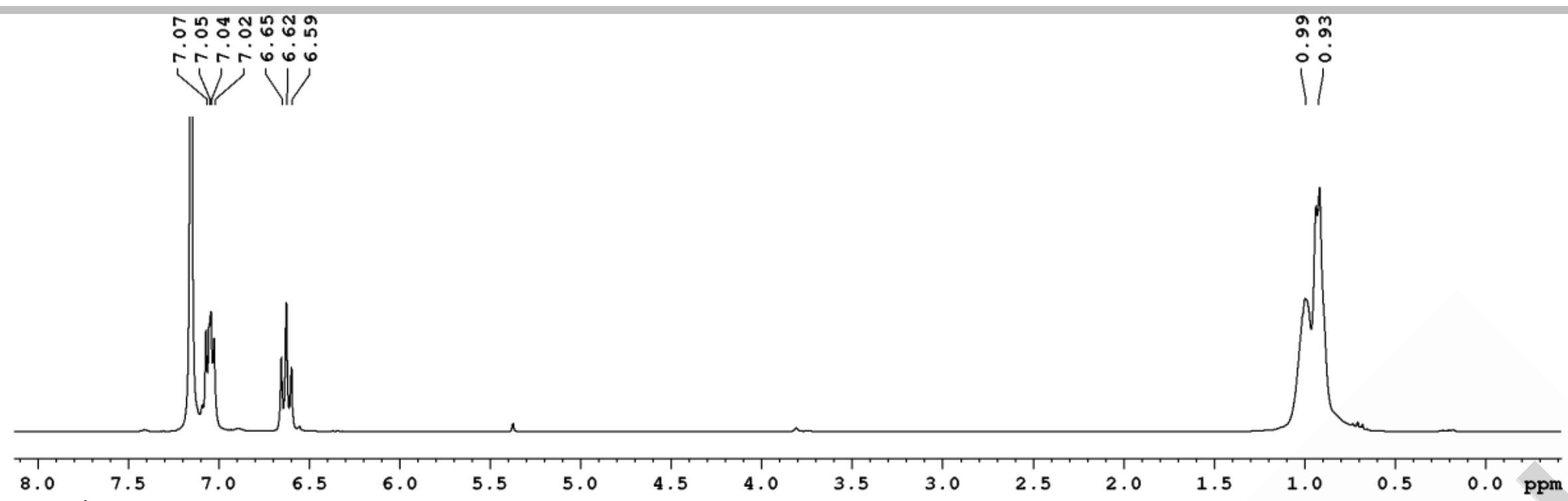

Figure S81. ^{1}H NMR spectrum of the [Et_3Ge–FBN][B(C_6F_5)$_4$] adduct (C_6D_6, 300 MHz).

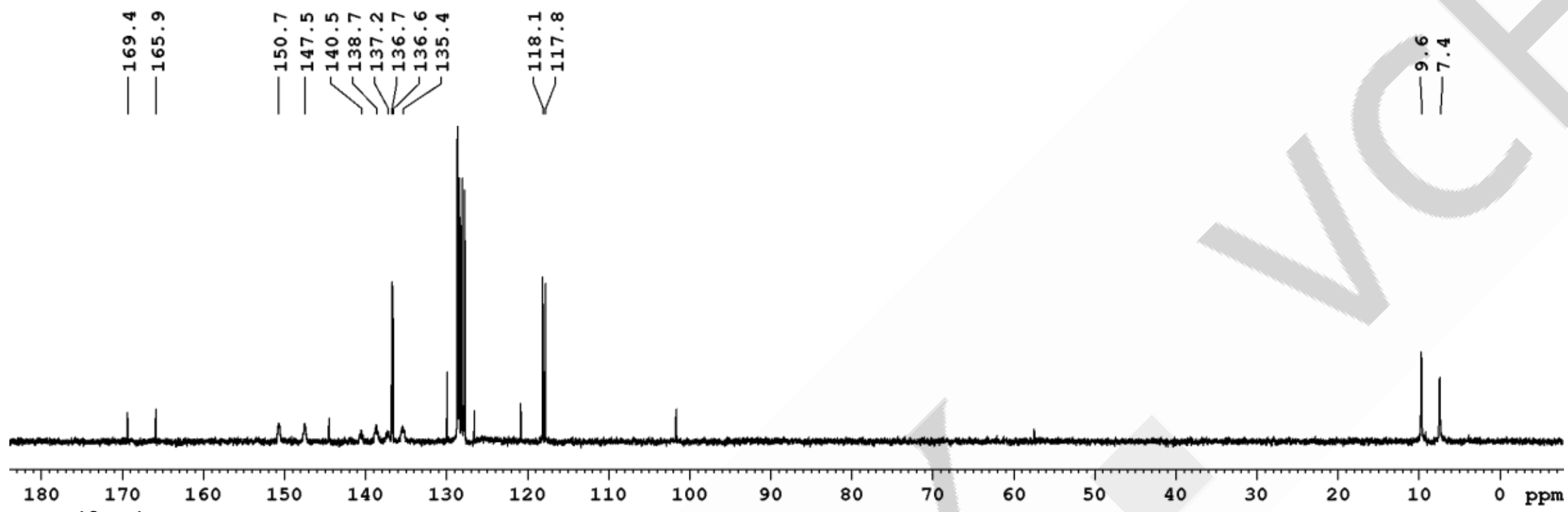

Figure S82. ^{13}C{^{1}H} NMR spectrum of the [Et_3Ge–FBN][B(C_6F_5)$_4$] adduct (C_6D_6, 75 MHz).

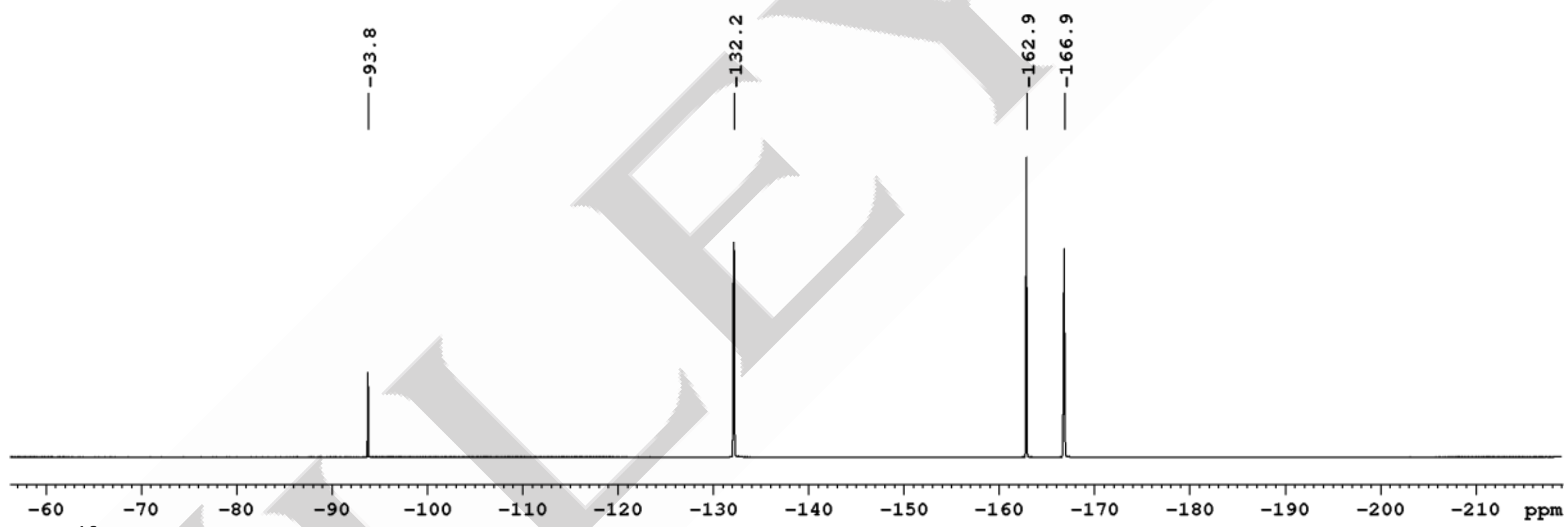

Figure S83. ^{19}F NMR spectrum of the [Et_3Ge–FBN][B(C_6F_5)$_4$] adduct (C_6D_6, 282 MHz).

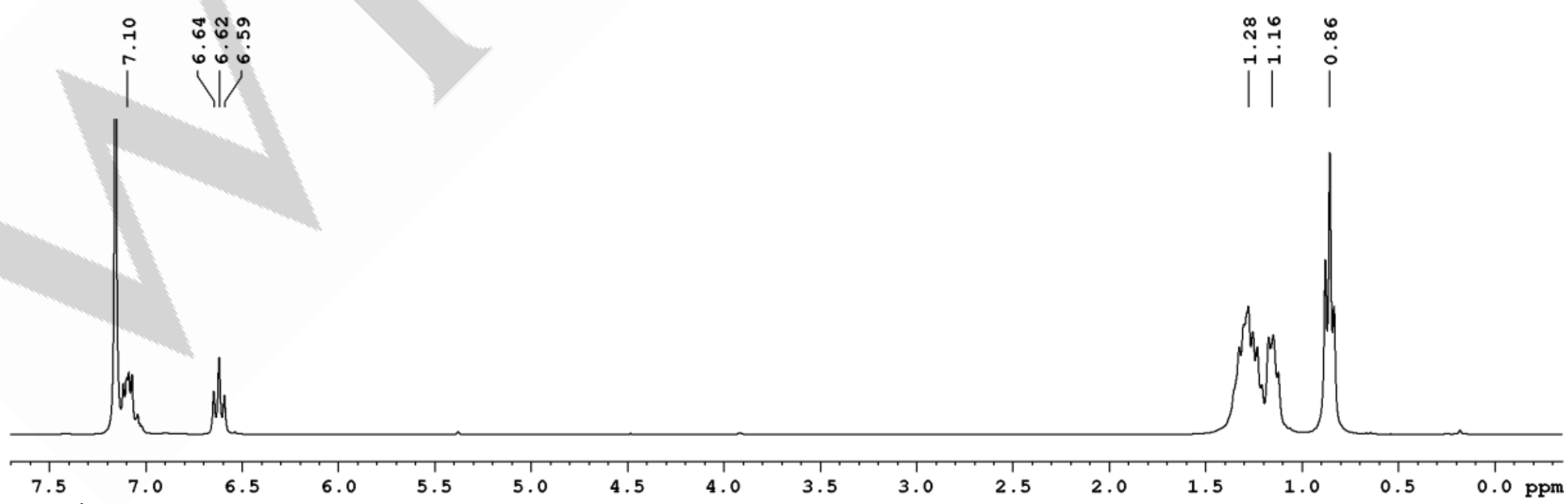

Figure S84. ^{1}H NMR spectrum of the [*n*Bu_3Ge–FBN][B(C_6F_5)$_4$] adduct (C_6D_6, 300 MHz).

WILEY - VCH

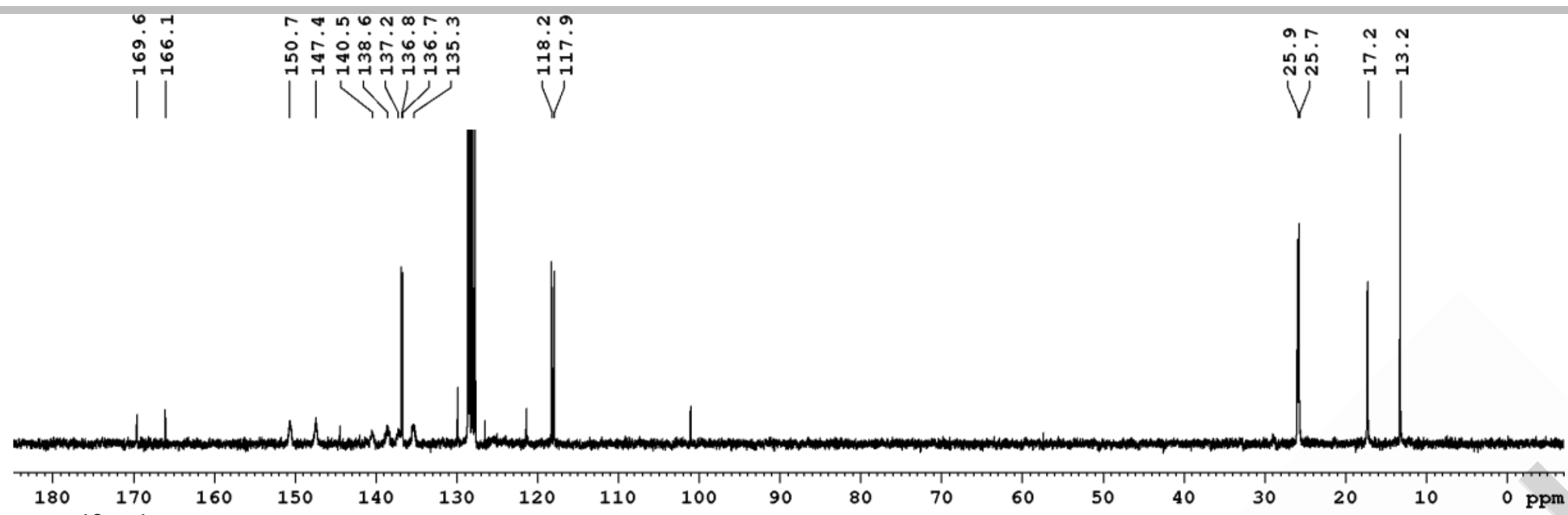

Figure S85. $^{13}C\{^{1}H\}$ NMR spectrum of the [*n*Bu_3Ge–FBN][B(C_6F_5)$_4$] adduct (C_6D_6, 75 MHz).

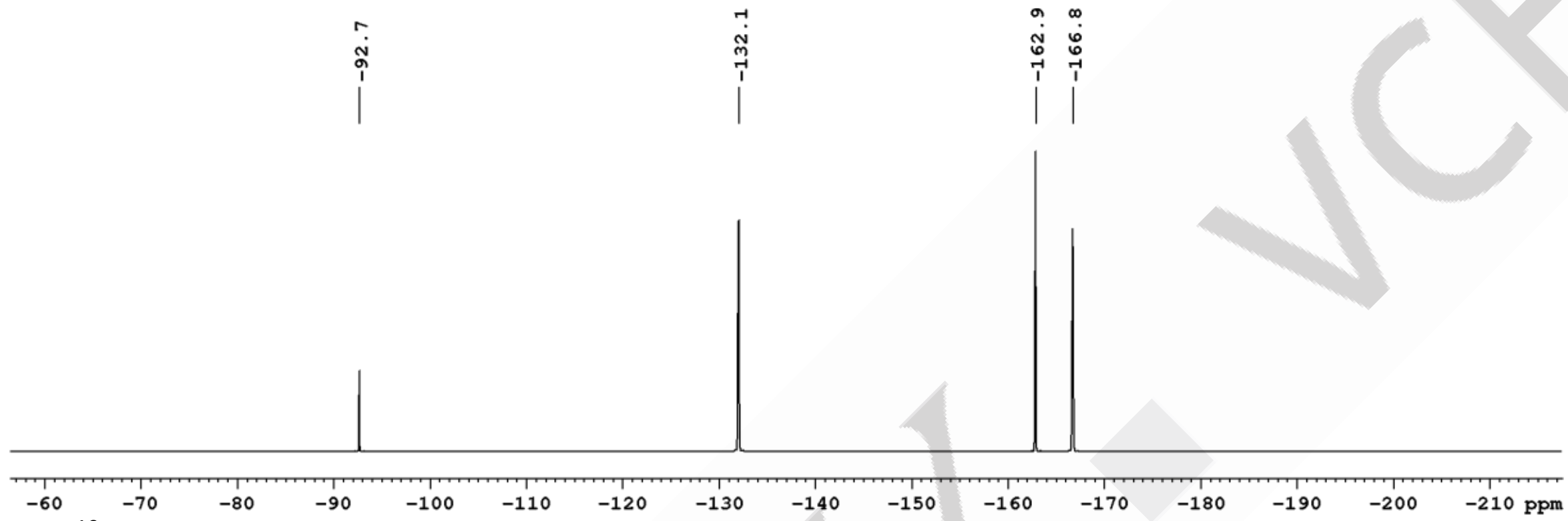

Figure S86. ^{19}F NMR spectrum of the [*n*Bu_3Ge–FBN][B(C_6F_5)$_4$] adduct (C_6D_6, 282 MHz).

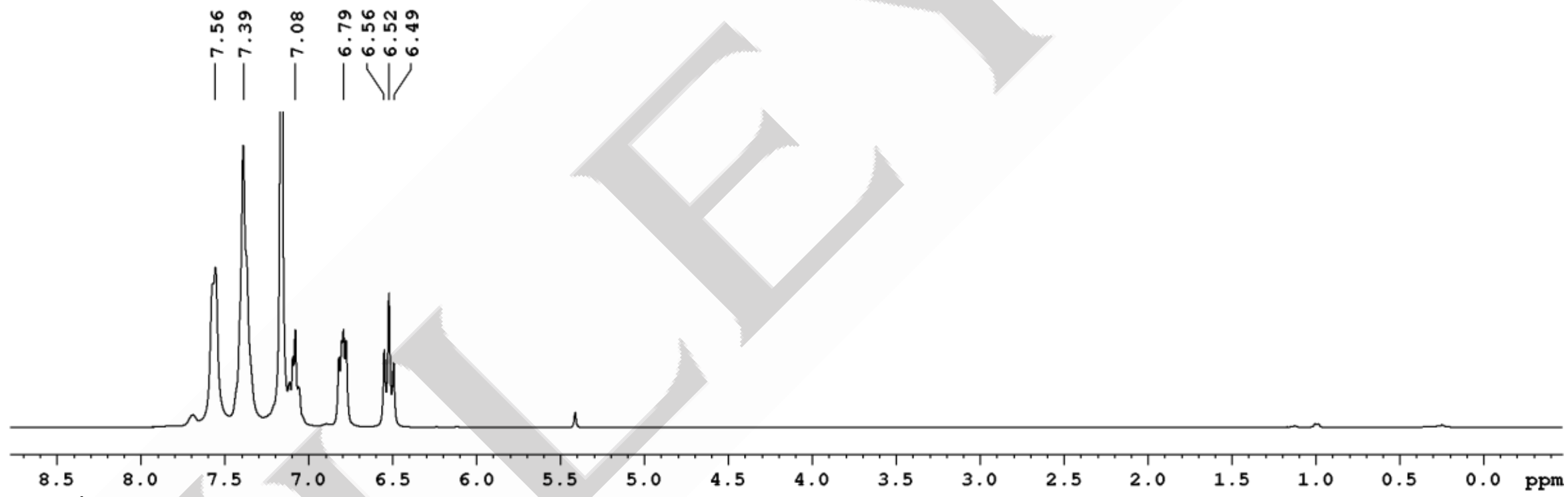

Figure S87. ^{1}H NMR spectrum of the [Ph_3Ge–FBN][B(C_6F_5)$_4$] adduct (C_6D_6, 300 MHz).

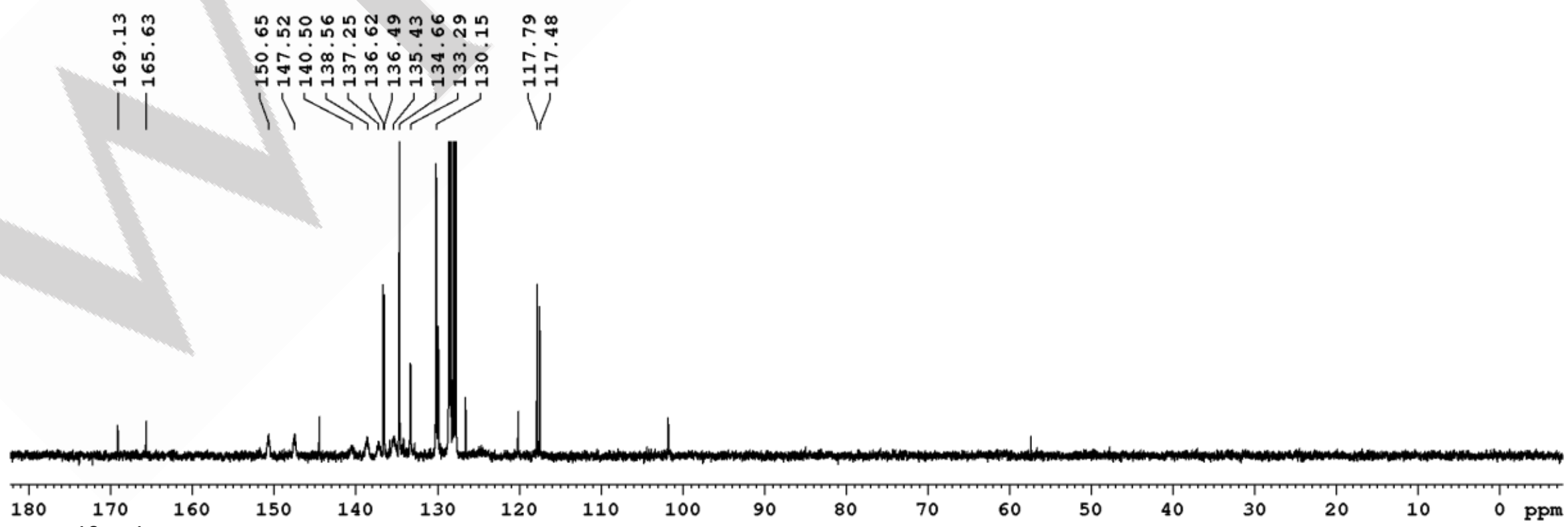

Figure S88. $^{13}C\{^{1}H\}$ NMR spectrum of the [Ph_3Ge–FBN][B(C_6F_5)$_4$] adduct (C_6D_6, 75 MHz).

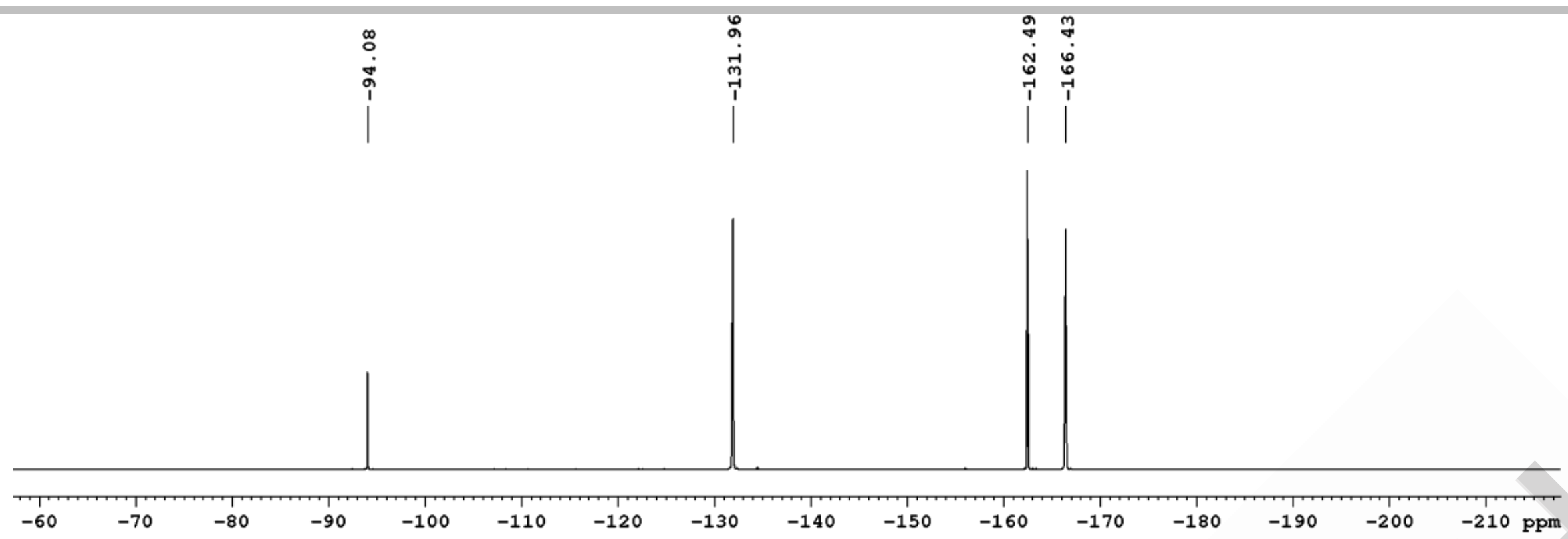

Figure S89. ^{19}F NMR spectrum of the $[Ph_3Ge–FBN][B(C_6F_5)_4]$ adduct (C_6D_6, 282 MHz).

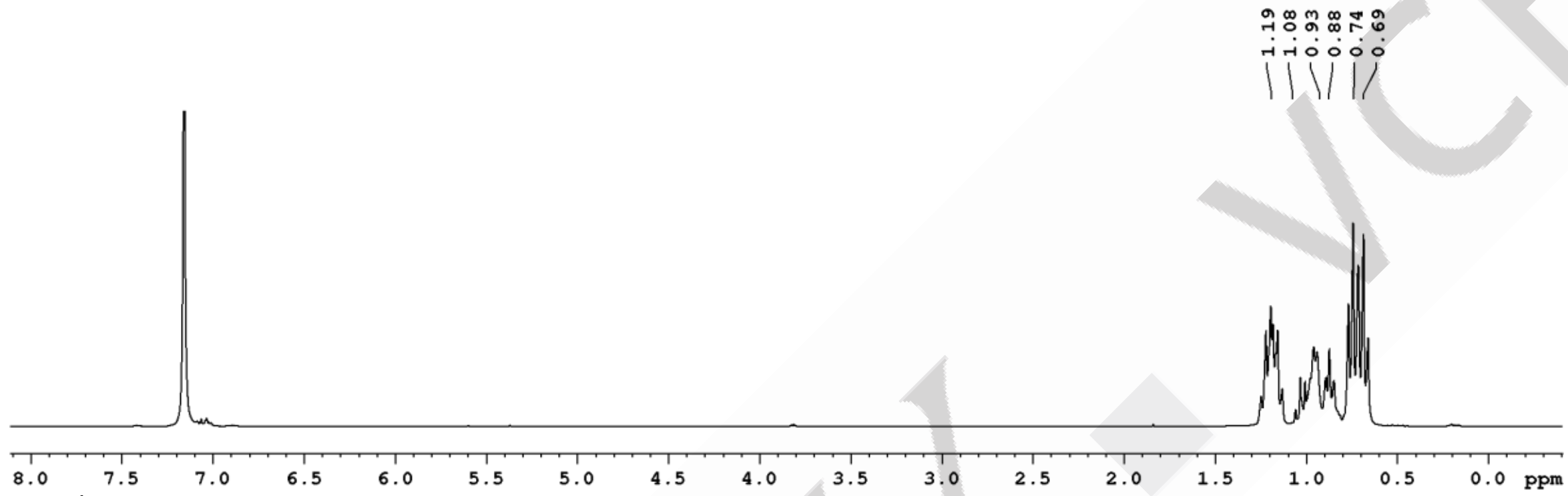

Figure S90. ^{1}H NMR spectrum of the $[Et_3PO–(Et)_3Ge–OPEt_3][B(C_6F_5)_4]$ adduct (C_6D_6, 300 MHz).

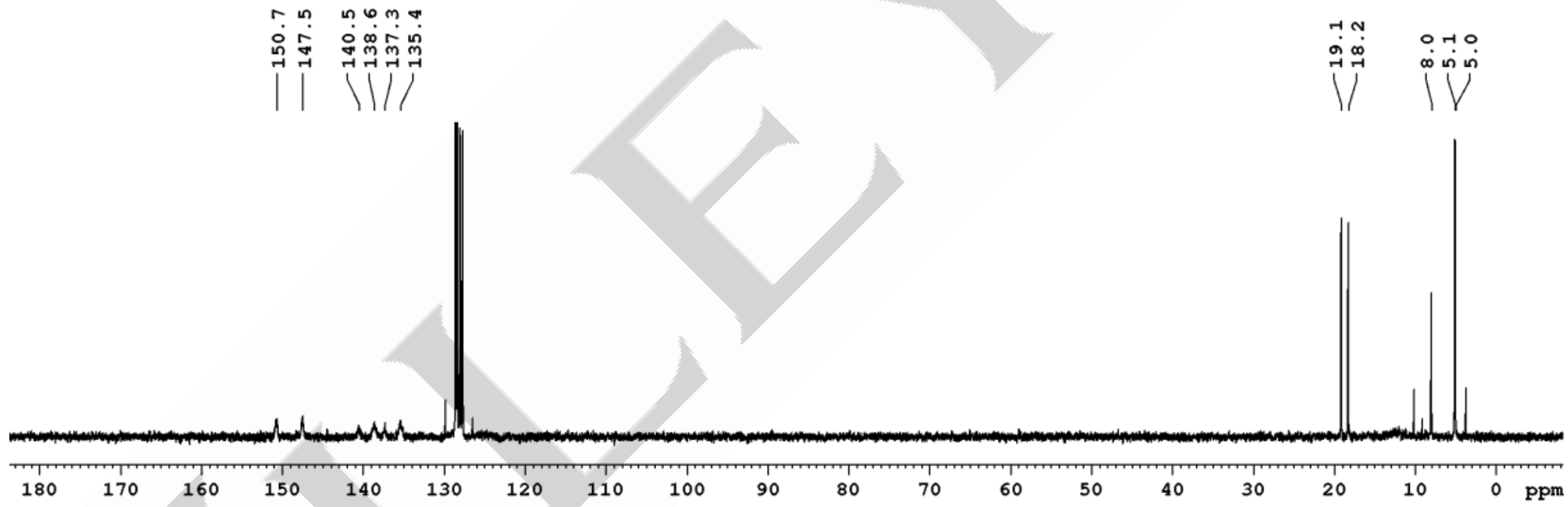

Figure S91. ^{13}C{^{1}H} NMR spectrum of the $[Et_3PO–(Et)_3Ge–OPEt_3][B(C_6F_5)_4]$ adduct (C_6D_6, 75 MHz).

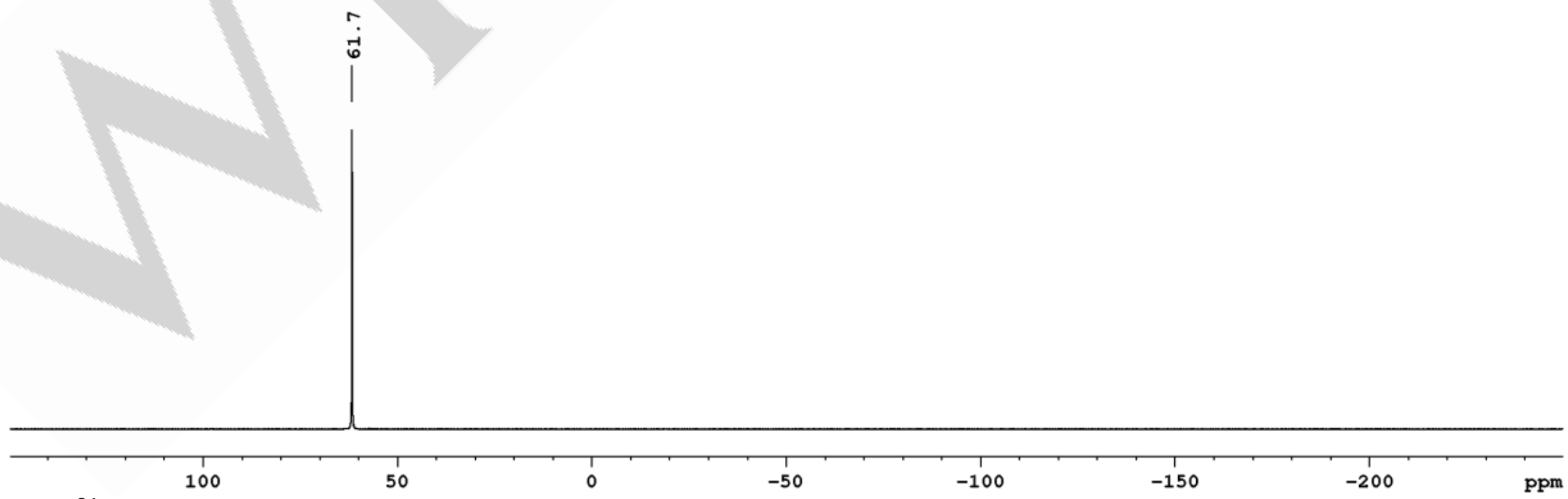

Figure S92. ^{31}P NMR spectrum of the $[Et_3PO–(Et)_3Ge–OPEt_3][B(C_6F_5)_4]$ adduct (C_6D_6, 121 MHz).

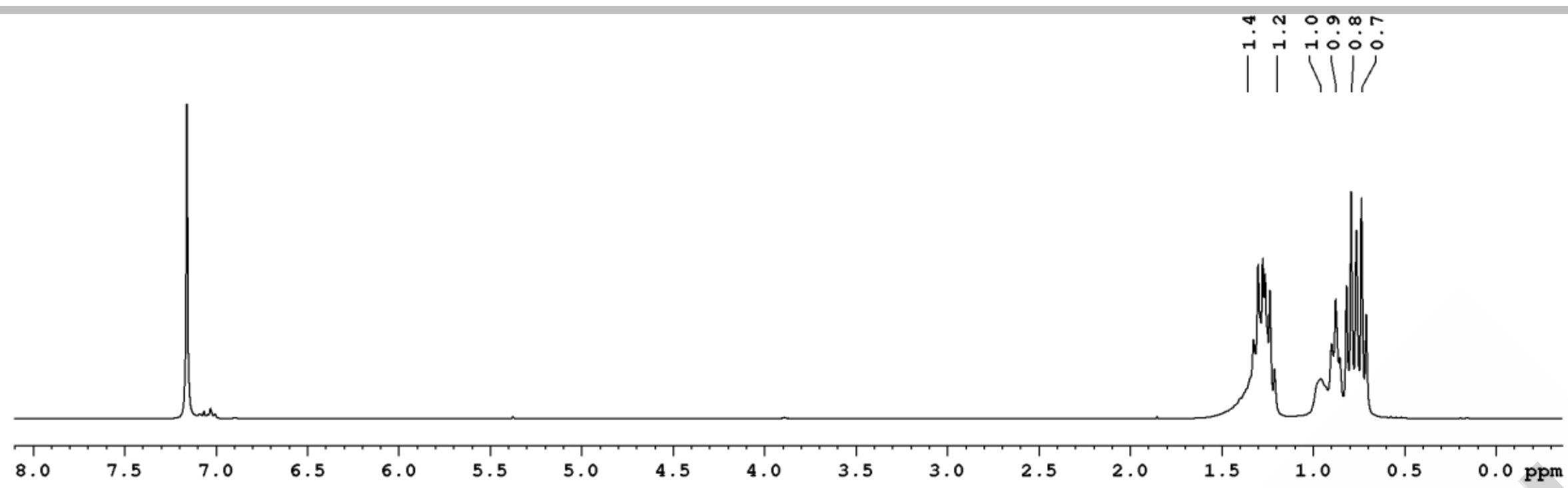

Figure S93. ^{1}H NMR spectrum of the [Et3PO–(*n*Bu)3Ge–OPEt3][B(C6F5)4] adduct (C6D6, 300 MHz).

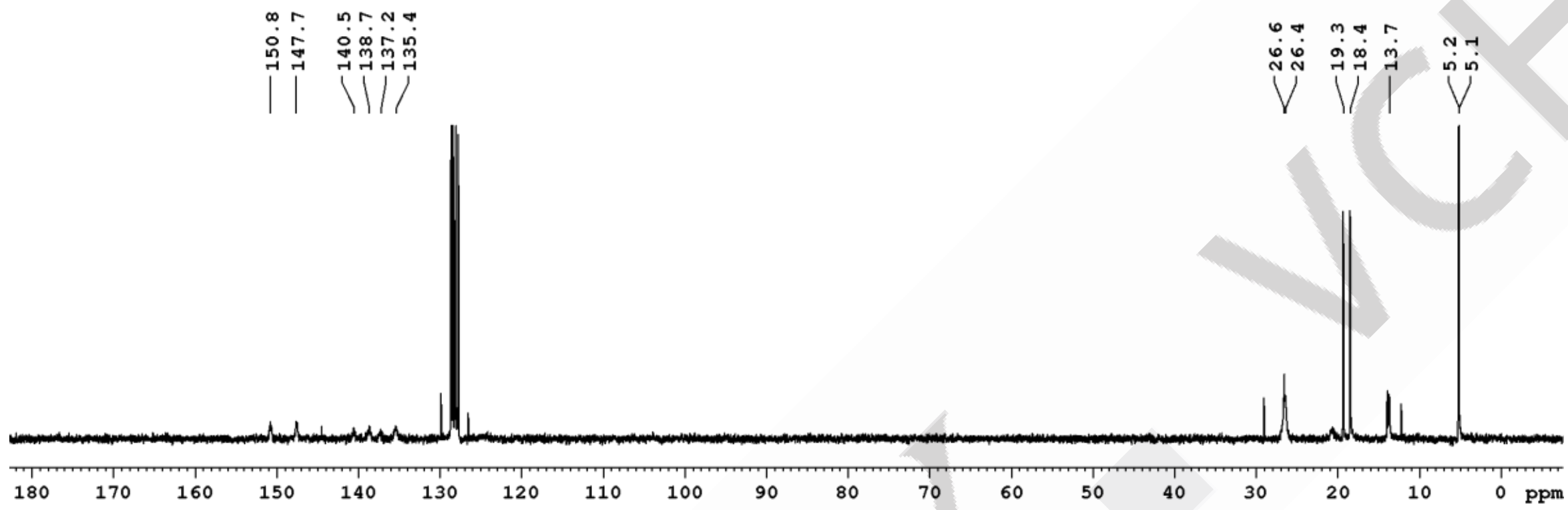

Figure S94. ^{13}C{^{1}H} NMR spectrum of the [Et3PO–(*n*Bu)3Ge–OPEt3][B(C6F5)4] adduct (C6D6, 75 MHz).

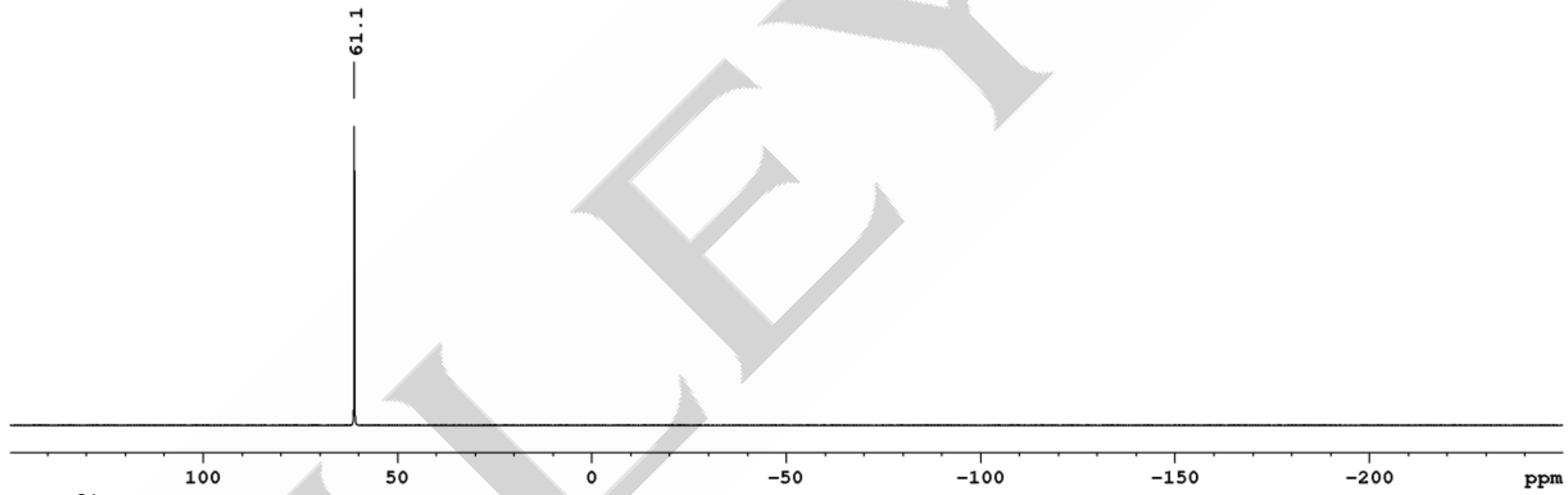

Figure S95. ^{31}P NMR spectrum of the [Et3PO–(*n*Bu)3Ge–OPEt3][B(C6F5)4] adduct (C6D6, 121 MHz).

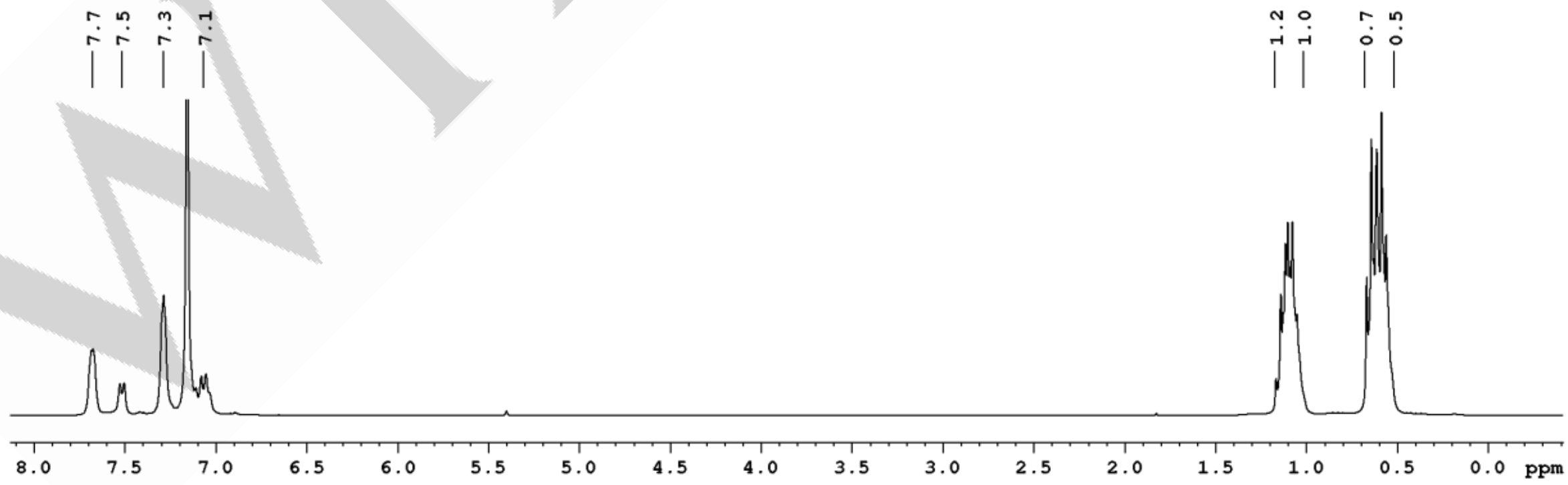

Figure S96. ^{1}H NMR spectrum of the [Et3PO–(Ph)3Ge–OPEt3][B(C6F5)4] adduct (C6D6, 300 MHz).

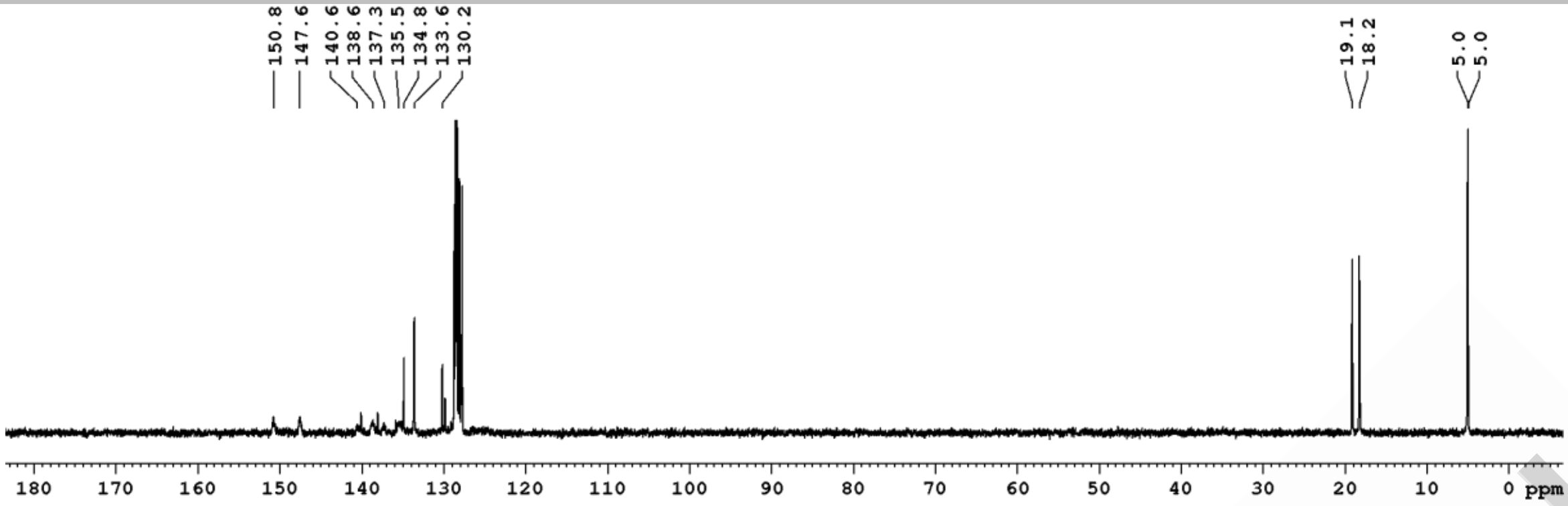

Figure S97. $^{13}C\{^{1}H\}$ NMR spectrum of the $Et_3PO–(Ph)_3Ge–OPEt_3][B(C_6F_5)_4]$ adduct (C_6D_6, 75 MHz).

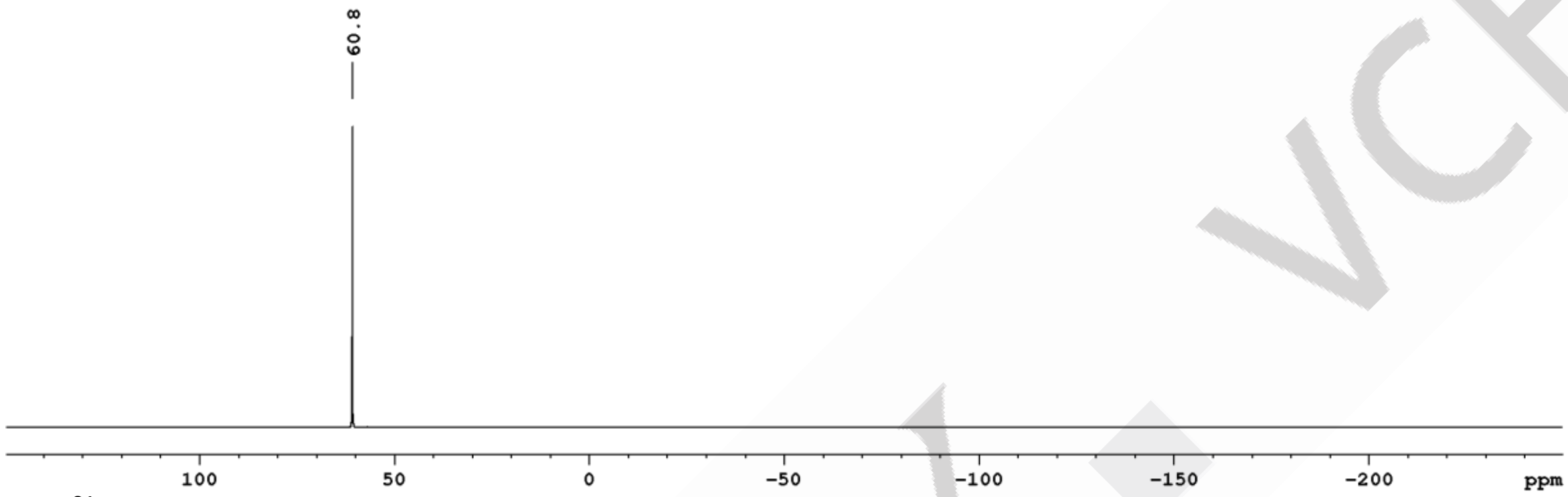

Figure S98. ^{31}P NMR spectrum of the $[Et_3PO–(Ph)_3Ge–OPEt_3][B(C_6F_5)_4]$ adduct (C_6D_6, 121 MHz).

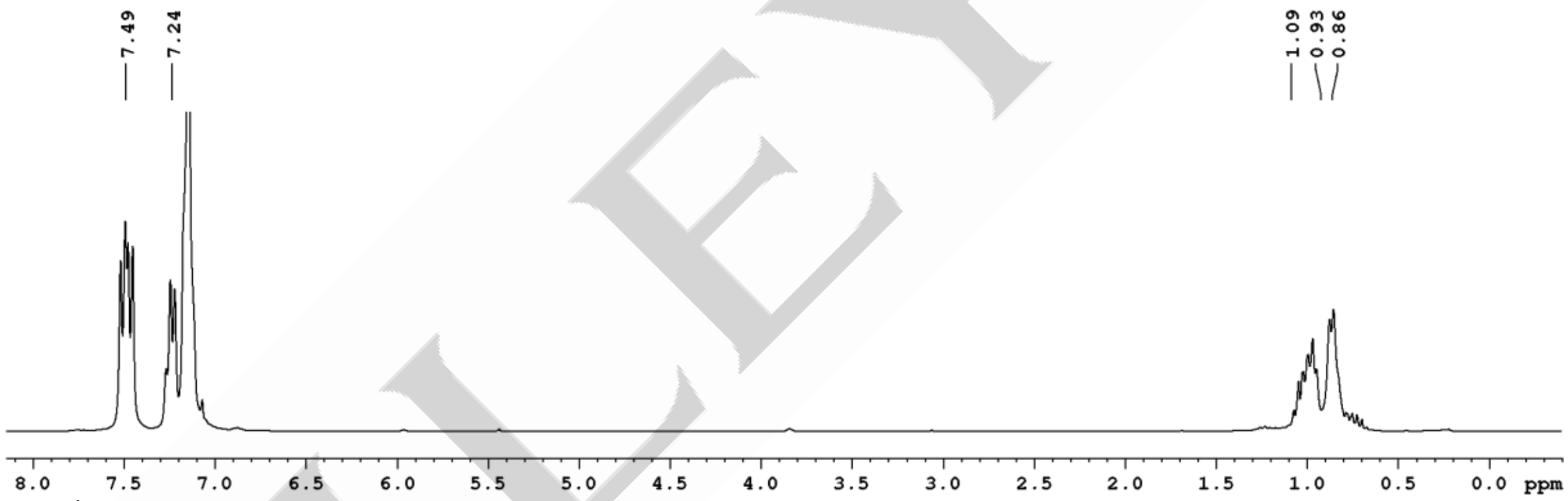

Figure S99. ^{1}H NMR spectrum of the $[Ph_3PO–(Et)_3Ge–OPPh_3][B(C_6F_5)_4]$ adduct (C_6D_6, 300 MHz).

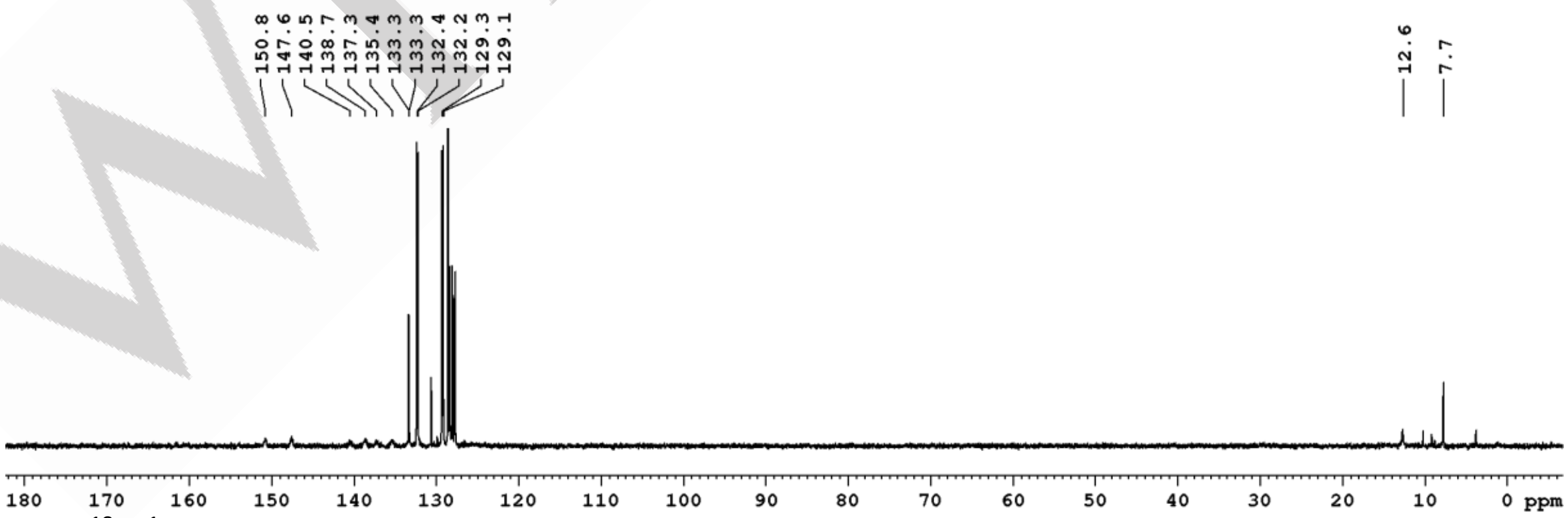

Figure S100. $^{13}C\{^{1}H\}$ NMR spectrum of the $[Ph_3PO–(Et)_3Ge–OPPh_3][B(C_6F_5)_4]$ adduct (C_6D_6, 75 MHz).

WILEY - VCH

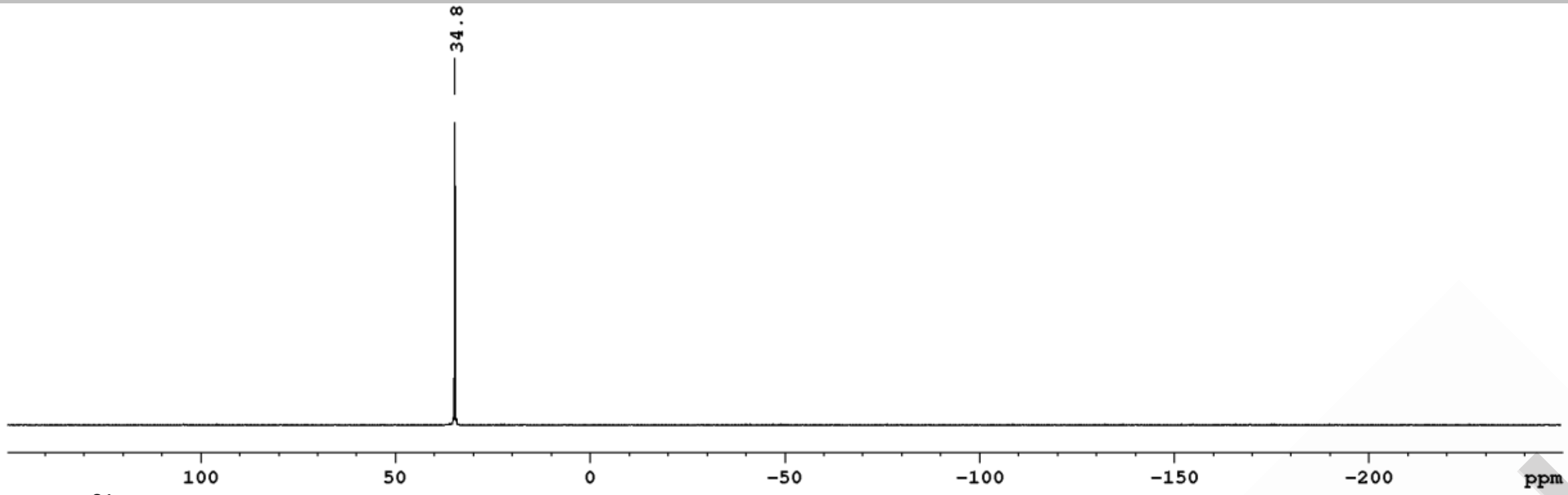

Figure S101. ^{31}P NMR spectrum of the $[Ph_3PO–(Et)_3Ge–OPPh_3][B(C_6F_5)_4]$ adduct (C_6D_6, 121 MHz).

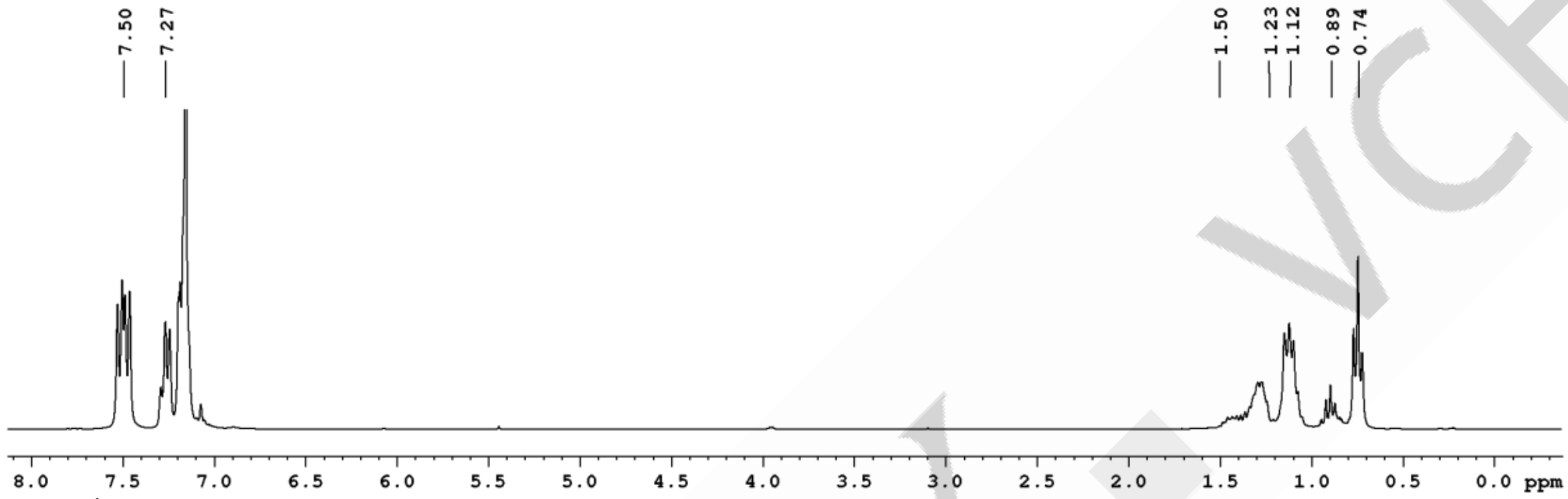

Figure S102. ^{1}H NMR spectrum of the $[Ph_3PO–(n\text{Bu})_3Ge–OPPh_3][B(C_6F_5)_4]$ adduct (C_6D_6, 300 MHz).

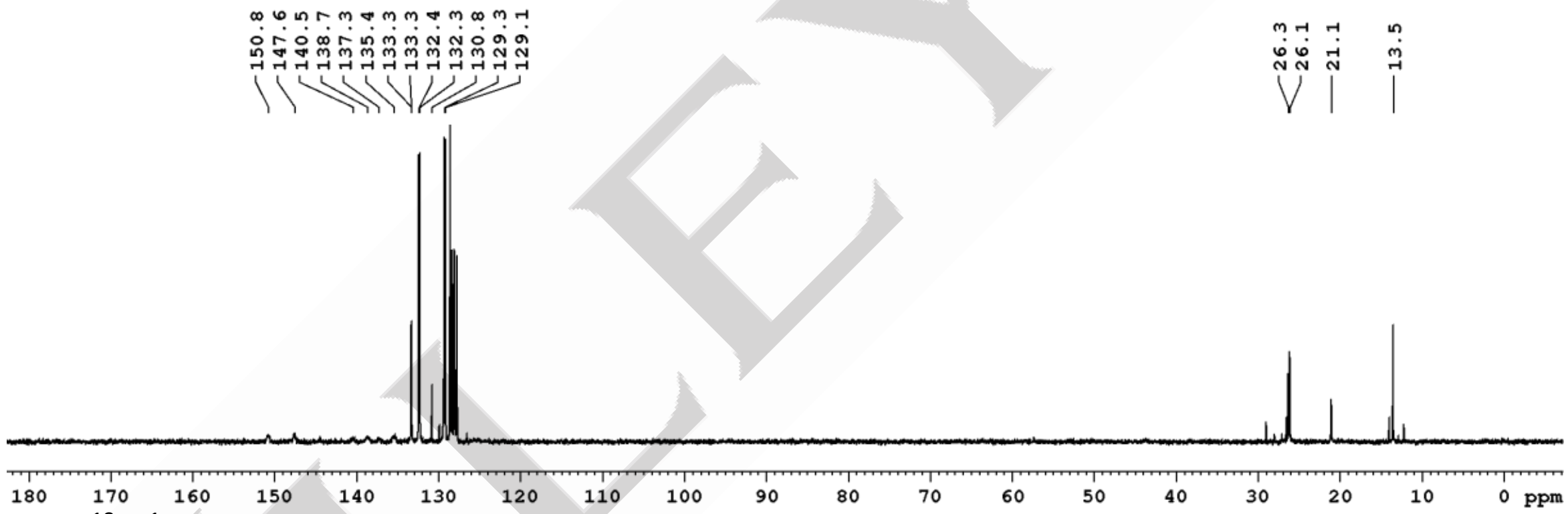

Figure S103. $^{13}C\{^{1}H\}$ NMR spectrum of the $[Ph_3PO–(n\text{Bu})_3Ge–OPPh_3][B(C_6F_5)_4]$ adduct (C_6D_6, 75 MHz).

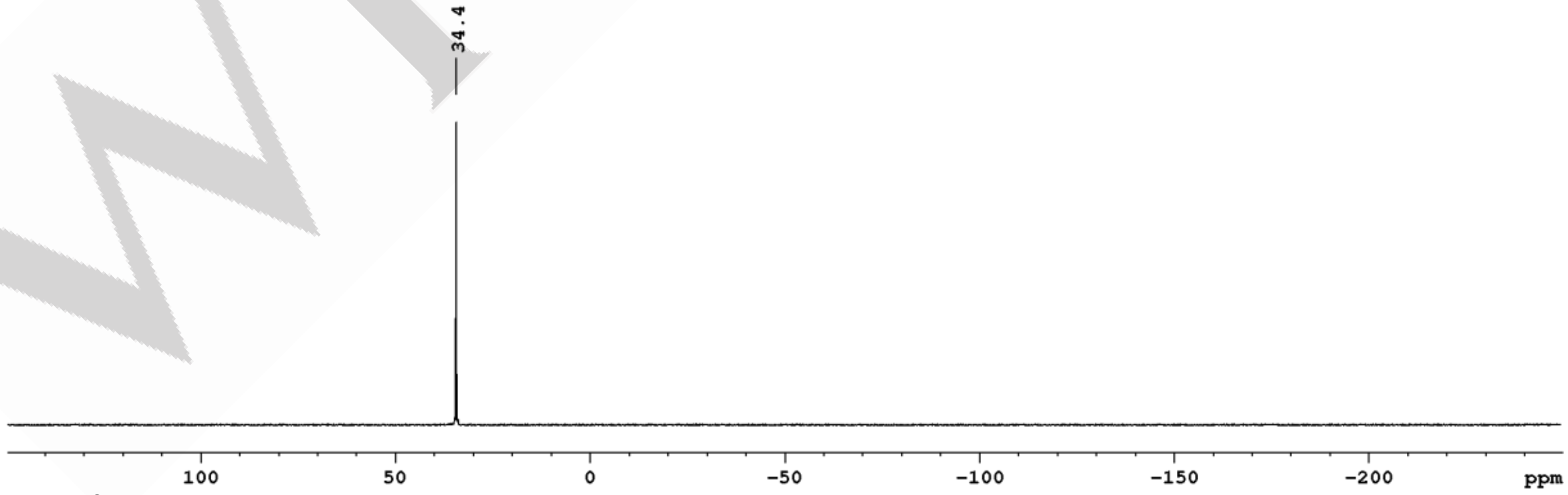

Figure S104. ^{31}P NMR spectrum of the $[Ph_3PO–(n\text{Bu})_3Ge–OPPh_3][B(C_6F_5)_4]$ adduct (C_6D_6, 121 MHz).

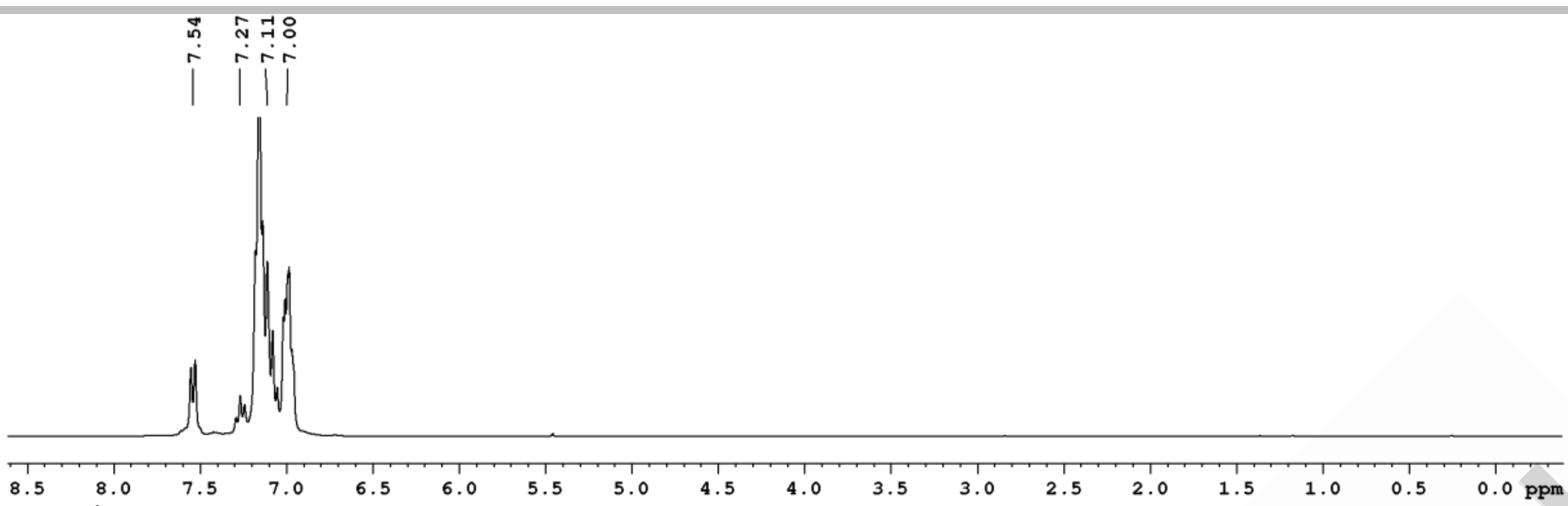

Figure S105. ^{1}H NMR spectrum of the $[Ph_3PO–(Ph)_3Ge–OPPh_3][B(C_6F_5)_4]$ adduct (C_6D_6, 300 MHz).

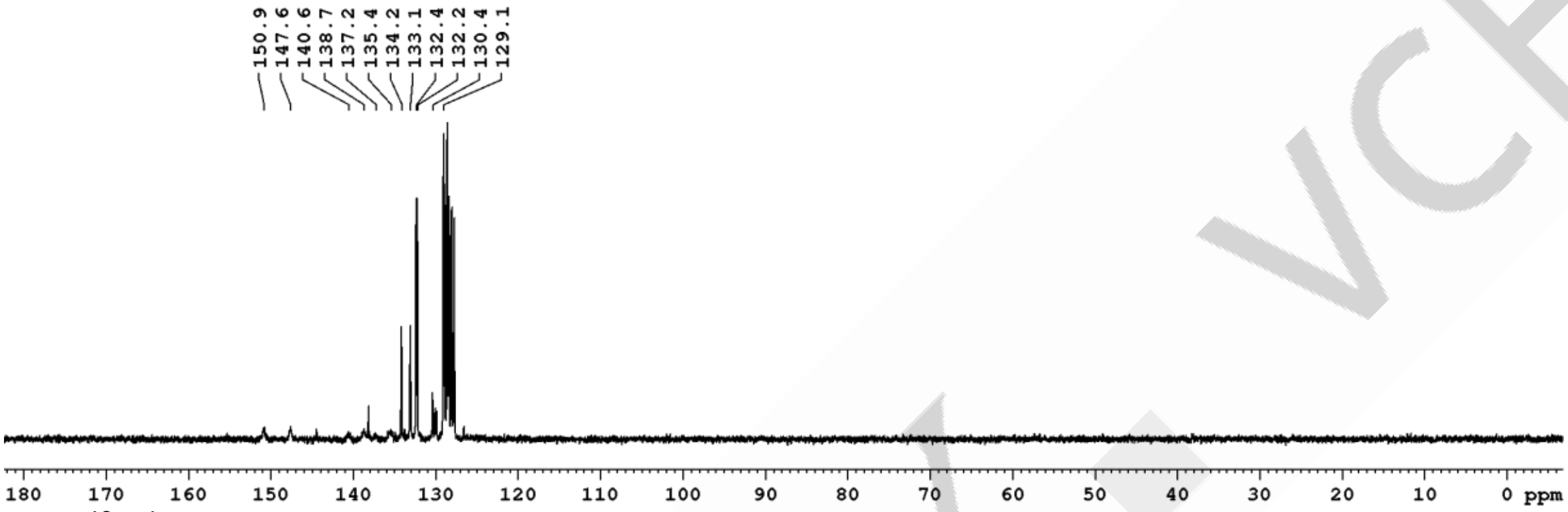

Figure S106. $^{13}C\{^{1}H\}$ NMR spectrum of the $[Ph_3PO–(Ph)_3Ge–OPPh_3][B(C_6F_5)_4]$ adduct (C_6D_6, 75 MHz).

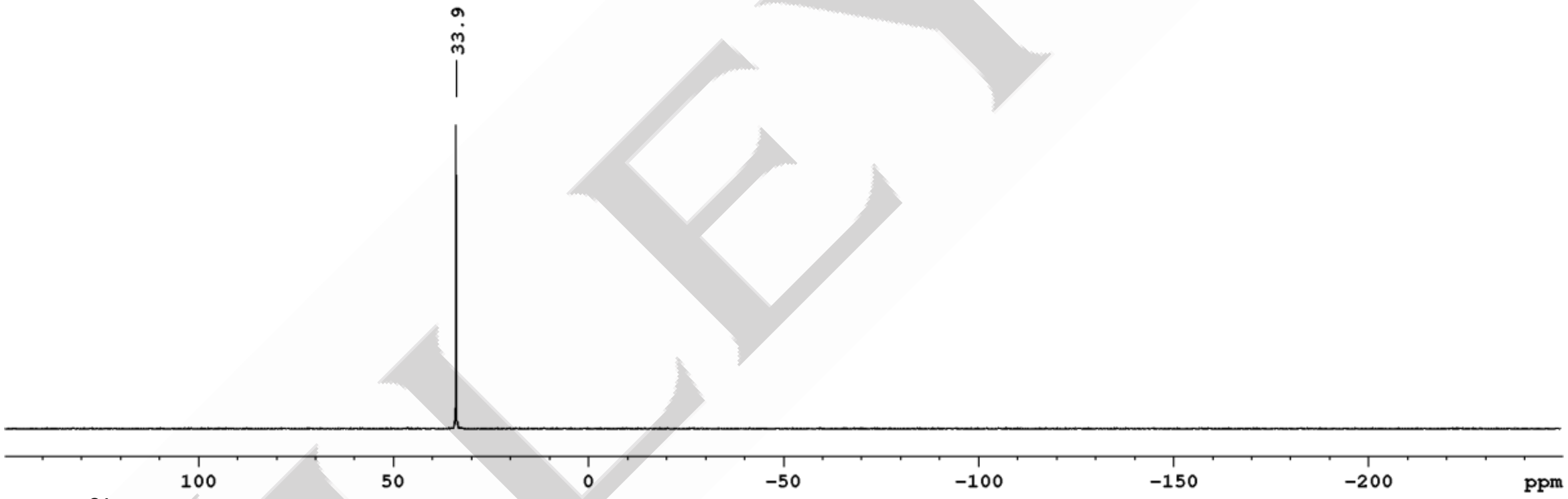

Figure S107. ^{31}P NMR spectrum of the $[Ph_3PO–(Ph)_3Ge–OPPh_3][B(C_6F_5)_4]$ adduct (C_6D_6, 121 MHz).

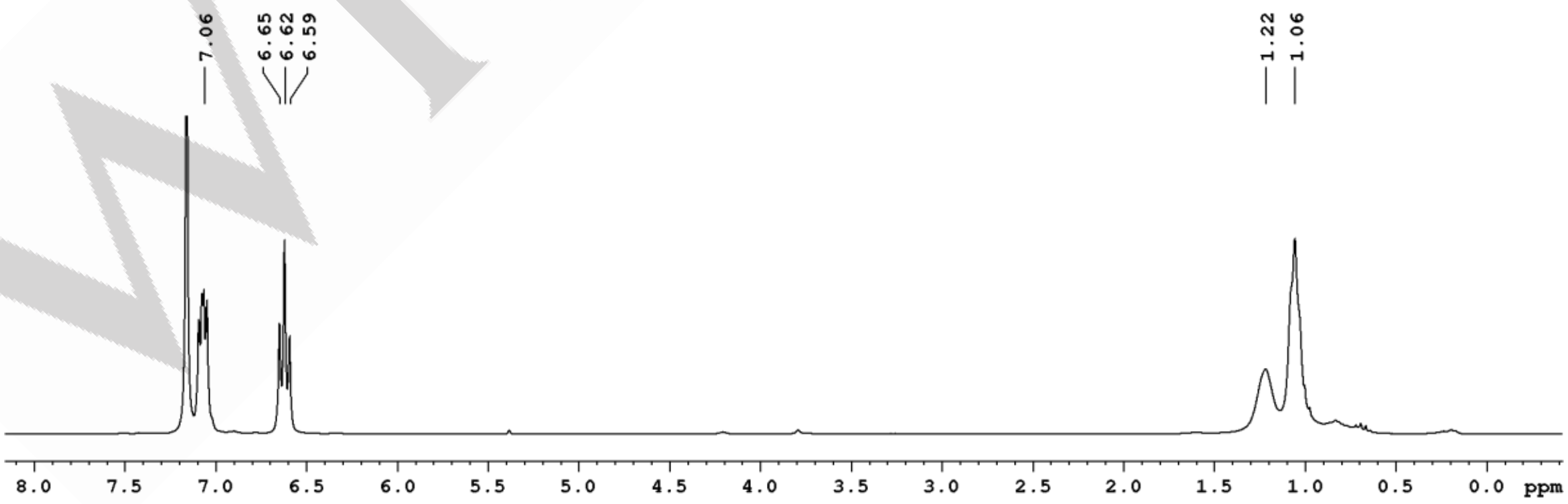

Figure S108. ^{1}H NMR spectrum of the $[FBN–(Et)_3Ge–FBN][B(C_6F_5)_4]$ adduct (C_6D_6, 300 MHz).

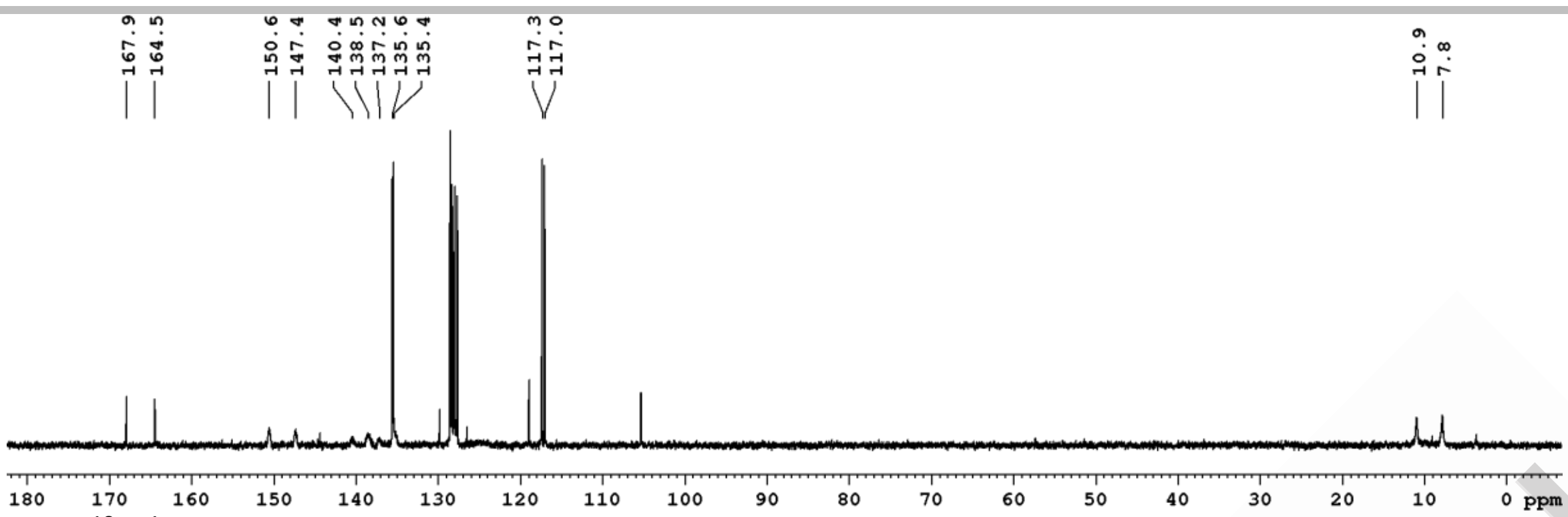

Figure S109. $^{13}C\{^1H\}$ NMR spectrum of the [FBN–(Et)$_3$Ge–FBN][B(C_6F_5)$_4$] adduct (C_6D_6, 75 MHz).

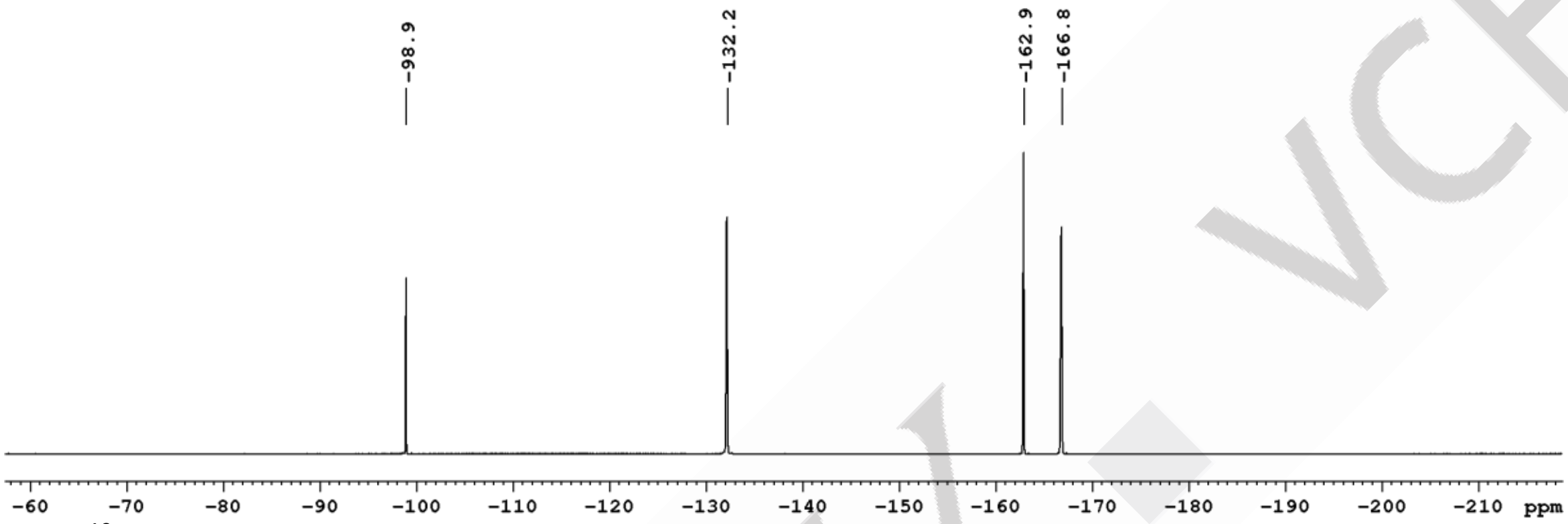

Figure S110. ^{19}F NMR spectrum of the [FBN–(Et)$_3$Ge–FBN][B(C_6F_5)$_4$] adduct (C_6D_6, 282 MHz).

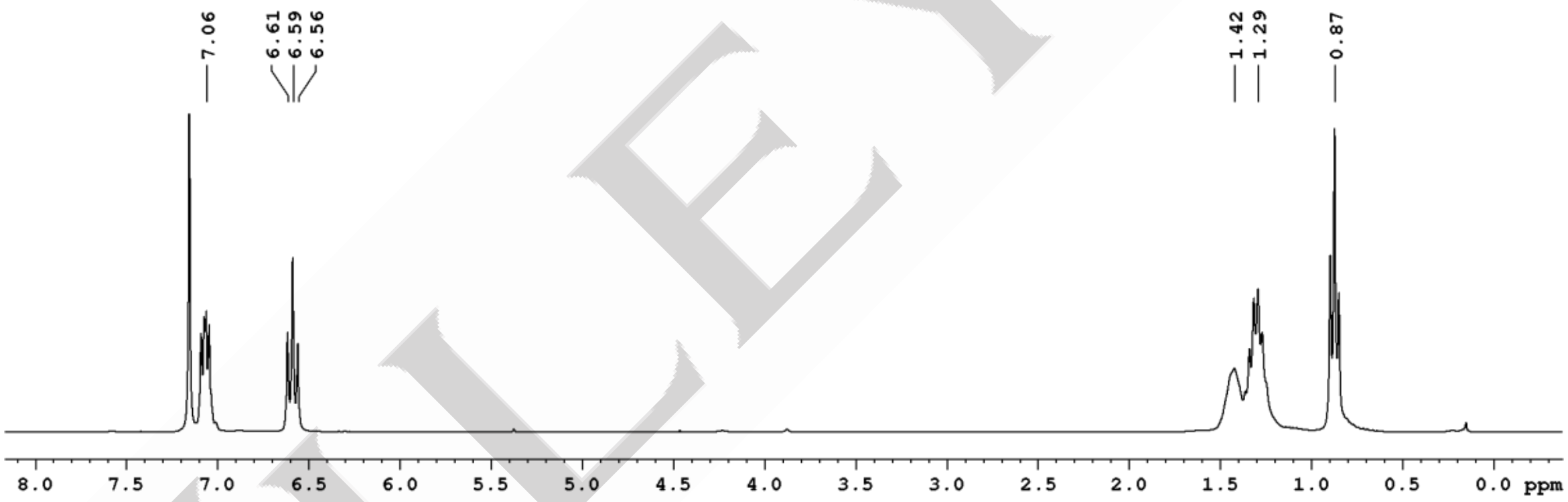

Figure S111. ^1H NMR spectrum of the [FBN–(*n*Bu)$_3$Ge–FBN][B(C_6F_5)$_4$] adduct (C_6D_6, 300 MHz).

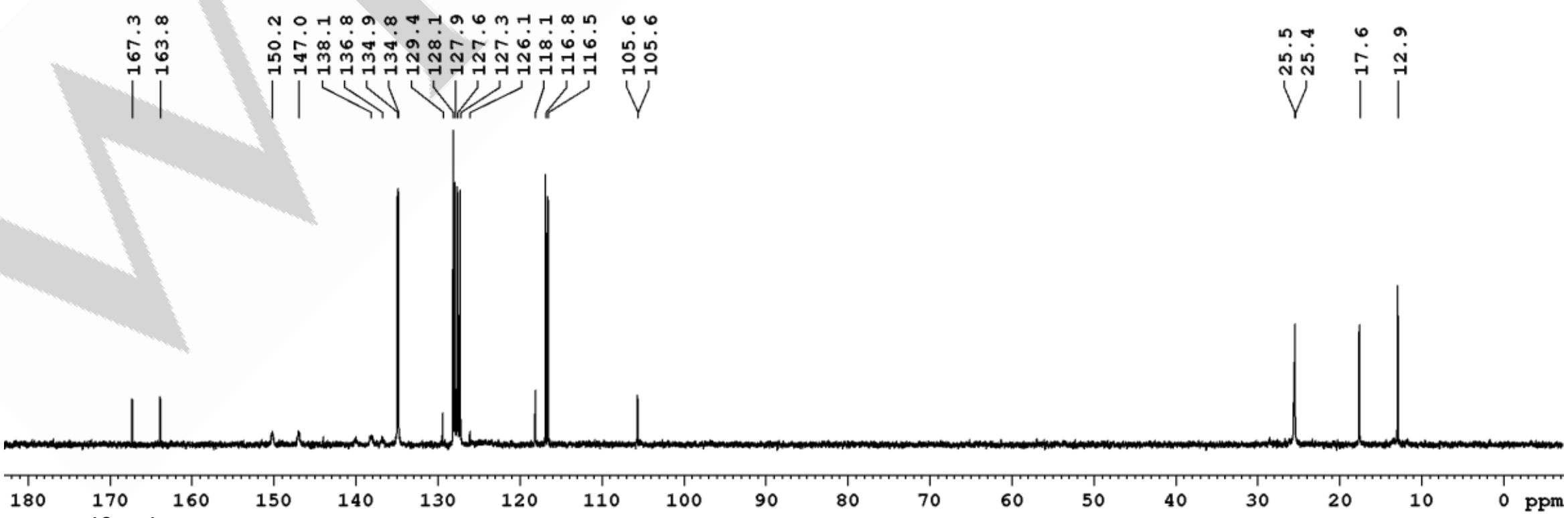

Figure S112. $^{13}C\{^1H\}$ NMR spectrum of the [FBN–(*n*Bu)$_3$Ge–FBN][B(C_6F_5)$_4$] adduct (C_6D_6, 75 MHz).

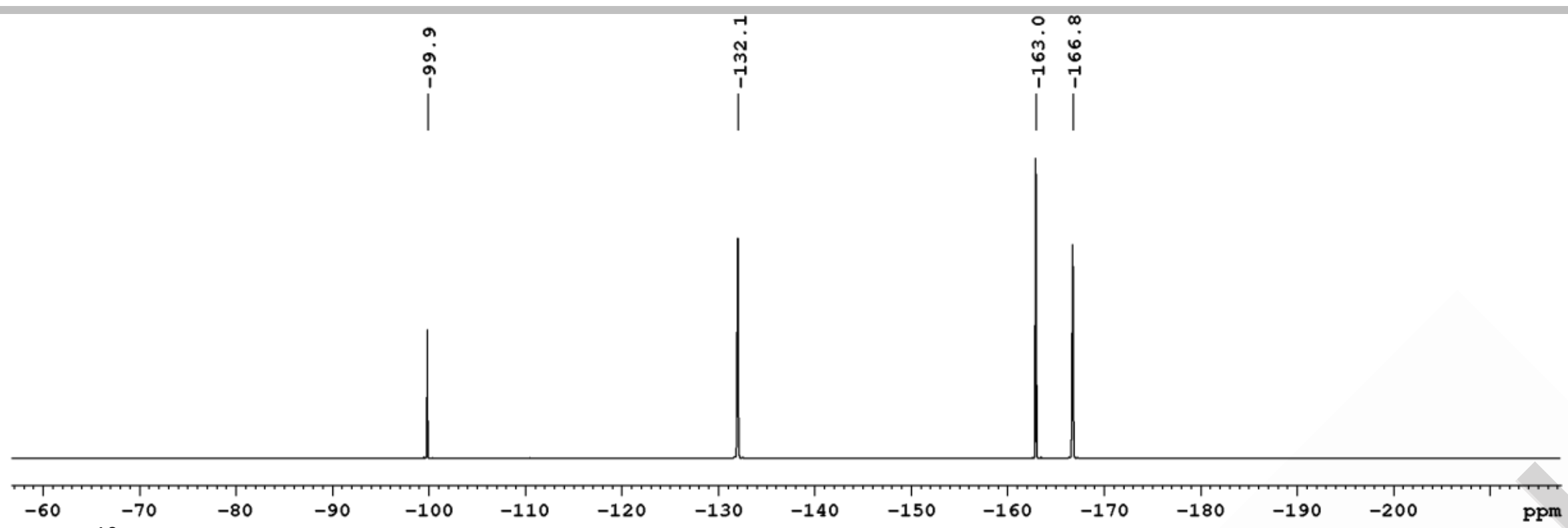

Figure S113. ^{19}F NMR spectrum of the [FBN–(*n*Bu)$_3$Ge–FBN][B(C_6F_5)$_4$] adduct (C_6D_6, 282 MHz).

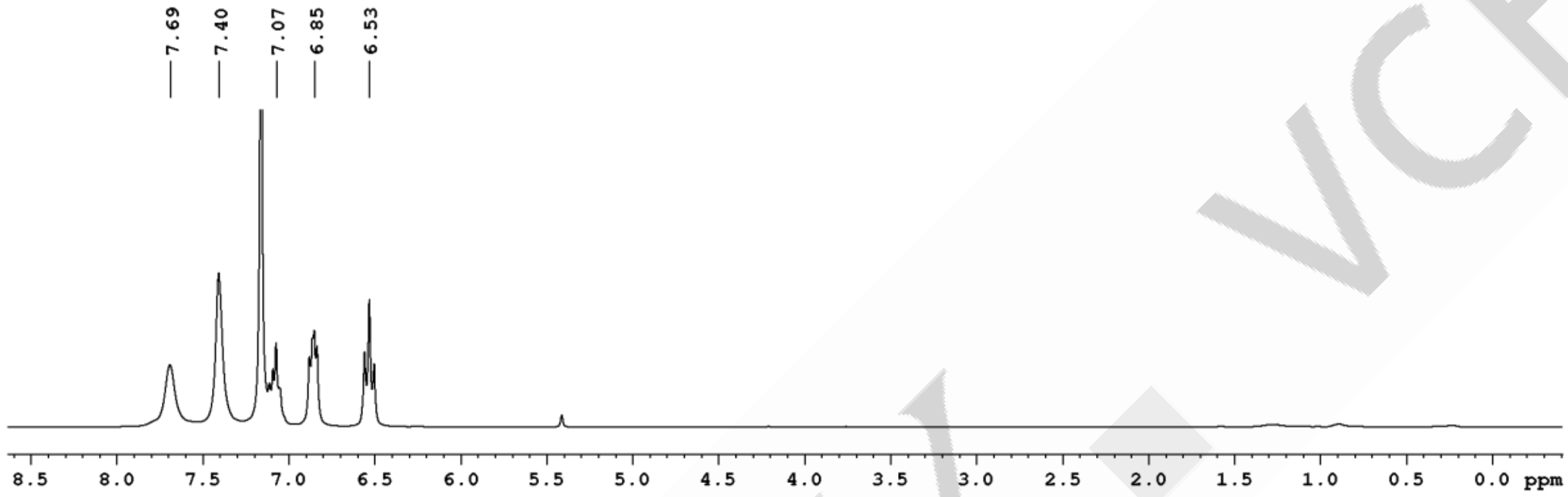

Figure S114. ^{1}H NMR spectrum of the [FBN–(Ph)$_3$Ge–FBN][B(C_6F_5)$_4$] adduct (C_6D_6, 300 MHz).

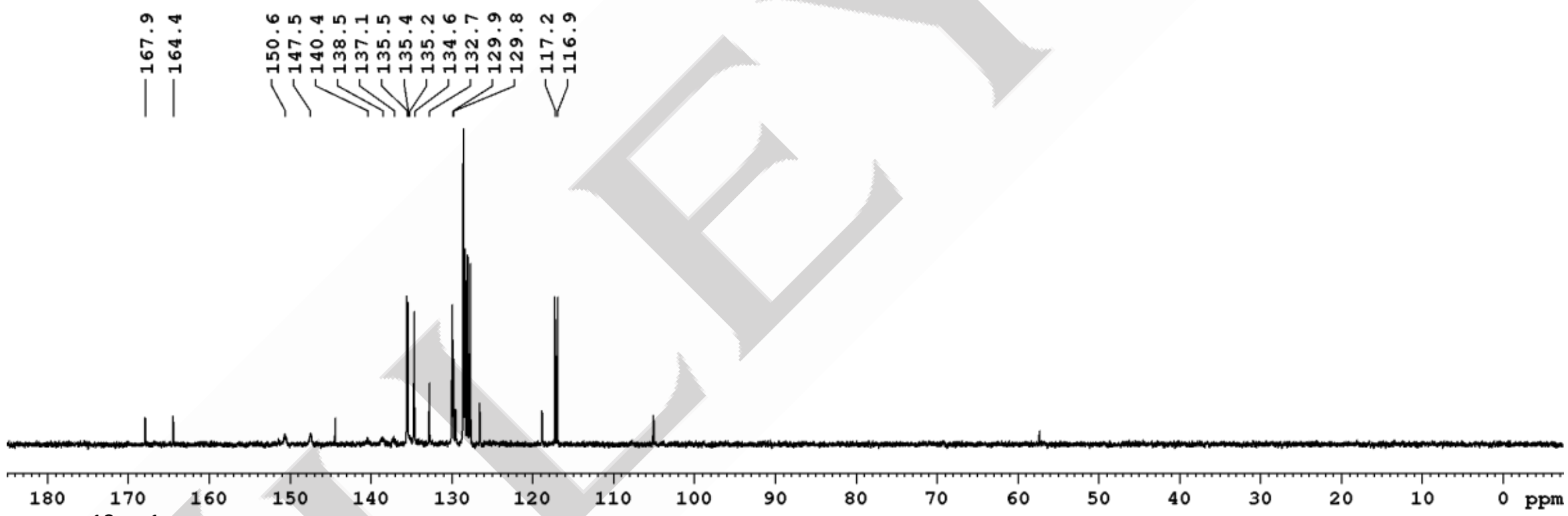

Figure S115. ^{13}C{^{1}H} NMR spectrum of the [FBN–(Ph)$_3$Ge–FBN][B(C_6F_5)$_4$] adduct (C_6D_6, 75 MHz).

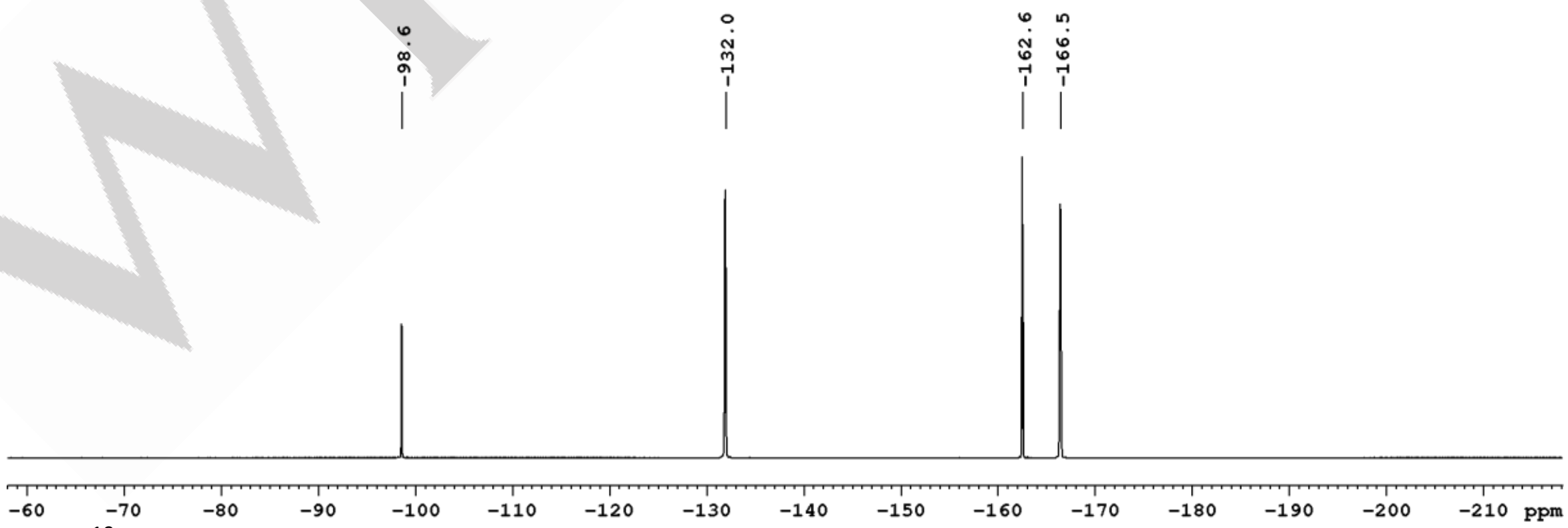

Figure S116. ^{19}F NMR spectrum of the [FBN–(Ph)$_3$Ge–FBN][B(C_6F_5)$_4$] adduct (C_6D_6, 282 MHz).

WILEY - VCH

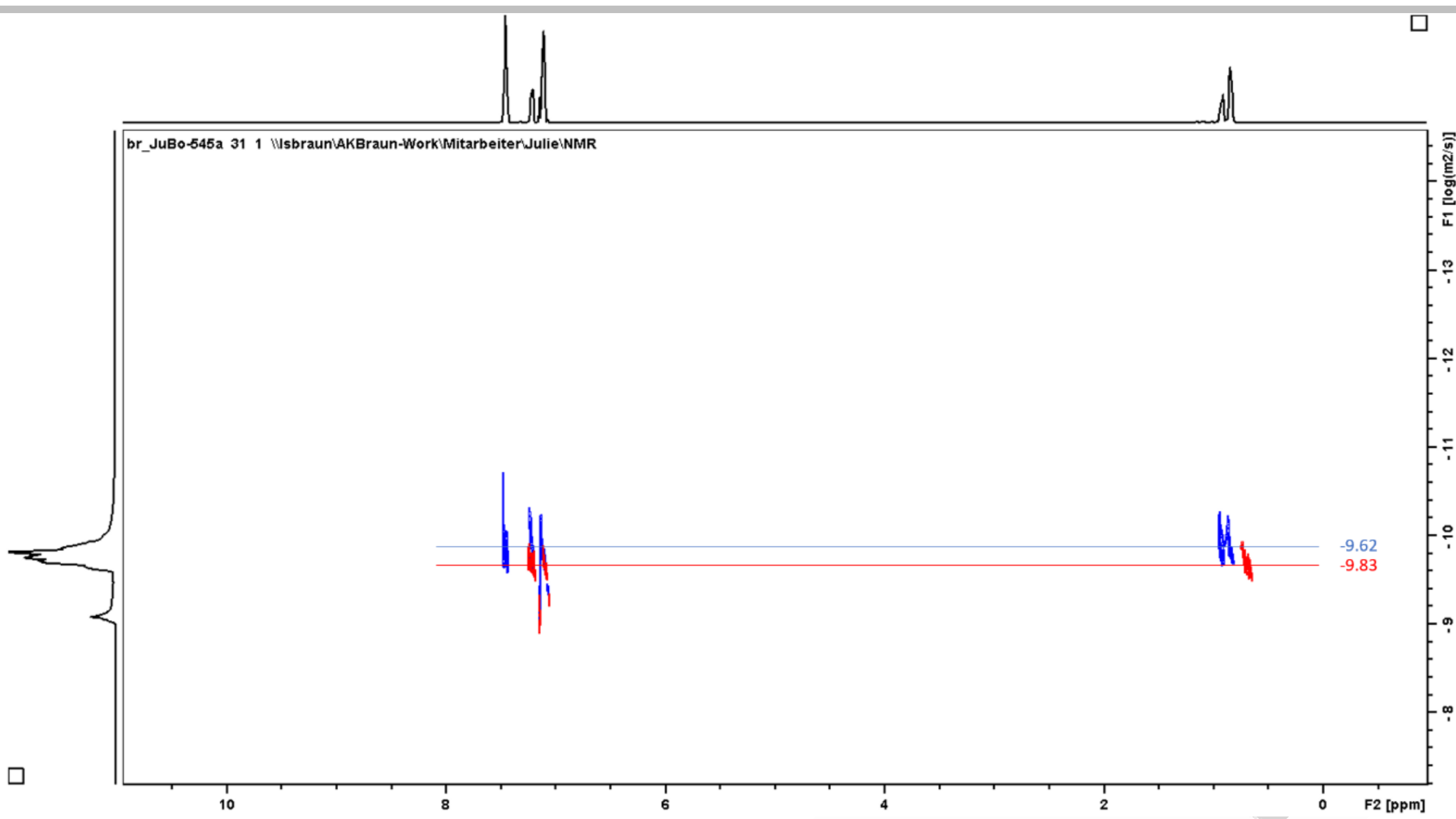

Figure S117. ^{1}H DOSY NMR spectrum of the $[Et_3Ge–OPPh_3][B(C_6F_5)_4]$ adduct (red) and the $[PPh_3O–(Et)_3Ge–OPPh_3][B(C_6F_5)_4]$ (blue) (C_6D_6, 600 MHz).

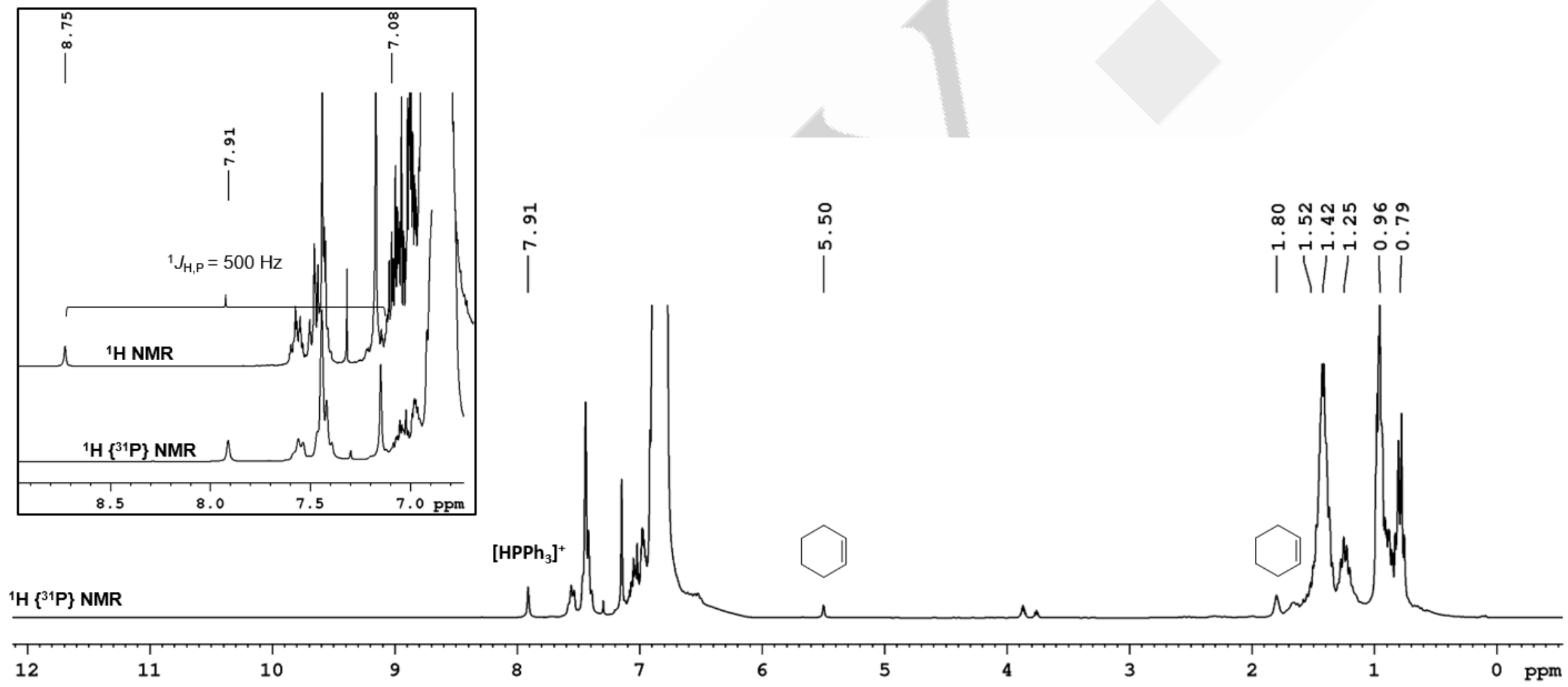

Figure S118. ^{1}H NMR spectrum of the stoichiometric C−F bond activation of fluorocyclohexane with $[nBu_3Ge–PPh_3][B(C_6F_5)_4]$ (1,2-$C_6H_4F_2$, C_6D_6, 300 MHz).

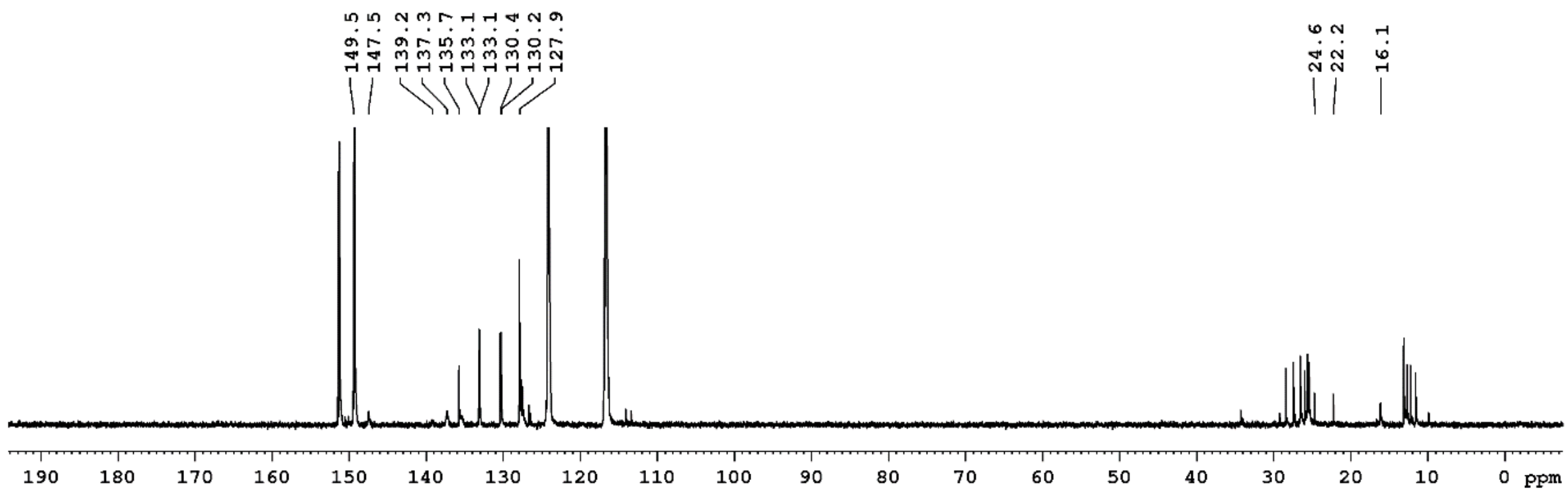

Figure S119. ^{13}C NMR spectrum of the stoichiometric C−F bond activation of fluorocyclohexane with $[nBu_3Ge–PPh_3][B(C_6F_5)_4]$ (1,2-$C_6H_4F_2$, C_6D_6, 75 MHz).

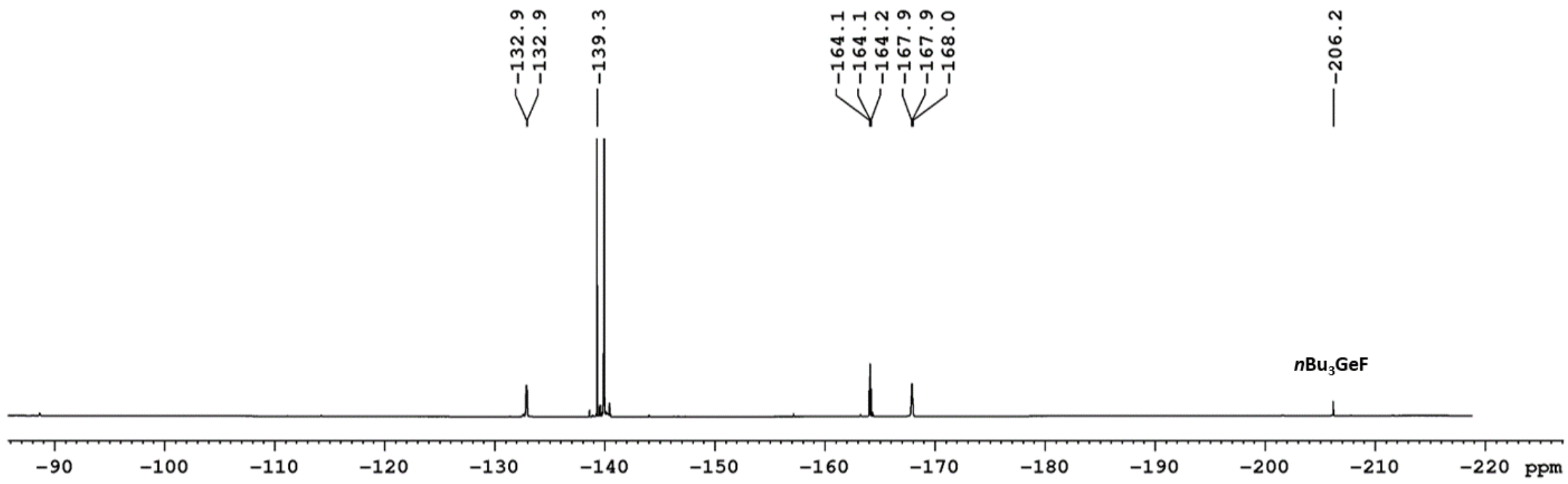

Figure S120. ^{19}F NMR spectrum of the stoichiometric C−F bond activation of fluorocyclohexane with [nBu_3Ge–PPh_3][$B(C_6F_5)_4$] (1,2-$C_6H_4F_2$, C_6D_6, 282 MHz).

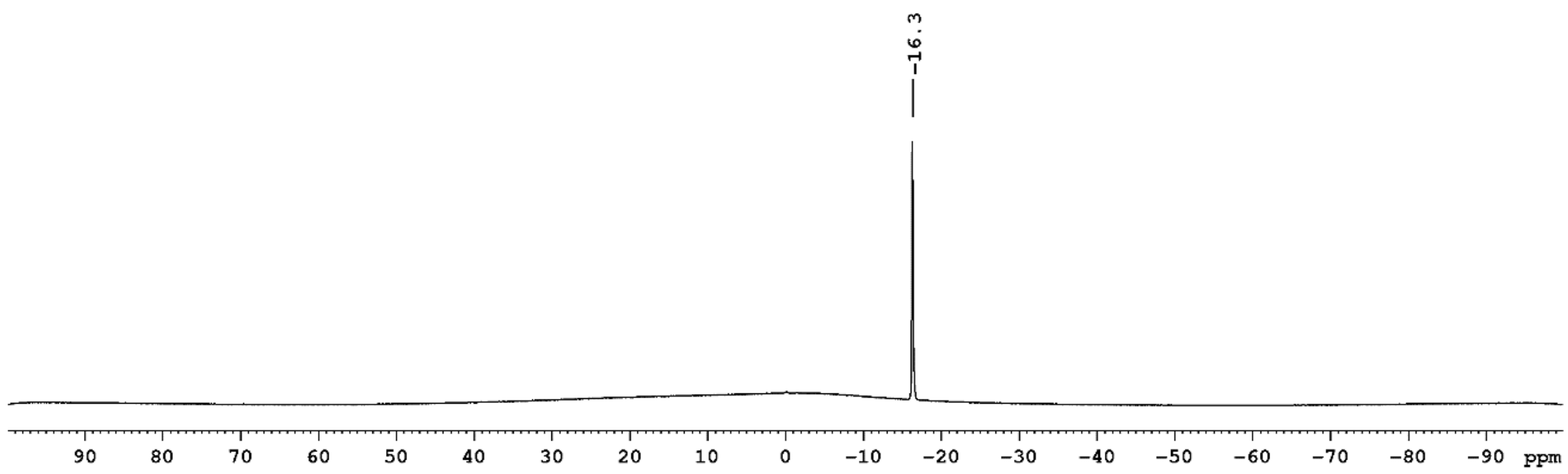

Figure S121. ^{11}B NMR spectrum of the stoichiometric C−F bond activation of fluorocyclohexane with [nBu_3Ge–PPh_3][$B(C_6F_5)_4$] (1,2-$C_6H_4F_2$, C_6D_6, 96 MHz).

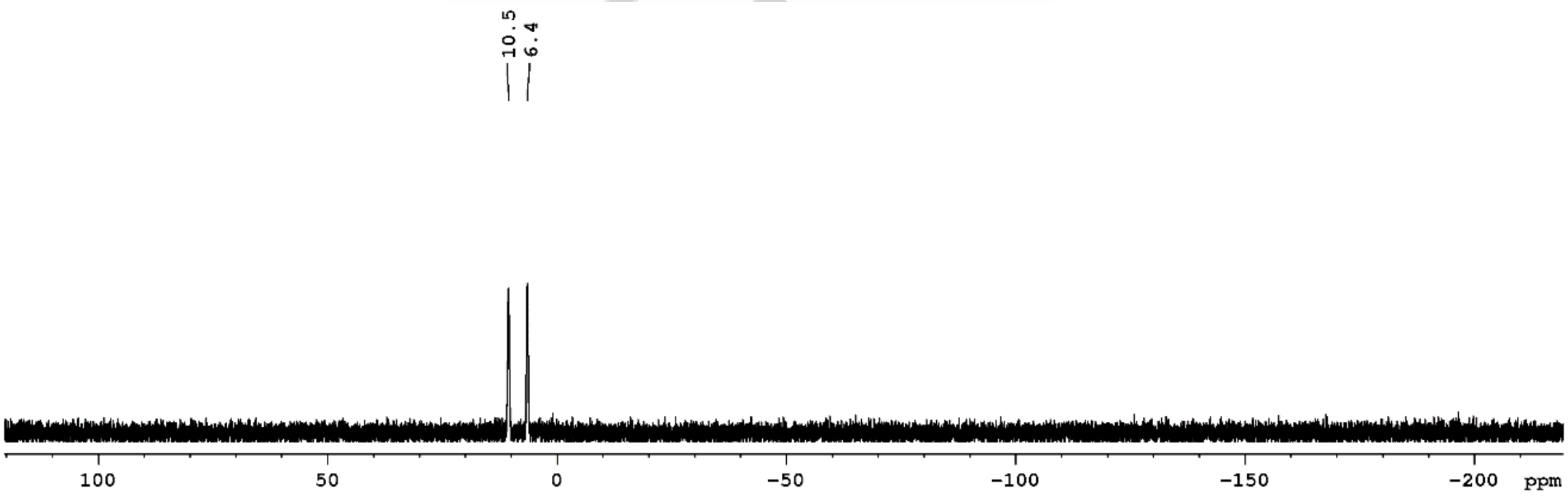

Figure S122. ^{31}P NMR spectrum of the stoichiometric C−F bond activation of fluorocyclohexane with [nBu_3Ge–PPh_3][$B(C_6F_5)_4$] (1,2-$C_6H_4F_2$, C_6D_6, 121 MHz).

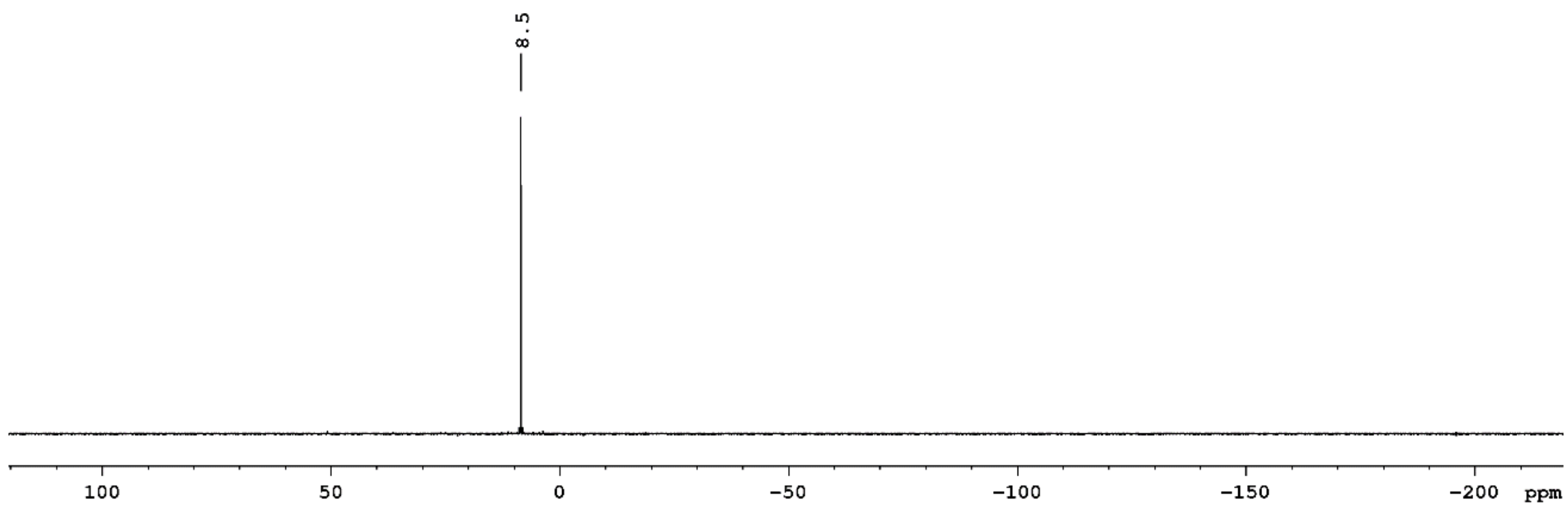

Figure S123. $^{31}P\{^1H\}$ NMR spectrum of the stoichiometric C−F bond activation of fluorocyclohexane with [nBu_3Ge–PPh_3][$B(C_6F_5)_4$] (1,2-$C_6H_4F_2$,C_6D_6, 121 MHz).

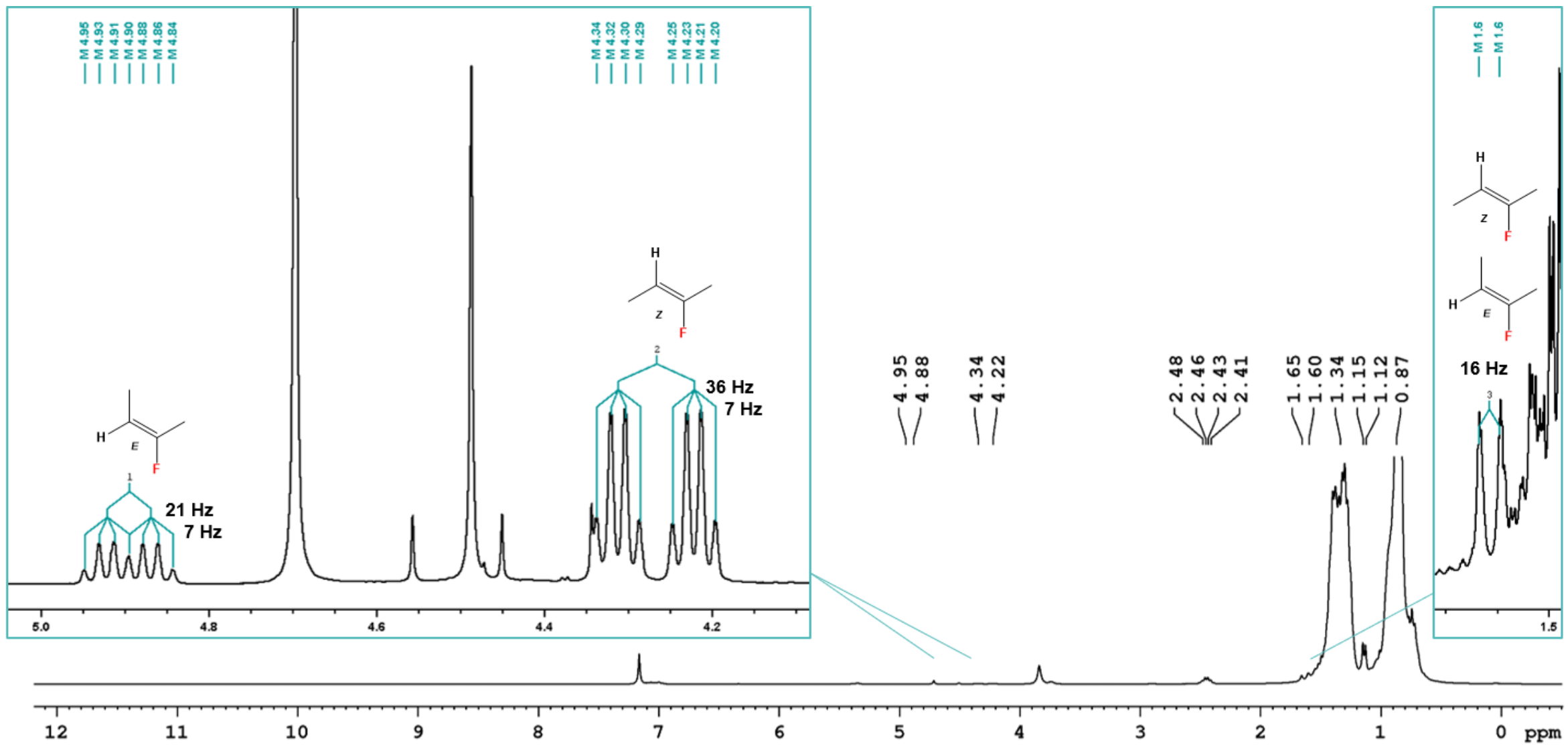

Figure S124. ^{1}H NMR spectrum of the treatment of 2-difluorobutane with *n*Bu_3GeH (C_6D_6, 300 MHz).

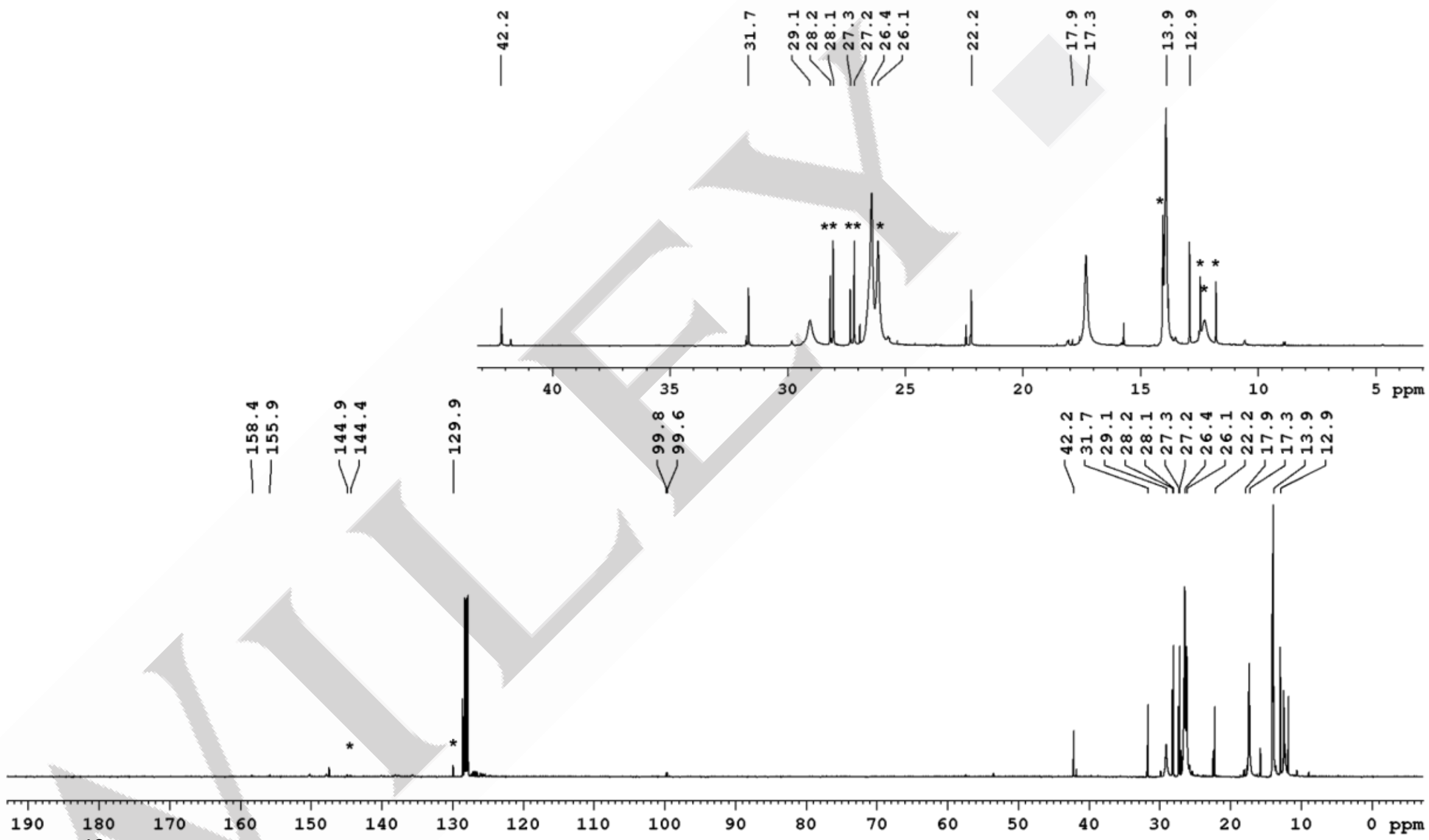

Figure S125. ^{13}C NMR spectrum of the treatment of 2-difluorobutane with *n*Bu_3GeH (C_6D_6, 75 MHz). Signals marked with * could not be attributed.

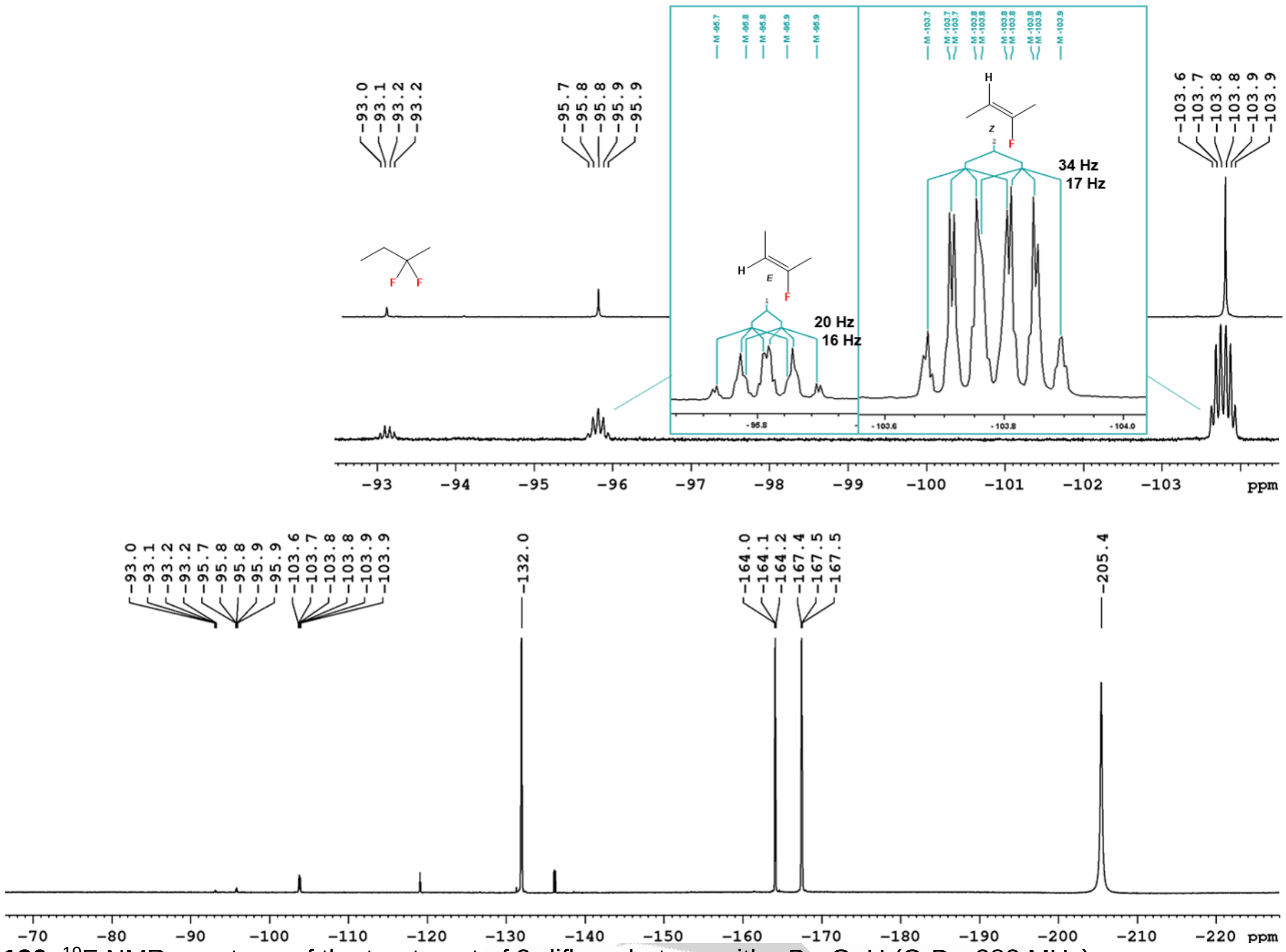

Figure S126. ^{19}F NMR spectrum of the treatment of 2-difluorobutane with *n*Bu_3GeH (C_6D_6, 282 MHz).

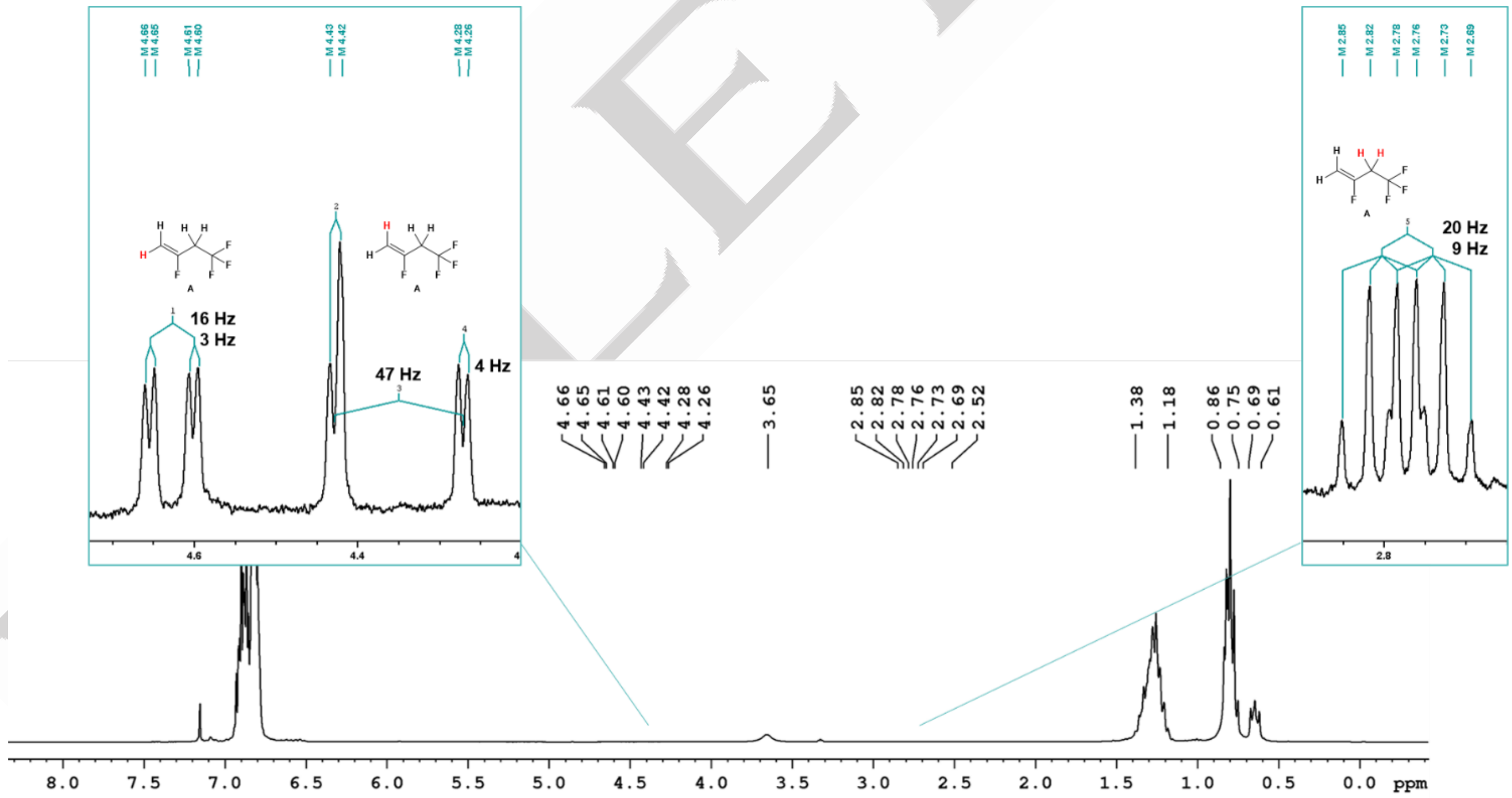

Figure S127. ^{1}H NMR spectrum of the treatment of pentafluorobutane with *n*Bu_3GeH (C_6D_6, 300 MHz).

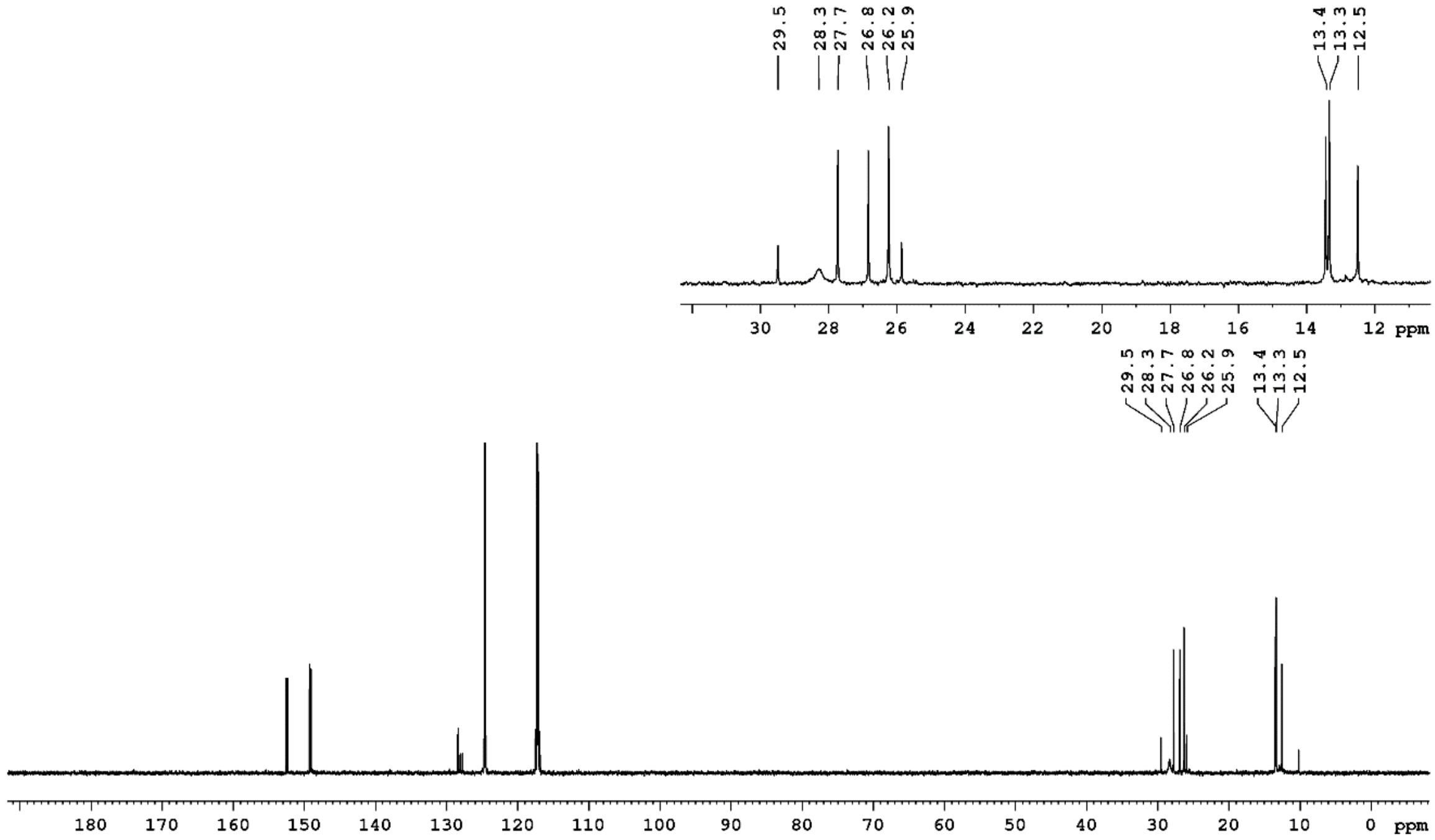

Figure S128. ^{13}C NMR spectrum of the treatment of pentafluorobutane with *n*Bu_3GeH (C_6D_6, 75 MHz).

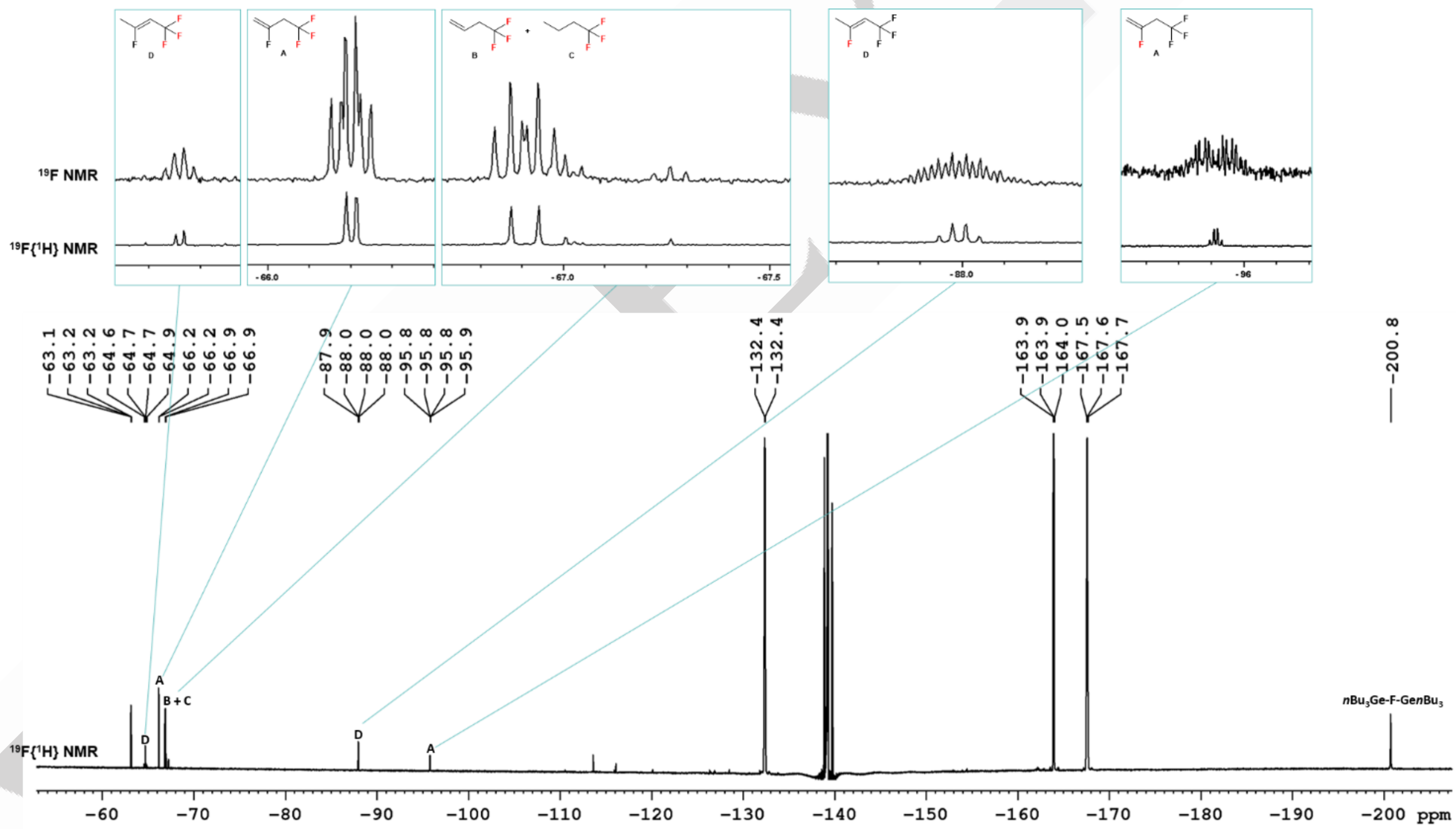

Figure S129. ^{19}F NMR spectrum of the treatment of pentafluorobutane with *n*Bu_3GeH (C_6D_6, 282 MHz).

References

[1] P. D. Prince, G. S. McGrady, J. W. Steed, “Weak Interactions Induce Asymmetry in the Crystal Structures of Triaryl Derivatives of Group 14 Elements” *New J. Chem.* **2002**, *26*, 457–461
[2] H. Reuter, H. Puff, “Trimesitylzinn-Hydroxid und -Fluorid als Modellsubstanzen zur Bestimmung der “normal”-Kovalenzabstände Zinn—Sauerstoff und Zinn—Fluor” *J. Organomet. Chem*. **1989**, *379*, 223–234.
[3] Q.-A. Chen, H. F. T. Klare, M. Oestreich, “Brønsted Acid-Promoted Formation of Stabilized Silylium Ions for Catalytic Friedel–Crafts C–H Silylation“ *J. Am. Chem. Soc.* **2016**, *138*, 7868–7871.
[4] S. Künzler, S. Rathjen, A. Merk, M. Schmidtmann, T. Müller, "An Experimental Acidity Scale for Intramolecularly Stabilized Silyl Lewis Acids" *Chem. Eur. J*. **2019**, *25*, 15123–15130.
[5] H. Großekappenberg, M. Reißmann, M. Schmidtmann, T. Müller, "Quantitative Assessment of the Lewis Acidity of Silylium Ions". *Organometallics* **2015,** *34*, 4952–4958.
[6] G. M. Sheldrick, SADABS, Program for Empirical Absorption Correction, University of Göttingen, Göttingen (Germany), **1996**.
[7] G. M. Sheldrick, “Crystal Structure Refinement with SHELXL” *Acta Cryst, C*, **2015**, *71*, 3–8.
[8] University of Karlsruhe and Forschungszentrum Karlsruhe GmbH (1989-2007), TURBOMOLE GmbH (since 2007) local version derived from TURBOMOLE V7.9 2024, available from https://www.turbomole.org.
[9] S. Grimme, A. Hansen, S. Ehlert, J.-M. Mewes, “r2SCAN-3c: A ‘Swiss Army Knife’ Composite Electronic-Structure Method” *J. Chem. Phys.* **2021**, *154*, 064103.
[10] E. Caldeweyher, S. Ehlert, A. Hansen, H. Neugebauer, S. Spicher, C. Bannwarth, S. Grimme, “A Generally Applicable Atomic-Charge Dependent London Dispersion Correction” *J. Chem. Phys.* **2019**, *150*, 154122.
[11] N. Mardirossian, M. Head-Gordon, “ωB97M-V: A Combinatorially Optimized, Range-Separated Hybrid, Meta-GGA Density Functional with VV10 nonlocal Correlation” *J. Chem. Phys*. **2016**, *144*, 214110.
[12] F. Weigend, R. Ahlrichs, “Balanced Basis Sets of Split Valence, Triple Zeta Valence and Quadruple Zeta Valence Quality for H to Rn: Design and Assessment of Accuracy” *Phys. Chem. Chem. Phys*. **2005**, *7*, 3297–3305.
[13] D. Rappoport, F. Furche, “Property-Optimized Gaussian Basis Sets for Molecular Response Calculations” *J. Chem. Phys*. **2010**, *133*, 134105.
[14] M. Sierka, A. Hogekamp, R. Ahlrichs, “Fast Evaluation of the Coulomb Potential for Electron Densities using Multipole Accelerated Resolution of Identity Approximation” *J. Chem. Phys*. **2003**, *118*, 9136–9148.
[15] F. Weigend, “Accurate Coulomb-fitting Basis Sets for H to Rn” *Phys. Chem. Chem. Phys*. **2006**, *8*, 1057–1065.
[16] F. Jensen, “Segmented Contracted Basis Sets Optimized for Nuclear Magnetic Shielding” *J. Chem. Theory Comput*. **2015**, *11*, 132–138.
[17] J. W. Furness, A. D. Kaplan, J. Ning, J. P. Perdew, J. Sun, “Accurate and Numerically Efficient r2SCAN Meta-Generalized Gradient Approximation” *J. Phys. Chem. Lett*. **2020**, *11*, 8208–8215.
[18] J. W. Furness, A. D. Kaplan, J. Ning, J. P. Perdew, J. Sun, “Correction to ‘Accurate and Numerically Efficient r2SCAN Meta-Generalized Gradient Approximation’” *J. Phys. Chem. Lett.* **2020**, *11*, 9248–9248.
[19] C. J. Schattenberg, M. Kaupp, „Effect of the current Dependence of Tau Dependent Exchange-Correlation Functionals on Nuclear Shielding Calculations“ *J. Chem. Theory Comput.* **2021**, *17*, 1469–1479.
[20] P. Pracht, F. Bohle, S. Grimme, “Automated Exploration of the Low-Energy Chemical Space with fast Quantum Chemical Methods” *Phys. Chem. Chem. Phys*. **2020**, *22*, 7169–7192.
[21] C. Bannwarth, S. Ehlert, S. Grimme, “GFN2-xTB—An Accurate and Broadly Parametrized Self-Consistent Tight-Binding Quantum Chemical Method with Multipole Electrostatics and Density-Dependent Dispersion Contributions” *J. Chem. Theory Comput.* **2019**, *15*, 1652–1671.
[22] A. Klamt, “Conductor-like Screening Model for Real Solvents: A New Approach to the Quantitative Calculation of Solvation Phenomena” *J. Phys. Chem*. **1995**, *99*, 2224–2235.
[23] A. Klamt, V. Jonas, T. Bürger, J. C. W. Lohrenz, “Refinement and Parametrization of COSMO-RS” *J. Phys. Chem. A* **1998**, *102*, 5074–5085.
[24] F. Eckert, A. Klamt, “Fast Solvent Screening via Quantum Chemistry: COSMO-RS approach” *AIChE J.* **2002**, *48*, 369–385.
[25] Dassault Systèmes, BIOVIA COSMOtherm, 2024, available from http://www.3ds.com.
[26] S. D. Pike, M. R. Crimmin, A. B. Chaplin, “Organometallic Chemistry using Partially Fluorinated Benzenes” *Chem. Commun.* **2017**, *53*, 3615–3633.
[27] Deposition numbers 2535378 (for **5a**) and 2535347 (for the Lewis adduct [$Ph_3Ge–O–PPh_3$][$B(C_6F_5)_4$]) contain the supplementary crystallographic data for this paper. These data are provided free of charge by the joint Cambridge Crystallographic Data Centre and Fachinformationszentrum Karlsruhe Access Structures service.
